# Use of Expectation Disconfirmation Theory to Test Patient Satisfaction with Asynchronous Telemedicine for Diabetic Retinopathy Detection

**DOI:** 10.1155/2018/7015272

**Published:** 2018-10-11

**Authors:** Christina I. Serrano, Vishal Shah, Michael D. Abràmoff

**Affiliations:** ^1^Department of Computer Information Systems, Colorado State University, Fort Collins, CO, USA; ^2^Department of Business Information Systems, Central Michigan University, Mt. Pleasant, MI, USA; ^3^Department of Ophthalmology and Visual Sciences, University of Iowa, Iowa City, IA, USA

## Abstract

**Objective:**

The purpose of the study is to extend research on patient satisfaction with telemedicine services by employing the theoretical framework of Expectation Disconfirmation Theory (EDT) for diabetic retinopathy screenings focusing on rural patients.

**Method:**

Adult subjects (n=220) with diabetes were recruited from a single family practice office in rural Iowa. Subjects completed a “pre” survey concerning their forward-looking perceptions of telemedicine prior to using telemedicine for detection of diabetic retinopathy and a “post” survey after they received recommendations from the distant ophthalmologists.

**Results:**

All hypotheses of the EDT model were supported. Patient satisfaction is influenced by both patients' expectations (*P*<.001) and disconfirmation of expectations (*P*<.001), and patient satisfaction has a positive impact on patient preference for telemedicine services (*P*<.001). Overall, patients who received telemedicine services were highly satisfied with telemedicine and developed a favorable disposition towards telemedicine services.

**Conclusions:**

The EDT model is a viable framework to study patient satisfaction of telemedicine services. While previous feasibility studies have shown that telemedicine for diabetic retinopathy screenings yields diagnostic efficacy, this study applies a theoretical framework to demonstrate the viability of telemedicine for diabetic retinopathy screenings in rural areas.

## 1. Introduction

In the United States (US), diabetic retinopathy is the main cause of blindness among individuals who are 20 to 74 years old [1, 2], and its estimated prevalence is approximately 29 percent among adults with diabetes [3]. Early detection and effective treatment of diabetic retinopathy can prevent blindness and visual loss in almost all cases, but most patients are symptomless until the retinopathy has reached advanced stages [4, 5]. Therefore, the American Diabetes Association, the American Academy of Ophthalmology, and other scientific organizations recommend regular or annual screenings for diabetic retinopathy; however, a significant proportion of people with diabetes do not undergo such examinations [4, 5]. Prior studies show that patient satisfaction is critical in improving patients' decisions to seek appropriate healthcare [6, 7] and this extends to diabetic retinopathy exams [8]. In fact, recent work suggests that telemedicine shows promise for diabetic retinopathy screening, with the potential to increase patient adherence with prescribed disease management practices (e.g., blood sugar control, office visits, and medication compliance), all of which can help prevent end-organ damage [7]. Thus, it becomes important to investigate factors driving patients' satisfaction and preferences, as they are important consumers of healthcare services. To this end, we develop a research model of patients' preference for diabetic retinopathy exams using an asynchronous telemedicine service.

Telemedicine involves the use of telecommunication networks to enable information exchange between healthcare providers and patients who are geographically separated. The two primary modes of telemedicine delivery are store-and-forward, which is asynchronous, and real-time, which is synchronous. With increasing advances in telecommunications networks and technology, telemedicine has shown promise in providing healthcare services to patients remotely [9, 10], particularly to patients in rural areas, who have limited access to many healthcare services locally [11]. In rural areas, access to eye care specialists is limited; thus, applications of telemedicine that enable rural patients to access diabetic retinopathy screenings in their local primary care setting may facilitate their compliance with annual eye exams [12, 13]. Telemedicine has been successfully used to detect asymptomatic retinal abnormalities in Spain [14]. A recent meta-analysis showed that the use of telemedicine for type 2 diabetes mellitus (DM) patients showed promising results for self-management of the disease [15]. Thus, it becomes important to understand the disposition of diabetic patients towards telemedicine. Recent research [7] suggests designing telemedicine systems for diabetic retinopathy screening as well advocates studying patients' perceptions.

For this application of telemedicine to be successful, patients with diabetes will need to have positive perceptions concerning the actual experience of using telemedicine services for these screenings. Research has shown that the application of telemedicine for diabetic retinopathy screenings is both feasible and efficacious [12, 13, 16–20], not only in the US, but also in other countries, such as India [21], South Africa [22], and China [23]. Although some studies have shown that patients reported high levels of satisfaction with using telemedicine for diabetic retinopathy screenings [20, 21, 24–27], none of these studies investigate changes in patient perceptions over time, before and after usage of telemedicine. Furthermore, no studies to date concerning telemedicine patient satisfaction have employed a theoretical framework to investigate patient satisfaction for diabetic retinopathy screenings. Thus, the purpose of this study is to examine the antecedents and consequences of patient satisfaction with telemedicine for diabetic retinopathy screenings by employing the theoretical framework of Expectation Disconfirmation Theory (EDT) with a longitudinal field study design (assessing “pre” and “post” service encounter beliefs) to gain a deeper understanding of this phenomenon.

We concentrate on patient satisfaction for two primary reasons. First, patient satisfaction is considered an important component of the quality of care a patient receives [28, 29] and is linked to improved treatment adherence and clinical outcomes [26, 30]. Second, existing studies of patient satisfaction have been criticized for their lack of conceptual unity and theoretical development [6], particularly within the context of telemedicine [31, 32]. In fact, many telemedicine patient satisfaction studies conflate the concept of satisfaction with related but conceptually distinct factors, such as telemedicine acceptance and perceived quality of care [31, 32]. A recent systematic review of the impact of medical informatics (including telemedicine) on patient satisfaction found that, though most studies report improvements in patient satisfaction, results are largely inconsistent across studies, and few studies focused on patient satisfaction [33]. Another recent systematic review focused on patient satisfaction with telemedicine for cardiology and found that none of the existing studies provided a clear definition or used the same measures for patient satisfaction [34]. There is a need to conduct scholarly research that grounds the empirical assessment of patient satisfaction within valid theoretical frameworks as scholars and practitioners alike generally agree that patient satisfaction is an important component of healthcare quality [35]. Otherwise, it becomes difficult to synthesize findings across patient satisfaction studies and to build on existing research in this domain.

Some healthcare studies have leveraged the well-established consumer satisfaction literature in the marketing discipline to conceptualize patient satisfaction [36], one of the most prominent theories being EDT [37, 38]. Studies in marketing have applied EDT to predict consumer preferences [39], repurchase intentions [40, 41], and customer loyalty [40, 42]. Furthermore, EDT has been used in the information systems literature to predict technology users' intentions to continue using information technologies [43, 44]. In healthcare, the EDT model also has been adapted in patient satisfaction studies across a variety of contexts, such as medication-related services [45], surgical treatment outcomes [46], waiting times for surgery [47], emergency department services [48], and eHealth Website users [49]. Although several telemedicine studies concerning patient satisfaction exist [50, 51], they are largely atheoretical in nature [31, 32, 35].

We address this gap by building on existing research related to patient satisfaction with telemedicine [50, 51], specifically employing the theoretical framework of EDT to conceptualize and empirically investigate patient satisfaction with telemedicine for diabetic retinopathy screenings. Our goal is to develop a nomological model for patients' preference behavior. Because our research stems from the behavioral psychology paradigm, our focus is on perceptions; i.e., various constructs/variables in the model represent patients' perceptions of reality. It has been well established that perceptions offer valuable insights in studying human behavior, as is the case for this study [52, 53].

Although EDT has been leveraged in patient satisfaction studies, it is important to note that some scholars criticize research that employs theories of consumer behavior in the healthcare context. The criticism stems from the notion that market conditions in the US healthcare industry differ from markets that are traditionally studied in economically driven models [54]. At the same time, some scholars note that healthcare in various countries is increasingly becoming commoditized [55, 56] and patients can be regarded as consumers who make choices about their healthcare [57]. Given these rising shifts in healthcare, it becomes even more important to study patient perceptions and experiences, particularly because patient experiences and satisfaction are core components of the Triple Aim framework aimed at guiding healthcare improvements [58]. Furthermore, from a service point of view, it is important to understand what makes patients satisfied with a specific service. This need is more pronounced when trying to understand the feasibility of implementing telemedicine in rural areas. If potential patients do not “buy in” to the proposed method of medical delivery (in this case, telemedicine), then the exercise of rolling out such a service may be futile. Thus, there is merit to examining patient perceptions. Theories such as EDT provide an overarching framework to examine patient satisfaction and preferences systematically. This pilot study is a step in the same direction.

## 2. Theoretical Framework and Research Hypotheses

**Figure 1:**
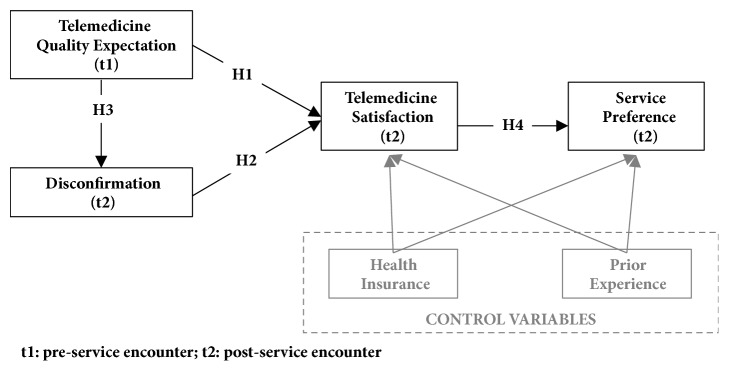
Research model.

According to EDT, satisfaction is defined as a consumer's judgment that a product or service provided a pleasurable level of consumption-related fulfillment [38]. Satisfaction is determined by consumers' preconsumption expectations about a product or service and expectation disconfirmation; each explained next.  Figure 1 shows the research model (because the focus of the study is on telemedicine services, at times, we refer to telemedicine services as services).

Expectations are forward-looking beliefs concerning the service encounter and reflect patient perceptions of the telemedicine encounter prior to receiving this service. A priori expectations represent a baseline standard of comparison or comparative referent, to form postconsumption judgments. In other words, the observed performance of a service postconsumption only has meaning if it is compared to some baseline standard, the a priori expectations [38]. Expectations can be influenced by prior perceived experience and communication messages from salespeople, physicians, nurses, other personnel, and social referents [37].

Expectation disconfirmation is defined as the difference between the preconsumption expectations and postconsumption observed performance [37]. Positive disconfirmation results when the observed performance of the service exceeds preconsumption expectations (i.e., “better than expected”); negative disconfirmation results when the observed performance of the service falls below preconsumption expectations (i.e., “worse than expected”). Both expectations and disconfirmation jointly positively predict consumer satisfaction, and these relationships are explained through two primary mechanisms: the assimilation effect and the contrast effect.

The positive relationship between expectations and satisfaction is explained through an assimilation effect. When patients observe that their postconsumption service experience performs closely to their preconsumption expectations, they tend to “assimilate” their postconsumption perceptions towards their baseline expectations (i.e., the service “meets expectations”) and rely heavily on these initial expectations to form satisfaction judgments [38]. Therefore, we hypothesize the following.

*H1: Patient's Preconsumption Expectations Will Positively Influence Satisfaction with Telemedicine Services.* The positive relationship between disconfirmation and satisfaction is explained through a contrast effect. When individuals observe postconsumption experiences that deviate notably from their preconsumption expectations, they tend to exaggerate these differences such that service performance observed to be better than expected is considered exceptionally good, whereas service performance that is worse than anticipated is regarded as exceptionally bad [38]. When service performance is perceived as exceptionally good (i.e., positively disconfirmed), individuals are more satisfied as compared to when service performance is perceived as exceptionally bad (i.e., negatively disconfirmed), the latter leading to dissatisfaction.

*H2: Patient's Expectation Disconfirmation Will Positively Influence Satisfaction with Telemedicine Services.* The relationship between expectations and disconfirmation has yielded mixed findings in empirical studies that have tested EDT [38, 43]. Typically, this relationship has been modeled as negative [42, 59]. Most commonly, the negative relationship between expectations and disconfirmation is explained through a ceiling/floor effect [38]. The “ceiling effect” refers to situations in which the actual performance of the service will be incapable of reaching extremely high (i.e., ceiling) expectation levels, leading to negative disconfirmation. The same applies to low levels of expectations, the lowest level possible representing “the floor.” There is a lower likelihood that the actual performance will reach these extreme low (i.e., floor) expectation levels, resulting in positive disconfirmation. In our telemedicine context, this means that the higher the patients' expectations of the telemedicine service, the greater the likelihood of negative disconfirmation (i.e., perceptions of the telemedicine service being “worse than expected”), due to the ceiling effect. Alternatively, the lower the patients' expectations of the telemedicine service, the greater the likelihood of positive disconfirmation (i.e., perceptions of the telemedicine service being “better than expected”), due to the floor effect.

*H3: Patient's Preconsumption Expectations Will Negatively Influence Expectation Disconfirmation.* The EDT framework has been extended to include cognitive outcomes of satisfaction, including consumer preference, which is positively influenced by satisfaction [60]. In our study, this means that the more satisfied patients are with the telemedicine service, the higher their preference for consuming telemedicine.

*H4: Patient Satisfaction Will Positively Influence Preference for Receiving Telemedicine Services.* Two control variables, health insurance and prior perceived experience with ophthalmologist exams, which may influence patient satisfaction and patient preference, are included in the research model to control for rival explanations. Though these control variables are outside the scope of the EDT framework, they have been proposed as predisposing and enabling factors that influence patients' utilization of health services and ultimately patient satisfaction [61].

## 3. Method

### 3.1. Sample and Procedure

Subjects were recruited from a single family practice office in rural Iowa. In this rural location, patients did not have an alternative to telemedicine, other than refusal of the service, because there were no available ophthalmologists within a reasonable driving distance of the rural community. The study protocol and consent procedures were approved by the Institutional Review Board of the University of Iowa and the family practice office. Written informed consent was obtained from all participants, and the study was performed according to the tenets of the Declaration of Helsinki. One week prior to the start of the study, the family practice office was equipped with a digital camera and other types of equipment required for Internet-based remote diabetic retinopathy screening using telemedicine. This equipment and the system, operational in the Netherlands as well as in the Midwest US, have been described previously [17].

To participate in the study, the patients had to be at least 18 years of age and must have had a diagnosis of diabetes 1 or 2 by the American Diabetes Association criteria. Patients were excluded if they had a documented dilated retinal exam within the last 12 months or had ever utilized telemedicine. By precluding patients with any previous experience with telemedicine, we attempted to ensure that we indeed measured expectations of first-time users.

Consecutive patients with diabetes that visited the primary care clinic for diabetes follow-up were identified and consented by one of the practice nurses. After explaining the nature of the study and the process of digital photography and remote detection, the “pre” survey was administered. Participants were then photographed by the same nurse with the Topcon NW-200 “nonmydriatic” digital fundus camera (Topcon, Paramus, NJ), with pharmacological dilation if deemed necessary by the nurse. Pharmacological dilation was done for certain patients to ensure sufficient image quality. The nurse entered the digital retinal images and clinical data including Haemoglobin A1c, (HbA1C), duration of diabetes, and any risk factors on a secure Internet website. Within two working days, the images were reviewed electronically by retina fellowship-trained ophthalmologists at the University of Iowa. The International Clinical Diabetic Retinopathy Disease Severity Scale was used to document the severity of diabetic retinopathy and the recommended course of action: annual follow-up with telemedicine or referral to an ophthalmologist for follow-up or treatment [62]. The subject then returned for a follow-up appointment, during which the nurse accessed the subject-specific report via the Internet website and explained the report to the subject. After this interaction, the subject completed the “post” survey. Both “pre” and “post” surveys were paper-based.

### 3.2. Measures and Descriptive Statistics

Variables used for the analysis were captured before consumption via the presurvey and after consumption via the post survey.  Table 1 describes the variables measured in the study and specifies which are included in the research model and post hoc analysis. Given the behavioral perspective of this study, all constructs in this study are perceptual measures. Patients' expectations (preusage) and their preference for telemedicine service prior to (preusage) and after its use (postusage) were measured. The difference between patients' expectations of the quality of the retinal exam (preusage) and their postusage perceptions of the actual quality of the exam is referred to as “disconfirmation.” Expectations, disconfirmation, and satisfaction constitute the core ideas of EDT. Because the study is anchored in behavioral psychology, we used Likert scales to measure the various constructs in the study. Expectations about service are a construct that is relevant prior to using the service and hence only measured before use (i.e., pre). Patients' preference for a particular service can change based on their actual experiences with the service; thus, patient preference is measured before and after use of a telemedicine retinal exam (i.e., pre and post). Satisfaction with the service is measured after use (i.e., post). Because we precluded participants with telemedicine experience from participating in the study, we did not measure satisfaction with telemedicine prior to its consumption. According to EDT, expectations and disconfirmation are the primary drivers of satisfaction.

Most scales were measured using a 5-point Likert scale. The two control variables were measured using a binary scale (yes/no), and the disconfirmation variable was computed using a difference score, which is one of two main approaches to measuring disconfirmation [63]. The measure of satisfaction was an overall (global) measure that captured patients' level of satisfaction with the telemedicine service. Satisfaction can be measured at the overall service encounter level or at specific attribute levels to capture multidimensional aspects of satisfaction (e.g., wait time and staff friendliness) [27, 29, 30]. As EDT has not been employed in a telemedicine context, we opted to use an omnibus, unidimensional measure of satisfaction to improve parsimony and generalizability. Furthermore, a unidimensional measure of satisfaction is the most common approach in the consumer satisfaction literature, in which EDT is rooted [38].

A total of 247 respondents submitted both “pre” and “post” surveys. Of the 247 respondents, 101 (40.9%) were male, and 146 (59.1%) were female. Their ages ranged from 24 to 94. The vast majority of patients, 220 of the 247 patients (89.1%), were over 50 years of age, with 27 of the 247 patients (10.9%) being between 24 and 49 years of age. Because demographic data were collected separately and unpaired from the surveys, we were not able to use these variables as covariates in the data analysis. Descriptive statistics reported for age and sex reflect the total pool of respondents (n=247). Due to incomplete responses, 27 observations were dropped, yielding a final sample size of 220 patient surveys reflecting both “pre” and “post” perceptions. Descriptive statistics of the study variables are shown in Table 2. Because patients did not have an alternative to telemedicine, it precluded us from comparing results with a group that may have never wanted to use telemedicine, but our goal was not to compare two groups. Given our goal to develop a model to understand how patients' perceptions about telemedicine changed once they consumed it firsthand, the current convenience sample served our purpose and can be considered a pilot study. Future studies can extend this work to include a group that receives the same retinopathy screening and diagnosis without the use of telemedicine (i.e., a face-to-face exam).

Because our sample included only those subjects who utilized telemedicine for remote diagnosis (as face-to-face services were not a feasible option), we investigated the extent to which our sample may be positively biased by self-selection. Using unpaired t-tests, we compared (1) answers on patient perceptions reported by subjects who participated in the first part of the telemedicine service only (“pre” survey) and never returned for the telemedicine diagnosis to (2) answers on expectations reported by subjects who experienced the full telemedicine service. This type of extrapolation method is commonly used in survey research to designate subjects who participate “less readily” (e.g., those who partially participate or require prodding to participate) as proxies for those who would choose not to participate at all [64]. Unpaired t-test results shown in Table 3 reveal that there were no significant differences between survey responses provided by subjects who received the telemedicine diagnosis and those who did not complete the study, providing some assurance that self-selection bias was not a major concern with our sample.

## 4. Results

**Figure 2:**
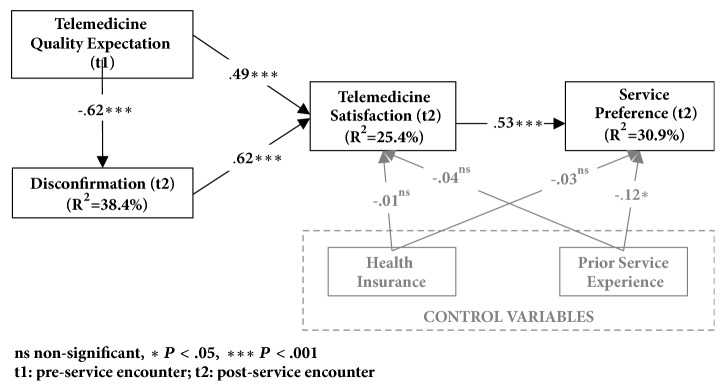
PLS analysis results.

To test our hypotheses, we used structural equation modeling (SEM) utilizing the partial least squares (PLS) method with SmartPLS version 2.0.M3 [65]. We used SEM because we want to explain the interaction of various latent variables (captured by constructs using Likert scales and difference scores) in the nomological model. Since we are proposing a model of patient satisfaction based on EDT, PLS is ideal for our theory building purposes [66–68]. PLS also has been employed in previous telemedicine research that proposes nomological models of psychological constructs [69]. Results of the PLS analysis are shown in Figure 2.

All hypotheses from our research model were supported, as illustrated in Figure 2. Both preusage expectation and disconfirmation positively predicted patient satisfaction with telemedicine (both at* P* <.001), and preusage expectation negatively influenced positive disconfirmation (*P*<.001). These factors explained 25.4% of the variance in patient satisfaction. Furthermore, patient satisfaction significantly and positively impacted patient preference for telemedicine (*P*<.001), explaining 30.9% of the variance in patient preference. The control variable of patients' possession of health insurance was a nonsignificant predictor of patient satisfaction (*P*=.87) and patients' preference for telemedicine services (*P*=.62). We surmise that since all patients had insurance, there was little variability in the data and, hence, its relationship with patient satisfaction and preference was not statistically significant. However, there was a significant negative relationship between the patients' prior experience (within the past five years) with ophthalmologist exams and patients' preference for telemedicine services (*P*=.04). In our case, the results suggest that if patients had prior experience with face-to-face ophthalmologist exams, they were less likely to prefer telemedicine services to screen for diabetic retinopathy. However, prior experience with face-to-face ophthalmologist exams had no significant effect on patient satisfaction with telemedicine services (*P*=.55).

As a post hoc analysis, we compared “pre” and “post” telemedicine usage beliefs using paired t-tests using SPSS 20.0 [70]. The results (shown in Table 4) reveal significant increases both in patients' perceptions of the quality of telemedicine services and in patients' preferences for telemedicine services (versus face-to-face services) after the patients gained firsthand experience with telemedicine. In other words, patients' perceptions of using telemedicine for diabetic retinopathy screenings significantly improved between the “pre” and “post” periods.

Therefore, the results show that introduction of diabetic retinopathy screening using telemedicine was preferred by patients attending the rural family practice office. The post hoc analysis reveals that telemedicine also improved the perceived quality of the dilated eye exam for these patients. Both perceptions of satisfaction with and preference for telemedicine were stronger after the patients had gained firsthand experience using the service.

## 5. Discussion

The purpose of this study is to examine patient satisfaction with using telemedicine for diabetic retinopathy screenings leveraging reputed theories of consumer satisfaction from the marketing discipline. Reviews of the telemedicine literature have cited many challenges with patient satisfaction studies, highlighting the limited application of cohesive theories underlying satisfaction [35, 50, 51]. To address this gap, we applied the EDT model using a longitudinal study design to assess patient satisfaction with telemedicine.

Findings from our study indicate that the EDT framework can be leveraged to evaluate patient satisfaction with telemedicine. All hypotheses from the research model were supported. Further analyses of the data also reveal that patient perceptions of telemedicine significantly improved between the preusage and postusage stages. Overall, patients were satisfied with the telemedicine screenings and preferred the telemedicine service over a face-to-face visit. However, patients with prior experience with face-to-face ophthalmology exams were less likely to prefer telemedicine services, even after using telemedicine.

### 5.1. Contributions to the Literature

By applying the EDT framework, we contribute to the telemedicine literature by offering a new theoretical lens to study patient satisfaction of telemedicine services used to diagnose diabetic retinopathy. Furthermore, expectations, by definition, are forward-looking beliefs concerning the service encounter. Thus, an accurate account of patient expectations requires measurement of patient expectation perceptions* prior to *the telemedicine service encounter. To assess the other constructs of EDT and test the model's relationships, patient perceptions should also be measured* after* the telemedicine service encounter. Hence, this requires a longitudinal design that incorporates a “pre” and “post” assessment of perceptions [37, 43]. A few telemedicine patient satisfaction studies have measured perceptions at two points in time [71], and this study addresses this limitation as well. Furthermore, our study addresses recent calls to study patient satisfaction as a focal outcome within the context of medical informatics and specifically within the subcategory of telemedicine [32].

### 5.2. Contributions to Practice

The study informs practice in many ways. First, the study focuses on both the antecedents and consequences of patient satisfaction, and these factors may influence the manner in which healthcare providers and administrators deliver healthcare services to improve patient satisfaction. The success of these efforts is important because research has shown that patients who are satisfied with their healthcare services are more likely to adhere to medication and treatment advice [26, 72, 73] and return to their source of care [74].

The findings from our study suggest that both the patients' expectations of telemedicine services and the disconfirmation of these expectations (i.e., better or worse than expected) influence their perceived satisfaction with telemedicine. Thus, while it may seem counter-intuitive, one practical recommendation is that healthcare providers and administrators who wish to implement a successful telemedicine program should take into account patient expectations prior to the patients' actual service encounter and exercise caution so as not to “overhype” the service in order to avoid extreme expectations that would be difficult to positively disconfirm. This recommendation is in line with existing research in information systems that have shown the importance of accounting for preusage expectations, suggesting that those involved in system implementations should ensure they do not deliberately or inadvertently set unrealistically high expectations when trying to “sell” the benefits of the system to management and users [75].

Recent health informatics studies support this notion as well. A study investigating medical residents' use of iPads in hospital settings found that many residents, prior to the iPad implementation, reported extremely high expectations of the benefits they would reap from using the iPads. However, four months after the deployment, a significant number of residents reported that benefits of the iPad use fell short of their initial expectations, and more residents indicated a preference for pen and paper usage to complete tasks than they did prior to the iPad deployment [76]. Another study evaluated health professionals' expectations versus actual experiences of telemonitoring and found that, although the health professionals expressed high expectations of telemonitoring benefits, they reported actual experiences that were significantly lower than their initial expectations, possibly leading to disappointment [77]. In both of these studies, a characteristic of the respondents in the preusage stage was that they had exceptionally high expectations of the health information technology. Because these extremely high expectations were difficult to positively disconfirm, the actual experiences of the health professionals in the postusage stage substantially fell short of their initial expectations, leading to negative perceptions. Had their baseline expectations been tempered, there would have been a greater likelihood of satisfaction.

Furthermore, while telemedicine has its critics, the fact that 88 out of 220 subjects (40%) with diabetes reported not seeing any eye care provider in the previous five years underscores the need for increased accessibility of the dilated eye exam for these populations, including, but not necessarily limited to, telemedicine. This study builds on existing telemedicine studies and supports the notion that telemedicine solutions for diabetic retinopathy screenings are feasible in the primary care setting [12, 17, 18]. We extend prior telemedicine research by employing the EDT framework. This framework gives researchers a way to understand the factors that drive patient satisfaction. Patient satisfaction with telemedicine has been shown to result in improved adherence to preventive screenings [26] and better clinical outcomes [30]. Hence, positive patient perceptions may enhance compliance with dilated eye exam guidelines, which has been shown to be essential for timely intervention to prevent blindness and vision loss in diabetic patients [13].

### 5.3. Limitations and Future Research

As with all research, our study is not without limitations. One potential limitation is that we measured variables using single items, an approach that is sometimes criticized [78, 79]. However, recent literature has revealed that single item measures have been shown statistically to be equally as reliable and valid as multiple-item measures of the same constructs [80, 81]. In the job satisfaction literature, for example, there has been growing support for the use of single item measures of satisfaction [82–85], and single items are commonly used to measure patient satisfaction with telemedicine [86, 87]. Single item measures are primarily supported when measuring* concrete *constructs, that is, concepts that respondents would clearly understand and have a similar agreement about the meaning of the concepts [81, 88, 89]. Examples of concrete concepts that are most appropriately measured with single items are likability, quality, satisfaction, and price perception [90], whereas examples of abstract concepts (requiring multiple-item measures) include creativity, power, and culture, as these latter concepts are highly complex in meaning [89]. The concepts measured in our study are all examples of concrete concepts because they are simple, well-formed ideas that are easy for respondents to understand, making them appropriate constructs for single item measures. Furthermore, unidimensional constructs, as modeled in our study, are most suitable for single item measurement [89].

The main advantages to using single item measures are that these measures are less time-intensive and taxing on the respondents and considered more flexible than multiple-item scales [81, 82]. These factors reduce respondents' refusal to participate in studies, which is of particular concern in healthcare contexts, where patients in busy clinical practices are already pressed for time [90]. In our study, the respondents were mostly elderly, which further warranted the use of a short questionnaire to reduce the participation burden. Thus, there are many valid reasons to use short scales with single items [84]. However, that being said, future research can incorporate multiple-item measures of EDT constructs within telemedicine studies, whenever it is feasible. Use of single item measures did not allow patients to differentiate between the precise components of the telemedicine service. However, that is not the goal of the study; the study measures overall patient satisfaction as a first step and the factors that lead to it. Recent research suggests that patient satisfaction can be measured as a multidimensional construct [6]. Future research can look at patient satisfaction with respect to various dimensions of the telemedicine service.

Another possible limitation of the study is that we employ the use of a difference score for the measure of disconfirmation. Difference scores have been a topic of considerable debate in the previous literature [91, 92]. However, difference scores can, in fact, represent an individual change in an unbiased manner and are well suited to measure change [92, 93], as they reduce true score variance and increase the power of the significance tests [92]. Further, difference scores may be used validly in multiple regressions on which the PLS analysis used in this study is based, and there is no reason to avoid their use when they suit the context [94]. However, as there are alternative measures of disconfirmation, future research should also investigate other disconfirmation measures within the scope of telemedicine patient satisfaction.

Furthermore, because this study uses a single cross-section of patients attending a rural family practice office in the Midwest of the US, future studies should address larger samples of rural patients and other populations, including urban underserved patients because it is possible that perceptions may differ across these populations. Additionally, though rural or underserved populations are typically the targeted groups to receive telemedicine services, as telemedicine applications diffuse and become more widespread, it will be important to explore patient perceptions across a wider cross-section of patient populations. Nevertheless, rural populations continue to need innovative solutions to improve their access to healthcare, so future research should explore the extent to which rural patients would take advantage of telemedicine services.

Another limitation is that the study does not use a control group of patients who underwent diagnosis and treatment in a face-to-face context. The goal of this pilot study was to develop a model to understand factors that lead to telemedicine preference in and of itself based on EDT. Future research can compare two groups of patients, one using telemedicine while the other visits the ophthalmologist in person. In this study, given the sample limitations, we focus on developing a theoretical model explaining patient satisfaction and preferences once they consume/use telemedicine.

Additionally, our sample only includes patients who utilize store-and-forward (asynchronous) telemedicine for diabetic retinopathy screenings and remote diagnosis. Thus, it is not clear whether our findings will generalize to use of telemedicine in other medical specialties or for other types of telemedicine applications (e.g., interactive video telemedicine or telemonitoring). While preliminary research suggests that patients report high levels of satisfaction using both asynchronous and synchronous telemedicine for diabetes care and that the asynchronous mode is widely used for management of chronic disease such as diabetes [27, 95], future research concerning patient satisfaction with telemedicine should test the EDT framework in these different telemedicine contexts.

It is also important to note that the questionnaire asked patients about their visit specifically to an ophthalmologist in the past five years. However, because both optometrists and ophthalmologists provide routine eye care, it may be possible that patients visited an optometrist instead of an ophthalmologist and reported their past experience based on visits to either specialist. Future studies should consider recording experience with both ophthalmologists as well as optometrists.

Further, it should be noted that our study focuses on perceptions, as it is important to understand how patients perceive a new development, such as telemedicine. However, the link between a user's perception and actual behavior and outcomes is contested and largely termed the “intention-behavior” gap in various domains, such as technology use, consumer purchasing behavior, and physical activity [96–100]. Patients may form favorable perceptions of telemedicine but may not actually interact with or use the target technology. However, this concern is mitigated to a certain extent in our study, as the patients were not actively trying to manage or use the equipment but were recipients of the diagnosis. Thus, studying perceptions would be a relevant measure of whether participants will voluntarily be recipients of such asynchronous telemedicine services in the future. Additional longitudinal studies that measure patients' actual future behavior and objective clinical outcomes, to include correlations between these outcomes and patient perceptions, are needed.

Moreover, there are several caveats that researchers should be aware of when focusing on patient-reported expectations (PREM) and/or outcomes (PROM). Although there is research suggesting that patient-reported expectations and perceived outcomes are correlated with objective clinical outcomes [28, 29], this link is still contested in the literature, and some studies have presented counter evidence [101]. It may be possible that patients' perceived satisfaction would bias them towards the service regardless of the actual clinical outcomes. Furthermore, a recent metareview of telemedicine use for chronic disease management found that the majority of studies on telemedicine interventions have reported positive effects with very few studies reporting negative effects, which suggests a publication bias [102]. Future research on telemedicine interventions should consider and account for potential negative consequences of telemedicine.

Another avenue of fruitful research would be to explore additional dimensions of patient perceptions that could influence disconfirmation and patient satisfaction. While our study assessed patients' expectations and perceived preference of the quality of care provided, other dimensions, such as patient perceptions of the teleproviders' competence or the quality of information exchange via telemedicine, may also be relevant to study. Recent work on telemedicine indicates that it can detect not only diabetic retinopathy but also other visually significant eye diseases [103]. Thus, future work can consider studying telemedicine for screening a wider group of ocular diseases. Lastly, because our study is the first to test the EDT framework in a telemedicine context, we only included the key variables relevant to EDT. Future work may build on this model by investigating additional factors (such as social influence and trust in the teleprovider) that may impact patient satisfaction and telemedicine preference.

## 6. Conclusion

In summary, the results show that patients were very satisfied with their use of telemedicine for diabetic retinal screenings and preferred telemedicine services. Our study found that patients' satisfaction with the telemedicine service leads to a preference to use the service. The study was not aimed at making a comparison between telemedicine and face-to-face services using a randomized controlled trial—i.e., we did not have a control group (face-to-face condition). Our goal was to develop a theoretical model of telemedicine satisfaction and preferences using EDT. Previous studies of telemedicine satisfaction and preferences have been largely descriptive and atheoretical [77, 87, 104–106]. Some studies have described telemedicine implementations in detail [107], while others report a positive preference for telemedicine [108–110]. However, the theoretical mechanism that may guide patients' perceived satisfaction and preference of telemedicine has not been widely studied. This specific pilot study fills this gap in the telemedicine literature by building on the theoretical foundations of EDT.

This pilot study focused on a preliminary examination of patient perceptions. We found that the implementation approach used in this telemedicine study was perceived favorably based on patients' satisfaction with it. The results of the study suggest that telemedicine initiatives aimed at achieving this end, according to the EDT framework, should consider implementation approaches that will provide the patient with a “better than expected” telemedicine experience, which will lead to improved patient satisfaction and greater patient preference for the use of telemedicine. Offering telemedicine services for diabetic retinal screenings in primary care settings has the potential to increase compliance with routine dilated eye exams for diabetic patients. Future research can investigate this link in greater detail.

## Figures and Tables

**Table 1:** Summary of variables included in the study.

**Time**	**Variable**	**Description and role in the research model**	**Scale**	**Included in Research Model**	**Included in Post Hoc Analysis**
Pre (t1)	Service Quality Expectation	Forward-looking belief concerning the quality of the health service that provides a baseline value of expectations.	Likert scale of 1-5, ophthalmologist's office to telemedicine	Yes	Yes

Pre (t1)	Service Preference	Pre-consumption inclination for face-to-face or telemedicine service that provides a baseline value of service preference. This variable is used in the post-hoc analysis.	Likert scale of 1-5, ophthalmologist's office to telemedicine	No	Yes

Control (t1)	Health Insurance	Possession of health insurance, used as a control variable in the research model.	Binary scale, Yes/No (0=no, 1=yes)	Yes	No

Control (t1)	Prior Service Experience	Prior experience (in the last five years) with ophthalmologist exams, used as a control variable in the research model	Binary scale, Yes/No (0=no, 1=yes)	Yes	No

Post (t2)	Service Quality Performance	Post-consumption judgment that the actual service delivered quality outcomes. This variable is used in the post-hoc analysis.	Likert scale of 1-5, ophthalmologist's office to telemedicine	No	Yes

Post (t2)	Service Preference	Post-consumption preference for face-to-face or telemedicine service.	Likert scale of 1-5, ophthalmologist's office to telemedicine	Yes	Yes

Post (t2)	Disconfirmation	The difference between pre-consumption expectation and post-consumption performance. It may be positive or negative.	Calculated difference score between Service Quality Expectation (t1) and Service Quality Performance (t2)	Yes	No

Post (t2)	Satisfaction	A patient's overall satisfaction with the telemedicine service.	Likert scale of 1-5, Terrible to Very good	Yes	No

a. Four of the variables were measured using a Likert scale of 1-5, ophthalmologist's office to telemedicine, which represents a continuous scale according to the richness of medium (ophthalmologist's office=richer medium; telemedicine=leaner medium).

**Table 2:** Descriptive statistics of variables (n=220).

**Time**	**Variable**	**Mean**	**Std. Dev.**	**Min**	**Max**
Pre (t1)	Service Quality Expectation	3.02	1.19	1	5

Pre (t1)	Service Preference	3.43	1.26	1	5

Control (t1)	Health Insurance	0.95	0.22	0	1

Control (t1)	Prior Service Experience	0.60	0.49	0	1

Post (t2)	Service Quality Performance	4.01	1.16	1	5

Post (t2)	Service Preference	4.22	1.09	1	5

Post (t2)	Disconfirmation	0.99	1.43	-3	4

Post (t2)	Satisfaction	4.50	0.77	2	5

**Table 3:** Post hoc assessment of self-selection bias.

**Variable**	**Study sample**		**Dropped cases**		** T-Test**	
	**Mean**	**Std. Dev.**	**Mean**	**Std. Dev.**	**t-Stat.**	**Sig.**
**Service Quality Expectation (pre) **	3.02	1.19	3.4	1.30	1.29	*P*=0.23

**Service Preference (pre)**	3.43	1.26	3.16	1.28	0.77	*P*=0.44

**Table 4:** Post hoc comparison of pre- and postbeliefs (n=220).

**Variable**	**Pre-Encounter**		**Post-Encounter**		**Paired T-Test**	
	**Mean**	**Std. Dev.**	**Mean**	**Std. Dev.**	**t-Stat.**	**Sig.**
**Service Quality Expectation (pre)/Actual Service Quality Performance (post)**	3.02	1.19	4.01	1.16	-10.28	*P*<.001

**Service Preference**	3.43	1.26	4.22	1.09	-8.80	*P*<.001

## Data Availability

Please find the survey files here https://www.dropbox.com/sh/ad3s9xaq9ldlrwh/AADXfVkuc5gRjiSLYSFQgSc9a?dl=0 or from the corresponding author upon request

## Supplementary Materials

Supplementary Materials The survey questions were referenced in Table 1. The survey had two sections, “Before” and “After”. A patient completed the “Before” survey prior to the diagnosis using the telemedicine and completed “After” survey in at the follow-up meeting when receiving the telediagnosis.
