# Supplementary material for: Use of Expectation Disconfirmation Theory to Test Patient Satisfaction with Asynchronous Telemedicine for Diabetic Retinopathy Detection
Source: Int J Telemed Appl. 2018 Oct 11;2018:7015272. doi: 10.1155/2018/7015272 (PMC6201495; doi:10.1155/2018/7015272)
Supplement: Supplementary Materials — The survey questions were referenced in Table 1. The survey had two sections, “Before” and “After”. A patient completed the “Before” survey prior to the diagnosis using the telemedicine and completed “After” survey in at the follow-up meeting when receiving the telediagnosis. [file 7015272.f1.pdf]

Patient questionnaire

BEFORE telediagnosis

Name[redacted]________ **Date:**_[redacted]

Thank you for your willingness to participate in this research project.
You are about to undergo digital photographing and diagnosis over the internet by ophthalmologists who are retinal specialists at the University of Iowa. They will evaluate your photographs for signs of diabetic retinopathy, a complication of diabetes. This new technique is called 'telediagnosis'. Until telediagnosis was available, you could only be screened in an ophthalmologist's office with a dilated retinal exam.

Please give your best answer to the following questions

Circle where you expect the quality of the retinal exam to be better:

1	2	3	4	5
Ophthalmologist's office			Telediagnosis	

Circle which retinal exam you prefer:

1	2	3	4	5
Ophthalmologist's office			Telediagnosis	

- □ yes ☒ no Do you expect the camera flashlight to bother you
- □ yes ☒ no Do you expect the pupil dilating drops to bother you
- □ yes ☒ no In the past five years have you been examined by an ophthalmologist
- ☒ yes □ no Do you currently carry health insurance
- ☒ yes □ no In some cases the photographs can only be made if your pupils are dilated with a drop. Were you aware of this?

Patient questionnaire

[redacted]R telediagnosis results have been discussed

Name:[redacted]________ **Date:**_[redacted]___

Circle your satisfaction with digital photography of your retina

1	2	3	4	5
Terrible				Very good

Circle where you think the retinal exam is better:

1	2	3	4	5
Ophthalmologist's office				Telediagnosis

Circle which retinal exam you prefer now:

1	2	3	4	5
Ophthalmologist's office				Telediagnosis

- □ yes ☒ no Did the camera flashlight bother you
- □ yes □ no Did the pupil dilating drops bother you

After form is completed, please enter into Excel spreadsheet or send to:

Dr. Michael D. Abramoff, MD, PhD
Department of Ophthalmology and Visual Sciences
PFP 11290C
University of Iowa Hospitals and Clinics
200 Hawkins, Iowa City, IA 52242
Tel (319) 384 5833, fax: (319) 353 7996

[redacted]

[redacted]

Patient questionnaire

[redacted]EFORE telediagnosis

Name: [redacted] **Date:** [redacted]

Thank you for your willingness to participate in this research project.
You are about to undergo digital photographing and diagnosis over the internet by ophthalmologists who are retinal specialists at the University of Iowa. They will evaluate your photographs for signs of diabetic retinopathy, a complication of diabetes. This new technique is called 'telediagnosis'. Until telediagnosis was available, you could only be screened in an ophthalmologist's office with a dilated retinal exam.

Please give your best answer to the following questions

Circle where you expect the quality of the retinal exam to be better:

1	2	(3)	4	5
Ophthalmologist's office			Telediagnosis	

Circle which retinal exam you prefer:

1	2	(3)	4	5
Ophthalmologist's office			Telediagnosis	

☐ yes ☐ no Do you expect the camera flashlight to bother you
☐ yes ☒ no Do you expect the pupil dilating drops to bother you
☒ yes ☐ no In the past five years have you been examined by an ophthalmologist
☒ yes ☐ no Do you currently carry health insurance
☒ yes ☐ no In some cases the photographs can only be made if your pupils are dilated with a drop. Were you aware of this?

Patient questionnaire

[redacted]gnosis results have been discussed

Name: [redacted] **Date:** [redacted]

Circle your satisfaction with digital photography of your retina

1	2	(3)	4	5
Terrible				Very good

Circle where you think the retinal exam is better:

1	2	(3)	4	5
Ophthalmologist's office				Telediagnosis

Circle which retinal exam you prefer now:

1	2	3	(4)	5
Ophthalmologist's office				Telediagnosis

☐ yes ☒ no Did the camera flashlight bother you
☐ yes ☐ no Did the pupil dilating drops bother you

After form is completed, please enter into Excel spreadsheet or send to:

Dr. Michael D. Abramoff, MD, PhD
Department of Ophthalmology and Visual Sciences
PFP 11290C
University of Iowa Hospitals and Clinics
200 Hawkins, Iowa City, IA 52242
Tel (319) 384 5833, fax: (319) 353 7996

[redacted]

[redacted]

Patient questionnaire

[redacted]EFORE teledi[redacted]

Name: [redacted] Date: [redacted]

Thank you for your willingness to participate in this research project.

You are about to undergo digital photographing and diagnosis over the internet by ophthalmologists who are retinal specialists at the University of Iowa. They will evaluate your photographs for signs of diabetic retinopathy, a complication of diabetes. This new technique is called 'telediagnosis'. Until telediagnosis was available, you could only be screened in an ophthalmologist's office with a dilated retinal exam.

Please give your best answer to the following questions

Circle where you expect the quality of the retinal exam to be better:

1	2	3	4	5
Ophthalmologist's office			Telediagnosis	

Circle which retinal exam you prefer:

1	2	3	4	5
Ophthalmologist's office			Telediagnosis	

- ☐ yes ☑ no Do you expect the camera flashlight to bother you
- ☐ yes ☑ no Do you expect the pupil dilating drops to bother you
- ☑ yes ☐ no In the past five years have you been examined by an ophthalmologist
- ☑ yes ☐ no Do you currently carry health insurance
- ☑ yes ☐ no In some cases the photographs can only be made if your pupils are dilated with a drop. Were you aware of this?

Patient questionnaire

[redacted]gnosis results have been discussed

Name: [redacted] Date: [redacted]

Circle your satisfaction with digital photography of your retina

1	2	3	4	5
Terrible				Very good

Circle where you think the retinal exam is better:

1	2	3	4	5
Ophthalmologist's office				Telediagnosis

Circle which retinal exam you prefer now:

1	2	3	4	5
Ophthalmologist's office				Telediagnosis

- ☐ yes ☒ no Did the camera flashlight bother you
- ☐ yes ☐ no Did the pupil dilating drops bother you [redacted]

After form is completed, please enter into Excel spreadsheet or send to:

Dr. Michael D. Abramoff, MD, PhD
Department of Ophthalmology and Visual Sciences
PFP 11290C
University of Iowa Hospitals and Clinics
200 Hawkins, Iowa City, IA 52242
Tel (319) 384 5833, fax: (319) 353 7996

[redacted]

[redacted]

Patient questionnaire

BEFORE telediagnosis

Name: [redacted] **Date:** [redacted]

Thank you for your willingness to participate in this research project.
You are about to undergo digital photographing and diagnosis over the internet by ophthalmologists who are retinal specialists at the University of Iowa. They will evaluate your photographs for signs of diabetic retinopathy, a complication of diabetes. This new technique is called 'telediagnosis'. Until telediagnosis was available, you could only be screened in an ophthalmologist's office with a dilated retinal exam.

Please give your best answer to the following questions

Circle where you expect the quality of the retinal exam to be better:

1 2 3 4 5

Ophthalmologist's office — Telediagnosis

Circle which retinal exam you prefer:

1 2 3 4 5

Ophthalmologist's office — Telediagnosis

☒ yes □ no Do you expect the camera flashlight to bother you
□ yes ☒ no Do you expect the pupil dilating drops to bother you
□ yes ☒ no In the past five years have you been examined by an ophthalmologist
☒ yes □ no Do you currently carry health insurance
☒ yes □ no In some cases the photographs can only be made if your pupils are dilated with a drop. Were you aware of this?

Patient questionnaire

[redacted]osis resul[redacted]ssed

Name: [redacted] **Date:** [redacted]

Circle your satisfaction with digital photography of your retina

1 2 3 4 5

Terrible — Very good

Circle where you think the retinal exam is better:

1 2 3 4 5

Ophthalmologist's office — Telediagnosis

Circle which retinal exam you prefer now:

1 2 3 4 5

Ophthalmologist's office — Telediagnosis

□ yes ☒ no Did the camera flashlight bother you
□ yes □ no Did the pupil dilating drops bother you

After form is completed, please enter into Excel spreadsheet or send to:

Dr. Michael D. Abramoff, MD, PhD
Department of Ophthalmology and Visual Sciences
PFP 11290C
University of Iowa Hospitals and Clinics
200 Hawkins, Iowa City, IA 52242
Tel (319) 384 5833, fax: (319) 353 7996

[redacted]

[redacted]

Patient questionnaire

BEFORE telediagnosis

Name: [redacted] Date: [redacted]

Thank you for your willingness to participate in this research project.

You are about to undergo digital photographing and diagnosis over the internet by ophthalmologists who are retinal specialists at the University of Iowa. They will evaluate your photographs for signs of diabetic retinopathy, a complication of diabetes. This new technique is called 'telediagnosis'. Until telediagnosis was available, you could only be screened in an ophthalmologist's office with a dilated retinal exam.

Please give your best answer to the following questions

Circle where you expect the quality of the retinal exam to be better:

1 Ophthalmologist's office — **(2)** — 3 — 4 Telediagnosis — 5

Circle which retinal exam you prefer:

1 Ophthalmologist's office — 2 — 3 — **(4)** Telediagnosis — 5

☐ yes ☑ no Do you expect the camera flashlight to bother you
☐ yes ☑ no Do you expect the pupil dilating drops to bother you
☐ yes ☑ no In the past five years have you been examined by an ophthalmologist
☑ yes ☐ no Do you currently carry health insurance
☑ yes ☐ no In some cases the photographs can only be made if your pupils are dilated with a drop. Were you aware of this?

Patient questionnaire

[redacted]TER telediagnosis results have been discussed

Name: [redacted] Date: [redacted]

Circle your satisfaction with digital photography of your retina

1 Terrible — 2 — 3 — **(4)** — 5 Very good

Circle where you think the retinal exam is better:

1 Ophthalmologist's office — 2 — 3 — **(4)** — 5 Telediagnosis

Circle which retinal exam you prefer now:

1 Ophthalmologist's office — 2 — 3 — **(4)** — 5 Telediagnosis

☐ yes ☑ no Did the camera flashlight bother you
☐ yes ☑ no Did the pupil dilating drops bother you

After form is completed, please enter into Excel spreadsheet or send to:

Dr. Michael D. Abramoff, MD, PhD
Department of Ophthalmology and Visual Sciences
PFP 11290C
University of Iowa Hospitals and Clinics
200 Hawkins, Iowa City, IA 52242
Tel (319) 384 5833, fax: (319) 353 7996

[redacted]

[redacted]

Patient questionnaire

[redacted]**ORE telediagnosis**

Name:[redacted] **Date:**[redacted]

Thank you for your willingness to participate in this research project.
You are about to undergo digital photographing and diagnosis over the internet by ophthalmologists who are retinal specialists at the University of Iowa. They will evaluate your photographs for signs of diabetic retinopathy, a complication of diabetes. This new technique is called 'telediagnosis'. Until telediagnosis was available, you could only be screened in an ophthalmologist's office with a dilated retinal exam.

Please give your best answer to the following questions

Circle where you expect the quality of the retinal exam to be better:

1	2	3	4	5
Ophthalmologist's office			Telediagnosis	

Circle which retinal exam you prefer:

1	2	3	4	5
Ophthalmologist's office			Telediagnosis	

☐ yes ☑ no Do you expect the camera flashlight to bother you
☐ yes ☑ no Do you expect the pupil dilating drops to bother you
☑ yes ☐ no In the past five years have you been examined by an ophthalmologist
☑ yes ☐ no Do you currently carry health insurance
☐ yes ☑ no In some cases the photographs can only be made if your pupils are dilated with a drop. Were you aware of this?

Patient questionnaire

AFTER telediagnosis results have been discussed

Name:____________________ **Date:**__________

Circle your satisfaction with digital photography of your retina

1	2	3	4	5
Terrible				Very good

Circle where you think the retinal exam is better:

1	2	3	4	5
Ophthalmologist's office				Telediagnosis

Circle which retinal exam you prefer now:

1	2	3	4	5
Ophthalmologist's office				Telediagnosis

☐ yes ☐ no Did the camera flashlight bother you
☐ yes ☐ no Did the pupil dilating drops bother you

After form is completed, please enter into Excel spreadsheet or send to:

Dr. Michael D. Abramoff, MD, PhD
Department of Ophthalmology and Visual Sciences
PFP 11290C
University of Iowa Hospitals and Clinics
200 Hawkins, Iowa City, IA 52242
Tel (319) 384 5833, fax: (319) 353 7996

[redacted]

[redacted]

Patient questionnaire

BEFORE telediagnosis

[redacted]

Name: [redacted] Date: [redacted]

Thank you for your willingness to participate in this research project.

You are about to undergo digital photographing and diagnosis over the internet by ophthalmologists who are retinal specialists at the University of Iowa. They will evaluate your photographs for signs of diabetic retinopathy, a complication of diabetes. This new technique is called 'telediagnosis'. Until telediagnosis was available, you could only be screened in an ophthalmologist's office with a dilated retinal exam.

Please give your best answer to the following questions

Circle where you expect the quality of the retinal exam to be better:

1 2 3 4 5

Ophthalmologist's office — Telediagnosis

Circle which retinal exam you prefer:

1 2 3 4 5

Ophthalmologist's office — Telediagnosis

☐ yes ☐ no Do you expect the camera flashlight to bother you

☐ yes ☐ no Do you expect the pupil dilating drops to bother you

☐ yes ☐ no In the past five years have you been examined by an ophthalmologist

☐ yes ☐ no Do you currently carry health insurance

☐ yes ☐ no In some cases the photographs can only be made if your pupils are dilated with a drop. Were you aware of this?

Patient questionnaire

[redacted]lediagnosis results have been discussed

Name: [redacted] Date: [redacted]

Circle your satisfaction with digital photography of your retina

1 2 3 4 5

Terrible — Very good

Circle where you think the retinal exam is better:

1 2 3 4 5

Ophthalmologist's office — Telediagnosis

Circle which retinal exam you prefer now:

1 2 3 4 5

Ophthalmologist's office — Telediagnosis

☐ yes ☐ no Did the camera flashlight bother you

☐ yes ☐ no Did the pupil dilating drops bother you

After form is completed, please enter into Excel spreadsheet or send to:

Dr. Michael D. Abramoff, MD, PhD
Department of Ophthalmology and Visual Sciences
PFP 11290C
University of Iowa Hospitals and Clinics
200 Hawkins, Iowa City, IA 52242
Tel (319) 384 5833, fax: (319) 353 7996

[redacted]

[redacted]

Patient questionnaire

[redacted]RE telediagnosis

Name:[redacted]_ **Date:**_[redacted]

Thank you for your willingness to participate in this research project.

You are about to undergo digital photographing and diagnosis over the internet by ophthalmologists who are retinal specialists at the University of Iowa. They will evaluate your photographs for signs of diabetic retinopathy, a complication of diabetes. This new technique is called 'telediagnosis'. Until telediagnosis was available, you could only be screened in an ophthalmologist's office with a dilated retinal exam.

Please give your best answer to the following questions

Circle where you expect the quality of the retinal exam to be better:

1 2 3 4 5

Ophthalmologist's office — Telediagnosis

Circle which retinal exam you prefer:

1 2 3 4 5

Ophthalmologist's office — Telediagnosis

- □ yes □ no Do you expect the camera flashlight to bother you
- □ yes □ no Do you expect the pupil dilating drops to bother you
- □ yes □ no In the past five years have you been examined by an ophthalmologist
- □ yes □ no Do you currently carry health insurance
- □ yes □ no In some cases the photographs can only be made if your pupils are dilated with a drop. Were you aware of this?

Patient questionnaire

[redacted]s results have been discussed

Name:[redacted]_ **Date:**_[redacted]

Circle your satisfaction with digital photography of your retina

1 2 3 4 5

Terrible — Very good

Circle where you think the retinal exam is better:

1 2 3 4 5

Ophthalmologist's office — Telediagnosis

Circle which retinal exam you prefer now:

1 2 3 4 5

Ophthalmologist's office — Telediagnosis

- □ yes □ no Did the camera flashlight bother you
- □ yes □ no Did the pupil dilating drops bother you

After form is completed, please enter into Excel spreadsheet or send to:

Dr. Michael D. Abramoff, MD, PhD
Department of Ophthalmology and Visual Sciences
PFP 11290C
University of Iowa Hospitals and Clinics
200 Hawkins, Iowa City, IA 52242
Tel (319) 384 5833, fax: (319) 353 7996

[redacted]

[redacted]

Patient questionnaire

BEFORE tele[redacted]

Name:[redacted] **Date:**[redacted]

Thank you for your willingness to participate in this research project.
You are about to undergo digital photographing and diagnosis over the internet by ophthalmologists who are retinal specialists at the University of Iowa. They will evaluate your photographs for signs of diabetic retinopathy, a complication of diabetes. This new technique is called 'telediagnosis'. Until telediagnosis was available, you could only be screened in an ophthalmologist's office with a dilated retinal exam.

Please give your best answer to the following questions

Circle where you expect the quality of the retinal exam to be better:

1	2	3	4	5
Ophthalmologist's office			Telediagnosis	

Circle which retinal exam you prefer:

1	2	3	4	5
Ophthalmologist's office			Telediagnosis	

□ yes ☒ no Do you expect the camera flashlight to bother you
☒ yes □ no Do you expect the pupil dilating drops to bother you
☒ yes □ no In the past five years have you been examined by an ophthalmologist
☒ yes □ no Do you currently carry health insurance
☒ yes □ no In some cases the photographs can only be made if your pupils are dilated with a drop. Were you aware of this?

Patient questionnaire

AFTER telediagnosis results have been discussed

Name:[redacted] **Date:**[redacted]

Circle your satisfaction with digital photography of your retina

1	2	3	4	5
Terrible				Very good

Circle where you think the retinal exam is better:

1	2	3	4	5
Ophthalmologist's office				Telediagnosis

Circle which retinal exam you prefer now:

1	2	3	4	5
Ophthalmologist's office				Telediagnosis

□ yes ☒ no Did the camera flashlight bother you
□ yes □ no Did the pupil dilating drops bother you

After form is completed, please enter into Excel spreadsheet or send to:

Dr. Michael D. Abramoff, MD, PhD
Department of Ophthalmology and Visual Sciences
PFP 11290C
University of Iowa Hospitals and Clinics
200 Hawkins, Iowa City, IA 52242
Tel (319) 384 5833, fax: (319) 353 7996

[redacted]

[redacted]

Patient questionnaire

[redacted]FORE telediagnosis

Name[redacted]**Date:**[redacted]

Thank you for your willingness to participate in this research project.

You are about to undergo digital photographing and diagnosis over the internet by ophthalmologists who are retinal specialists at the University of Iowa. They will evaluate your photographs for signs of diabetic retinopathy, a complication of diabetes. This new technique is called 'telediagnosis'. Until telediagnosis was available, you could only be screened in an ophthalmologist's office with a dilated retinal exam.

Please give your best answer to the following questions

Circle where you expect the quality of the retinal exam to be better:

1 2 3 4 5

Ophthalmologist's office — Telediagnosis

Circle which retinal exam you prefer:

1 2 3 4 5

Ophthalmologist's office — Telediagnosis

- ☐ yes ☒ no Do you expect the camera flashlight to bother you
- ☐ yes ☒ no Do you expect the pupil dilating drops to bother you
- ☒ yes ☐ no In the past five years have you been examined by an ophthalmologist
- ☒ yes ☐ no Do you currently carry health insurance
- ☒ yes ☐ no In some cases the photographs can only be made if your pupils are dilated with a drop. Were you aware of this?

Patient questionnaire

[redacted]gnosis results have been discussed

Name:[redacted] **Date:**________________

Circle your satisfaction with digital photography of your retina

1 2 3 4 5

Terrible — Very good

Circle where you think the retinal exam is better:

1 2 3 4 5

Ophthalmologist's office — Telediagnosis

Circle which retinal exam you prefer now:

1 2 3 4 5

Ophthalmologist's office — Telediagnosis

- ☐ yes ☒ no Did the camera flashlight bother you
- ☐ yes ☒ no Did the pupil dilating drops bother you

After form is completed, please enter into Excel spreadsheet or send to:

Dr. Michael D. Abramoff, MD, PhD
Department of Ophthalmology and Visual Sciences
PFP 11290C
University of Iowa Hospitals and Clinics
200 Hawkins, Iowa City, IA 52242
Tel (319) 384 5833, fax: (319) 353 7996

[redacted]

[redacted]

Patient questionnaire

[redacted]RE telediagnosis

Name: [redacted] **Date:** [redacted]

Thank you for your willingness to participate in this research project.

You are about to undergo digital photographing and diagnosis over the internet by ophthalmologists who are retinal specialists at the University of Iowa. They will evaluate your photographs for signs of diabetic retinopathy, a complication of diabetes. This new technique is called 'telediagnosis'. Until telediagnosis was available, you could only be screened in an ophthalmologist's office with a dilated retinal exam.

Please give your best answer to the following questions

Circle where you expect the quality of the retinal exam to be better:

1	2	3	4	5
Ophthalmologist's office			Telediagnosis	

Circle which retinal exam you prefer:

1	2	3	4	5
Ophthalmologist's office			Telediagnosis	

□ yes □ no Do you expect the camera flashlight to bother you
□ yes □ no Do you expect the pupil dilating drops to bother you
□ yes □ no In the past five years have you been examined by an ophthalmologist
□ yes □ no Do you currently carry health insurance
□ yes □ no In some cases the photographs can only be made if your pupils are dilated with a drop. Were you aware of this?

Patient questionnaire

AFTER telediagnosis results have been discussed

Name:______________________ **Date:**______________

Circle your satisfaction with digital photography of your retina

1	2	3	4	5
Terrible				Very good

Circle where you think the retinal exam is better:

1	2	3	4	5
Ophthalmologist's office				Telediagnosis

Circle which retinal exam you prefer now:

1	2	3	4	5
Ophthalmologist's office				Telediagnosis

□ yes □ no Did the camera flashlight bother you
□ yes □ no Did the pupil dilating drops bother you

After form is completed, please enter into Excel spreadsheet or send to:

Dr. Michael D. Abramoff, MD, PhD
Department of Ophthalmology and Visual Sciences
PFP 11290C
University of Iowa Hospitals and Clinics
200 Hawkins, Iowa City, IA 52242
Tel (319) 384 5833, fax: (319) 353 7996

[redacted]

[redacted]

[redacted]atient questionnaire

[redacted] BEFORE telediagnosis

Name: [redacted] **Date:** [redacted]

Thank you for your willingness to participate in this research project.

You are about to undergo digital photographing and diagnosis over the internet by ophthalmologists who are retinal specialists at the University of Iowa. They will evaluate your photographs for signs of diabetic retinopathy, a complication of diabetes. This new technique is called 'telediagnosis'. Until telediagnosis was available, you could only be screened in an ophthalmologist's office with a dilated retinal exam.

Please give your best answer to the following questions

Circle where you expect the quality of the retinal exam to be better:

1	2	3	4	5
Ophthalmologist's office			Telediagnosis	

Circle which retinal exam you prefer:

1	2	3	4	5
Ophthalmologist's office			Telediagnosis	

- ☐ yes ☑ no Do you expect the camera flashlight to bother you
- ☑ yes ☐ no Do you expect the pupil dilating drops to bother you
- ☑ yes ☐ no In the past five years have you been examined by an ophthalmologist
- ☑ yes ☐ no Do you currently carry health insurance
- ☑ yes ☐ no In some cases the photographs can only be made if your pupils are dilated with a drop. Were you aware of this?

Patient questionnaire

AFTER telediagnosis results have been discussed

Name:____________________ **Date:**__________

Circle your satisfaction with digital photography of your retina

1	2	3	4	5
Terrible				Very good

Circle where you think the retinal exam is better:

1	2	3	4	5
Ophthalmologist's office				Telediagnosis

Circle which retinal exam you prefer now:

1	2	3	4	5
Ophthalmologist's office				Telediagnosis

- ☐ yes ☑ no Did the camera flashlight bother you
- ☐ yes ☑ no Did the pupil dilating drops bother you [redacted]

After form is completed, please enter into Excel spreadsheet or send to:

Dr. Michael D. Abramoff, MD, PhD
Department of Ophthalmology and Visual Sciences
PFP 11290C
University of Iowa Hospitals and Clinics
200 Hawkins, Iowa City, IA 52242
Tel (319) 384 5833, fax: (319) 353 7996

[redacted]

[redacted]

Patient questionnaire

BEFORE telediagnosis

Name: [redacted] **Date:** [redacted]

Thank you for your willingness to participate in this researc[redacted]

You are about to undergo digital photographing and diagnosis over the internet by ophthalmologists who are retinal specialists at the University of Iowa. They will evaluate your photographs for signs of diabetic retinopathy, a complication of diabetes. This new technique is called 'telediagnosis'. Until telediagnosis was available, you could only be screened in an ophthalmologist's office with a dilated retinal exam.

Please give your best answer to the following questions

Circle where you expect the quality of the retinal exam to be better:

1	2	3	4	5
Ophthalmologist's office			Telediagnosis	

Circle which retinal exam you prefer:

1	2	3	4	5
Ophthalmologist's office			Telediagnosis	

☐ yes ☒ no Do you expect the camera flashlight to bother you
☒ yes ☐ no Do you expect the pupil dilating drops to bother you
☒ yes ☐ no In the past five years have you been examined by an ophthalmologist
☒ yes ☐ no Do you currently carry health insurance
☐ yes ☒ no In some cases the photographs can only be made if your pupils are dilated with a drop. Were you aware of this?

Patient questionnaire

[redacted]ediagnosis results have been discussed

Name: [redacted] **Date:** [redacted]

Circle your satisfaction with digital photography of your retina

1	2	3	4	5
Terrible				Very good

Circle where you think the retinal exam is better:

1	2	3	4	5
Ophthalmologist's office				Telediagnosis

Circle which retinal exam you prefer now:

1	2	3	4	5
Ophthalmologist's office				Telediagnosis

☒ yes ☐ no Did the camera flashlight bother you
☐ yes ☒ no Did the pupil dilating drops bother you

After form is completed, please enter into Excel spreadsheet or send to:

Dr. Michael D. Abramoff, MD, PhD
Department of Ophthalmology and Visual Sciences
PFP 11290C
University of Iowa Hospitals and Clinics
200 Hawkins, Iowa City, IA 52242
Tel (319) 384 5833, fax: (319) 353 7996

[redacted]

[redacted]

Patient questionnaire
BEFORE telediagnosis

Name [redacted] **Date:** [redacted]

Thank you for your willingness to participate in this research project.
You are about to undergo digital photographing and diagnosis over the internet by ophthalmologists who are retinal specialists at the University of Iowa. They will evaluate your photographs for signs of diabetic retinopathy, a complication of diabetes. This new technique is called 'telediagnosis'. Until telediagnosis was available, you could only be screened in an ophthalmologist's office with a dilated retinal exam.

Please give your best answer to the following questions

Circle where you expect the quality of the retinal exam to be better:

1	2	(3)	4	5
Ophthalmologist's office			Telediagnosis	

Circle which retinal exam you prefer:

1	2	(3)	4	5
Ophthalmologist's office			Telediagnosis	

- □ yes □ no Do you expect the camera flashlight to bother you
- □ yes □ no Do you expect the pupil dilating drops to bother you
- □ yes □ no In the past five years have you been examined by an ophthalmologist
- □ yes □ no Do you currently carry health insurance
- □ yes □ no In some cases the photographs can only be made if your pupils are dilated with a drop. Were you aware of this?

Patient questionnaire
AFTER telediagnosis results have been discussed

Name:________________________ **Date:**______________

Circle your satisfaction with digital photography of your retina

1	2	3	4	(5)
Terrible				Very good

Circle where you think the retinal exam is better:

1	2	3	4	(5)
Ophthalmologist's office				Telediagnosis

Circle which retinal exam you prefer now:

1	2	3	4	(5)
Ophthalmologist's office				Telediagnosis

- □ yes ☑ no Did the camera flashlight bother you
- □ yes □ no[redacted]e pupil dilating drops bother you

After form is completed, please enter into Excel spreadsheet or send to:

Dr. Michael D. Abramoff, MD, PhD
Department of Ophthalmology and Visual Sciences
PFP 11290C
University of Iowa Hospitals and Clinics
200 Hawkins, Iowa City, IA 52242
Tel (319) 384 5833, fax: (319) 353 7996

[redacted]

[redacted]

Patient questionnaire

[redacted]EFORE telediagnosis

Name:[redacted]______ **Date:**_[redacted]

Thank you for your willingness to participate in this research project.

You are about to undergo digital photographing and diagnosis over the internet by ophthalmologists who are retinal specialists at the University of Iowa. They will evaluate your photographs for signs of diabetic retinopathy, a complication of diabetes. This new technique is called 'telediagnosis'. Until telediagnosis was available, you could only be screened in an ophthalmologist's office with a dilated retinal exam.

Please give your best answer to the following questions

Circle where you expect the quality of the retinal exam to be better:

1	2	3	4	5
Ophthalmologist's office			Telediagnosis	

Circle which retinal exam you prefer:

1	2	3	4	5
Ophthalmologist's office			Telediagnosis	

□ yes □ no Do you expect the camera flashlight to bother you
□ yes □ no Do you expect the pupil dilating drops to bother you
□ yes □ no In the past five years have you been examined by an ophthalmologist
□ yes □ no Do you currently carry health insurance
□ yes □ no In some cases the photographs can only be made if your pupils are dilated with a drop. Were you aware of this?

[redacted]

Patient questionnaire

[redacted]diagnosis results have been discussed

Name:[redacted]______ **Date:**___[redacted]_

Circle your satisfaction with digital photography of your retina

1	2	3	4	5
Terrible				Very good

Circle where you think the retinal exam is better:

1	2	3	4	5
Ophthalmologist's office				Telediagnosis

Circle which retinal exam you prefer now:

1	2	3	4	5
Ophthalmologist's office				Telediagnosis

□ yes □ no Did the camera flashlight bother you
□ yes □ no Did the pupil dilating drops bother you

After form is completed, please enter into Excel spreadsheet or send to:

Dr. Michael D. Abramoff, MD, PhD
Department of Ophthalmology and Visual Sciences
PFP 11290C
University of Iowa Hospitals and Clinics
200 Hawkins, Iowa City, IA 52242
Tel (319) 384 5833, fax: (319) 353 7996

[redacted]

[redacted]

Patient questionnaire

[redacted] **BEFORE telediagnosis**

Nam[redacted] **Date:**[redacted]

Thank you for your willingness to participate in this research project.
You are about to undergo digital photographing and diagnosis over the internet by ophthalmologists who are retinal specialists at the University of Iowa. They will evaluate your photographs for signs of diabetic retinopathy, a complication of diabetes. This new technique is called 'telediagnosis'. Until telediagnosis was available, you could only be screened in an ophthalmologist's office with a dilated retinal exam.

Please give your best answer to the following questions

Circle where you expect the quality of the retinal exam to be better:

1 2 3 4 5

Ophthalmologist's office Telediagnosis

Circle which retinal exam you prefer:

1 2 3 4 5

Ophthalmologist's office Telediagnosis

□ yes ☒ no Do you expect the camera flashlight to bother you
□ yes ☒ no Do you expect the pupil dilating drops to bother you
☒ yes □ no In the past five years have you been examined by an ophthalmologist
☒ yes □ no Do you currently carry health insurance
☒ yes □ no In some cases the photographs can only be made if your pupils are dilated with a drop. Were you aware of this?

Patient questionnaire

[redacted]**ER telediagnosis results have been discussed**

Name:[redacted] **Date:**[redacted]

Circle your satisfaction with digital photography of your retina

1 2 3 4 5

Terrible Very good

Circle where you think the retinal exam is better:

1 2 3 4 5

Ophthalmologist's office Telediagnosis

Circle which retinal exam you prefer now:

1 2 3 4 5

Ophthalmologist's office Telediagnosis

[redacted]

□ yes ☒ no Did the camera flashlight bother you
□ yes ☒ no Did the pupil dilating drops bother you

After form is completed, please enter into Excel spreadsheet or send to:

Dr. Michael D. Abramoff, MD, PhD
Department of Ophthalmology and Visual Sciences
PFP 11290C
University of Iowa Hospitals and Clinics
200 Hawkins, Iowa City, IA 52242
Tel (319) 384 5833, fax: (319) 353 7996

[redacted]

[redacted]

Patient questionnaire

BEFORE telediagnosis

Name: [redacted] **Date:** [redacted]

Thank you for your willingness to participate in this research project.

You are about to undergo digital photographing and diagnosis over the internet by ophthalmologists who are retinal specialists at the University of Iowa. They will evaluate your photographs for signs of diabetic retinopathy, a complication of diabetes. This new technique is called 'telediagnosis'. Until telediagnosis was available, you could only be screened in an ophthalmologist's office with a dilated retinal exam.

Please give your best answer to the following questions

Circle where you expect the quality of the retinal exam to be better:

1	2	3	4	5
Ophthalmologist's office			Telediagnosis	

Circle which retinal exam you prefer:

1	2	3	4	5
Ophthalmologist's office			Telediagnosis	

□ yes ☒ no Do you expect the camera flashlight to bother you
□ yes ☒ no Do you expect the pupil dilating drops to bother you
☒ yes □ no In the past five years have you been examined by an ophthalmologist
☒ yes □ no Do you currently carry health insurance
□ yes ☒ no In some cases the photographs can only be made if your pupils are dilated with a drop. Were you aware of this?

Patient questionnaire

[redacted]**iagnosis results have been discussed**

Name: [redacted] **Date:** [redacted]

Circle your satisfaction with digital photography of your retina

1	2	3	4	5
Terrible				Very good

Circle where you think the retinal exam is better:

1	2	3	4	5
Ophthalmologist's office				Telediagnosis

Circle which retinal exam you prefer now:

1	2	3	4	5
Ophthalmologist's office				Telediagnosis

□ yes ☒ no Did the camera flashlight bother you
□ yes [redacted] the pupil dilating drops bother you

After form is completed, please enter into Excel spreadsheet or send to:

Dr. Michael D. Abramoff, MD, PhD
Department of Ophthalmology and Visual Sciences
PFP 11290C
University of Iowa Hospitals and Clinics
200 Hawkins, Iowa City, IA 52242
Tel (319) 384 5833, fax: (319) 353 7996

[redacted]

Patient questionnaire

BEFORE tel[redacted]

Name: [redacted] **Date**[redacted]

Thank you for your willingness to participate in this research project.
You are about to undergo digital photographing and diagnosis over the internet by ophthalmologists who are retinal specialists at the University of Iowa. They will evaluate your photographs for signs of diabetic retinopathy, a complication of diabetes. This new technique is called 'telediagnosis'. Until telediagnosis was available, you could only be screened in an ophthalmologist's office with a dilated retinal exam.

Please give your best answer to the following questions

Circle where you expect the quality of the retinal exam to be better:

1	2	3	4	5
Ophthalmologist's office			Telediagnosis	

Circle which retinal exam you prefer:

1	2	3	4	5
Ophthalmologist's office			Telediagnosis	

- ☐ yes ☐ no Do you expect the camera flashlight to bother you
- ☐ yes ☐ no Do you expect the pupil dilating drops to bother you
- ☐ yes ☐ no In the past five years have you been examined by an ophthalmologist
- ☐ yes ☐ no Do you currently carry health insurance
- ☐ yes ☐ no In some cases the photographs can only be made if your pupils are dilated with a drop. Were you aware of this?

Patient questionnaire

[redacted]diagnosis results have been discussed

Name: [redacted] **Date:** [redacted]

Circle your satisfaction with digital photography of your retina

1	2	3	4	5
Terrible				Very good

Circle where you think the retinal exam is better:

1	2	3	4	5
Ophthalmologist's office				Telediagnosis

Circle which retinal exam you prefer now:

1	2	3	4	5
Ophthalmologist's office				Telediagnosis

- ☐ yes ☐ no Did the camera flashlight bother you
- ☐ yes ☐ no Did the pupil dilating drops bother you

After form is completed, please enter into Excel spreadsheet or send to:

Dr. Michael D. Abramoff, MD, PhD
Department of Ophthalmology and Visual Sciences
PFP 11290C
University of Iowa Hospitals and Clinics
200 Hawkins, Iowa City, IA 52242
Tel (319) 384 5833, fax: (319) 353 7996

[redacted]

[redacted]

Patient questionnaire

BEFORE telediagnosis

Name:[redacted] **Date:**[redacted]

Thank you for your willingness to participate in this research project.
You are about to undergo digital photographing and diagnosis over the internet by ophthalmologists who are retinal specialists at the University of Iowa. They will evaluate your photographs for signs of diabetic retinopathy, a complication of diabetes. This new technique is called 'telediagnosis'. Until telediagnosis was available, you could only be screened in an ophthalmologist's office with a dilated retinal exam.

Please give your best answer to the following questions

Circle where you expect the quality of the retinal exam to be better:

1	2	3	4	5
Ophthalmologist's office			Telediagnosis	

Circle which retinal exam you prefer:

1	2	3	4	5
Ophthalmologist's office			Telediagnosis	

□ yes □ no Do you expect the camera flashlight to bother you
□ yes □ no Do you expect the pupil dilating drops to bother you
□ yes □ no In the past five years have you been examined by an ophthalmologist
□ yes □ no Do you currently carry health insurance
□ yes □ no In some cases the photographs can only be made if your pupils are dilated with a drop. Were you aware of this?

Patient questionnaire

AFTER telediagnosis results have been discussed

Name:____________________________ **Date:**______________

Circle your satisfaction with digital photography of your retina

1	2	3	4	5
Terrible				Very good

Circle where you think the retinal exam is better:

1	2	3	4	5
Ophthalmologist's office				Telediagnosis

Circle which retinal exam you prefer now:

1	2	3	4	5
Ophthalmologist's office				Telediagnosis

□ yes □ no Did the camera flashlight bother you
□ yes □ n[redacted]pil dilating drops bother you

After form is completed, please enter into Excel spreadsheet or send to:
Dr. Michael D. Abramoff, MD, PhD
Department of Ophthalmology and Visual Sciences
PFP 11290C
University of Iowa Hospitals and Clinics
200 Hawkins, Iowa City, IA 52242
Tel (319) 384 5833, fax: (319) 353 7996

[redacted]

[redacted]

Patient questionnaire

BEFORE telediagnosis

Name: [redacted] **Date:** [redacted]

Thank you for your willingness to participate in this research project.
You are about to undergo digital photographing and diagnosis over the internet by ophthalmologists who are retinal specialists at the University of Iowa. They will evaluate your photographs for signs of diabetic retinopathy, a complication of diabetes. This new technique is called 'telediagnosis'. Until telediagnosis was available, you could only be screened in an ophthalmologist's office with a dilated retinal exam.

Please give your best answer to the following questions

Circle where you expect the quality of the retinal exam to be better:

1	2	3	4	5
Ophthalmologist's office			Telediagnosis	

Circle which retinal exam you prefer:

1	2	3	4	5
Ophthalmologist's office			Telediagnosis	

□ yes □ no Do you expect the camera flashlight to bother you
□ yes □ no Do you expect the pupil dilating drops to bother you
□ yes □ no In the past five years have you been examined by an ophthalmologist
□ yes □ no Do you currently carry health insurance
□ yes □ no In some cases the photographs can only be made if your pupils are dilated with a drop. Were you aware of this?

Patient questionnaire

AFTER telediagnosis results have been discussed

Name:______________________________ **Date:**________________

Circle your satisfaction with digital photography of your retina

1	2	3	4	5
Terrible				Very good

Circle where you think the retinal exam is better:

1	2	3	4	5
Ophthalmologist's office				Telediagnosis

Circle which retinal exam you prefer now:

1	2	3	4	5
Ophthalmologist's office				Telediagnosis

□ yes □ no Did the camera flashlight bother you
□ yes □ [redacted] pupil dilating drops bother you

After form is completed, please enter into Excel spreadsheet or send to:
Dr. Michael D. Abramoff, MD, PhD
Department of Ophthalmology and Visual Sciences
PFP 11290C
University of Iowa Hospitals and Clinics
200 Hawkins, Iowa City, IA 52242
Tel (319) 384 5833, fax: (319) 353 7996

[redacted]

[redacted]

Patient questionnaire

[redacted]ORE telediagnosis

Name:[redacted] **Date:**[redacted]

Thank you for your willingness to participate in this research project.
You are about to undergo digital photographing and diagnosis over the internet by ophthalmologists who are retinal specialists at the University of Iowa. They will evaluate your photographs for signs of diabetic retinopathy, a complication of diabetes. This new technique is called 'telediagnosis'. Until telediagnosis was available, you could only be screened in an ophthalmologist's office with a dilated retinal exam.

Please give your best answer to the following questions

Circle where you expect the quality of the retinal exam to be better:

1	2	3	4	5
Ophthalmologist's office			Telediagnosis	

Circle which retinal exam you prefer:

1	2	3	4	5
Ophthalmologist's office			Telediagnosis	

□ yes □ no Do you expect the camera flashlight to bother you
□ yes □ no Do you expect the pupil dilating drops to bother you
□ yes □ no In the past five years have you been examined by an ophthalmologist
□ yes □ no Do you currently carry health insurance
□ yes □ no In some cases the photographs can only be made if your pupils are dilated with a drop. Were you aware of this?

Patient questionnaire

AFTER telediagnosis results have been discussed

Name:____________________ **Date:**__________

Circle your satisfaction with digital photography of your retina

1	2	3	4	5
Terrible				Very good

Circle where you think the retinal exam is better:

1	2	3	4	5
Ophthalmologist's office				Telediagnosis

Circle which retinal exam you prefer now:

1	2	3	4	5
Ophthalmologist's office				Telediagnosis

□ yes □ no Did the camera flashlight bother you [redacted]
□ yes □ no Did [redacted]ing drops bother you

After form is completed, please enter into Excel spreadsheet or send to:

Dr. Michael D. Abramoff, MD, PhD
Department of Ophthalmology and Visual Sciences
PFP 11290C
University of Iowa Hospitals and Clinics
200 Hawkins, Iowa City, IA 52242
Tel (319) 384 5833, fax: (319) 353 7996

[redacted]

[redacted]

Patient questionnaire

BEFORE telediagnosis

Name: [redacted] **Date** [redacted]

Thank you for your willingness to participate in this research project.
You are about to undergo digital photographing and diagnosis over the internet by ophthalmologists who are retinal specialists at the University of Iowa. They will evaluate your photographs for signs of diabetic retinopathy, a complication of diabetes. This new technique is called 'telediagnosis'. Until telediagnosis was available, you could only be screened in an ophthalmologist's office with a dilated retinal exam.

Please give your best answer to the following questions

Circle where you expect the quality of the retinal exam to be better:

1 2 3 (4) 5

Ophthalmologist's office — Telediagnosis

Circle which retinal exam you prefer:

1 2 3 (4) 5

Ophthalmologist's office — Telediagnosis

☐ yes ☑ no Do you expect the camera flashlight to bother you
☑ yes ☐ no Do you expect the pupil dilating drops to bother you
☑ yes ☐ no In the past five years have you been examined by an ophthalmologist
☑ yes ☐ no Do you currently carry health insurance
☐ yes ☑ no In some cases the photographs can only be made if your pupils are dilated with a drop. Were you aware of this?

Patient questionnaire

[redacted]ER telediagnosis results have been discussed

Name: [redacted] **Date:** [redacted]

Circle your satisfaction with digital photography of your retina

1 2 3 (4) 5

Terrible — Very good

Circle where you think the retinal exam is better:

1 2 3 (4) 5

Ophthalmologist's office — Telediagnosis

Circle which retinal exam you prefer now:

1 2 3 (4) 5

Ophthalmologist's office — Telediagnosis

☐ yes ☑ no Did the camera flashlight bother you
☑ yes ☐ no Did the pupil dilating drops bother you

After form is completed, please enter into Excel spreadsheet or send to:

Dr. Michael D. Abramoff, MD, PhD
Department of Ophthalmology and Visual Sciences
PFP 11290C
University of Iowa Hospitals and Clinics
200 Hawkins, Iowa City, IA 52242
Tel (319) 384 5833, fax: (319) 353 7996

[redacted]

[redacted]

Patient questionnaire

BEFORE telediagnosis

Name: [redacted] **Date** [redacted]

Thank you for your willingness to participate in this research project.
You are about to undergo digital photographing and diagnosis over the internet by ophthalmologists who are retinal specialists at the University of Iowa. They will evaluate your photographs for signs of diabetic retinopathy, a complication of diabetes. This new technique is called 'telediagnosis'. Until telediagnosis was available, you could only be screened in an ophthalmologist's office with a dilated retinal exam.

Please give your best answer to the following questions

Circle where you expect the quality of the retinal exam to be better:

1 2 3 4 5
Ophthalmologist's office — Telediagnosis

Circle which retinal exam you prefer:

1 2 3 4 5
Ophthalmologist's office — Telediagnosis

- ☐ yes ☑ no Do you expect the camera flashlight to bother you
- ☐ yes ☑ no Do you expect the pupil dilating drops to bother you
- ☐ yes ☑ no In the past five years have you been examined by an ophthalmologist
- ☑ yes ☐ no Do you currently carry health insurance
- ☑ yes ☐ no In some cases the photographs can only be made if your pupils are dilated with a drop. Were you aware of this?

Patient questionnaire

[redacted] **telediagnosis results have been discussed**

Name: [redacted] **Date:** ____________

Circle your satisfaction with digital photography of your retina

1 2 3 4 5
Terrible — Very good

Circle where you think the retinal exam is better:

1 2 3 4 5
Ophthalmologist's office — Telediagnosis

Circle which retinal exam you prefer now:

1 2 3 4 5
Ophthalmologist's office — Telediagnosis

- ☐ yes ☑ no Did the camera flashlight bother you
- ☐ yes ☐ [redacted] e pupil dilating drops bother you

After form is completed, please enter into Excel spreadsheet or send to:

Dr. Michael D. Abramoff, MD, PhD
Department of Ophthalmology and Visual Sciences
PFP 11290C
University of Iowa Hospitals and Clinics
200 Hawkins, Iowa City, IA 52242
Tel (319) 384 5833, fax: (319) 353 7996

[redacted]

[redacted]

Patient questionnaire

BEFORE telediagnosis

Name: [redacted] **Date:** [redacted]

Thank you for your willingness to participate in this research project.

You are about to undergo digital photographing and diagnosis over the internet by ophthalmologists who are retinal specialists at the University of Iowa. They will evaluate your photographs for signs of diabetic retinopathy, a complication of diabetes. This new technique is called 'telediagnosis'. Until telediagnosis was available, you could only be screened in an ophthalmologist's office with a dilated retinal exam.

Please give your best answer to the following questions

Circle where you expect the quality of the retinal exam to be better:

1 2 3 (4) 5

Ophthalmologist's office — Telediagnosis

Circle which retinal exam you prefer:

1 2 3 (4) 5

Ophthalmologist's office — Telediagnosis

- ☐ yes ☒ no Do you expect the camera flashlight to bother you
- ☐ yes ☒ no Do you expect the pupil dilating drops to bother you
- ☒ yes ☐ no In the past five years have you been examined by an ophthalmologist
- ☒ yes ☐ no Do you currently carry health insurance
- ☒ yes ☐ no In some cases the photographs can only be made if your pupils are dilated with a drop. Were you aware of this?

Patient questionnaire

[redacted]ediagnosis results have been discussed

Name: [redacted] **Date:** [redacted]

Circle your satisfaction with digital photography of your retina

1 2 3 4 (5)

Terrible — Very good

Circle where you think the retinal exam is better:

1 2 3 (4) 5

Ophthalmologist's office — Telediagnosis

Circle which retinal exam you prefer now:

1 2 3 (4) 5

Ophthalmologist's office — Telediagnosis

- ☐ yes ☒ no Did the camera flashlight bother you
- ☐ yes ☒ no Did the pupil dilating drops bother you

After form is completed, please enter into Excel spreadsheet or send to:

Dr. Michael D. Abramoff, MD, PhD
Department of Ophthalmology and Visual Sciences
PFP 11290C
University of Iowa Hospitals and Clinics
200 Hawkins, Iowa City, IA 52242
Tel (319) 384 5833, fax: (319) 353 7996

[redacted]

[redacted]

Patient questionnaire

BEFORE telediagnosis

Name:[redacted] **Date:**[redacted]

Thank you for your willingness to participate in this research p[redacted]

You are about to undergo digital photographing and diagnosis over the internet by ophthalmologists who are retinal specialists at the University of Iowa. They will evaluate your photographs for signs of diabetic retinopathy, a complication of diabetes. This new technique is called 'telediagnosis'. Until telediagnosis was available, you could only be screened in an ophthalmologist's office with a dilated retinal exam.

Please give your best answer to the following questions

Circle where you expect the quality of the retinal exam to be better:

1	2	3	4	5
Ophthalmologist's office			Telediagnosis	

Circle which retinal exam you prefer:

1	2	3	4	5
Ophthalmologist's office			Telediagnosis	

☐ yes ☑ no Do you expect the camera flashlight to bother you

☐ yes ☐ no Do you expect the pupil dilating drops to bother you

☑ yes ☐ no In the past five years have you been examined by an ophthalmologist

☑ yes ☐ no Do you currently carry health insurance

☐ yes ☐ no In some cases the photographs can only be made if your pupils are dilated with a drop. Were you aware of this?

Patient questionnaire

AFTER telediagnosis results have been discussed

Name:____________________ **Date:**____________

Circle your satisfaction with digital photography of your retina

1	2	3	4	(5)
Terrible				Very good

Circle where you think the retinal exam is better:

1	2	3	4	(5)
Ophthalmologist's office				Telediagnosis

Circle which retinal exam you prefer now:

1	2	3	4	(5)
Ophthalmologist's office				Telediagnosis

☐ yes ☑ no Did the camera flashlight bother you

☐ yes ☐ no Did the pupil dilating drops bother you

After form is completed, please enter into Excel spreadsheet or send to:

Dr. Michael D. Abramoff, MD, PhD
Department of Ophthalmology and Visual Sciences
PFP 11290C
University of Iowa Hospitals and Clinics
200 Hawkins, Iowa City, IA 52242
Tel (319) 384 5833, fax: (319) 353 7996

[redacted]

[redacted]

Patient questionnaire

BEFORE telediagnosis

Name:[redacted] **Dat**[redacted]

Thank you for your willingness to participate in this research project.

You are about to undergo digital photographing and diagnosis over the internet by ophthalmologists who are retinal specialists at the University of Iowa. They will evaluate your photographs for signs of diabetic retinopathy, a complication of diabetes. This new technique is called 'telediagnosis'. Until telediagnosis was available, you could only be screened in an ophthalmologist's office with a dilated retinal exam.

Please give your best answer to the following questions

Circle where you expect the quality of the retinal exam to be better:

1	2	3	4	5
Ophthalmologist's office			(Telediagnosis) circled	

Circle which retinal exam you prefer:

1	2	3	4	5
Ophthalmologist's office			(Telediagnosis) circled	

☐ yes ☑ no Do you expect the camera flashlight to bother you
☑ yes ☐ no Do you expect the pupil dilating drops to bother you
☑ yes ☐ no In the past five years have you been examined by an ophthalmologist
☑ yes ☐ no Do you currently carry health insurance
☐ yes ☑ no In some cases the photographs can only be made if your pupils are dilated with a drop. Were you aware of this?

Patient questionnaire

[redacted]lediagnosis results have been discussed

Name:[redacted] **Date:**[redacted]

Circle your satisfaction with digital photography of your retina

1	2	3	4	5
Terrible				(Very good) circled

Circle where you think the retinal exam is better:

1	2	3	4	5
Ophthalmologist's office				(Telediagnosis) circled

Circle which retinal exam you prefer now:

1	2	3	4	5
Ophthalmologist's office				(Telediagnosis) circled

☐ yes ☑ no Did the camera flashlight bother you
☐ yes ☑ no Did the pupil dilating drops bother you

After form is completed, please enter into Excel spreadsheet or send to:

Dr. Michael D. Abramoff, MD, PhD
Department of Ophthalmology and Visual Sciences
PFP 11290C
University of Iowa Hospitals and Clinics
200 Hawkins, Iowa City, IA 52242
Tel (319) 384 5833, fax: (319) 353 7996

[redacted]

[redacted]

Patient questionnaire

BEFORE telediagnosis

Name: [redacted] Date: [redacted]

Thank you for your willingness to participate in this research project.

You are about to undergo digital photographing and diagnosis over the internet by ophthalmologists who are retinal specialists at the University of Iowa. They will evaluate your photographs for signs of diabetic retinopathy, a complication of diabetes. This new technique is called 'telediagnosis'. Until telediagnosis was available, you could only be screened in an ophthalmologist's office with a dilated retinal exam.

Please give your best answer to the following questions

Circle where you expect the quality of the retinal exam to be better:

1 Ophthalmologist's office — 2 — 3 — 4 Telediagnosis — (5) [circled]

Circle which retinal exam you prefer:

1 Ophthalmologist's office — 2 — 3 — 4 Telediagnosis — 5

- ☐ yes ☑ no — Do you expect the camera flashlight to bother you
- ☐ yes ☑ no — Do you expect the pupil dilating drops to bother you
- ☑ yes ☐ no — In the past five years have you been examined by an ophthalmologist
- ☑ yes ☐ no — Do you currently carry health insurance
- ☑ yes ☐ no — In some cases the photographs can only be made if your pupils are dilated with [redacted] drop. Were you aware of this?

Patient questionnaire

AFTER telediagnosis results have been discussed

Name:____________________ Date:____________

Circle your satisfaction with digital photography of your retina

1 Terrible — 2 — 3 — 4 — 5 Very good

Circle where you think the retinal exam is better:

1 Ophthalmologist's office — 2 — 3 — 4 — 5 Telediagnosis

Circle which retinal exam you prefer now:

1 Ophthalmologist's office — 2 — 3 — 4 — 5 Telediagnosis

- ☐ yes ☐ no — Did the camera flashlight bother you
- ☐ yes ☐ no — Did the pupil dilating drops bother you

After form is completed, please enter into Excel spreadsheet or send to: [illegible]

Dr. Michael D. Abramoff, MD, PhD
Department of Ophthalmology and Visual Sciences
PFP 11290C
University of Iowa Hospitals and Clinics
200 Hawkins, Iowa City, IA 52242
Tel (319) 384 5833, fax: (319) 353 7996

[redacted]

[redacted]

[redacted]

Patient questionnaire

BEFORE telediagnosis

Name: [redacted] **Date:** [redacted]

Thank you for your willingness to participate in this research project.

You are about to undergo digital photographing and diagnosis over the internet by ophthalmologists who are retinal specialists at the University of Iowa. They will evaluate your photographs for signs of diabetic retinopathy, a complication of diabetes. This new technique is called 'telediagnosis'. Until telediagnosis was available, you could only be screened in an ophthalmologist's office with a dilated retinal exam.

Please give your best answer to the following questions

Circle where you expect the quality of the retinal exam to be better:

1	2	(3)	4	5
Ophthalmologist's office			Telediagnosis	

Circle which retinal exam you prefer:

1	2	(3)	4	5
Ophthalmologist's office			Telediagnosis	

- □ yes ☑ no Do you expect the camera flashlight to bother you
- □ yes ☑ no Do you expect the pupil dilating drops to bother you
- ☑ yes □ no In the past five years have you been examined by an ophthalmologist
- ☑ yes □ no Do you currently carry health insurance
- ☑ yes □ no In some cases the photographs can only be made if your pupils are dilated with a drop. Were you aware of this?

Patient questionnaire

[redacted]agnosis results have been discussed

Name: [redacted] **Date:** [redacted]

Circle your satisfaction with digital photography of your retina

1	2	(3)	4	5
Terrible				Very good

Circle where you think the retinal exam is better:

1	2	(3)	4	5
Ophthalmologist's office				Telediagnosis

Circle which retinal exam you prefer now:

1	2	(3)	4	5
Ophthalmologist's office				Telediagnosis

- □ yes ☑ no Did the camera flashlight bother you
- □ yes □ [redacted]d the pupil dilating drops bother you

After form is completed, please enter into Excel spreadsheet or send to:

Dr. Michael D. Abramoff, MD, PhD
Department of Ophthalmology and Visual Sciences
PFP 11290C
University of Iowa Hospitals and Clinics
200 Hawkins, Iowa City, IA 52242
Tel (319) 384 5833, fax: (319) 353 7996

[redacted]

[redacted]

Patient questionnaire
BEFORE telediagnosis

Name: [redacted] **Date:** [redacted]

Thank you for your willingness to participate in this research project.
You are about to undergo digital photographing and diagnosis over the internet by ophthalmologists who are retinal specialists at the University of Iowa. They will evaluate your photographs for signs of diabetic retinopathy, a complication of diabetes. This new technique is called 'telediagnosis'. Until telediagnosis was available, you could only be screened in an ophthalmologist's office with a dilated retinal exam.

Please give your best answer to the following questions

Circle where you expect the quality of the retinal exam to be better:

1 2 3 4 5
Ophthalmologist's office Telediagnosis

Circle which retinal exam you prefer:

1 2 3 4 5
Ophthalmologist's office Telediagnosis

- ☐ yes ☐ no Do you expect the camera flashlight to bother you
- ☑ yes ☐ no Do you expect the pupil dilating drops to bother you
- ☐ yes ☑ no In the past five years have you been examined by an ophthalmologist
- ☑ yes ☐ no Do you currently carry health insurance
- ☐ yes ☑ no In some cases the photographs can only be made if your pupils are dilated with a drop. Were you aware of this?

Patient questionnaire
AFTER telediagnosis results have been discussed

Name: [redacted] **Date:** [redacted]

Circle your satisfaction with digital photography of your retina

1 2 3 4 5
Terrible Very good

Circle where you think the retinal exam is better:

1 2 3 4 5
Ophthalmologist's office Telediagnosis

Circle which retinal exam you prefer now:

1 2 3 4 5
Ophthalmologist's office Telediagnosis

- ☑ yes ☐ no Did the camera flashlight bother you [redacted]
- ☐ yes ☑ no Did the pupil dilating drops bother you

After form is completed, please enter into Excel spreadsheet or send to:

Dr. Michael D. Abramoff, MD, PhD
Department of Ophthalmology and Visual Sciences
PFP 11290C
University of Iowa Hospitals and Clinics
200 Hawkins, Iowa City, IA 52242
Tel (319) 384 5833, fax: (319) 353 7996

[redacted]

[redacted]

Patient questionnaire

BEFORE telediagnosis

Nam[redacted] Date:_[redacted]

Thank you for your willingness to participate in this research project.
You are about to undergo digital photographing and diagnosis over the internet by ophthalmologists who are retinal specialists at the University of Iowa. They will evaluate your photographs for signs of diabetic retinopathy, a complication of diabetes. This new technique is called 'telediagnosis'. Until telediagnosis was available, you could only be screened in an ophthalmologist's office with a dilated retinal exam.

Please give your best answer to the following questions

Circle where you expect the quality of the retinal exam to be better:

1	2	3	4	5
Ophthalmologist's office			Telediagnosis	

Circle which retinal exam you prefer:

1	2	3	4	5
Ophthalmologist's office			Telediagnosis	

☐ yes ☐ no Do you expect the camera flashlight to bother you
☐ yes ☐ no Do you expect the pupil dilating drops to bother you
☐ yes ☐ no In the past five years have you been examined by an ophthalmologist
☐ yes ☐ no Do you currently carry health insurance
☐ yes ☐ no In some cases the photographs can only be made if your pupils are dilated with a drop. Were you aware of this?

Patient questionnaire

AFTER telediagnosis results have been discussed

Name:______________________________ **Date:**________________

Circle your satisfaction with digital photography of your retina

1	2	3	4	5
Terrible				Very good

Circle where you think the retinal exam is better:

1	2	3	4	5
Ophthalmologist's office				Telediagnosis

Circle which retinal exam you prefer now:

1	2	3	4	5
Ophthalmologist's office				Telediagnosis

☐ yes ☐ no Did the camera flashlight bother you
☐ y[redacted]he pupil dilating drops bother you

After form is completed, please enter into Excel spreadsheet or send to:
Dr. Michael D. Abramoff, MD, PhD
Department of Ophthalmology and Visual Sciences
PFP 11290C
University of Iowa Hospitals and Clinics
200 Hawkins, Iowa City, IA 52242
Tel (319) 384 5833, fax: (319) 353 7996

[redacted]

[redacted]

Patient questionnaire

BEFORE telediagnosis

Name:[redacted]ate[redacted]

Thank you for your willingness to participate in this research project.

You are about to undergo digital photographing and diagnosis over the internet by ophthalmologists who are retinal specialists at the University of Iowa. They will evaluate your photographs for signs of diabetic retinopathy, a complication of diabetes. This new technique is called 'telediagnosis'. Until telediagnosis was available, you could only be screened in an ophthalmologist's office with a dilated retinal exam.

Please give your best answer to the following questions

Circle where you expect the quality of the retinal exam to be better:

1	2 (circled)	3	4	5
Ophthalmologist's office (circled)			Telediagnosis	

Circle which retinal exam you prefer:

1	2	3	4 (circled)	5
Ophthalmologist's office			Telediagnosis (circled)	

- ☐ yes ☒ no Do you expect the camera flashlight to bother you
- ☐ yes ☒ no Do you expect the pupil dilating drops to bother you
- ☒ yes ☐ no In the past five years have you been examined by an ophthalmologist
- ☒ yes ☐ no Do you currently carry health insurance
- ☐ yes ☒ no In some cases the photographs can only be made if your pupils are dilated with a drop. Were you aware of this?

Patient questionnaire

[redacted]diagnosis results have been discussed

Name:[redacted]________ **Date:**[redacted]__

Circle your satisfaction with digital photography of your retina

1	2	3	4 (circled)	5
Terrible				Very good

Circle where you think the retinal exam is better:

1	2	3	4 (circled)	5
Ophthalmologist's office				Telediagnosis (circled)

Circle which retinal exam you prefer now:

1	2	3	4 (circled)	5
Ophthalmologist's office				Telediagnosis (circled)

- ☐ yes ☒ no Did the camera flashlight bother you
- ☐ yes ☒ no Did the pupil dilating drops bother you

After form is completed, please enter into Excel spreadsheet or send to:

Dr. Michael D. Abramoff, MD, PhD
Department of Ophthalmology and Visual Sciences
PFP 11290C
University of Iowa Hospitals and Clinics
200 Hawkins, Iowa City, IA 52242
Tel (319) 384 5833, fax: (319) 353 7996

[redacted]

[redacted]

Patient questionnaire

BEFORE telediagnosis

Name: [redacted] Date: [redacted]

Thank you for your willingness to participate in this research project.

You are about to undergo digital photographing and diagnosis over the internet by ophthalmologists who are retinal specialists at the University of Iowa. They will evaluate your photographs for signs of diabetic retinopathy, a complication of diabetes. This new technique is called 'telediagnosis'. Until telediagnosis was available, you could only be screened in an ophthalmologist's office with a dilated retinal exam.

Please give your best answer to the following questions

Circle where you expect the quality of the retinal exam to be better:

1	2	3	4	5
Ophthalmologist's office			Telediagnosis	

Circle which retinal exam you prefer:

1	2	3	4	5
Ophthalmologist's office			Telediagnosis	

- □ yes □ no Do you expect the camera flashlight to bother you
- □ yes □ no Do you expect the pupil dilating drops to bother you
- □ yes □ no In the past five years have you been examined by an ophthalmologist
- □ yes □ no Do you currently carry health insurance
- □ yes □ no In some cases the photographs can only be made if your pupils are dilated with a drop. Were you aware of this?

Patient questionnaire

AFTER telediagnosis results have been discussed

Name:____________________ Date:____________

Circle your satisfaction with digital photography of your retina

1	2	3	4	5
Terrible				Very good

Circle where you think the retinal exam is better:

1	2	3	4	5
Ophthalmologist's office				Telediagnosis

Circle which retinal exam you prefer now:

1	2	3	4	5
Ophthalmologist's office				Telediagnosis

- □ yes □ no Did the camera flashlight bother you
- □ yes [redacted] the pupil dilating drops bother you

After form is completed, please enter into Excel spreadsheet or send to:

Dr. Michael D. Abramoff, MD, PhD
Department of Ophthalmology and Visual Sciences
PFP 11290C
University of Iowa Hospitals and Clinics
200 Hawkins, Iowa City, IA 52242
Tel (319) 384 5833, fax: (319) 353 7996

[redacted]

[redacted]

Patient questionnaire

BEFORE telediagnosis

Name [redacted]__________ **Date:**_[redacted]

Thank you for your willingness to participate in this research project.

You are about to undergo digital photographing and diagnosis over the internet by ophthalmologists who are retinal specialists at the University of Iowa. They will evaluate your photographs for signs of diabetic retinopathy, a complication of diabetes. This new technique is called 'telediagnosis'. Until telediagnosis was available, you could only be screened in an ophthalmologist's office with a dilated retinal exam.

Please give your best answer to the following questions

Circle where you expect the quality of the retinal exam to be better:

1	2	3	4	5
Ophthalmologist's office			Telediagnosis	

Circle which retinal exam you prefer:

1	2	3	4	5
Ophthalmologist's office			Telediagnosis	

□ yes □ no Do you expect the camera flashlight to bother you [redacted]
□ yes □ no Do you expect the pupil dilating drops to bother yo[redacted]
□ yes □ no In the past five years have you been examined by an [redacted]
□ yes □ no Do you currently carry health insurance
□ yes □ no In some cases the photographs can only be made if your pupils are dilated with a drop. Were you aware of this?

Patient questionnaire

AFTER telediagnosis results have been discussed

Name:________________________________ **Date:**______________

Circle your satisfaction with digital photography of your retina

1	2	3	4	5
Terrible				Very good

Circle where you think the retinal exam is better:

1	2	3	4	5
Ophthalmologist's office				Telediagnosis

Circle which retinal exam you prefer now:

1	2	3	4	5
Ophthalmologist's office				Telediagnosis

□ yes □ no Did the camera flashlight bother you
□ yes □ no Did the [redacted] drops bother you

After form is completed, please enter into Excel spreadsheet or send to:

Dr. Michael D. Abramoff, MD, PhD
Department of Ophthalmology and Visual Sciences
PFP 11290C
University of Iowa Hospitals and Clinics
200 Hawkins, Iowa City, IA 52242
Tel (319) 384 5833, fax: (319) 353 7996

[redacted]

[redacted]

[redacted]

Patient questionnaire

BEFORE tele[redacted]

Name:[redacted]_____ **Date:**[redacted]

Thank you for your willingness to participate in this research [redacted]

You are about to undergo digital photographing and diagnosis over the internet by ophthalmologists who are retinal specialists at the University of Iowa. They will evaluate your photographs for signs of diabetic retinopathy, a complication of diabetes. This new technique is called 'telediagnosis'. Until telediagnosis was available, you could only be screened in an ophthalmologist's office with a dilated retinal exam.

Please give your best answer to the following questions

Circle where you expect the quality of the retinal exam to be better:

1	2	3	4	5
Ophthalmologist's office			Telediagnosis	

Circle which retinal exam you prefer:

1	2	3	4	5
Ophthalmologist's office			Telediagnosis	

- □ yes □ no Do you expect the camera flashlight to bother you
- □ yes □ no Do you expect the pupil dilating drops to bother you
- □ yes □ no In the past five years have you been examined by an ophthalmologist
- □ yes □ no Do you currently carry health insurance
- □ yes □ no In some cases the photographs can only be made if your pupils are dilated with a drop. Were you aware of this?

Patient questionnaire

AFTER telediagnosis results have been discussed

Name:______________________ **Date:**__________

Circle your satisfaction with digital photography of your retina

1	2	3	4	5
Terrible				Very good

Circle where you think the retinal exam is better:

1	2	3	4	5
Ophthalmologist's office				Telediagnosis

Circle which retinal exam you prefer now:

1	2	3	4	5
Ophthalmologist's office				Telediagnosis

- □ yes □ no Did the camera flashlight bother you
- □ yes □ no Did the pupil dilating drops bother you

After form is completed, please enter into Excel spreadsheet or send to:

Dr. Michael D. Abramoff, MD, PhD
Department of Ophthalmology and Visual Sciences
PFP 11290C
University of Iowa Hospitals and Clinics
200 Hawkins, Iowa City, IA 52242
Tel (319) 384 5833, fax: (319) 353 7996

[redacted]

[redacted]

Patient questionnaire

BEFORE telediagnosis

Name: [redacted]

Thank you for your willingness to participate in this research project.
You are about to undergo digital photographing and diagnosis over the internet by ophthalmologists who are retinal specialists at the University of Iowa. They will evaluate your photographs for signs of diabetic retinopathy, a complication of diabetes. This new technique is called 'telediagnosis'. Until telediagnosis was available, you could only be screened in an ophthalmologist's office with a dilated retinal exam.

Please give your best answer to the following questions

Circle where you expect the quality of the retinal exam to be better:

1 Ophthalmologist's office — 2 — (3) — 4 Telediagnosis — 5

Circle which retinal exam you prefer:

1 Ophthalmologist's office — 2 — (3) — 4 Telediagnosis — 5

☑ yes ☐ no Do you expect the camera flashlight to bother you
☐ yes ☑ no Do you expect the pupil dilating drops to bother you
☑ yes ☐ no In the past five years have you been examined by an ophthalmologist
☑ yes ☐ no Do you currently carry health insurance
☐ yes ☐ no In some cases the photographs can only be made if your pupils are dilated with a drop. Were you aware of this?

Patient questionnaire

AFTER telediagnosis results have been discussed

Name: [redacted]

Circle your satisfaction with digital photography of your retina

1 Terrible — 2 — (3) — 4 — 5 Very good

Circle where you think the retinal exam is better:

1 Ophthalmologist's office — 2 — (3) — 4 — 5 Telediagnosis

Circle which retinal exam you prefer now:

1 Ophthalmologist's office — 2 — (3) — 4 — 5 Telediagnosis

☐ yes ☑ no Did the camera flashlight bother you
☐ yes ☑ no Did the pupil dilating drops bother you

After form is completed, please enter into Excel spreadsheet or send to:

Dr. Michael D. Abramoff, MD, PhD
Department of Ophthalmology and Visual Sciences
PFP 11290C
University of Iowa Hospitals and Clinics
200 Hawkins, Iowa City, IA 52242
Tel (319) 384 5833, fax: (319) 353 7996

[redacted]

[redacted]

Patient questionnaire

BEFORE telediagnosis

Name: [redacted]

Thank you for your willingness to participate in this research project.

You are about to undergo digital photographing and diagnosis over the internet by ophthalmologists who are retinal specialists at the University of Iowa. They will evaluate your photographs for signs of diabetic retinopathy, a complication of diabetes. This new technique is called 'telediagnosis'. Until telediagnosis was available, you could only be screened in an ophthalmologist's office with a dilated retinal exam.

Please give your best answer to the following questions

Circle where you expect the quality of the retinal exam to be better:

1	2	3	(4)	5
Ophthalmologist's office			Telediagnosis	

Circle which retinal exam you prefer:

1	2	3	(4)	5
Ophthalmologist's office			Telediagnosis	

- ☐ yes ☑ no Do you expect the camera flashlight to bother you
- ☑ yes ☐ no Do you expect the pupil dilating drops to bother you
- ☑ yes ☐ no In the past five years have you been examined by an ophthalmologist
- ☑ yes ☐ no Do you currently carry health insurance
- ☐ yes ☑ no In some cases the photographs can only be made if your pupils are dilated with a drop. Were you aware of this?

Patient questionnaire

AFTER telediagnosis results have been discussed

Name: [redacted]

Circle your satisfaction with digital photography of your retina

1	2	3	(4)	5
Terrible				Very good

Circle where you think the retinal exam is better:

1	2	3	(4)	5
Ophthalmologist's office				Telediagnosis

Circle which retinal exam you prefer now:

1	2	3	(4)	5
Ophthalmologist's office				Telediagnosis

- ☐ yes ☑ no Did the camera flashlight bother you
- ☑ yes ☐ no Did the pupil dilating drops bother you

After form is completed, please enter into Excel spreadsheet or send to:

Dr. Michael D. Abramoff, MD, PhD
Department of Ophthalmology and Visual Sciences
PFP 11290C
University of Iowa Hospitals and Clinics
200 Hawkins, Iowa City, IA 52242
Tel (319) 384 5833, fax: (319) 353 7996

[redacted]

[redacted]

Patient questionnaire

BEFORE telediagnosis

[redacted]

Name:[redacted]

Thank you for your willingness to participate in this research project.

You are about to undergo digital photographing and diagnosis over the internet by ophthalmologists who are retinal specialists at the University of Iowa. They will evaluate your photographs for signs of diabetic retinopathy, a complication of diabetes. This new technique is called 'telediagnosis'. Until telediagnosis was available, you could only be screened in an ophthalmologist's office with a dilated retinal exam.

Please give your best answer to the following questions

Circle where you expect the quality of the retinal exam to be better:

1	2	3	(4)	5
Ophthalmologist's office			Telediagnosis	

Circle which retinal exam you prefer:

1	2	3	(4)	5
Ophthalmologist's office			Telediagnosis	

- ☐ yes ☑ no Do you expect the camera flashlight to bother you
- ☐ yes ☑ no Do you expect the pupil dilating drops to bother you
- ☑ yes ☐ no In the past five years have you been examined by an ophthalmologist
- ☑ yes ☐ no Do you currently carry health insurance
- ☑ yes ☐ no In some cases the photographs can only be made if your pupils are dilated with a drop. Were you aware of this?

Patient questionnaire

AFTER telediagnosis results have been discussed

Name:______________________________ **Date:**______________

Circle your satisfaction with digital photography of your retina

1	2	3	(4)	5
Terrible				Very good

Circle where you think the retinal exam is better:

1	2	3	(4)	5
Ophthalmologist's office				Telediagnosis

Circle which retinal exam you prefer now:

1	2	3	(4)	5
Ophthalmologist's office				Telediagnosis

- ☐ yes ☑ no Did the camera flashlight bother you
- ☐ yes ☑ no Did the pupil dilating drops bother you

After form is completed, please enter into Excel spreadsheet or send to:

Dr. Michael D. Abramoff, MD, PhD
Department of Ophthalmology and Visual Sciences
PFP 11290C
University of Iowa Hospitals and Clinics
200 Hawkins, Iowa City, IA 52242
Tel (319) 384 5833, fax: (319) 353 7996

[redacted]

[redacted]

Patient questionnaire

BEFORE telediagnosis

Name: [redacted]

Thank you for your willingness to participate in this research project.
You are about to undergo digital photographing and diagnosis over the internet by ophthalmologists who are retinal specialists at the University of Iowa. They will evaluate your photographs for signs of diabetic retinopathy, a complication of diabetes. This new technique is called 'telediagnosis'. Until telediagnosis was available, you could only be screened in an ophthalmologist's office with a dilated retinal exam.

Please give your best answer to the following questions

Circle where you expect the quality of the retinal exam to be better:

1	2	3	4	5
Ophthalmologist's office			Telediagnosis	

Circle which retinal exam you prefer:

1	2	3	4	5
Ophthalmologist's office			Telediagnosis	

- □ yes ☑ no Do you expect the camera flashlight to bother you
- □ yes ☑ no Do you expect the pupil dilating drops to bother you
- □ yes ☑ no In the past five years have you been examined by an ophthalmologist
- ☑ yes □ no Do you currently carry health insurance
- ☑ yes □ no In some cases the photographs can only be made if your pupils are dilated with a drop. Were you aware of this?

Patient questionnaire

AFTER telediagnosis results have been discussed

Name:____________________ **Date:**__________

Circle your satisfaction with digital photography of your retina

1	2	3	4	5
Terrible				Very good

Circle where you think the retinal exam is better:

1	2	3	4	5
Ophthalmologist's office				Telediagnosis

Circle which retinal exam you prefer now:

1	2	3	4	5
Ophthalmologist's office				Telediagnosis

- □ yes ☑ no Did the camera flashlight bother you [redacted]
- □ yes □ no Did the pupil dilating drops bother you

After form is completed, please enter into Excel spreadsheet or send to:

Dr. Michael D. Abramoff, MD, PhD
Department of Ophthalmology and Visual Sciences
PFP 11290C
University of Iowa Hospitals and Clinics
200 Hawkins, Iowa City, IA 52242
Tel (319) 384 5833, fax: (319) 353 7996

[redacted]

[redacted]

Patient questionnaire

[redacted]

Name: [redacted]

Thank you for your willingness to participate in this research project.

You are about to undergo digital photographing and diagnosis over the internet by ophthalmologists who are retinal specialists at the University of Iowa. They will evaluate your photographs for signs of diabetic retinopathy, a complication of diabetes. This new technique is called 'telediagnosis'. Until telediagnosis was available, you could only be screened in an ophthalmologist's office with a dilated retinal exam.

Please give your best answer to the following questions

Circle where you expect the quality of the retinal exam to be better:

1	2	3	4	5
Ophthalmologist's office [redacted]			Telediagnosis	

Circle which retinal exam you prefer:

1	2	3	4	5
Ophthalmologist's office			Telediagnosis	

□ yes □ no Do you expect the camera flashlight to bother you
□ yes □ no Do you expect the pupil dilating drops to bother you
□ yes □ no In the past five years have you been examined by an ophthalmologist
□ yes □ no Do you currently carry health insurance
□ yes □ no In some cases the photographs can only be made if your pupils are dilated with a drop. Were you aware of this?

Patient questionnaire

AFTER telediagnosis results have been discussed

Name:____________________________ **Date:**______________

Circle your satisfaction with digital photography of your retina

1	2	3	4	5
Terrible				Very good

Circle where you think the retinal exam is better:

1	2	3	4	5
Ophthalmologist's office				Telediagnosis

Circle which retinal exam you prefer now:

1	2	3	4	5
Ophthalmologist's office				Telediagnosis

□ yes □ no Did the camera flashlight bother you
□ yes □ [redacted]e pupil dilating drops bother you

After form is completed, please enter into Excel spreadsheet or send to:

Dr. Michael D. Abramoff, MD, PhD
Department of Ophthalmology and Visual Sciences
PFP 11290C
University of Iowa Hospitals and Clinics
200 Hawkins, Iowa City, IA 52242
Tel (319) 384 5833, fax: (319) 353 7996

[redacted]

[redacted]

Patient questionnaire

BEFORE telediagnosis

[redacted]

Name: [redacted]

Thank you for your willingness to participate in this research project.
You are about to undergo digital photographing and diagnosis over the internet by ophthalmologists who are retinal specialists at the University of Iowa. They will evaluate your photographs for signs of diabetic retinopathy, a complication of diabetes. This new technique is called 'telediagnosis'. Until telediagnosis was available, you could only be screened in an ophthalmologist's office with a dilated retinal exam.

Please give your best answer to the following questions

Circle where you expect the quality of the retinal exam to be better:

1	2	(3)	4	5
Ophthalmologist's office			Telediagnosis	

Circle which retinal exam you prefer:

1	2	(3)	4	5
Ophthalmologist's office			Telediagnosis	

- ☐ yes ☑ no Do you expect the camera flashlight to bother you
- ☑ yes ☐ no Do you expect the pupil dilating drops to bother you
- ☐ yes ☑ no In the past five years have you been examined by an ophthalmologist
- ☑ yes ☐ no Do you currently carry health insurance
- ☐ yes ☑ no In some cases the photographs can only be made if your pupils are dilated with a drop. Were you aware of this?

Patient questionnaire

AFTER telediagnosis results have been discussed

Name: [redacted]

Circle your satisfaction with digital photography of your retina

1	2	3	4	(5)
Terrible				Very good

Circle where you think the retinal exam is better:

1	2	(3)	4	5
Ophthalmologist's office				Telediagnosis

Circle which retinal exam you prefer now:

1	2	3	(4)	5
Ophthalmologist's office				Telediagnosis

- ☐ yes ☑ no Did the camera flashlight bother you
- ☐ yes ☐ no Did the pupil dilating drops bother you

After form is completed, please enter into Excel spreadsheet or send to:

Dr. Michael D. Abramoff, MD, PhD
Department of Ophthalmology and Visual Sciences
PFP 11290C
University of Iowa Hospitals and Clinics
200 Hawkins, Iowa City, IA 52242
Tel (319) 384 5833, fax: (319) 353 7996

[redacted]

[redacted]

Patient questionnaire

BEFORE telediagnosis

[redacted]

Name: [redacted]

Thank you for your willingness to participate in this research project.

You are about to undergo digital photographing and diagnosis over the internet by ophthalmologists who are retinal specialists at the University of Iowa. They will evaluate your photographs for signs of diabetic retinopathy, a complication of diabetes. This new technique is called 'telediagnosis'. Until telediagnosis was available, you could only be screened in an ophthalmologist's office with a dilated retinal exam.

Please give your best answer to the following questions

Circle where you expect the quality of the retinal exam to be better:

1 2 3 (4) 5

(Ophthalmologist's office) Telediagnosis

[redacted]

Circle which retinal exam you prefer:

1 2 3 (4) 5

(Ophthalmologist's office) Telediagnosis

- ☐ yes ☑ no Do you expect the camera flashlight to bother you
- ☐ yes ☑ no Do you expect the pupil dilating drops to bother you
- ☑ yes ☐ no In the past five years have you been examined by an ophthalmologist
- ☑ yes ☐ no Do you currently carry health insurance
- ☐ yes ☑ no In some cases the photographs can only be made if your pupils are dilated with a drop. Were you aware of this?

Patient questionnaire

AFTER telediagnosis results have been discussed

Name [redacted]

Circle your satisfaction with digital photography of your retina

1 2 3 4 (5)

Terrible (Very good)

Circle where you think the retinal exam is better:

1 2 3 (4) 5

Ophthalmologist's office (Telediagnosis)

Circle which retinal exam you prefer now:

1 2 3 (4) 5

Ophthalmologist's office (Telediagnosis)

- ☐ yes ☑ no Did the camera flashlight bother you
- ☐ yes ☑ no Did the pupil dilating drops bother you

After form is completed, please enter into Excel spreadsheet or send to:

Dr. Michael D. Abramoff, MD, PhD
Department of Ophthalmology and Visual Sciences
PFP 11290C
University of Iowa Hospitals and Clinics
200 Hawkins, Iowa City, IA 52242
Tel (319) 384 5833, fax: (319) 353 7996

[redacted]

[redacted]

Patient questionnaire

BEFORE telediagnosis

Name: [redacted]

Thank you for your willingness to participate in this research project.
You are about to undergo digital photographing and diagnosis over the internet by ophthalmologists who are retinal specialists at the University of Iowa. They will evaluate your photographs for signs of diabetic retinopathy, a complication of diabetes. This new technique is called 'telediagnosis'. Until telediagnosis was available, you could only be screened in an ophthalmologist's office with a dilated retinal exam.

Please give your best answer to the following questions

Circle where you expect the quality of the retinal exam to be better:

1 — Ophthalmologist's office 2 3 (4) 5 — Telediagnosis

Circle which retinal exam you prefer:

1 — Ophthalmologist's office 2 3 (4) 5 — Telediagnosis

- ☐ yes ☒ no — Do you expect the camera flashlight to bother you
- ☐ yes ☒ no — Do you expect the pupil dilating drops to bother you
- ☒ yes ☐ no — In the past five years have you been examined by an ophthalmologist
- ☒ yes ☐ no — Do you currently carry health insurance
- ☐ yes ☒ no — In some cases the photographs can only be made if your pupils are dilated with a drop. Were you aware of this?

Patient questionnaire

[redacted]

Name: [redacted]

Circle your satisfaction with digital photography of your retina

1 — Terrible 2 3 4 (5) — Very good

Circle where you think the retinal exam is better:

1 — Ophthalmologist's office 2 3 4 (5) — Telediagnosis

Circle which retinal exam you prefer now:

1 — Ophthalmologist's office 2 3 4 (5) — Telediagnosis

- ☐ yes ☒ no — Did the camera flashlight bother you
- ☐ yes ☐ no — Did the pupil dilating drops bother you

After form is completed, please enter into Excel spreadsheet or send to:
Dr. Michael D. Abramoff, MD, PhD
Department of Ophthalmology and Visual Sciences
PFP 11290C
University of Iowa Hospitals and Clinics
200 Hawkins, Iowa City, IA 52242
Tel (319) 384 5833, fax: (319) 353 7996

[redacted]

[redacted]

Patient questionnaire

BEFORE telediagnosis

[redacted]

Name: [redacted]

Thank you for your willingness to participate in this research project.

You are about to undergo digital photographing and diagnosis over the internet by ophthalmologists who are retinal specialists at the University of Iowa. They will evaluate your photographs for signs of diabetic retinopathy, a complication of diabetes. This new technique is called 'telediagnosis'. Until telediagnosis was available, you could only be screened in an ophthalmologist's office with a dilated retinal exam.

Please give your best answer to the following questions

Circle where you expect the quality of the retinal exam to be better:

1	2	3	4	5
Ophthalmologist's office			Telediagnosis	

Circle which retinal exam you prefer:

1	2	3	4	5
Ophthalmologist's office			Telediagnosis	

☐ yes ☒ no Do you expect the camera flashlight to bother you
☐ yes ☒ no Do you expect the pupil dilating drops to bother you
☒ yes ☐ no In the past five years have you been examined by an ophthalmologist
☒ yes ☐ no Do you currently carry health insurance
☒ yes ☐ no In some cases the photographs can only be made if your pupils are dilated with a drop. Were you aware of this?

Patient questionnaire

AFTER telediagnosis results have been discussed

Name:____________________ **Date:**__________

Circle your satisfaction with digital photography of your retina

1	2	(3) circled	4	5
Terrible				Very good

Circle where you think the retinal exam is better:

1	2	(3) circled	4	5
Ophthalmologist's office				Telediagnosis

Circle which retinal exam you prefer now:

(1) circled	2	3	4	5
Ophthalmologist's office				Telediagnosis

☐ yes ☒ no Did the camera flashlight bother you
☐ yes ☐ no Did the pupil dilating drops bother you

After form is completed, please enter into Excel spreadsheet or send to:

Dr. Michael D. Abramoff, MD, PhD
Department of Ophthalmology and Visual Sci[redacted]
PFP 11290C
University of Iowa Hospitals and Clinics
200 Hawkins, Iowa City, IA 52242
Tel (319) 384 5833, fax: (319) 353 7996

[redacted]

Patient questionnaire

BEFORE telediagnosis

Name: [redacted]

Thank you for your willingness to participate in this research project.
You are about to undergo digital photographing and diagnosis over the internet by ophthalmologists who are retinal specialists at the University of Iowa. They will evaluate your photographs for signs of diabetic retinopathy, a complication of diabetes. This new technique is called 'telediagnosis'. Until telediagnosis was available, you could only be screened in an ophthalmologist's office with a dilated retinal exam.

Please give your best answer to the following questions

Circle where you expect the quality of the retinal exam to be better:

1 Ophthalmologist's office — 2 — (3) — 4 Telediagnosis — 5

Circle which retinal exam you prefer:

1 Ophthalmologist's office — (2) — 3 — 4 Telediagnosis — 5

- ☐ yes ☑ no Do you expect the camera flashlight to bother you
- ☐ yes ☑ no Do you expect the pupil dilating drops to bother you
- ☑ yes ☐ no In the past five years have you been examined by an ophthalmologist
- ☐ yes ☑ no Do you currently carry health insurance
- ☑ yes ☐ no In some cases the photographs can only be made if your pupils are dilated with a drop. Were you aware of this?

[redacted]

Patient questionnaire

[redacted]

Name [redacted]

Circle your satisfaction with digital photography of your retina

1 Terrible — 2 — 3 — 4 — (5) Very good

Circle where you think the retinal exam is better:

1 Ophthalmologist's office — 2 — (3) — 4 — 5 Telediagnosis

Circle which retinal exam you prefer now:

1 Ophthalmologist's office — 2 — (3) — 4 — 5 Telediagnosis

- ☐ yes ☑ no Did the camera flashlight bother you
- ☐ yes ☑ no Did the pupil dilating drops bother you

After form is completed, please enter into Excel spreadsheet or send to:
Dr. Michael D. Abramoff, MD, PhD
Department of Ophthalmology and Visual Sciences
PFP 11290C
University of Iowa Hospitals and Clinics
200 Hawkins, Iowa City, IA 52242
Tel (319) 384 5833, fax: (319) 353 7996

[redacted]

[redacted]

[redacted]

Patient questionnaire
BEFORE telediagnosis

Name: [redacted]

Thank you for your willingness to participate in this research project.
You are about to undergo digital photographing and diagnosis over the internet by ophthalmologists who are retinal specialists at the University of Iowa. They will evaluate your photographs for signs of diabetic retinopathy, a complication of diabetes. This new technique is called 'telediagnosis'. Until telediagnosis was available, you could only be screened in an ophthalmologist's office with a dilated retinal exam.

Please give your best answer to the following questions

Circle where you expect the quality of the retinal exam to be better:

1 Ophthalmologist's office — 2 — (3) — 4 Telediagnosis — 5

Circle which retinal exam you prefer:

1 Ophthalmologist's office — 2 — (3) — 4 Telediagnosis — 5

☑ yes ☐ no Do you expect the camera flashlight to bother you
☐ yes ☑ no Do you expect the pupil dilating drops to bother you
☑ yes ☐ no In the past five years have you been examined by an ophthalmologist
☑ yes ☐ no Do you currently carry health insurance
☐ yes ☑ no In some cases the photographs can only be made if your pupils are dilated with a drop. Were you aware of this?

Patient questionnaire

Nam[redacted]

Circle your satisfaction with digital photography of your retina

1 Terrible — 2 — 3 — 4 — (5) Very good

Circle where you think the retinal exam is better:

1 Ophthalmologist's office — 2 — (3) — 4 — 5 Telediagnosis

Circle which retinal exam you prefer now:

1 Ophthalmologist's office — 2 — (3) — 4 — 5 Telediagnosis

☑ yes ☐ no Did the camera flashlight bother you
☐ yes ☑ no Did the pupil dilating drops bother you

After form is completed, please enter into Excel spreadsheet or send to:

Dr. Michael D. Abramoff, MD, PhD
Department of Ophthalmology and Visual Sciences
PFP 11290C
University of Iowa Hospitals and Clinics
200 Hawkins, Iowa City, IA 52242
Tel (319) 384 5833, fax: (319) 353 7996

[redacted]

[redacted]

[redacted]

Patient questionnaire

[redacted]

Name: [redacted]

Thank you for your willingness to participate in this research project.
You are about to undergo digital photographing and diagnosis over the internet by ophthalmologists who are retinal specialists at the University of Iowa. They will evaluate your photographs for signs of diabetic retinopathy, a complication of diabetes. This new technique is called 'telediagnosis'. Until telediagnosis was available, you could only be screened in an ophthalmologist's office with a dilated retinal exam.

Please give your best answer to the following questions

Circle where you expect the quality of the retinal exam to be better:

1 Ophthalmologist's office | 2 | 3 | 4 Telediagnosis | 5

Circle which retinal exam you prefer:

1 Ophthalmologist's office | 2 | 3 | 4 Telediagnosis | 5

[redacted]

- ☐ yes ☐ no Do you expect the camera flashlight to bother you
- ☐ yes ☐ no Do you expect the pupil dilating drops to bother you
- ☐ yes ☐ no In the past five years have you been examined by an ophthalmologist
- ☐ yes ☐ no Do you currently carry health insurance
- ☐ yes ☐ no In some cases the photographs can only be made if your pupils are dilated with a drop. Were you aware of this?

Patient questionnaire

AFTER telediagnosis results have been discussed

Name:__________________________ **Date:**______________

Circle your satisfaction with digital photography of your retina

1 Terrible | 2 | 3 | 4 | 5 Very good

Circle where you think the retinal exam is better:

1 Ophthalmologist's office | 2 | 3 | 4 | 5 Telediagnosis

Circle which retinal exam you prefer now:

1 Ophthalmologist's office | 2 | 3 | 4 | 5 Telediagnosis

- ☐ yes ☐ no Did the camera flashlight bother you
- ☐ yes ☐ no Did the pupil dilating drops bother you

After form is completed, please enter into Excel spreadsheet or [illegible]

Dr. Michael D. Abramoff, MD, PhD
Department of Ophthalmology and Visual Sciences
PFP 11290C
University of Iowa Hospitals and Clinics
200 Hawkins, Iowa City, IA 52242
Tel (319) 384 5833, fax: (319) 353 7996

[redacted]

[redacted]

Patient questionnaire

BEFORE telediagnosis

Name [redacted]

Thank you for your willingness to participate in this research project.

You are about to undergo digital photographing and diagnosis over the internet by ophthalmologists who are retinal specialists at the University of Iowa. They will evaluate your photographs for signs of diabetic retinopathy, a complication of diabetes. This new technique is called 'telediagnosis'. Until telediagnosis was available, you could only be screened in an ophthalmologist's office with a dilated retinal exam.

Please give your best answer to the following questions

Circle where you expect the quality of the retinal exam to be better:

1 (circled)	2	3	4	5
Ophthalmologist's office			Telediagnosis	

Circle which retinal exam you prefer:

1 (circled)	2	3	4	5
Ophthalmologist's office			Telediagnosis	

- ☐ yes ☒ no Do you expect the camera flashlight to bother you
- ☐ yes ☒ no Do you expect the pupil dilating drops to bother you
- ☐ yes ☒ no In the past five years have you been examined by an ophthalmologist
- ☒ yes ☐ no Do you currently carry health insurance
- ☒ yes ☐ no In some cases the photographs can only be made if your pupils are dilated with a drop. Were you aware of this?

Patient questionnaire

AFTER telediagnosis results have been discussed

Name: [redacted]

Circle your satisfaction with digital photography of your retina

1	2	3	4 (circled)	5
Terrible				Very good

Circle where you think the retinal exam is better:

1 (circled)	2	3	4	5
Ophthalmologist's office				Telediagnosis

Circle which retinal exam you prefer now:

1	2	3	4 (circled)	5
Ophthalmologist's office				Telediagnosis

- ☐ yes ☒ no Did the camera flashlight bother you
- ☐ yes ☐ no Did the pupil dilating drops bother you

After form is completed, please enter into Excel spreadsheet or send to:

Dr. Michael D. Abramoff, MD, PhD
Department of Ophthalmology and Visual Sciences
PFP 11290C
University of Iowa Hospitals and Clinics
200 Hawkins, Iowa City, IA 52242
Tel (319) 384 5833, fax: (319) 353 7996

[redacted]

[redacted]

Patient questionnaire

BEFORE telediagnosis

[redacted]

Name [redacted]

Thank you for your willingness to participate in this research project.

You are about to undergo digital photographing and diagnosis over the internet by ophthalmologists who are retinal specialists at the University of Iowa. They will evaluate your photographs for signs of diabetic retinopathy, a complication of diabetes. This new technique is called 'telediagnosis'. Until telediagnosis was available, you could only be screened in an ophthalmologist's office with a dilated retinal exam.

Please give your best answer to the following questions

Circle where you expect the quality of the retinal exam to be better:

1	2	3	4	5
Ophthalmologist's office			Telediagnosis	

Circle which retinal exam you prefer:

1	2	3	4	5
Ophthalmologist's office			Telediagnosis	

- □ yes □ no Do you expect the camera flashlight to bother you
- □ yes □ no Do you expect the pupil dilating drops to bother you
- □ yes □ no In the past five years have you been examined by an ophthalmologist
- □ yes □ no Do you currently carry health insurance
- □ yes □ no In some cases the photographs can only be made if your pupils are dilated with a drop. Were you aware of this?

Patient questionnaire

AFTER telediagnosis results have been discussed

Nam[redacted]

Circle your satisfaction with digital photography of your retina

1	2	3	4	5
Terrible				Very good

Circle where you think the retinal exam is better:

1	2	3	4	5
Ophthalmologist's office				Telediagnosis

Circle which retinal exam you prefer now:

1	2	3	4	5
Ophthalmologist's office				Telediagnosis

- □ yes □ no Did the camera flashlight bother you
- □ yes □ no Did the pupil dilating drops bother you

After form is completed, please enter into Excel spreadsheet or [illegible]

Dr. Michael D. Abramoff, MD, PhD
Department of Ophthalmology and Visual Sciences
PFP 11290C
University of Iowa Hospitals and Clinics
200 Hawkins, Iowa City, IA 52242
Tel (319) 384 5833, fax: (319) 353 7996

[redacted]

[redacted]

Patient questionnaire

BEFORE telediagnosis

Na[redacted]

Thank you for your willingness to participate in this research project.

You are about to undergo digital photographing and diagnosis over the internet by ophthalmologists who are retinal specialists at the University of Iowa. They will evaluate your photographs for signs of diabetic retinopathy, a complication of diabetes. This new technique is called 'telediagnosis'. Until telediagnosis was available, you could only be screened in an ophthalmologist's office with a dilated retinal exam.

Please give your best answer to the following questions

Circle where you expect the quality of the retinal exam to be better:

1 Ophthalmologist's office — 2 — (3) — 4 — 5 Telediagnosis

Circle which retinal exam you prefer:

1 Ophthalmologist's office — 2 — (3) — 4 — 5 Telediagnosis

- ☐ yes ☑ no Do you expect the camera flashlight to bother you
- ☐ yes ☑ no Do you expect the pupil dilating drops to bother you
- ☐ yes ☑ no In the past five years have you been examined by an ophthalmologist
- ☑ yes ☐ no Do you currently carry health insurance
- ☑ yes ☐ no In some cases the photographs can only be made if your pupils are dilated with a drop. Were you aware of this?

[redacted]

Patient questionnaire

AFTER telediagnosis results have been discussed

Name:____________________________ **Date:**________________

Circle your satisfaction with digital photography of your retina

1 Terrible — 2 — 3 — 4 — 5 Very good

Circle where you think the retinal exam is better:

1 Ophthalmologist's office — 2 — 3 — 4 — 5 Telediagnosis

Circle which retinal exam you prefer now:

1 Ophthalmologist's office — 2 — 3 — 4 — 5 Telediagnosis

- ☐ yes ☐ no Did the camera flashlight bother you
- ☐ yes ☐ no Did the pupil dilating drops bother you

After form is completed, please enter into Excel spreadsheet or [illegible]

Dr. Michael D. Abramoff, MD, PhD
Department of Ophthalmology and Visual Sciences
PFP 11290C
University of Iowa Hospitals and Clinics
200 Hawkins, Iowa City, IA 52242
Tel (319) 384 5833, fax: (319) 353 7996

[redacted]

[redacted]

Patient questionnaire

BEFORE telediagnosis

N[redacted]

Thank you for your willingness to participate in this research project.

You are about to undergo digital photographing and diagnosis over the internet by ophthalmologists who are retinal specialists at the University of Iowa. They will evaluate your photographs for signs of diabetic retinopathy, a complication of diabetes. This new technique is called 'telediagnosis'. Until telediagnosis was available, you could only be screened in an ophthalmologist's office with a dilated retinal exam.

Please give your best answer to the following questions

Circle where you expect the quality of the retinal exam to be better:

1	2	3	4	5
Ophthalmologist's office			Telediagnosis	

Circle which retinal exam you prefer:

1	2	3	4	5
Ophthalmologist's office			Telediagnosis	

□ yes □ no Do you expect the camera flashlight to bother you
□ yes □ no Do you expect the pupil dilating drops to bother you
□ yes □ no In the past five years have you been examined by an ophthalmologist
□ yes □ no Do you currently carry health insurance
□ yes □ no In some cases the photographs can only be made if your pupils are dilated with a drop. Were you aware of this?

Patient questionnaire

AFTER telediagnosis results have been discussed

Name:______________________________ **Date:**______________

Circle your satisfaction with digital photography of your retina

1	2	3	4	5
Terrible				Very good

Circle where you think the retinal exam is better:

1	2	3	4	5
Ophthalmologist's office				Telediagnosis

Circle which retinal exam you prefer now:

1	2	3	4	5
Ophthalmologist's office				Telediagnosis

□ yes □ no Did the camera flashlight bother you
□ yes □ no Did the pupil dilating drops bother you

After form is completed, please enter into Excel spreadsheet or send to:

Dr. Michael D. Abramoff, MD, PhD
Department of Ophthalmology and Visual Sciences
PFP 11290C
University of Iowa Hospitals and Clinics
200 Hawkins, Iowa City, IA 52242
Tel (319) 384 5833, fax: (319) 353 7996

[redacted]

[redacted]

Patient questionnaire

BEFORE telediagnosis

[redacted]

Name: [redacted]

Thank you for your willingness to participate in this research project.

You are about to undergo digital photographing and diagnosis over the internet by ophthalmologists who are retinal specialists at the University of Iowa. They will evaluate your photographs for signs of diabetic retinopathy, a complication of diabetes. This new technique is called 'telediagnosis'. Until telediagnosis was available, you could only be screened in an ophthalmologist's office with a dilated retinal exam.

Please give your best answer to the following questions

Circle where you expect the quality of the retinal exam to be better:

1 (circled)	2	3	4	5
Ophthalmologist's office			Telediagnosis	

Circle which retinal exam you prefer:

1 (circled)	2	3	4	5
Ophthalmologist's office			Telediagnosis	

- ☐ yes ☒ no Do you expect the camera flashlight to bother you
- ☐ yes ☒ no Do you expect the pupil dilating drops to bother you
- ☐ yes ☒ no In the past five years have you been examined by an ophthalmologist
- ☐ yes ☒ no Do you currently carry health insurance
- ☒ yes ☐ no In some cases the photographs can only be made if your pupils are dilated with a drop. Were you aware of this?

Patient questionnaire

AFTER telediagnosis results have been discussed [redacted]

Name: [redacted]

Circle your satisfaction with digital photography of your retina

1	2	3	4	5 (circled)
Terrible				Very good

Circle where you think the retinal exam is better:

1	2	3	4	5 (circled)
Ophthalmologist's office				Telediagnosis

Circle which retinal exam you prefer now:

1	2	3	4	5 (circled)
Ophthalmologist's office				Telediagnosis

- ☐ yes ☒ no Did the camera flashlight bother you
- ☐ yes ☐ no /Did the pupil dilating drops bother you

[redacted]

After form is completed, please enter into Excel spreadsheet or [illegible]

Dr. Michael D. Abramoff, MD, PhD
Department of Ophthalmology and Visual Sciences
PFP 11290C
University of Iowa Hospitals and Clinics
200 Hawkins, Iowa City, IA 52242
Tel (319) 384 5833, fax: (319) 353 7996

[redacted]

[redacted]

Patient questionnaire

BEFORE telediagnosis

[redacted]

Name[redacted]

Thank you for your willingness to participate in this research project.
You are about to undergo digital photographing and diagnosis over the internet by ophthalmologists who are retinal specialists at the University of Iowa. They will evaluate your photographs for signs of diabetic retinopathy, a complication of diabetes. This new technique is called 'telediagnosis'. Until telediagnosis was available, you could only be screened in an ophthalmologist's office with a dilated retinal exam.

Please give your best answer to the following questions

Circle where you expect the quality of the retinal exam to be better:

1 2 (3) 4 5

Ophthalmologist's office — Telediagnosis

Circle which retinal exam you prefer:

1 2 (3) 4 5

Ophthalmologist's office — Telediagnosis

- ☐ yes ☒ no Do you expect the camera flashlight to bother you
- ☐ yes ☒ no Do you expect the pupil dilating drops to bother you
- ☒ yes ☐ no In the past five years have you been examined by an ophthalmologist
- ☒ yes ☐ no Do you currently carry health insurance
- ☒ yes ☐ no In some cases the photographs can only be made if your pupils are dilated with a drop. Were you aware of this?

Patient questionnaire

AFTER telediagnosis results have been discussed

Name[redacted]

Circle your satisfaction with digital photography of your retina

1 2 3 4 (5)

Terrible — Very good

Circle where you think the retinal exam is better:

1 2 3 4 (5)

Ophthalmologist's office — Telediagnosis

Circle which retinal exam you prefer now:

1 2 3 4 (5)

Ophthalmologist's office — Telediagnosis

- ☐ yes ☒ no Did the camera flashlight bother you
- ☐ yes ☐ no Did the pupil dilating drops bother you

After form is completed, please enter into Excel spreadsheet or send to:

Dr. Michael D. Abramoff, MD, PhD
Department of Ophthalmology and Visual Sciences
PFP 11290C
University of Iowa Hospitals and Clinics
200 Hawkins, Iowa City, IA 52242
Tel (319) 384 5833, fax: (319) 353 7996

[redacted]

[redacted]

Patient questionnaire

BEFORE telediagnosis

[redacted]

Name: [redacted]

Thank you for your willingness to participate in this research project.

You are about to undergo digital photographing and diagnosis over the internet by ophthalmologists who are retinal specialists at the University of Iowa. They will evaluate your photographs for signs of diabetic retinopathy, a complication of diabetes. This new technique is called 'telediagnosis'. Until telediagnosis was available, you could only be screened in an ophthalmologist's office with a dilated retinal exam.

Please give your best answer to the following questions

Circle where you expect the quality of the retinal exam to be better:

1	2	3	4	5
Ophthalmologist's office			Telediagnosis	

Circle which retinal exam you prefer:

1	2	3	4	5
Ophthalmologist's office			Telediagnosis	

- ☐ yes ☑ no Do you expect the camera flashlight to bother you
- ☑ yes ☐ no Do you expect the pupil dilating drops to bother you
- ☑ yes ☐ no In the past five years have you been examined by an ophthalmologist
- ☑ yes ☐ no Do you currently carry health insurance
- ☑ yes ☐ no In some cases the photographs can only be made if your pupils are dilated with a drop. Were you aware of this?

Patient questionnaire

AFTER telediagnosis results have been discussed

Name:______________________ **Date:**______________

Circle your satisfaction with digital photography of your retina

1	2	3	4	5
Terrible				Very good

Circle where you think the retinal exam is better:

1	2	3	4	5
Ophthalmologist's office				Telediagnosis

Circle which retinal exam you prefer now:

1	2	3	4	5
Ophthalmologist's office				Telediagnosis

- ☐ yes ☑ no Did the camera flashlight bother you
- ☑ yes ☐ no Did the pupil dilating drops bother you

After form is completed, please enter into Excel spreadsheet or send to:

Dr. Michael D. Abramoff, MD, PhD
Department of Ophthalmology and Visual Sciences
PFP 11290C
University of Iowa Hospitals and Clinics
200 Hawkins, Iowa City, IA 52242
Tel (319) 384 5833, fax: (319) 353 7996

[redacted]

[redacted]

[redacted]

Patient questionnaire
BEFORE telediagnosis

Name [redacted]

Thank you for your willingness to participate in this research project.
You are about to undergo digital photographing and diagnosis over the internet by ophthalmologists who are retinal specialists at the University of Iowa. They will evaluate your photographs for signs of diabetic retinopathy, a complication of diabetes. This new technique is called 'telediagnosis'. Until telediagnosis was available, you could only be screened in an ophthalmologist's office with a dilated retinal exam.

Please give your best answer to the following questions

Circle where you expect the quality of the retinal exam to be better:
1 Ophthalmologist's office — 2 — (3) — 4 Telediagnosis — 5

Circle which retinal exam you prefer:
1 Ophthalmologist's office — 2 — (3) — 4 Telediagnosis — 5

- ☐ yes ☑ no Do you expect the camera flashlight to bother you
- ☑ yes ☐ no Do you expect the pupil dilating drops to bother you
- ☑ yes ☐ no In the past five years have you been examined by an ophthalmologist
- ☑ yes ☐ no Do you currently carry health insurance
- ☑ yes ☐ no In some cases the photographs can only be made if your pupils are dilated with a drop. Were you aware of this?

Patient questionnaire
AFTER telediagnosis results have been discussed

Name:____________________ **Date:**__________

Circle your satisfaction with digital photography of your retina
1 Terrible — 2 — (3) — 4 — 5 Very good

Circle where you think the retinal exam is better:
1 Ophthalmologist's office — 2 — (3) — 4 — 5 Telediagnosis

Circle which retinal exam you prefer now:
1 Ophthalmologist's office — 2 — (3) — 4 — 5 Telediagnosis

- ☐ yes ☐ no Did the camera flashlight bother you
- ☐ yes ☐ no Did the pupil dilating drops bother you

After form is completed, please enter into Excel spreadsheet or send to:

Dr. Michael D. Abramoff, MD, PhD
Department of Ophthalmology and Visual Sciences
PFP 11290C
University of Iowa Hospitals and Clinics
200 Hawkins, Iowa City, IA 52242
Tel (319) 384 5833, fax: (319) 353 7996

[redacted]

[redacted]

Patient questionnaire

BEFORE telediagnosis [redacted]

Name:[redacted]

Thank you for your willingness to participate in this research project.

You are about to undergo digital photographing and diagnosis over the internet by ophthalmologists who are retinal specialists at the University of Iowa. They will evaluate your photographs for signs of diabetic retinopathy, a complication of diabetes. This new technique is called 'telediagnosis'. Until telediagnosis was available, you could only be screened in an ophthalmologist's office with a dilated retinal exam.

Please give your best answer to the following questions

Circle where you expect the quality of the retinal exam to be better:

1	2	3	4	5
Ophthalmologist's office			Telediagnosis	

Circle which retinal exam you prefer:

1	2	3	4	5
Ophthalmologist's office			Telediagnosis	

- ☐ yes ☒ no Do you expect the camera flashlight to bother you
- ☐ yes ☒ no Do you expect the pupil dilating drops to bother you
- ☒ yes ☐ no In the past five years have you been examined by an ophthalmologist
- ☒ yes ☐ no Do you currently carry health insurance
- ☐ yes ☒ no In some cases the photographs can only be made if your pupils are dilated with a drop. Were you aware of this?

Patient questionnaire

AFTER telediagnosis results have been discussed

Name:________________________ **Date:**______________

Circle your satisfaction with digital photography of your retina

1	2	3	4	5
Terrible				Very good

Circle where you think the retinal exam is better:

1	2	3	4	5
Ophthalmologist's office				Telediagnosis

Circle which retinal exam you prefer now:

1	2	3	4	5
Ophthalmologist's office				Telediagnosis

- ☐ yes ☒ no Did the camera flashlight bother you
- ☐ yes ☒ no Did the pupil dilating drops bother you

After form is completed, please enter into Excel spreadsheet or send to:

Dr. Michael D. Abramoff, MD, PhD
Department of Ophthalmology and Visual Sciences
PFP 11290C
University of Iowa Hospitals and Clinics
200 Hawkins, Iowa City, IA 52242
Tel (319) 384 5833, fax: (319) 353 7996

[redacted]

[redacted]

Patient questionnaire

BEFORE telediagnosis

[redacted]

Name: [redacted]

Thank you for your willingness to participate in this research project.

You are about to undergo digital photographing and diagnosis over the internet by ophthalmologists who are retinal specialists at the University of Iowa. They will evaluate your photographs for signs of diabetic retinopathy, a complication of diabetes. This new technique is called 'telediagnosis'. Until telediagnosis was available, you could only be screened in an ophthalmologist's office with a dilated retinal exam.

Please give your best answer to the following questions

Circle where you expect the quality of the retinal exam to be better:

1 Ophthalmologist's office — 2 — (3) — 4 — 5 Telediagnosis

Circle which retinal exam you prefer:

1 Ophthalmologist's office — 2 — (3) — 4 — 5 Telediagnosis

- ☐ yes ☒ no — Do you expect the camera flashlight to bother you
- ☐ yes ☒ no — Do you expect the pupil dilating drops to bother you
- ☒ yes ☐ no — In the past five years have you been examined by an ophthalmologist [redacted]
- ☒ yes ☐ no — Do you currently carry health insurance [redacted]
- ☒ yes ☐ no — In some cases the photographs can only be made if your pupils are dilated with a drop. Were you aware of this?

Patient questionnaire

AFTER telediagnosis results have been discussed

Name:__________________________ Date:____________

Circle your satisfaction with digital photography of your retina

1 Terrible — 2 — 3 — 4 — (5 Very good)

Circle where you think the retinal exam is better:

1 Ophthalmologist's office — 2 — 3 — 4 — (5 Telediagnosis)

Circle which retinal exam you prefer now:

1 Ophthalmologist's office — 2 — 3 — 4 — (5 Telediagnosis)

- ☐ yes ☒ no — Did the camera flashlight bother you
- ☐ yes ☒ no — Did the pupil dilating drops bother you

After form is completed, please enter into Excel spreadsheet or send to:

Dr. Michael D. Abramoff, MD, PhD
Department of Ophthalmology and Visual Sciences
PFP 11290C
University of Iowa Hospitals and Clinics
200 Hawkins, Iowa City, IA 52242
Tel (319) 384 5833, fax: (319) 353 7996

[redacted]

[redacted]

[redacted]

Patient questionnaire

BEFORE telediagnosis

Name: [redacted]

Thank you for your willingness to participate in this research project.
You are about to undergo digital photographing and diagnosis over the internet by ophthalmologists who are retinal specialists at the University of Iowa. They will evaluate your photographs for signs of diabetic retinopathy, a complication of diabetes. This new technique is called 'telediagnosis'. Until telediagnosis was available, you could only be screened in an ophthalmologist's office with a dilated retinal exam.

Please give your best answer to the following questions

Circle where you expect the quality of the retinal exam to be better:

1	2	3	4	5
Ophthalmologist's office		(circled)	Telediagnosis	

Circle which retinal exam you prefer:

1	2	3	4	5
Ophthalmologist's office		(circled)	Telediagnosis	

- ☐ yes ☒ no — Do you expect the camera flashlight to bother you
- ☐ yes ☒ no — Do you expect the pupil dilating drops to bother you
- ☐ yes ☒ no — In the past five years have you been examined by an ophthalmologist
- ☒ yes ☐ no — Do you currently carry health insurance
- ☐ yes ☒ no — In some cases the photographs can only be made if your pupils are dilated with a drop. Were you aware of this?

Patient questionnaire

AFTER telediagnosis results have been discussed

Name:______________________ Date:______________

Circle your satisfaction with digital photography of your retina

1	2	3	4	5
Terrible		(circled)		Very good

Circle where you think the retinal exam is better:

1	2	3	4	5
Ophthalmologist's office		(circled)		Telediagnosis

Circle which retinal exam you prefer now:

1	2	3	4	5
Ophthalmologist's office		(circled)		Telediagnosis

- ☐ yes ☒ no — Did the camera flashlight bother you
- ☐ yes ☐ no — Did the pupil dil[redacted]

After form is completed, please enter into Excel spreadsheet or [illegible]

Dr. Michael D. Abramoff, MD, PhD
Department of Ophthalmology and Visual Sciences
PFP 11290C
University of Iowa Hospitals and Clinics
200 Hawkins, Iowa City, IA 52242
Tel (319) 384 5833, fax: (319) 353 7996

[redacted]

[redacted]

Patient questionnaire

BEFORE telediagnosis

Name: [redacted]

Thank you for your willingness to participate in this research project.
You are about to undergo digital photographing and diagnosis over the internet by ophthalmologists who are retinal specialists at the University of Iowa. They will evaluate your photographs for signs of diabetic retinopathy, a complication of diabetes. This new technique is called 'telediagnosis'. Until telediagnosis was available, you could only be screened in an ophthalmologist's office with a dilated retinal exam.

Please give your best answer to the following questions

Circle where you expect the quality of the retinal exam to be better:

1	2	(3)	4	5
Ophthalmologist's office			Telediagnosis	

Circle which retinal exam you prefer:

1	2	(3)	4	5
Ophthalmologist's office			Telediagnosis	

- ☒ yes ☐ no — Do you expect the camera flashlight to bother you
- ☒ yes ☐ no — Do you expect the pupil dilating drops to bother you
- ☒ yes ☐ no — In the past five years have you been examined by an ophthalmologist
- ☒ yes ☐ no — Do you currently carry health insurance
- ☒ yes ☐ no — In some cases the photographs can only be made if your pupils are dilated with a drop. Were you aware of this?

Patient questionnaire

AFTER telediagnosis results have been discussed

Name:__________________________ **Date:**______________

Circle your satisfaction with digital photography of your retina

1	2	3	(4)	5
Terrible				Very good

Circle where you think the retinal exam is better:

1	2	(3)	4	5
Ophthalmologist's office				Telediagnosis

Circle which retinal exam you prefer now:

1	2	(3)	4	5
Ophthalmologist's office				Telediagnosis

- ☒ yes ☐ no — Did the camera flashlight bother you
- ☒ yes ☐ no — Did the pupil dilating drops bother you

After form is completed, please enter into Excel spreadsheet or [illegible]

Dr. Michael D. Abramoff, MD, PhD
Department of Ophthalmology and Visual Sciences
PFP 11290C
University of Iowa Hospitals and Clinics
200 Hawkins, Iowa City, IA 52242
Tel (319) 384 5833, fax: (319) 353 7996

[redacted]

[redacted]

Patient questionnaire

[redacted]

Name: [redacted]

Thank you for your willingness to participate in this research project.

You are about to undergo digital photographing and diagnosis over the internet by ophthalmologists who are retinal specialists at the University of Iowa. They will evaluate your photographs for signs of diabetic retinopathy, a complication of diabetes. This new technique is called 'telediagnosis'. Until telediagnosis was available, you could only be screened in an ophthalmologist's office with a dilated retinal exam.

Please give your best answer to the following questions

Circle where you expect the quality of the retinal exam to be better:

1 Ophthalmologist's office — 2 — 3 — 4 Telediagnosis — (5)

Circle which retinal exam you prefer:

1 Ophthalmologist's office — 2 — 3 — 4 Telediagnosis — (5)

- ☐ yes ☒ no Do you expect the camera flashlight to bother you
- ☐ yes ☒ no Do you expect the pupil dilating drops to bother you
- ☐ yes ☒ no In the past five years have you been examined by an ophthalmologist
- ☒ yes ☐ no Do you currently carry health insurance
- ☐ yes ☒ no In some cases the photographs can only be made if your pupils are dilated with a drop. Were you aware of this?

Patient questionnaire

AFTER telediagnosis results have been discussed

Name:______________________________ **Date:**______________

Circle your satisfaction with digital photography of your retina

1 Terrible — 2 — 3 — 4 — (5) Very good

Circle where you think the retinal exam is better:

1 Ophthalmologist's office — 2 — 3 — 4 — (5) Telediagnosis

Circle which retinal exam you prefer now:

1 Ophthalmologist's office — 2 — 3 — 4 — (5) Telediagnosis

- ☐ yes ☒ no Did the camera flashlight bother you
- ☐ yes ☒ no Did the pupil dilating drops bother you

After form is completed, please enter into Excel spreadsheet or send to:

Dr. Michael D. Abramoff, MD, PhD
Department of Ophthalmology and Visual Sciences
PFP 11290C
University of Iowa Hospitals and Clinics
200 Hawkins, Iowa City, IA 52242
Tel (319) 384 5833, fax: (319) 353 7996

[redacted]

[redacted]

Patient questionnaire

BEFORE telediagnosis

Name: [redacted]

Thank you for your willingness to participate in this research project.
You are about to undergo digital photographing and diagnosis over the internet by ophthalmologists who are retinal specialists at the University of Iowa. They will evaluate your photographs for signs of diabetic retinopathy, a complication of diabetes. This new technique is called 'telediagnosis'. Until telediagnosis was available, you could only be screened in an ophthalmologist's office with a dilated retinal exam.

Please give your best answer to the following questions

Circle where you expect the quality of the retinal exam to be better:

1	2	3	4	5
Ophthalmologist's office			Telediagnosis	

Circle which retinal exam you prefer:

1	2	3	4	5
Ophthalmologist's office			Telediagnosis	

- ☐ yes ☐ no Do you expect the camera flashlight to bother you
- ☐ yes ☐ no Do you expect the pupil dilating drops to bother you
- ☐ yes ☐ no In the past five years have you been examined by an ophthalmologist
- ☐ yes ☐ no Do you currently carry health insurance
- ☐ yes ☐ no In some cases the photographs can only be made if your pupils are dilated with a drop. Were you aware of this?

Patient questionnaire

AFTER telediagnosis results have been discussed

Name:________________________________ **Date:**________________

Circle your satisfaction with digital photography of your retina

1	2	3	4	5
Terrible				Very good

Circle where you think the retinal exam is better:

1	2	3	4	5
Ophthalmologist's office				Telediagnosis

Circle which retinal exam you prefer now:

1	2	3	4	5
Ophthalmologist's office				Telediagnosis

- ☐ yes ☐ no Did the camera flashlight bother you
- ☐ yes ☐ no Did the pupil dilating drops bother you

[redacted]

After form is completed, please enter into Excel spreadsheet or send to:

Dr. Michael D. Abramoff, MD, PhD
Department of Ophthalmology and Visual Sciences
PFP 11290C
University of Iowa Hospitals and Clinics
200 Hawkins, Iowa City, IA 52242
Tel (319) 384 5833, fax: (319) 353 7996

[redacted]

[redacted]

Patient questionnaire

BEFORE telediagnosis

Name: [redacted]

Thank you for your willingness to participate in this research project.

You are about to undergo digital photographing and diagnosis over the internet by ophthalmologists who are retinal specialists at the University of Iowa. They will evaluate your photographs for signs of diabetic retinopathy, a complication of diabetes. This new technique is called 'telediagnosis'. Until telediagnosis was available, you could only be screened in an ophthalmologist's office with a dilated retinal exam.

Please give your best answer to the following questions

Circle where you expect the quality of the retinal exam to be better:

1	2	3	4	5
Ophthalmologist's office			Telediagnosis	

Circle which retinal exam you prefer:

1	2	3	4	5
Ophthalmologist's office			Telediagnosis	

☐ yes ☒ no Do you expect the camera flashlight to bother you
☐ yes ☒ no Do you expect the pupil dilating drops to bother you
☐ yes ☒ no In the past five years have you been examined by an ophthalmologist
☒ yes ☐ no Do you currently carry health insurance
☐ yes ☒ no In some cases the photographs can only be made if your pupils are dilated with a drop. Were you aware of this?

Patient questionnaire

[redacted]

Name: [redacted]

Circle your satisfaction with digital photography of your retina

1	2	3	4	5
Terrible				Very good

Circle where you think the retinal exam is better:

1	2	3	4	5
Ophthalmologist's office				Telediagnosis

Circle which retinal exam you prefer now:

1	2	3	4	5
Ophthalmologist's office				Telediagnosis

☐ yes ☒ no Did the camera flashlight bother you
☐ yes ☒ no Did the pupil dilating drops bother you

After form is completed, please enter into Excel spreadsheet [illegible]

Dr. Michael D. Abramoff, MD, PhD
Department of Ophthalmology and Visual Sciences
PFP 11290C
University of Iowa Hospitals and Clinics
200 Hawkins, Iowa City, IA 52242
Tel (319) 384 5833, fax: (319) 353 7996

[redacted]

[redacted]

Patient questionnaire

BEFORE telediagnosis

Name: [redacted]

Thank you for your willingness to participate in this research project.

You are about to undergo digital photographing and diagnosis over the internet by ophthalmologists who are retinal specialists at the University of Iowa. They will evaluate your photographs for signs of diabetic retinopathy, a complication of diabetes. This new technique is called 'telediagnosis'. Until telediagnosis was available, you could only be screened in an ophthalmologist's office with a dilated retinal exam.

Please give your best answer to the following questions

Circle where you expect the quality of the retinal exam to be better:

1 Ophthalmologist's office — 2 — 3 — 4 Telediagnosis — 5

Circle which retinal exam you prefer:

1 Ophthalmologist's office — 2 — 3 — 4 Telediagnosis — 5

- □ yes □ no Do you expect the camera flashlight to bother you
- □ yes □ no Do you expect the pupil dilating drops to bother you
- □ yes □ no In the past five years have you been examined by an ophthalmologist
- □ yes □ no Do you currently carry health insurance
- □ yes □ no In some cases the photographs can only be made if your pupils are dilated with a drop. Were you aware of this?

Patient questionnaire

AFTER telediagnosis results have been discussed

Name:____________________ **Date:**__________

Circle your satisfaction with digital photography of your retina

1 Terrible — 2 — 3 — 4 — 5 Very good

Circle where you think the retinal exam is better:

1 Ophthalmologist's office — 2 — 3 — 4 — 5 Telediagnosis

Circle which retinal exam you prefer now:

1 Ophthalmologist's office — 2 — 3 — 4 — 5 Telediagnosis

- □ yes □ no Did the camera flashlight bother you
- □ yes □ no Did the pupil dilating drops bother you [redacted]

After form is completed, please enter into Excel spreadsheet or send to:

Dr. Michael D. Abramoff, MD, PhD
Department of Ophthalmology and Visual Sciences
PFP 11290C
University of Iowa Hospitals and Clinics
200 Hawkins, Iowa City, IA 52242
Tel (319) 384 5833, fax: (319) 353 7996

[redacted]

Patient Questionnaire

[redacted]

Name [redacted]

BEFORE telediagnosis

Thank you for your willingness to participate in this research project.

You are about to undergo digital photographing and diagnosis over the internet by ophthalmolog sts who are retinal specialists at the University of Iowa. They will evaluate your photographs for signs of diabetic retinopathy, a complication of diabetes. This new technique is called 'telediagnosis'. Until telediagnosis was available, you could only be screened in an ophthalmolog st's office with a dilated retinal exam. Please give your best answer.

What number best expresses where you think the *quality* of the retinal exam is best:

1	2	3	4	5
(Better in ophthalmolog st's office)		undecided		(Better through Telediagnosis)

What number best expresses *where* you prefer to have the retinal exam:

1	2	3	4	5
(Prefer ophthalmologist' s office)		undecided		(Prefer family physician)

☐ yes ☐ no Do you expect the camera flashlight to bother you?

☑ yes ☐ no Do you expect the pupil dilating drops to bother you?

☑ yes ☐ no In the past five years have you been examined by an ophthalmologist?

☑ yes ☐ no Do you currently carry health insurance?

☑ yes ☐ no Sometimes photographs can only be made if your pupils are made larger with a drop. Were you aware of this?

AFTER telediagnosis

What number best expresses your *satisfaction* with telediagnosis:

1	2	3	4	5
(Terrible)		undecided		(Very good)

What number best expresses your where you think the *quality* of the retinal exam is best::

1	2	3	4	5
(Better in ophthalmolog st's office)		undecided		(Better through Telediagnosis)

What number best expresses *where* you prefer to have the retinal exam <u>now</u>:

1	2	3	4	5
(Prefer ophthalmologist' s office)		undecided		(Prefer family physician)

☐ yes ☑ no Did the camera flashlight bother you?

☐ yes ☐ no Did the pupil dilating drops bother you? ☑ not applicable

[redacted]

[redacted]onnaire

Name [redacted] Date [redacted]

BEFORE telediagnosis

Thank you for your willingness to participate in this research project.

You are about to undergo digital photographing and diagnosis over the internet by ophthalmologi sts who are retinal specialists at the University of Iowa. They will evaluate your photographs for signs of diabetic retinopathy, a complication of diabetes. This new technique is called 'telediagnosis'. Until telediagnosis was available, you could only be screened in an ophthalmologi st's office with a dilated retinal exam. Please give your best answer.

What number best expresses where you think the *quality* of the retinal exam is best:

1	2	3	4	5
(Better in ophthalmologi st's office)		undecided		(Better through Telediagnosis)

What number best expresses *where* you prefer to have the retinal exam:

1	2	3	4	5
(Prefer ophthalmologist' s office)		undecided		(Prefer family physician)

☐ yes ☑ no Do you expect the camera flashlight to bother you?

☐ yes ☑ no Do you expect the pupil dilating drops to bother you?

☐ yes ☑ no In the past five years have you been examined by an ophthalmologist?

☑ yes ☐ no Do you currently carry health insurance?

☑ yes ☐ no Sometimes photographs can only be made if your pupils are made larger with a drop. Were you aware of this?

AFTER telediagnosis

What number best expresses your *satisfaction* with telediagnosis:

1	2	3	4	5
(Terrible)		undecided		(Very good)

What number best expresses your where you think the *quality* of the retinal exam is best::

1	2	3	4	5
(Better in ophthalmolog st's office)		undecided		(Better through Telediagnosis)

What number best expresses *where* you prefer to have the retinal exam <u>now</u>:

1	2	3	4	5
(Prefer ophthalmologist' s office)		undecided		(Prefer family physician)

☐ yes ☑ no Did the camera flashlight bother you?

☐ yes ☐ no Did the pupil dilating drops bother you? ☑ not applicable

[redacted]

[redacted]

Patient questionnaire

[redacted]

Name: [redacted]

Thank you for your willingness to participate in this research project.
You are about to undergo digital photographing and diagnosis over the internet by ophthalmologists who are retinal specialists at the University of Iowa. They will evaluate your photographs for signs of diabetic retinopathy, a complication of diabetes. This new technique is called 'telediagnosis'. Until telediagnosis was available, you could only be screened in an ophthalmologist's office with a dilated retinal exam.

Please give your best answer to the following questions

Circle where you expect the quality of the retinal exam to be better:

1 Ophthalmologist's office — 2 — 3 *(circled)* — 4 Telediagnosis — 5

Circle which retinal exam you prefer:

1 Ophthalmologist's office — 2 — 3 — 4 *(circled)* Telediagnosis — 5

- ☐ yes ☑ no — Do you expect the camera flashlight to bother you
- ☐ yes ☑ no — Do you expect the pupil dilating drops to bother you
- ☐ yes ☒ no — In the past five years have you been examined by an ophthalmologist
- ☒ yes ☐ no — Do you currently carry health insurance
- ☒ yes ☐ no — In some cases the photographs can only be made if your pupils are dilated with a drop. Were you aware of this?

Patient questionnaire

AFTER telediagnosis results have been discussed

Name:______________________________ **Date:**______________

Circle your satisfaction with digital photography of your retina

1 Terrible — 2 — 3 — 4 — 5 *(circled)* Very good

Circle where you think the retinal exam is better:

1 Ophthalmologist's office — 2 — 3 — 4 — 5 *(circled)* Telediagnosis

Circle which retinal exam you prefer now:

1 Ophthalmologist's office — 2 — 3 — 4 — 5 *(circled)* Telediagnosis

- ☐ yes ☑ no *(checked)* — Did the camera flashlight bother you
- ☐ yes ☐ no *(checked)* — Did the pupil dilating drops bother you

After form is completed, please enter into Excel spreadsheet or send to:
Dr. Michael D. Abramoff, MD, PhD
Department of Ophthalmology and Visual Sciences
PFP 11290C
University of Iowa Hospitals and Clinics
200 Hawkins, Iowa City, IA 52242
Tel (319) 384 5833, fax: (319) 353 7996

[redacted]

[redacted]

[redacted]

[redacted]

Patient Questionnaire

Name [redacted] Date [redacted]

BEFORE telediagnosis

Thank you for your willingness to participate in this research project.

You are about to undergo digital photographing and diagnosis over the internet by ophthalmologists who are retinal specialists at the University of Iowa. They will evaluate your photographs for signs of diabetic retinopathy, a complication of diabetes. This new technique is called 'telediagnosis'. Until telediagnosis was available, you could only be screened in an ophthalmologist's office with a dilated retinal exam. Please give your best answer.

What number best expresses where you think the *quality* of the retinal exam is best:

1	2	3	4	5
(Better in ophthalmologist's office)		undecided		(Better through Telediagnosis)

(3 circled)

What number best expresses *where* you prefer to have the retinal exam:

1	2	3	4	5
(Prefer ophthalmologist's office)		undecided		(Prefer family physician)

(3 circled)

☐ yes ☒ no **Do you expect the camera flashlight to bother you?**

☒ yes ☐ no **Do you expect the pupil dilating drops to bother you?**

☒ yes ☐ no **In the past five years have you been examined by an ophthalmologist?**

☒ yes ☐ no **Do you currently carry health insurance?**

☒ yes ☐ no **Sometimes photographs can only be made if your pupils are made larger with a drop. Were you aware of this?**

AFTER telediagnosis

What number best expresses your *satisfaction* with telediagnosis:

1	2	3	4	5
(Terrible)		undecided		(Very good)

(3 circled)

What number best expresses your where you think the *quality* of the retinal exam is best::

1	2	3	4	5
(Better in ophthalmologist's office)		undecided		(Better through Telediagnosis)

(3 circled)

What number best expresses *where* you prefer to have the retinal exam <u>now</u>:

1	2	3	4	5
(Prefer ophthalmologist's office)		undecided		(Prefer family physician)

(3 circled)

☐ yes ☒ no **Did the camera flashlight bother you?**

☐ yes ☐ no **Did the pupil dilating drops bother you?** ☒ not applicable

[redacted]

[redacted]

Patient Questionnaire

Name [redacted] Date [redacted]

BEFORE telediagnosis

Thank you for your willingness to participate in this research project.

You are about to undergo digital photographing and diagnosis over the internet by ophthalmologists who are retinal specialists at the University of Iowa. They will evaluate your photographs for signs of diabetic retinopathy, a complication of diabetes. This new technique is called 'telediagnosis'. Until telediagnosis was available, you could only be screened in an ophthalmologist's office with a dilated retinal exam. Please give your best answer.

What number best expresses where you think the *quality* of the retinal exam is best:

1<br>(Better in ophthalmologist's office)	2	3<br>undecided	4	5<br>(Better through Telediagnosis)

What number best expresses *where* you prefer to have the retinal exam:

1<br>(Prefer ophthalmologist's office)	2	3<br>undecided	4	5<br>(Prefer family physician)

☐ yes ☐ no **Do you expect the camera flashlight to bother you?**

☐ yes ☐ no **Do you expect the pupil dilating drops to bother you?**

☐ yes ☐ no **In the past five years have you been examined by an ophthalmologist?**

☐ yes ☐ no **Do you currently carry health insurance?**

☐ yes ☐ no **Sometimes photographs can only be made if your pupils are made larger with a drop. Were you aware of this?**

AFTER telediagnosis

What number best expresses your *satisfaction* with telediagnosis:

1<br>(Terrible)	2	3<br>undecided	4	5<br>(Very good)

What number best expresses your where you think the *quality* of the retinal exam is best::

1<br>(Better in ophthalmologist's office)	2	3<br>undecided	4	5<br>(Better through Telediagnosis)

What number best expresses *where* you prefer to have the retinal exam now:

1<br>(Prefer ophthalmologist's office)	2	3<br>undecided	4	5<br>(Prefer family physician)

☐ yes ☐ no **Did the camera flashlight bother you?**

☐ yes ☐ no **Did the pupil dilating drops bother you?** ☐ not applicable

[redacted]

[redacted]

Patient Questionnaire

Name [redacted] Date [redacted]

BEFORE telediagnosis

Thank you for your willingness to participate in this research project.

You are about to undergo digital photographing and diagnosis over the internet by ophthalmologists who are retinal specialists at the University of Iowa. They will evaluate your photographs for signs of diabetic retinopathy, a complication of diabetes. This new technique is called 'telediagnosis'. Until telediagnosis was available, you could only be screened in an ophthalmologist's office with a dilated retinal exam. Please give your best answer.

What number best expresses where you think the *quality* of the retinal exam is best:

1	2	3	4	5
(Better in ophthalmologist's office)		undecided		(Better through Telediagnosis)

What number best expresses *where* you prefer to have the retinal exam:

1	2	3	4	5
(Prefer ophthalmologist's office)		undecided		(Prefer family physician)

☐ yes ☑ no **Do you expect the camera flashlight to bother you?**

☐ yes ☑ no **Do you expect the pupil dilating drops to bother you?**

☐ yes ☑ no **In the past five years have you been examined by an ophthalmologist?**

☑ yes ☐ no **Do you currently carry health insurance?**

☑ yes ☐ no **Sometimes photographs can only be made if your pupils are made larger with a drop. Were you aware of this?**

AFTER telediagnosis

What number best expresses your *satisfaction* with telediagnosis:

1	2	3	4	5
(Terrible)		undecided		(Very good)

What number best expresses your where you think the *quality* of the retinal exam is best::

1	2	3	4	5
(Better in ophthalmologist's office)		undecided		(Better through Telediagnosis)

What number best expresses *where* you prefer to have the retinal exam <u>now</u>:

1	2	3	4	5
(Prefer ophthalmologist's office)		undecided		(Prefer family physician)

☐ yes ☑ no **Did the camera flashlight bother you?**

☐ yes ☐ no **Did the pupil dilating drops bother you?** ☑ not applicable

[redacted]

[redacted]

[redacted]estionnaire

Name [redacted] Date [redacted]

BEFORE telediagnosis

Thank you for your willingness to participate in this research project.

You are about to undergo digital photographing and diagnosis over the internet by ophthalmologists who are retinal specialists at the University of Iowa. They will evaluate your photographs for signs of diabetic retinopathy, a complication of diabetes. This new technique is called 'telediagnosis'. Until telediagnosis was available, you could only be screened in an ophthalmologist's office with a dilated retinal exam. Please give your best answer.

What number best expresses where you think the *quality* of the retinal exam is best:

1	2	3	4	5
(Better in ophthalmologist's office)		undecided		(Better through Telediagnosis)

What number best expresses *where* you prefer to have the retinal exam:

1	2	3	4	5
(Prefer ophthalmologist's office)		undecided		(Prefer family physician)

☐ yes ☑ no **Do you expect the camera flashlight to bother you?**

☑ yes ☐ no **Do you expect the pupil dilating drops to bother you?**

☑ yes ☐ no **In the past five years have you been examined by an ophthalmologist?**

☑ yes ☐ no **Do you currently carry health insurance?**

☑ yes ☐ no **Sometimes photographs can only be made if your pupils are made larger with a drop. Were you aware of this?**

AFTER telediagnosis

What number best expresses your *satisfaction* with telediagnosis:

1	2	3	4	5
(Terrible)		undecided		(Very good)

What number best expresses your where you think the *quality* of the retinal exam is best::

1	2	3	4	5
(Better in ophthalmologist's office)		undecided		(Better through Telediagnosis)

What number best expresses *where* you prefer to have the retinal exam <u>now</u>:

1	2	3	4	5
(Prefer ophthalmologist's office)		undecided		(Prefer family physician)

☐ yes ☑ no **Did the camera flashlight bother you?**

☐ yes ☐ no **Did the pupil dilating drops bother you?** ☑ not applicable

[redacted]

[redacted]

Patient questionnaire

BEFORE telediagnosis

Nam[redacted]

Thank you for your willingness to participate in this research project.

You are about to undergo digital photographing and diagnosis over the internet by ophthalmologists who are retinal specialists at the University of Iowa. They will evaluate your photographs for signs of diabetic retinopathy, a complication of diabetes. This new technique is called 'telediagnosis'. Until telediagnosis was available, you could only be screened in an ophthalmologist's office with a dilated retinal exam.

Please give your best answer to the following questions

Circle where you expect the quality of the retinal exam to be better:

(1) 2 3 4 5

Ophthalmologist's office — Telediagnosis

Circle which retinal exam you prefer:

1 2 3 (4) 5

Ophthalmologist's office — Telediagnosis

□ yes ☒ no Do you expect the camera flashlight to bother you

□ yes ☒ no Do you expect the pupil dilating drops to bother you

☒ yes □ no In the past five years have you been examined by an ophthalmologist

☒ yes □ no Do you currently carry health insurance

☒ yes □ no In some cases the photographs can only be made if your pupils are dilated with a drop. Were you aware of this?

Patient questionnaire

AFTER telediagnosis results have been discussed

Name:______________________________ **Date:**_______________

Circle your satisfaction with digital photography of your retina

1 2 3 4 (5)

Terrible — Very good

Circle where you think the retinal exam is better:

(1) 2 3 4 5

Ophthalmologist's office — Telediagnosis

Circle which retinal exam you prefer now:

1 2 3 4 (5)

Ophthalmologist's office — Telediagnosis

□ yes ☒ no Did the camera flashlight bother you

□ yes ☒ no Did the pupil dilating drops bother you

After form is completed, please enter into Excel spreadsheet or send to:

Dr. Michael D. Abramoff, MD, PhD
Department of Ophthalmology and Visual Sciences
PFP 11290C
University of Iowa Hospitals and Clinics
200 Hawkins, Iowa City, IA 52242
Tel (319) 384 5833, fax: (319) 353 7996

[redacted]

[redacted]

Patient Questionnaire

Name [redacted] Date [redacted]

BEFORE telediagnosis

Thank you for your willingness to participate in this research project.

You are about to undergo digital photographing and diagnosis over the internet by ophthalmologists who are retinal specialists at the University of Iowa. They will evaluate your photographs for signs of diabetic retinopathy, a complication of diabetes. This new technique is called 'telediagnosis'. Until telediagnosis was available, you could only be screened in an ophthalmologist's office with a dilated retinal exam. Please give your best answer.

What number best expresses where you think the *quality* of the retinal exam is best:

1<br>(Better in ophthalmologist's office)	2	**3**<br>undecided	4	5<br>(Better through Telediagnosis)

What number best expresses *where* you prefer to have the retinal exam:

1<br>(Prefer ophthalmologist's office)	2	**3**<br>undecided	4	5<br>(Prefer family physician)

☐ yes ☑ no **Do you expect the camera flashlight to bother you?**

☐ yes ☑ no **Do you expect the pupil dilating drops to bother you?**

☑ yes ☐ no **In the past five years have you been examined by an ophthalmologist?**

☑ yes ☐ no **Do you currently carry health insurance?**

☑ yes ☐ no **Sometimes photographs can only be made if your pupils are made larger with a drop. Were you aware of this?**

AFTER telediagnosis

What number best expresses your *satisfaction* with telediagnosis:

1<br>(Terrible)	2	3<br>undecided	**4**	5<br>(Very good)

What number best expresses your where you think the *quality* of the retinal exam is best::

1<br>(Better in ophthalmologist's office)	2	3<br>undecided	**4**	5<br>(Better through Telediagnosis)

What number best expresses *where* you prefer to have the retinal exam now:

1<br>(Prefer ophthalmologist's office)	2	3<br>undecided	**4**	5<br>(Prefer family physician)

☐ yes ☑ no **Did the camera flashlight bother you?**

☐ yes ☐ no **Did the pupil dilating drops bother you?** ☑ not applicable

[redacted]

[redacted]

Patient Questionnaire

Name [redacted] Date [redacted]

BEFORE telediagnosis

Thank you for your willingness to participate in this research project.

You are about to undergo digital photographing and diagnosis over the internet by ophthalmologists who are retinal specialists at the University of Iowa. They will evaluate your photographs for signs of diabetic retinopathy, a complication of diabetes. This new technique is called 'telediagnosis'. Until telediagnosis was available, you could only be screened in an ophthalmologist's office with a dilated retinal exam. Please give your best answer.

What number best expresses where you think the *quality* of the retinal exam is best:

1	2	3	4	5
(Better in ophthalmologist's office)		undecided		(Better through Telediagnosis)

What number best expresses *where* you prefer to have the retinal exam:

1	2	3	4	5
(Prefer ophthalmologist's office)		undecided		(Prefer family physician)

☐ yes ☒ no **Do you expect the camera flashlight to bother you?**

☐ yes ☒ no **Do you expect the pupil dilating drops to bother you?**

☐ yes ☒ no **In the past five years have you been examined by an ophthalmologist?**

☒ yes ☐ no **Do you currently carry health insurance?**

☐ yes ☒ no **Sometimes photographs can only be made if your pupils are made larger with a drop. Were you aware of this?**

AFTER telediagnosis

What number best expresses your *satisfaction* with telediagnosis:

1	2	3	4	5
(Terrible)		undecided		(Very good)

What number best expresses your where you think the *quality* of the retinal exam is best::

1	2	3	4	5
(Better in ophthalmologist's office)		undecided		(Better through Telediagnosis)

What number best expresses *where* you prefer to have the retinal exam now:

1	2	3	4	5
(Prefer ophthalmologist's office)		undecided		(Prefer family physician)

☐ yes ☐ no **Did the camera flashlight bother you?**

☐ yes ☐ no **Did the pupil dilating drops bother you?** ☐ not applicable

[redacted]

[redacted]

[redacted]

[redacted]Questionnaire

Name [redacted] **Date**[redacted]

BEFORE telediagnosis

Thank you for your willingness to participate in this research project.

You are about to undergo digital photographing and diagnosis over the internet by ophthalmologists who are retinal specialists at the University of Iowa. They will evaluate your photographs for signs of diabetic retinopathy, a complication of diabetes. This new technique is called 'telediagnosis'. Until telediagnosis was available, you could only be screened in an ophthalmologist's office with a dilated retinal exam. Please give your best answer.

What number best expresses where you think the *quality* of the retinal exam is best:

1 (circled) (Better in ophthalmologist's office)	2	3 undecided	4	5 (Better through Telediagnosis)

What number best expresses *where* you prefer to have the retinal exam:

1 (circled) (Prefer ophthalmologist's office)	2	3 undecided	4	5 (Prefer family physician)

☐ yes ☑ no **Do you expect the camera flashlight to bother you?**

☐ yes ☑ no **Do you expect the pupil dilating drops to bother you?**

☑ yes ☐ no **In the past five years have you been examined by an ophthalmologist?**

☑ yes ☐ no **Do you currently carry health insurance?**

☑ yes ☐ no **Sometimes photographs can only be made if your pupils are made larger with a drop. Were you aware of this?**

AFTER telediagnosis

What number best expresses your *satisfaction* with telediagnosis:

1 (Terrible)	2	3 undecided	4	5 (Very good)

What number best expresses your where you think the *quality* of the retinal exam is best::

1 (Better in ophthalmologist's office)	2	3 undecided	4	5 (Better through Telediagnosis)

What number best expresses *where* you prefer to have the retinal exam <u>now</u>:

1 (Prefer ophthalmologist's office)	2	3 undecided	4	5 (Prefer family physician)

☐ yes ☐ no **Did the camera flashlight bother you?**

☐ yes ☐ no **Did the pupil dilating drops bother you?** ☐ not applicable

[redacted]

[redacted]

[redacted]

Patient Questionnaire

Name [redacted] Date [redacted]

BEFORE telediagnosis

Thank you for your willingness to participate in this research project.

You are about to undergo digital photographing and diagnosis over the internet by ophthalmologists who are retinal specialists at the University of Iowa. They will evaluate your photographs for signs of diabetic retinopathy, a complication of diabetes. This new technique is called 'telediagnosis'. Until telediagnosis was available, you could only be screened in an ophthalmologist's office with a dilated retinal exam. Please give your best answer.

What number best expresses where you think the *quality* of the retinal exam is best:

1	2	3	4	5
(Better in ophthalmologist's office)		undecided		(Better through Telediagnosis)

What number best expresses *where* you prefer to have the retinal exam:

1	2	3	4	5
(Prefer ophthalmologist's office)		undecided		(Prefer family physician)

☐ yes ☒ no **Do you expect the camera flashlight to bother you?**

☐ yes ☒ no **Do you expect the pupil dilating drops to bother you?**

☒ yes ☐ no **In the past five years have you been examined by an ophthalmologist?**

☒ yes ☐ no **Do you currently carry health insurance?**

☐ yes ☒ no **Sometimes photographs can only be made if your pupils are made larger with a drop. Were you aware of this?**

AFTER telediagnosis

What number best expresses your *satisfaction* with telediagnosis:

1	2	3	4	5
(Terrible)		undecided		(Very good)

What number best expresses your where you think the *quality* of the retinal exam is best::

1	2	3	4	5
(Better in ophthalmologist's office)		undecided		(Better through Telediagnosis)

What number best expresses *where* you prefer to have the retinal exam <u>now</u>:

1	2	3	4	5
(Prefer ophthalmologist's office)		undecided		(Prefer family physician)

☐ yes ☒ no **Did the camera flashlight bother you?**

☐ yes ☐ no **Did the pupil dilating drops bother you?** ☒ not applicable

[redacted]

[redacted]

[redacted] Questionnaire

Name [redacted] Date [redacted]

BEFORE telediagnosis

Thank you for your willingness to participate in this research project.

You are about to undergo digital photographing and diagnosis over the internet by ophthalmologists who are retinal specialists at the University of Iowa. They will evaluate your photographs for signs of diabetic retinopathy, a complication of diabetes. This new technique is called 'telediagnosis'. Until telediagnosis was available, you could only be screened in an ophthalmologist's office with a dilated retinal exam. Please give your best answer.

What number best expresses where you think the *quality* of the retinal exam is best:

(1)	2	3	4	5
(Better in ophthalmologist's office)		undecided	(Better through Telediagnosis)	

What number best expresses *where* you prefer to have the retinal exam:

1	2	3	(4)	5
(Prefer ophthalmologist's office)		undecided	(Prefer family physician)	

☐ yes ☒ no **Do you expect the camera flashlight to bother you?**

☐ yes ☒ no **Do you expect the pupil dilating drops to bother you?**

☐ yes ☒ no **In the past five years have you been examined by an ophthalmologist?**

☒ yes ☐ no **Do you currently carry health insurance?**

☐ yes ☒ no **Sometimes photographs can only be made if your pupils are made larger with a drop. Were you aware of this?**

AFTER telediagnosis

What number best expresses your *satisfaction* with telediagnosis:

1	2	(3)	4	5
(Terrible)		undecided		(Very good)

What number best expresses your where you think the *quality* of the retinal exam is best::

1	2	3	4	(5)
(Better in ophthalmologist's office)		undecided	(Better through Telediagnosis)	

What number best expresses *where* you prefer to have the retinal exam <u>now</u>:

1	2	3	4	(5)
(Prefer ophthalmologist's office)		undecided	(Prefer family physician)	

☐ yes ☒ no **Did the camera flashlight bother you?**

☐ yes ☐ no **Did the pupil dilating drops bother you?** ☒ not applicable

[redacted]

[redacted]

Patient Questionnaire

Name [redacted] ______ Date [redacted]

BEFORE telediagnosis

Thank you for your willingness to participate in this research project.

You are about to undergo digital photographing and diagnosis over the internet by ophthalmologists who are retinal specialists at the University of Iowa. They will evaluate your photographs for signs of diabetic retinopathy, a complication of diabetes. This new technique is called 'telediagnosis'. Until telediagnosis was available, you could only be screened in an ophthalmologist's office with a dilated retinal exam. Please give your best answer.

What number best expresses where you think the *quality* of the retinal exam is best:

1	2	3	4	5
(Better in ophthalmologist's office)		undecided		(Better through Telediagnosis)

What number best expresses *where* you prefer to have the retinal exam:

1	2	3	4	5
(Prefer ophthalmologist's office)		undecided		(Prefer family physician)

☐ yes ☑ no **Do you expect the camera flashlight to bother you?**

☐ yes ☑ no **Do you expect the pupil dilating drops to bother you?**

☐ yes ☑ no **In the past five years have you been examined by an ophthalmologist?**

☑ yes ☐ no **Do you currently carry health insurance?**

☑ yes ☐ no **Sometimes photographs can only be made if your pupils are made larger with a drop. Were you aware of this?**

AFTER telediagnosis

What number best expresses your *satisfaction* with telediagnosis:

1	2	3	4	5
(Terrible)		undecided		(Very good)

What number best expresses your where you think the *quality* of the retinal exam is best::

1	2	3	4	5
(Better in ophthalmologist's office)		undecided		(Better through Telediagnosis)

What number best expresses *where* you prefer to have the retinal exam now:

1	2	3	4	5
(Prefer ophthalmologist's office)		undecided		(Prefer family physician)

☐ yes ☑ no **Did the camera flashlight bother you?**

☐ yes ☐ no **Did the pupil dilating drops bother you?** ☑ not applicable

[redacted]

[redacted]

Patient Questionnaire

Name [redacted] Date [redacted]

BEFORE telediagnosis

Thank you for your willingness to participate in this research project.

You are about to undergo digital photographing and diagnosis over the internet by ophthalmologists who are retinal specialists at the University of Iowa. They will evaluate your photographs for signs of diabetic retinopathy, a complication of diabetes. This new technique is called 'telediagnosis'. Until telediagnosis was available, you could only be screened in an ophthalmologist's office with a dilated retinal exam. Please give your best answer.

What number best expresses where you think the *quality* of the retinal exam is best:

1 (circled)	2	3	4	5
(Better in ophthalmologist's office)		undecided		(Better through Telediagnosis)

What number best expresses *where* you prefer to have the retinal exam:

1	2	3	4 (circled)	5
(Prefer ophthalmologist's office)		undecided		(Prefer family physician)

☐ yes ☒ no **Do you expect the camera flashlight to bother you?**

☒ yes ☐ no **Do you expect the pupil dilating drops to bother you?**

☒ yes ☐ no **In the past five years have you been examined by an ophthalmologist?**

☒ yes ☐ no **Do you currently carry health insurance?**

☒ yes ☐ no **Sometimes photographs can only be made if your pupils are made larger with a drop. Were you aware of this?**

AFTER telediagnosis

What number best expresses your *satisfaction* with telediagnosis:

1	2	3	4	5 (circled)
(Terrible)		undecided		(Very good)

What number best expresses your where you think the *quality* of the retinal exam is best::

1	2	3 (circled)	4	5
(Better in ophthalmologist's office)		undecided		(Better through Telediagnosis)

What number best expresses *where* you prefer to have the retinal exam now:

1	2	3	4	5 (circled)
(Prefer ophthalmologist's office)		undecided		(Prefer family physician)

☐ yes ☒ no **Did the camera flashlight bother you?**

☐ yes ☐ no **Did the pupil dilating drops bother you?** ☒ not applicable

[redacted]

[redacted]

Patient Questionnaire

Name [redacted] Date [redacted]

BEFORE telediagnosis

Thank you for your willingness to participate in this research project.

You are about to undergo digital photographing and diagnosis over the internet by ophthalmologists who are retinal specialists at the University of Iowa. They will evaluate your photographs for signs of diabetic retinopathy, a complication of diabetes. This new technique is called 'telediagnosis'. Until telediagnosis was available, you could only be screened in an ophthalmologist's office with a dilated retinal exam. Please give your best answer.

What number best expresses where you think the *quality* of the retinal exam is best:

1	2	3	4	5
(Better in ophthalmologist's office)		undecided		(Better through Telediagnosis)

What number best expresses *where* you prefer to have the retinal exam:

1	2	3	4	5
(Prefer ophthalmologist's office)		undecided		(Prefer family physician)

☐ yes ☐ no **Do you expect the camera flashlight to bother you?**

☐ yes ☐ no **Do you expect the pupil dilating drops to bother you?**

☐ yes ☐ no **In the past five years have you been examined by an ophthalmologist?**

☐ yes ☐ no **Do you currently carry health insurance?**

☐ yes ☐ no **Sometimes photographs can only be made if your pupils are made larger with a drop. Were you aware of this?**

AFTER telediagnosis

What number best expresses your *satisfaction* with telediagnosis:

1	2	3	4	5
(Terrible)		undecided		(Very good)

What number best expresses your where you think the *quality* of the retinal exam is best::

1	2	3	4	5
(Better in ophthalmologist's office)		undecided		(Better through Telediagnosis)

What number best expresses *where* you prefer to have the retinal exam now:

1	2	3	4	5
(Prefer ophthalmologist's office)		undecided		(Prefer family physician)

☐ yes ☐ no **Did the camera flashlight bother you?**

☐ yes ☐ no **Did the pupil dilating drops bother you?** ☐ not applicable

[redacted]

[redacted]

Patient Questionnaire

Name [redacted] Date [redacted]

BEFORE telediagnosis

Thank you for your willingness to participate in this research project.

You are about to undergo digital photographing and diagnosis over the internet by ophthalmologists who are retinal specialists at the University of Iowa. They will evaluate your photographs for signs of diabetic retinopathy, a complication of diabetes. This new technique is called 'telediagnosis'. Until telediagnosis was available, you could only be screened in an ophthalmologist's office with a dilated retinal exam. Please give your best answer.

What number best expresses where you think the *quality* of the retinal exam is best:

1	2	3	4	5
(Better in ophthalmologist's office)		undecided		(Better through Telediagnosis)

What number best expresses *where* you prefer to have the retinal exam:

1	2	3	4	5
(Prefer ophthalmologist's office)		undecided		(Prefer family physician)

☐ yes ☒ no **Do you expect the camera flashlight to bother you?**

☐ yes ☒ no **Do you expect the pupil dilating drops to bother you?**

☐ yes ☒ no **In the past five years have you been examined by an ophthalmologist?**

☒ yes ☒ no **Do you currently carry health insurance?** [redacted]

☒ yes ☐ no **Sometimes photographs can only be made if your pupils are made larger with a drop. Were you aware of this?**

AFTER telediagnosis

What number best expresses your *satisfaction* with telediagnosis:

1	2	3	4	5
(Terrible)		undecided		(Very good)

What number best expresses your where you think the *quality* of the retinal exam is best::

1	2	3	4	5
(Better in ophthalmologist's office)		undecided		(Better through Telediagnosis)

What number best expresses *where* you prefer to have the retinal exam now:

1	2	3	4	5
(Prefer ophthalmologist's office)		undecided		(Prefer family physician)

☒ yes ☐ no **Did the camera flashlight bother you?**

☐ yes ☐ no **Did the pupil dilating drops bother you?** ☒ not applicable

[redacted]

[redacted]

Patient Questionnaire

Name [redacted] Date [redacted]

BEFORE telediagnosis

Thank you for your willingness to participate in this research project.

You are about to undergo digital photographing and diagnosis over the internet by ophthalmologists who are retinal specialists at the University of Iowa. They will evaluate your photographs for signs of diabetic retinopathy, a complication of diabetes. This new technique is called 'telediagnosis'. Until telediagnosis was available, you could only be screened in an ophthalmologist's office with a dilated retinal exam. Please give your best answer.

What number best expresses where you think the *quality* of the retinal exam is best:

1	2	3	4	5
(Better in ophthalmologist's office)		undecided		(Better through Telediagnosis)

What number best expresses *where* you prefer to have the retinal exam:

1	2	3	4	5
(Prefer ophthalmologist's office)		undecided		(Prefer family physician)

☐ yes ☐ no **Do you expect the camera flashlight to bother you?**

☐ yes ☐ no **Do you expect the pupil dilating drops to bother you?**

☐ yes ☐ no **In the past five years have you been examined by an ophthalmologist?** [redacted]

☐ yes ☐ no **Do you currently carry health insurance?**

☐ yes ☐ no **Sometimes photographs can only be made if your pupils are made larger with a drop. Were you aware of this?** [redacted]

AFTER telediagnosis

What number best expresses your *satisfaction* with telediagnosis:

1	2	3	4	5
(Terrible)		undecided		(Very good)

What number best expresses your where you think the *quality* of the retinal exam is best::

1	2	3	4	5
(Better in ophthalmologist's office)		undecided		(Better through Telediagnosis)

What number best expresses *where* you prefer to have the retinal exam <u>now</u>:

1	2	3	4	5
(Prefer ophthalmologist's office)		undecided		(Prefer family physician)

☐ yes ☐ no **Did the camera flashlight bother you?**

☐ yes ☐ no **Did the pupil dilating drops bother you?** ☐ not applicable

[redacted]

[redacted]

Patient questionnaire

BEFORE telediagnosis

Name: [redacted]

Thank you for your willingness to participate in this research project. [redacted]

You are about to undergo digital photographing and diagnosis over the internet by ophthalmologists who are retinal specialists at the University of Iowa. They will evaluate your photographs for signs of diabetic retinopathy, a complication of diabetes. This new technique is called 'telediagnosis'. Until telediagnosis was available, you could only be screened in an ophthalmologist's office with a dilated retinal exam.

Please give your best answer to the following questions

Circle where you expect the quality of the retinal exam to be better:

1	2	3	4	5
Ophthalmologist's office			Telediagnosis	

Circle which retinal exam you prefer:

1	2	3	4	5
Ophthalmologist's office			Telediagnosis	

☐ yes ☑ no Do you expect the camera flashlight to bother you
☑ yes ☐ no Do you expect the pupil dilating drops to bother you
☑ yes ☐ no In the past five years have you been examined by an ophthalmologist
☑ yes ☐ no Do you currently carry health insurance
☐ yes ☑ no In some cases the photographs can only be made if your pupils are dilated with a drop. Were you aware of this?

Patient questionnaire

AFTER telediagnosis results have been discussed

Name: [redacted]

Circle your satisfaction with digital photography of your retina

1	2	3	4	5
Terrible				Very good

Circle where you think the retinal exam is better:

1	2	3	4	5
Ophthalmologist's office				Telediagnosis

Circle which retinal exam you prefer now:

1	2	3	4	5
Ophthalmologist's office				Telediagnosis

☐ yes ☑ no Did the camera flashlight bother you
☐ yes ☑ no Did the pupil dilating drops bother you

After form is completed, please enter into Excel spreadsheet or send to:

Dr. Michael D. Abramoff, MD, PhD
Department of Ophthalmology and Visual Sciences
PFP 11290C
University of Iowa Hospitals and Clinics
200 Hawkins, Iowa City, IA 52242
Tel (319) 384 5833, fax: (319) 353 7996

[redacted]

[redacted]

Patient Questionnaire

Name [redacted] **Date** [redacted]

BEFORE telediagnosis

Thank you for your willingness to participate in this research project.

You are about to undergo digital photographing and diagnosis over the internet by ophthalmologists who are retinal specialists at the University of Iowa. They will evaluate your photographs for signs of diabetic retinopathy, a complication of diabetes. This new technique is called 'telediagnosis'. Until telediagnosis was available, you could only be screened in an ophthalmologist's office with a dilated retinal exam. Please give your best answer.

What number best expresses where you think the *quality* of the retinal exam is best:

1	2	3	4	5
(Better in ophthalmologist's office)		undecided		(Better through Telediagnosis)

What number best expresses *where* you prefer to have the retinal exam:

1	2	3	4	5
(Prefer ophthalmologist's office)		undecided		(Prefer family physician)

☐ yes ■ no **Do you expect the camera flashlight to bother you?**

☐ yes ■ no **Do you expect the pupil dilating drops to bother you?**

☐ yes ■ no **In the past five years have you been examined by an ophthalmologist?**

■ yes ☐ no **Do you currently carry health insurance?**

■ yes ☐ no **Sometimes photographs can only be made if your pupils are made larger with a drop. Were you aware of this?**

AFTER telediagnosis

What number best expresses your *satisfaction* with telediagnosis:

1	2	3	4	5
(Terrible)		undecided		(Very good)

What number best expresses your where you think the *quality* of the retinal exam is best::

1	2	3	4	5
(Better in ophthalmologist's office)		undecided		(Better through Telediagnosis)

What number best expresses *where* you prefer to have the retinal exam <u>now</u>:

1	2	3	4	5
(Prefer ophthalmologist's office)		undecided		(Prefer family physician)

☐ yes ☐ no **Did the camera flashlight bother you?**

☐ yes ☐ no **Did the pupil dilating drops bother you?** ☐ not applicable

[redacted]

[redacted]

Patient Questionnaire

Name [redacted] **Date** [redacted]

BEFORE telediagnosis

Thank you for your willingness to participate in this research project.

You are about to undergo digital photographing and diagnosis over the internet by ophthalmologists who are retinal specialists at the University of Iowa. They will evaluate your photographs for signs of diabetic retinopathy, a complication of diabetes. This new technique is called 'telediagnosis'. Until telediagnosis was available, you could only be screened in an ophthalmologist's office with a dilated retinal exam. Please give your best answer.

What number best expresses where you think the *quality* of the retinal exam is best:

1	2	3	4	5
(Better in ophthalmologist's office)		undecided		(Better through Telediagnosis)

What number best expresses *where* you prefer to have the retinal exam:

1	2	3	4	5
(Prefer ophthalmologist's office)		undecided		(Prefer family physician)

☐ **yes** ☒ **no** **Do you expect the camera flashlight to bother you?**

☐ **yes** ☒ **no** **Do you expect the pupil dilating drops to bother you?**

☐ **yes** ☒ **no** **In the past five years have you been examined by an ophthalmologist?**

☒ **yes** ☐ **no** **Do you currently carry health insurance?**

☒ **yes** ☐ **no** **Sometimes photographs can only be made if your pupils are made larger with a drop. Were you aware of this?**

AFTER telediagnosis

What number best expresses your *satisfaction* with telediagnosis:

1	2	3	4	5
(Terrible)		undecided		(Very good)

What number best expresses your where you think the *quality* of the retinal exam is best::

1	2	3	4	5
(Better in ophthalmologist's office)		undecided		(Better through Telediagnosis)

What number best expresses *where* you prefer to have the retinal exam <u>now</u>:

1	2	3	4	5
(Prefer ophthalmologist's office)		undecided		(Prefer family physician)

☐ **yes** ☒ **no** **Did the camera flashlight bother you?**

☐ **yes** ☐ **no** **Did the pupil dilating drops bother you?** ☒ not applicable

[redacted]

[redacted]

Patient Questionnaire

Name [redacted] Date [redacted]

BEFORE telediagnosis

Thank you for your willingness to participate in this research project.

You are about to undergo digital photographing and diagnosis over the internet by ophthalmologists who are retinal specialists at the University of Iowa. They will evaluate your photographs for signs of diabetic retinopathy, a complication of diabetes. This new technique is called 'telediagnosis'. Until telediagnosis was available, you could only be screened in an ophthalmologist's office with a dilated retinal exam. Please give your best answer.

What number best expresses where you think the *quality* of the retinal exam is best:

1 (Better in ophthalmologist's office)	2	3 undecided	4	5 (Better through Telediagnosis)

What number best expresses *where* you prefer to have the retinal exam:

1 (Prefer ophthalmologist's office)	2	3 undecided	4	5 (Prefer family physician)

☐ yes ☒ no **Do you expect the camera flashlight to bother you?**

☐ yes ☒ no **Do you expect the pupil dilating drops to bother you?**

☒ yes ☐ no **In the past five years have you been examined by an ophthalmologist?**

☒ yes ☐ no **Do you currently carry health insurance?**

☐ yes ☒ no **Sometimes photographs can only be made if your pupils are made larger with a drop. Were you aware of this?**

AFTER telediagnosis

What number best expresses your *satisfaction* with telediagnosis:

1 (Terrible)	2	3 undecided	4	5 (Very good)

What number best expresses your where you think the *quality* of the retinal exam is best::

1 (Better in ophthalmologist's office)	2	3 undecided	4	5 (Better through Telediagnosis)

What number best expresses *where* you prefer to have the retinal exam <u>now</u>:

1 (Prefer ophthalmologist's office)	2	3 undecided	4	5 (Prefer family physician)

☐ yes ☒ no **Did the camera flashlight bother you?**

☐ yes ☐ no **Did the pupil dilating drops bother you?** ☒ not applicable

[redacted]

[redacted]

Patient Questionnaire

Name [redacted] Date [redacted]

BEFORE telediagnosis

Thank you for your willingness to participate in this research project.

You are about to undergo digital photographing and diagnosis over the internet by ophthalmologists who are retinal specialists at the University of Iowa. They will evaluate your photographs for signs of diabetic retinopathy, a complication of diabetes. This new technique is called 'telediagnosis'. Until telediagnosis was available, you could only be screened in an ophthalmologist's office with a dilated retinal exam. Please give your best answer.

What number best expresses where you think the *quality* of the retinal exam is best:

1	2	3	4	5
(Better in ophthalmologist's office)		undecided	(Better through Telediagnosis)	

(3 circled)

What number best expresses *where* you prefer to have the retinal exam:

1	2	3	4	5
(Prefer ophthalmologist's office)		undecided	(Prefer family physician)	

(5 circled)

☐ yes ☒ no **Do you expect the camera flashlight to bother you?**

☐ yes ☒ no **Do you expect the pupil dilating drops to bother you?**

☒ yes ☐ no **In the past five years have you been examined by an ophthalmologist?**

☒ yes ☐ no **Do you currently carry health insurance?**

☒ yes ☐ no **Sometimes photographs can only be made if your pupils are made larger with a drop. Were you aware of this?**

AFTER telediagnosis

What number best expresses your *satisfaction* with telediagnosis:

1	2	3	4	5
(Terrible)		undecided		(Very good)

(5 circled)

What number best expresses your where you think the *quality* of the retinal exam is best::

1	2	3	4	5
(Better in ophthalmologist's office)		undecided	(Better through Telediagnosis)	

(5 circled)

What number best expresses *where* you prefer to have the retinal exam now:

1	2	3	4	5
(Prefer ophthalmologist's office)		undecided	(Prefer family physician)	

(5 circled)

☐ yes ☒ no **Did the camera flashlight bother you?**

☐ yes ☐ no **Did the pupil dilating drops bother you?** ☐ not applicable

[redacted]

[redacted]

[redacted]t Questionnaire

Name [redacted] Date [redacted]

BEFO[redacted]

Thank you for your willingness to participate in this research project.

You are about to undergo digital photographing and diagnosis over the internet by ophthalmologists who are retinal specialists at the University of Iowa. They will evaluate your photographs for signs of diabetic retinopathy, a complication of diabetes. This new technique is called 'telediagnosis'. Until telediagnosis was available, you could only be screened in an ophthalmologist's office with a dilated retinal exam. Please give your best answer.

What number best expresses where you think the *quality* of the retinal exam is best:

1 (Better in ophthalmologist's office)	2	3 undecided	4	5 (Better through Telediagnosis)

What number best expresses *where* you prefer to have the retinal exam:

1 (Prefer ophthalmologist's office)	2	3 undecided	4	5 (Prefer family physician)

☐ yes ☒ no **Do you expect the camera flashlight to bother you?**

☐ yes ☒ no **Do you expect the pupil dilating drops to bother you?**

☒ yes ☐ no **In the past five years have you been examined by an ophthalmologist?**

☒ yes ☐ no **Do you currently carry health insurance?**

☒ yes ☐ no **Sometimes photographs can only be made if your pupils are made larger with a drop. Were you aware of this?**

AFTER telediagnosis

What number best expresses your *satisfaction* with telediagnosis:

1 (Terrible)	2	3 undecided	4	5 (Very good)

What number best expresses your where you think the *quality* of the retinal exam is best::

1 (Better in ophthalmologist's office)	2	3 undecided	4	5 (Better through Telediagnosis)

What number best expresses *where* you prefer to have the retinal exam <u>now</u>:

1 (Prefer ophthalmologist's office)	2	3 undecided	4	5 (Prefer family physician)

☐ yes ☒ no **Did the camera flashlight bother you?**

☐ yes ☒ no **Did the pupil dilating drops bother you?** ☐ not applicable

[redacted]

[redacted]

[redacted]uestionnaire

Name [redacted] Date [redacted]

BEFORE telediagnosis

Thank you for your willingness to participate in this research project.

You are about to undergo digital photographing and diagnosis over the internet by ophthalmologists who are retinal specialists at the University of Iowa. They will evaluate your photographs for signs of diabetic retinopathy, a complication of diabetes. This new technique is called 'telediagnosis'. Until telediagnosis was available, you could only be screened in an ophthalmologist's office with a dilated retinal exam. Please give your best answer.

What number best expresses where you think the *quality* of the retinal exam is best:

1	2	3	4	5
(Better in ophthalmologist's office)		undecided		(Better through Telediagnosis)

What number best expresses *where* you prefer to have the retinal exam:

1	2	3	4	5
(Prefer ophthalmologist's office)		undecided		(Prefer family physician)

☐ yes ☐ no **Do you expect the camera flashlight to bother you?**

☐ yes ☐ no **Do you expect the pupil dilating drops to bother you?**

☐ yes ☐ no **In the past five years have you been examined by an ophthalmologist?** [redacted]

☐ yes ☐ no **Do you currently carry health insurance?**

☐ yes ☐ no **Sometimes photographs can only be made if your pupils are made larger with a drop. Were you aware of this?** [redacted]

AFTER telediagnosis

What number best expresses your *satisfaction* with telediagnosis:

1	2	3	4	5
(Terrible)		undecided		(Very good)

What number best expresses your where you think the *quality* of the retinal exam is best::

1	2	3	4	5
(Better in ophthalmologist's office)		undecided		(Better through Telediagnosis)

What number best expresses *where* you prefer to have the retinal exam now:

1	2	3	4	5
(Prefer ophthalmologist's office)		undecided		(Prefer family physician)

☐ yes ☐ no **Did the camera flashlight bother you?**

☐ yes ☐ no **Did the pupil dilating drops bother you?** ☐ not applicable

[redacted]

[redacted]

[redacted]

Patient Questionnaire

Name [redacted] Date [redacted]

BEFORE telediagnosis

Thank you for your willingness to participate in this research project.

You are about to undergo digital photographing and diagnosis over the internet by ophthalmologists who are retinal specialists at the University of Iowa. They will evaluate your photographs for signs of diabetic retinopathy, a complication of diabetes. This new technique is called 'telediagnosis'. Until telediagnosis was available, you could only be screened in an ophthalmologist's office with a dilated retinal exam. Please give your best answer.

What number best expresses where you think the *quality* of the retinal exam is best:

1 (Better in ophthalmologist's office)	2	3 undecided	4	5 (Better through Telediagnosis)

What number best expresses *where* you prefer to have the retinal exam:

1 (Prefer ophthalmologist's office)	2	3 undecided	4	5 (Prefer family physician)

☐ yes ☒ no **Do you expect the camera flashlight to bother you?**

☐ yes ☒ no **Do you expect the pupil dilating drops to bother you?**

☒ yes ☐ no **In the past five years have you been examined by an ophthalmologist?**

☒ yes ☐ no **Do you currently carry health insurance?**

☒ yes ☐ no **Sometimes photographs can only be made if your pupils are made larger with a drop. Were you aware of this?**

AFTER telediagnosis

What number best expresses your *satisfaction* with telediagnosis:

1 (Terrible)	2	3 undecided	4	5 (Very good)

What number best expresses your where you think the *quality* of the retinal exam is best::

1 (Better in ophthalmologist's office)	2	3 undecided	4	5 (Better through Telediagnosis)

What number best expresses *where* you prefer to have the retinal exam now:

1 (Prefer ophthalmologist's office)	2	3 undecided	4	5 (Prefer family physician)

☐ yes ☑ no **Did the camera flashlight bother you?**

☐ yes ☑ no **Did the pupil dilating drops bother you?** ☐ not applicable

[redacted]

[redacted]

Patient questionnaire [redacted]

BEFORE telediagnosis

Name: [redacted]

Thank you for your willingness to participate in this research project.
You are about to undergo digital photographing and diagnosis over the internet by ophthalmologists who are retinal specialists at the University of Iowa. They will evaluate your photographs for signs of diabetic retinopathy, a complication of diabetes. This new technique is called 'telediagnosis'. Until telediagnosis was available, you could only be screened in an ophthalmologist's office with a dilated retinal exam.

Please give your best answer to the following questions

Circle where you expect the quality of the retinal exam to be better:

1	2	3	4	5
Ophthalmologist's office			Telediagnosis	

Circle which retinal exam you prefer:

1	2	3	4	5
Ophthalmologist's office			Telediagnosis	

☐ yes ☑ no Do you expect the camera flashlight to bother you
☐ yes ☑ no Do you expect the pupil dilating drops to bother you
☐ yes ☑ no In the past five years have you been examined by an ophthalmologist
☑ yes ☐ no Do you currently carry health insurance
☑ yes ☐ no In some cases the photographs can only be made if your pupils are dilated with a drop. Were you aware of this?

Patient questionnaire

[redacted] telediagnosis results have been discussed

Name: [redacted] Date:______________

Circle your satisfaction with digital photography of your retina

1	2	3	4	5
Terrible				Very good

Circle where you think the retinal exam is better:

1	2	3	4	5
Ophthalmologist's office				Telediagnosis

Circle which retinal exam you prefer now:

1	2	3	4	5
Ophthalmologist's office				Telediagnosis

☐ yes ☑ no Did the camera flashlight bother you
☐ yes ☑ no Did the pupil dilating drops bother you

After form is completed, please enter into Excel spreadsheet or send to:

Dr. Michael D. Abramoff, MD, PhD
Department of Ophthalmology and Visual Sciences
PFP 11290C
University of Iowa Hospitals and Clinics
200 Hawkins, Iowa City, IA 52242
Tel (319) 384 5833, fax: (319) 353 7996

[redacted]

[redacted]

Patient Questionnaire

Name [redacted] Date [redacted]

BEFORE telediagnosis

Thank you for your willingness to participate in this research project.

You are about to undergo digital photographing and diagnosis over the internet by ophthalmologists who are retinal specialists at the University of Iowa. They will evaluate your photographs for signs of diabetic retinopathy, a complication of diabetes. This new technique is called 'telediagnosis'. Until telediagnosis was available, you could only be screened in an ophthalmologist's office with a dilated retinal exam. Please give your best answer.

What number best expresses where you think the *quality* of the retinal exam is best:

1	2	3	4	5
(Better in ophthalmologist's office)		undecided		(Better through Telediagnosis)

What number best expresses *where* you prefer to have the retinal exam:

1	2	3	4	5
(Prefer ophthalmologist's office)		undecided		(Prefer family physician)

☒ yes ☐ no **Do you expect the camera flashlight to bother you?**

☒ yes ☐ no **Do you expect the pupil dilating drops to bother you?**

☒ yes ☐ no **In the past five years have you been examined by an ophthalmologist?**

☒ yes ☐ no **Do you currently carry health insurance?**

☐ yes ☒ no **Sometimes photographs can only be made if your pupils are made larger with a drop. Were you aware of this?**

AFTER telediagnosis

What number best expresses your *satisfaction* with telediagnosis:

1	2	3	4	5
(Terrible)		undecided		(Very good)

What number best expresses your where you think the *quality* of the retinal exam is best::

1	2	3	4	5
(Better in ophthalmologist's office)		undecided		(Better through Telediagnosis)

What number best expresses *where* you prefer to have the retinal exam now:

1	2	3	4	5
(Prefer ophthalmologist's office)		undecided		(Prefer family physician)

☐ yes ☒ no **Did the camera flashlight bother you?**

☐ yes ☒ no **Did the pupil dilating drops bother you?** ☐ not applicable

[redacted]

[redacted]

Patient Questionnaire

Name [redacted] **Date** [redacted]

BEFORE telediagnosis

Thank you for your willingness to participate in this research project.

You are about to undergo digital photographing and diagnosis over the internet by ophthalmologists who are retinal specialists at the University of Iowa. They will evaluate your photographs for signs of diabetic retinopathy, a complication of diabetes. This new technique is called 'telediagnosis'. Until telediagnosis was available, you could only be screened in an ophthalmologist's office with a dilated retinal exam. Please give your best answer.

What number best expresses where you think the *quality* of the retinal exam is best:

1	2	3	4	5
(Better in ophthalmologist's office)		undecided		(Better through Telediagnosis)

What number best expresses *where* you prefer to have the retinal exam:

1	2	3	4	5
(Prefer ophthalmologist's office)		undecided		(Prefer family physician)

☐ yes ☐ no **Do you expect the camera flashlight to bother you?**

☐ yes ☐ no **Do you expect the pupil dilating drops to bother you?**

☐ yes ☐ no **In the past five years have you been examined by an ophthalmologist?**

☐ yes ☐ no **Do you currently carry health insurance?**

☐ yes ☐ no **Sometimes photographs can only be made if your pupils are made larger with a drop. Were you aware of this?**

AFTER telediagnosis

What number best expresses your *satisfaction* with telediagnosis:

1	2	3	4	5
(Terrible)		undecided		(Very good)

What number best expresses your where you think the *quality* of the retinal exam is best::

1	2	3	4	5
(Better in ophthalmologist's office)		undecided		(Better through Telediagnosis)

What number best expresses *where* you prefer to have the retinal exam now:

1	2	3	4	5
(Prefer ophthalmologist's office)		undecided		(Prefer family physician)

☐ yes ☐ no **Did the camera flashlight bother you?**

☐ yes ☐ no **Did the pupil dilating drops bother you?** ☐ not applicable

[redacted]

[redacted]

Patient questionnaire

BEFORE telediagnosis

Name:[redacted]

Thank you for your willingness to participate in this research project.

You are about to undergo digital photographing and diagnosis over the internet by ophthalmologists who are retinal specialists at the University of Iowa. They will evaluate your photographs for signs of diabetic retinopathy, a complication of diabetes. This new technique is called 'telediagnosis'. Until telediagnosis was available, you could only be screened in an ophthalmologist's office with a dilated retinal exam.

Please give your best answer to the following questions

Circle where you expect the quality of the retinal exam to be better:

1 2 3 4 5

Ophthalmologist's office — Telediagnosis

Circle which retinal exam you prefer:

1 2 3 4 5

Ophthalmologist's office — Telediagnosis

☐ yes ☒ no Do you expect the camera flashlight to bother you
☐ yes ☒ no Do you expect the pupil dilating drops to bother you
☐ yes ☒ no In the past five years have you been examined by an ophthalmologist
☒ yes ☐ no Do you currently carry health insurance
☒ yes ☐ no In some cases the photographs can only be made if your pupils are dilated with a drop. Were you aware of this?

Patient questionnaire

[redacted]osis results have been discussed

Name:[redacted] **Date:**________________

Circle your satisfaction with digital photography of your retina

1 2 3 4 5

Terrible — Very good

Circle where you think the retinal exam is better:

1 2 3 4 5

Ophthalmologist's office — Telediagnosis

Circle which retinal exam you prefer now:

1 2 3 4 5

Ophthalmologist's office — Telediagnosis

☐ yes ☒ no Did the camera flashlight bother you
☐ yes ☒ no Did the pupil dilating drops bother you

After form is completed, please enter into Excel spreadsheet or send to:

Dr. Michael D. Abramoff, MD, PhD
Department of Ophthalmology and Visual Sciences
PFP 11290C
University of Iowa Hospitals and Clinics
200 Hawkins, Iowa City, IA 52242
Tel (319) 384 5833, fax: (319) 353 7996

[redacted]

[redacted]

[redacted]

Patient Questionnaire

Name [redacted] **Date** [redacted]

BEFORE telediagnosis

Thank you for your willingness to participate in this research project.

You are about to undergo digital photographing and diagnosis over the internet by ophthalmologists who are retinal specialists at the University of Iowa. They will evaluate your photographs for signs of diabetic retinopathy, a complication of diabetes. This new technique is called 'telediagnosis'. Until telediagnosis was available, you could only be screened in an ophthalmologist's office with a dilated retinal exam. Please give your best answer.

What number best expresses where you think the *quality* of the retinal exam is best:

1<br>(Better in ophthalmologist's office)	2	3<br>undecided	4	5<br>(Better through Telediagnosis)

What number best expresses *where* you prefer to have the retinal exam:

1<br>(Prefer ophthalmologist's office)	2	3<br>undecided	4	5<br>(Prefer family physician)

☐ yes ☒ no **Do you expect the camera flashlight to bother you?**

☒ yes ☐ no **Do you expect the pupil dilating drops to bother you?**

☒ yes ☐ no **In the past five years have you been examined by an ophthalmologist?**

☒ yes ☐ no **Do you currently carry health insurance?**

☐ yes ☒ no **Sometimes photographs can only be made if your pupils are made larger with a drop. Were you aware of this?**

AFTER telediagnosis

What number best expresses your *satisfaction* with telediagnosis:

1<br>(Terrible)	2	3<br>undecided	4 [redacted]	5<br>(Very good)

What number best expresses your where you think the *quality* of the retinal exam is best::

1<br>(Better in ophthalmologist's office)	2	3<br>undecided	4	5<br>(Better through Telediagnosis)

What number best expresses *where* you prefer to have the retinal exam <u>now</u>:

1<br>(Prefer ophthalmologist's office)	2	3<br>undecided	4	5<br>(Prefer family physician)

☐ yes ☒ no **Did the camera flashlight bother you?**

☐ yes ☐ no **Did the pupil dilating drops bother you?** ☒ not applicable

[redacted]

[redacted]

Patient Questionnaire

Name [redacted] Date [redacted]

BEFORE telediagnosis

Thank you for your willingness to participate in this research project.

You are about to undergo digital photographing and diagnosis over the internet by ophthalmologists who are retinal specialists at the University of Iowa. They will evaluate your photographs for signs of diabetic retinopathy, a complication of diabetes. This new technique is called 'telediagnosis'. Until telediagnosis was available, you could only be screened in an ophthalmologist's office with a dilated retinal exam. Please give your best answer.

What number best expresses where you think the *quality* of the retinal exam is best:

1<br>(Better in ophthalmologist's office)	2	3<br>undecided	4<br>(Better through Telediagnosis)	5

What number best expresses *where* you prefer to have the retinal exam:

1<br>(Prefer ophthalmologist's office)	2	3<br>undecided	4<br>(Prefer family physician)	5

☐ yes ☐ no **Do you expect the camera flashlight to bother you?**

☐ yes ☐ no **Do you expect the pupil dilating drops to bother you?**

☐ yes ☐ no **In the past five years have you been examined by an ophthalmologist?**

☐ yes ☐ no **Do you currently carry health insurance?**

☐ yes ☐ no **Sometimes photographs can only be made if your pupils are made larger with a drop. Were you aware of this?**

AFTER telediagnosis

What number best expresses your *satisfaction* with telediagnosis:

1<br>(Terrible)	2	3<br>undecided	4	5<br>(Very good)

What number best expresses your where you think the *quality* of the retinal exam is best::

1<br>(Better in ophthalmologist's office)	2	3<br>undecided	4<br>(Better through Telediagnosis)	5

What number best expresses *where* you prefer to have the retinal exam <u>now</u>:

1<br>(Prefer ophthalmologist's office)	2	3<br>undecided	4<br>(Prefer family physician)	5

☐ yes ☐ no **Did the camera flashlight bother you?**

☐ yes ☐ no **Did the pupil dilating drops bother you?** ☐ not applicable

[redacted]

[redacted]

[redacted]

Patient questionnaire

BEFORE telediagnosis

Name:___ [redacted]

Thank you for your willingness to participate in this research project.

You are about to undergo digital photographing and diagnosis over the internet by ophthalmologists who are retinal specialists at the University of Iowa. They will evaluate your photographs for signs of diabetic retinopathy, a complication of diabetes. This new technique is called 'telediagnosis'. Until telediagnosis was available, you could only be screened in an ophthalmologist's office with a dilated retinal exam.

Please give your best answer to the following questions

Circle where you expect the quality of the retinal exam to be better:

1	2	3	4	5
Ophthalmologist's office			Telediagnosis	

Circle which retinal exam you prefer:

1	2	3	4	5
Ophthalmologist's office			Telediagnosis	

☐ yes ☐ no Do you expect the camera flashlight to bother you
☐ yes ☐ no Do you expect the pupil dilating drops to bother you
☐ yes ☐ no In the past five years have you been examined by an ophthalmologist
☐ yes ☐ no Do you currently carry health insurance
☐ yes ☐ no In some cases the photographs can only be made if your pupils are dilated with a drop. Were you aware of this?

Patient questionnaire

AFTER telediagnosis results have been discussed

Name:________________________ **Date:**______________

Circle your satisfaction with digital photography of your retina

1	2	3	4	5
Terrible				Very good

Circle where you think the retinal exam is better:

1	2	3	4	5
Ophthalmologist's office				Telediagnosis

Circle which retinal exam you prefer now:

1	2	3	4	5
Ophthalmologist's office				Telediagnosis

☐ yes ☐ no Did the camera flashlight bother you
☐ yes ☐ no Did the pupil dilating drops bother you [redacted]

After form is completed, please enter into Excel spreadsheet or send to:

Dr. Michael D. Abramoff, MD, PhD
Department of Ophthalmology and Visual Sciences
PFP 11290C
University of Iowa Hospitals and Clinics
200 Hawkins, Iowa City, IA 52242
Tel (319) 384 5833, fax: (319) 353 7996

[redacted]

[redacted]

Patient questionnaire

[redacted]

Name: [redacted]

Thank you for your willingness to participate in this research project.

You are about to undergo digital photographing and diagnosis over the internet by ophthalmologists who are retinal specialists at the University of Iowa. They will evaluate your photographs for signs of diabetic retinopathy, a complication of diabetes. This new technique is called 'telediagnosis'. Until telediagnosis was available, you could only be screened in an ophthalmologist's office with a dilated retinal exam.

Please give your best answer to the following questions

Circle where you expect the quality of the retinal exam to be better:

1	2	3	4	5
Ophthalmologist's office			Telediagnosis	

Circle which retinal exam you prefer:

1	2	3	4	5
Ophthalmologist's office			Telediagnosis	

☐ yes ☐ no Do you expect the camera flashlight to bother you
☐ yes ☐ no Do you expect the pupil dilating drops to bother you
☐ yes ☐ no In the past five years have you been examined by an ophthalmologist
☐ yes ☐ no Do you currently carry health insurance
☐ yes ☐ no In some cases the photographs can only be made if your pupils are dilated with a drop. Were you aware of this?

Patient questionnaire

AFTER telediagnosis results have been discussed

Name:____________________ **Date:**__________

Circle your satisfaction with digital photography of your retina

1	2	3	4	5
Terrible				Very good

Circle where you think the retinal exam is better:

1	2	3	4	5
Ophthalmologist's office				Telediagnosis

Circle which retinal exam you prefer now:

1	2	3	4	5
Ophthalmologist's office				Telediagnosis

☐ yes ☐ no Did the camera flashlight bother you
☐ yes ☐ no Did the [redacted] drops bother you

[redacted]

After form is completed, please enter into Excel spreadsheet or send to:

Dr. Michael D. Abramoff, MD, PhD
Department of Ophthalmology and Visual Sciences
PFP 11290C
University of Iowa Hospitals and Clinics
200 Hawkins, Iowa City, IA 52242
Tel (319) 384 5833, fax: (319) 353 7996

[redacted]

[redacted]

Patient Questionnaire

Name [redacted] Date [redacted]

BEFORE telediagnosis

Thank you for your willingness to participate in this research project.

You are about to undergo digital photographing and diagnosis over the internet by ophthalmologists who are retinal specialists at the University of Iowa. They will evaluate your photographs for signs of diabetic retinopathy, a complication of diabetes. This new technique is called 'telediagnosis'. Until telediagnosis was available, you could only be screened in an ophthalmologist's office with a dilated retinal exam. Please give your best answer.

What number best expresses where you think the *quality* of the retinal exam is best:

1	2	3	4	5
(Better in ophthalmologist's office)		undecided		(Better through Telediagnosis)

What number best expresses *where* you prefer to have the retinal exam:

1	2	3	4	5
(Prefer ophthalmologist's office)		undecided		(Prefer family physician)

☐ yes ☒ no **Do you expect the camera flashlight to bother you?**

☒ yes ☐ no **Do you expect the pupil dilating drops to bother you?**

☒ yes ☐ no **In the past five years have you been examined by an ophthalmologist?**

☒ yes ☐ no **Do you currently carry health insurance?**

☒ yes ☒ no **Sometimes photographs can only be made if your pupils are made larger with a drop. Were you aware of this?**

AFTER telediagnosis

What number best expresses your *satisfaction* with telediagnosis:

1	2	3	4	5
(Terrible)		undecided		(Very good)

What number best expresses your where you think the *quality* of the retinal exam is best::

1	2	3	4	5
(Better in ophthalmologist's office)		undecided		(Better through Telediagnosis)

What number best expresses *where* you prefer to have the retinal exam now:

1	2	3	4	5
(Prefer ophthalmologist's office)		undecided		(Prefer family physician)

☐ yes ☒ no **Did the camera flashlight bother you?**

☐ yes ☐ no **Did the pupil dilating drops bother you?** ☒ not applicable [redacted]

[redacted]

Patient questionnaire

BEFORE telediagnosis

Name:[redacted]

Thank you for your willingness to participate in this research project.

You are about to undergo digital photographing and diagnosis over the internet by ophthalmologists who are retinal specialists at the University of Iowa. They will evaluate your photographs for signs of diabetic retinopathy, a complication of diabetes. This new technique is called 'telediagnosis'. Until telediagnosis was available, you could only be screened in an ophthalmologist's office with a dilated retinal exam.

Please give your best answer to the following questions

Circle where you expect the quality of the retinal exam to be better:

1 2 (3) 4 5

Ophthalmologist's office — Telediagnosis

Circle which retinal exam you prefer:

1 2 (3) 4 5

Ophthalmologist's office — Telediagnosis

- □ yes ☒ no Do you expect the camera flashlight to bother you
- □ yes ☒ no Do you expect the pupil dilating drops to bother you
- □ yes ☒ no In the past five years have you been examined by an ophthalmologist
- ☒ yes □ no Do you currently carry health insurance
- ☒ yes □ no In some cases the photographs can only be made if your pupils are dilated with a drop. Were you aware of this?

Patient questionnaire

AFTER telediagnosis results have been discussed

Name:____________________ **Date:**____________

Circle your satisfaction with digital photography of your retina

1 2 3 4 (5)

Terrible — Very good

Circle where you think the retinal exam is better:

1 2 (3) 4 5

Ophthalmologist's office — Telediagnosis

Circle which retinal exam you prefer now:

1 2 3 4 (5)

Ophthalmologist's office — Telediagnosis

- □ yes ☒ no Did the camera flashlight bother you
- □ yes ☒ no Did the pupil dilating drops bother you [redacted]

After form is completed, please enter into Excel spreadsheet or send to:

Dr. Michael D. Abramoff, MD, PhD
Department of Ophthalmology and Visual Sciences
PFP 11290C
University of Iowa Hospitals and Clinics
200 Hawkins, Iowa City, IA 52242
Tel (319) 384 5833, fax: (319) 353 7996

[redacted]

[redacted]aire

[redacted]

Name [redacted] **Date** [redacted]

BEFORE telediagnosis

Thank you for your willingness to participate in this research project.

You are about to undergo digital photographing and diagnosis over the internet by ophthalmologists who are retinal specialists at the University of Iowa. They will evaluate your photographs for signs of diabetic retinopathy, a complication of diabetes. This new technique is called 'telediagnosis'. Until telediagnosis was available, you could only be screened in an ophthalmologist's office with a dilated retinal exam. Please give your best answer.

What number best expresses where you think the *quality* of the retinal exam is best:

1	2	3	4	5
(Better in ophthalmologist's office)		undecided		(Better through Telediagnosis)

What number best expresses *where* you prefer to have the retinal exam:

1	2	3	4	5
(Prefer ophthalmologist's office)		undecided		(Prefer family physician)

☐ yes ☒ no **Do you expect the camera flashlight to bother you?**

☒ yes ☐ no **Do you expect the pupil dilating drops to bother you?**

☐ yes ☒ no **In the past five years have you been examined by an ophthalmologist?**

☒ yes ☐ no **Do you currently carry health insurance?**

☒ yes ☐ no **Sometimes photographs can only be made if your pupils are made larger with a drop. Were you aware of this?**

AFTER telediagnosis

What number best expresses your *satisfaction* with telediagnosis:

1	2	3	4	5
(Terrible)		undecided		(Very good)

What number best expresses your where you think the *quality* of the retinal exam is best::

1	2	3	4	5
(Better in ophthalmologist's office)		undecided		(Better through Telediagnosis)

What number best expresses *where* you prefer to have the retinal exam <u>now</u>:

1	2	3	4	5
(Prefer ophthalmologist's office)		undecided		(Prefer family physician)

☐ yes ☐ no **Did the camera flashlight bother you?**

☐ yes ☐ no **Did the pupil dilating drops bother you?** ☐ not applicable

[redacted]

[redacted]

Patient Questionnaire

Name [redacted] **Date** [redacted]

BEFORE telediagnosis

Thank you for your willingness to participate in this research project.

You are about to undergo digital photographing and diagnosis over the internet by ophthalmologists who are retinal specialists at the University of Iowa. They will evaluate your photographs for signs of diabetic retinopathy, a complication of diabetes. This new technique is called 'telediagnosis'. Until telediagnosis was available, you could only be screened in an ophthalmologist's office with a dilated retinal exam. Please give your best answer.

What number best expresses where you think the *quality* of the retinal exam is best:

1	2	3	4	5
(Better in ophthalmologist's office)		undecided		(Better through Telediagnosis)

What number best expresses *where* you prefer to have the retinal exam:

1	2	3	4	5
(Prefer ophthalmologist's office)		undecided		(Prefer family physician)

☐ yes ☒ no **Do you expect the camera flashlight to bother you?**

☐ yes ☒ no **Do you expect the pupil dilating drops to bother you?**

☐ yes ☒ no **In the past five years have you been examined by an ophthalmologist?**

☒ yes ☐ no **Do you currently carry health insurance?**

☐ yes ☒ no **Sometimes photographs can only be made if your pupils are made larger with a drop. Were you aware of this?**

AFTER telediagnosis

What number best expresses your *satisfaction* with telediagnosis:

1	2	3	4	5
(Terrible)		undecided		(Very good)

What number best expresses your where you think the *quality* of the retinal exam is best::

1	2	3	4	5
(Better in ophthalmologist's office)		undecided		(Better through Telediagnosis)

What number best expresses *where* you prefer to have the retinal exam <u>now</u>:

1	2	3	4	5
(Prefer ophthalmologist's office)		undecided		(Prefer family physician)

☐ yes ☒ no **Did the camera flashlight bother you?**

☐ yes ☒ no **Did the pupil dilating drops bother you?** ☐ not applicable

[redacted]

[redacted]

Patient Questionnaire

Name [redacted] Date [redacted]

BEFORE telediagnosis

Thank you for your willingness to participate in this research project.

You are about to undergo digital photographing and diagnosis over the internet by ophthalmologists who are retinal specialists at the University of Iowa. They will evaluate your photographs for signs of diabetic retinopathy, a complication of diabetes. This new technique is called 'telediagnosis'. Until telediagnosis was available, you could only be screened in an ophthalmologist's office with a dilated retinal exam. Please give your best answer.

What number best expresses where you think the *quality* of the retinal exam is best:

1	2	3	4	5
(Better in ophthalmologist's office)		undecided		(Better through Telediagnosis)

What number best expresses *where* you prefer to have the retinal exam:

1	2	3	4	5
(Prefer ophthalmologist's office)		undecided		(Prefer family physician)

☐ yes ☐ no **Do you expect the camera flashlight to bother you?**

☐ yes ☐ no **Do you expect the pupil dilating drops to bother you?**

☐ yes ☐ no **In the past five years have you been examined by an ophthalmologist?**

☐ yes ☐ no **Do you currently carry health insurance?**

☐ yes ☐ no **Sometimes photographs can only be made if your pupils are made larger with a drop. Were you aware of this?**

AFTER telediagnosis

What number best expresses your *satisfaction* with telediagnosis:

1	2	3	4	5
(Terrible)		undecided		(Very good)

What number best expresses your where you think the *quality* of the retinal exam is best::

1	2	3	4	5
(Better in ophthalmologist's office)		undecided		(Better through Telediagnosis)

What number best expresses *where* you prefer to have the retinal exam now:

1	2	3	4	5
(Prefer ophthalmologist's office)		undecided		(Prefer family physician)

☐ yes ☐ no **Did the camera flashlight bother you?**

☐ yes ☐ no **Did the pupil dilating drops bother you?** ☐ not applicable

[redacted]

[redacted]

Patient questionnaire

BEFORE telediagnosis

Name: [redacted]

Thank you for your willingness to participate in this research project.

You are about to undergo digital photographing and diagnosis over the internet by ophthalmologists who are retinal specialists at the University of Iowa. They will evaluate your photographs for signs of diabetic retinopathy, a complication of diabetes. This new technique is called 'telediagnosis'. Until telediagnosis was available, you could only be screened in an ophthalmologist's office with a dilated retinal exam.

Please give your best answer to the following questions

Circle where you expect the quality of the retinal exam to be better:

1	2	3	4	5
Ophthalmologist's office			Telediagnosis	

Circle which retinal exam you prefer:

1	2	3	4	5
Ophthalmologist's office			Telediagnosis	

□ yes □ no Do you expect the camera flashlight to bother you
□ yes □ no Do you expect the pupil dilating drops to bother you
□ yes □ no In the past five years have you been examined by an ophthalmologist
□ yes □ no Do you currently carry health insurance
□ yes □ no In some cases the photographs can only be made if your pupils are dilated with a drop. Were you aware of this?

Patient questionnaire

AFTER telediagnosis results have been discussed

Name: [redacted] **Date:**__________

Circle your satisfaction with digital photography of your retina

1	2	3	4	5
Terrible				Very good

Circle where you think the retinal exam is better:

1	2	3	4	5
Ophthalmologist's office				Telediagnosis

Circle which retinal exam you prefer now:

1	2	3	4	5
Ophthalmologist's office				Telediagnosis

□ yes □ no Did the camera flashlight bother you
□ yes □ no Did the pupil dilating drops bother you

After form is completed, please enter into Excel spreadsheet or send to:

Dr. Michael D. Abramoff, MD, PhD
Department of Ophthalmology and Visual Sciences
PFP 11290C
University of Iowa Hospitals and Clinics
200 Hawkins, Iowa City, IA 52242
Tel (319) 384 5833, fax: (319) 353 7996

[redacted]

[redacted]

Patient questionnaire

BEFORE telediagnosis

Name: [redacted]

Thank you for your willingness to participate in this research project.

You are about to undergo digital photographing and diagnosis over the internet by ophthalmologists who are retinal specialists at the University of Iowa. They will evaluate your photographs for signs of diabetic retinopathy, a complication of diabetes. This new technique is called 'telediagnosis'. Until telediagnosis was available, you could only be screened in an ophthalmologist's office with a dilated retinal exam.

Please give your best answer to the following questions

Circle where you expect the quality of the retinal exam to be better:

1 2 3 4 5

Ophthalmologist's office ______ Telediagnosis

Circle which retinal exam you prefer:

1 2 3 4 5

Ophthalmologist's office ______ Telediagnosis

- □ yes ☑ no Do you expect the camera flashlight to bother you
- □ yes ☑ no Do you expect the pupil dilating drops to bother you
- ☑ yes □ no In the past five years have you been examined by an ophthalmologist
- ☑ yes □ no Do you currently carry health insurance
- ☑ yes □ no In some cases the photographs can only be made if your pupils are dilated with a drop. Were you aware of this?

Patient questionnaire

AFTER telediagnosis results have been discussed

Name:______________________ **Date:**____________

Circle your satisfaction with digital photography of your retina

1 2 3 4 5

Terrible Very good

Circle where you think the retinal exam is better:

1 2 3 4 5

Ophthalmologist's office ______ Telediagnosis

Circle which retinal exam you prefer now:

1 2 3 4 5

Ophthalmologist's office ______ Telediagnosis

- □ yes ☑ no Did the camera flashlight bother you
- □ yes ☑ no Did the pupil dilating drops bother you

After form is completed, please enter into Excel spreadsheet or send to:

Dr. Michael D. Abramoff, MD, PhD
Department of Ophthalmology and Visual Sciences
PFP 11290C
University of Iowa Hospitals and Clinics
200 Hawkins, Iowa City, IA 52242
Tel (319) 384 5833, fax: (319) 353 7996

[redacted]

[redacted]

Patient questionnaire

BEFORE telediagnosis

Name:_[redacted]

Thank you for your willingness to participate in this research project.

You are about to undergo digital photographing and diagnosis over the internet by ophthalmologists who are retinal specialists at the University of Iowa. They will evaluate your photographs for signs of diabetic retinopathy, a complication of diabetes. This new technique is called 'telediagnosis'. Until telediagnosis was available, you could only be screened in an ophthalmologist's office with a dilated retinal exam.

Please give your best answer to the following questions

Circle where you expect the quality of the retinal exam to be better:

1 Ophthalmologist's office 2 3 4 Telediagnosis (5)

Circle which retinal exam you prefer:

1 Ophthalmologist's office 2 3 4 Telediagnosis (5)

- ☐ yes ☒ no Do you expect the camera flashlight to bother you
- ☐ yes ☒ no Do you expect the pupil dilating drops to bother you
- ☒ yes ☐ no In the past five years have you been examined by an ophthalmologist
- ☒ yes ☐ no Do you currently carry health insurance
- ☒ yes ☐ no In some cases the photographs can only be made if your pupils are dilated with a drop. Were you aware of this?

Patient questionnaire

AFTER telediagnosis results have been discussed

Name:________________ **Date:**__________

Circle your satisfaction with digital photography of your retina

1 Terrible 2 3 4 (5) Very good

Circle where you think the retinal exam is better:

1 Ophthalmologist's office 2 3 4 (5) Telediagnosis

Circle which retinal exam you prefer now:

1 Ophthalmologist's office 2 3 4 (5) Telediagnosis

- ☐ yes ☒ no Did the camera flashlight bother you
- ☐ yes ☒ no Did the pupil dilating drops bother you

After form is completed, please enter into Excel spreadsheet or send to:

Dr. Michael D. Abramoff, MD, PhD
Department of Ophthalmology and Visual Sciences
PFP 11290C
University of Iowa Hospitals and Clinics
200 Hawkins, Iowa City, IA 52242
Tel (319) 384 5833, fax: (319) 353 7996

[redacted]

[redacted]

Patient Questionnaire

Name[redacted] Date[redacted]

BEF[redacted] telediagnosis

Thank you for your willingness to participate in this research project.

You are about to undergo digital photographing and diagnosis over the internet by ophthalmologists who are retinal specialists at the University of Iowa. They will evaluate your photographs for signs of diabetic retinopathy, a complication of diabetes. This new technique is called 'telediagnosis'. Until telediagnosis was available, you could only be screened in an ophthalmologist's office with a dilated retinal exam. Please give your best answer.

What number best expresses where you think the *quality* of the retinal exam is best:

1	2	3	4	5
(Better in ophthalmologist's office)		**3** undecided		(Better through Telediagnosis)

What number best expresses *where* you prefer to have the retinal exam:

1	2	3	4	5
(Prefer ophthalmologist's office)		**3** undecided		(Prefer family physician)

☐ yes ☒ no **Do you expect the camera flashlight to bother you?**

☐ yes ☒ no **Do you expect the pupil dilating drops to bother you?**

☐ yes ☒ no **In the past five years have you been examined by an ophthalmologist?**

☒ yes ☐ no **Do you currently carry health insurance?**

☒ yes ☐ no **Sometimes photographs can only be made if your pupils are made larger with a drop. Were you aware of this?**

AFTER telediagnosis

What number best expresses your *satisfaction* with telediagnosis:

1	2	3	4	5
(Terrible)		undecided		**5** (Very good)

What number best expresses your where you think the *quality* of the retinal exam is best::

1	2	3	4	5
(Better in ophthalmologist's office)		**3** undecided		(Better through Telediagnosis)

What number best expresses *where* you prefer to have the retinal exam now:

1	2	3	4	5
(Prefer ophthalmologist's office)		undecided		**5** (Prefer family physician)

☐ yes ☒ no **Did the camera flashlight bother you?**

☐ yes ☒ no **Did the pupil dilating drops bother you?** ☐ not applicable

[redacted]

Patient Questionnaire

Name [redacted] Date [redacted]

BEFORE telediagnosis

Thank you for your willingness to participate in this research project.

You are about to undergo digital photographing and diagnosis over the internet by ophthalmologists who are retinal specialists at the University of Iowa. They will evaluate your photographs for signs of diabetic retinopathy, a complication of diabetes. This new technique is called 'telediagnosis'. Until telediagnosis was available, you could only be screened in an ophthalmologist's office with a dilated retinal exam. Please give your best answer.

What number best expresses where you think the *quality* of the retinal exam is best:

1	2	3	4	5
(Better in ophthalmologist's office)		undecided		(Better through Telediagnosis)

What number best expresses *where* you prefer to have the retinal exam:

1	2	3	4	5
(Prefer ophthalmologist's office)		undecided		(Prefer family physician)

☐ yes ☒ no **Do you expect the camera flashlight to bother you?**

☐ yes ☒ no **Do you expect the pupil dilating drops to bother you?**

☐ yes ☒ no **In the past five years have you been examined by an ophthalmologist?**

☒ yes ☐ no **Do you currently carry health insurance?**

☐ yes ☒ no **Sometimes photographs can only be made if your pupils are made larger with a drop. Were you aware of this?**

AFTER telediagnosis

What number best expresses your *satisfaction* with telediagnosis:

1	2	3	4	5
(Terrible)		undecided		(Very good)

What number best expresses your where you think the *quality* of the retinal exam is best::

1	2	3	4	5
(Better in ophthalmologist's office)		undecided		(Better through Telediagnosis)

What number best expresses *where* you prefer to have the retinal exam <u>now</u>:

1	2	3	4	5
(Prefer ophthalmologist's office)		undecided		(Prefer family physician)

☐ yes ☒ no **Did the camera flashlight bother you?**

☐ yes ☐ no **Did the pupil dilating drops bother you?** ☒ not applicable

[redacted]

[redacted]

Patient Questionnaire

Name [redacted]________ Date_[redacted]

BEFORE telediagnosis

Thank you for your willingness to participate in this research project.

You are about to undergo digital photographing and diagnosis over the internet by ophthalmologists who are retinal specialists at the University of Iowa. They will evaluate your photographs for signs of diabetic retinopathy, a complication of diabetes. This new technique is called 'telediagnosis'. Until telediagnosis was available, you could only be screened in an ophthalmologist's office with a dilated retinal exam. Please give your best answer.

What number best expresses where you think the *quality* of the retinal exam is best:

1	2	3	4	5
(Better in ophthalmologist's office)		undecided		(Better through Telediagnosis)

What number best expresses *where* you prefer to have the retinal exam:

1	2	3	4	5
(Prefer ophthalmologist's office)		undecided		(Prefer family physician)

☐ yes ☒ no **Do you expect the camera flashlight to bother you?**

☐ yes ☒ no **Do you expect the pupil dilating drops to bother you?**

☒ yes ☐ no **In the past five years have you been examined by an ophthalmologist?**

☒ yes ☒ no **Do you currently carry health insurance?** [redacted]

☒ yes ☐no **Sometimes photographs can only be made if your pupils are made larger with a drop. Were you aware of this?**

AFTER telediagnosis

What number best expresses your *satisfaction* with telediagnosis:

1	2	3	4	5
(Terrible)		undecided		(Very good)

What number best expresses your where you think the *quality* of the retinal exam is best::

1	2	3	4	5
(Better in ophthalmologist's office)		undecided		(Better through Telediagnosis)

What number best expresses *where* you prefer to have the retinal exam <u>now</u>:

1	2	3	4	5
(Prefer ophthalmologist's office)		undecided		(Prefer family physician)

☐ yes ☒ no **Did the camera flashlight bother you?**

☐ yes ☒ no **Did the pupil dilating drops bother you?** ☐ not applicable

[redacted]

[redacted]

[redacted]

[redacted]nt Questionnaire

Name [redacted] Date [redacted]

BEFORE telediagnosis

Thank you for your willingness to participate in this research project.

You are about to undergo digital photographing and diagnosis over the internet by ophthalmologists who are retinal specialists at the University of Iowa. They will evaluate your photographs for signs of diabetic retinopathy, a complication of diabetes. This new technique is called 'telediagnosis'. Until telediagnosis was available, you could only be screened in an ophthalmologist's office with a dilated retinal exam. Please give your best answer.

What number best expresses where you think the *quality* of the retinal exam is best:

1 (Better in ophthalmologist's office)	2	(3) undecided	4	5 (Better through Telediagnosis)

What number best expresses *where* you prefer to have the retinal exam:

1 (Prefer ophthalmologist's office)	2	3 undecided	(4)	(5) (Prefer family physician)

☐ yes ☒ no **Do you expect the camera flashlight to bother you?**

☐ yes ☒ no **Do you expect the pupil dilating drops to bother you?**

☒ yes ☐ no **In the past five years have you been examined by an ophthalmologist?**

☒ yes ☐ no **Do you currently carry health insurance?**

☒ yes ☐ no **Sometimes photographs can only be made if your pupils are made larger with a drop. Were you aware of this?**

AFTER telediagnosis

What number best expresses your *satisfaction* with telediagnosis:

1 (Terrible)	2	3 undecided	4	(5) (Very good)

What number best expresses your where you think the *quality* of the retinal exam is best::

1 (Better in ophthalmologist's office)	2	3 undecided	4	(5) (Better through Telediagnosis)

What number best expresses *where* you prefer to have the retinal exam now:

1 (Prefer ophthalmologist's office)	2	3 undecided	4	(5) (Prefer family physician)

☐ yes ☒ no **Did the camera flashlight bother you?**

☐ yes ☒ no **Did the pupil dilating drops bother you?** ☐ not applicable

[redacted]

[redacted]

Patient Questionnaire

Name [redacted] Date [redacted]

BEFORE telediagnosis

Thank you for your willingness to participate in this research project.

You are about to undergo digital photographing and diagnosis over the internet by ophthalmologists who are retinal specialists at the University of Iowa. They will evaluate your photographs for signs of diabetic retinopathy, a complication of diabetes. This new technique is called 'telediagnosis'. Until telediagnosis was available, you could only be screened in an ophthalmologist's office with a dilated retinal exam. Please give your best answer.

What number best expresses where you think the *quality* of the retinal exam is best:

1	2	3	4	5
(Better in ophthalmologist's office)		undecided (circled: **3**)		(Better through Telediagnosis)

What number best expresses *where* you prefer to have the retinal exam:

1	2	3	4	5
(Prefer ophthalmologist's office)		undecided (circled: **3**)		(Prefer family physician)

☐ yes ☒ no **Do you expect the camera flashlight to bother you?**

☐ yes ☒ no **Do you expect the pupil dilating drops to bother you?**

☐ yes ☒ no **In the past five years have you been examined by an ophthalmologist?**

☒ yes ☐ no **Do you currently carry health insurance?**

☒ yes ☐ no **Sometimes photographs can only be made if your pupils are made larger with a drop. Were you aware of this?**

AFTER telediagnosis

What number best expresses your *satisfaction* with telediagnosis:

1	2	3	4	5
(Terrible)		undecided	(circled: **4**)	(Very good)

What number best expresses your where you think the *quality* of the retinal exam is best::

1	2	3	4	5
(Better in ophthalmologist's office)		undecided (circled: **3**)		(Better through Telediagnosis)

What number best expresses *where* you prefer to have the retinal exam <u>now</u>:

1	2	3	4	5
(Prefer ophthalmologist's office)		undecided (circled: **3**)		(Prefer family physician)

☐ yes ☒ no **Did the camera flashlight bother you?**

☐ yes ☐ no **Did the pupil dilating drops bother you?** ☒ not applicable

[redacted]

[redacted]

[redacted]

Patient Questionnaire

Name [redacted] Date [redacted]

BEFORE telediagnosis

Thank you for your willingness to participate in this research project.

You are about to undergo digital photographing and diagnosis over the internet by ophthalmologists who are retinal specialists at the University of Iowa. They will evaluate your photographs for signs of diabetic retinopathy, a complication of diabetes. This new technique is called 'telediagnosis'. Until telediagnosis was available, you could only be screened in an ophthalmologist's office with a dilated retinal exam. Please give your best answer.

What number best expresses where you think the *quality* of the retinal exam is best:

1	2	3	4	5
(Better in ophthalmologist's office)		undecided		(Better through Telediagnosis)

What number best expresses *where* you prefer to have the retinal exam:

1	2	3	4	5
(Prefer ophthalmologist's office)		undecided		(Prefer family physician)

☐ yes ☒ no **Do you expect the camera flashlight to bother you?**

☒ yes ☐ no **Do you expect the pupil dilating drops to bother you?**

☒ yes ☐ no **In the past five years have you been examined by an ophthalmologist?**

☒ yes ☐ no **Do you currently carry health insurance?**

☒ yes ☐ no **Sometimes photographs can only be made if your pupils are made larger with a drop. Were you aware of this?**

AFTER telediagnosis

What number best expresses your *satisfaction* with telediagnosis:

1	2	3	4	5
(Terrible)		undecided		(Very good)

What number best expresses your where you think the *quality* of the retinal exam is best::

1	2	3	4	5
(Better in ophthalmologist's office)		undecided		(Better through Telediagnosis)

What number best expresses *where* you prefer to have the retinal exam now:

1	2	3	4	5
(Prefer ophthalmologist's office)		undecided		(Prefer family physician)

☐ yes ☒ no **Did the camera flashlight bother you?**

☐ yes ☒ no **Did the pupil dilating drops bother you?** ☐ not applicable

[redacted]

[redacted]

Patient Questionnaire

Name [redacted] Date [redacted]

BEFORE telediagnosis

Thank you for your willingness to participate in this research project.

You are about to undergo digital photographing and diagnosis over the internet by ophthalmologists who are retinal specialists at the University of Iowa. They will evaluate your photographs for signs of diabetic retinopathy, a complication of diabetes. This new technique is called 'telediagnosis'. Until telediagnosis was available, you could only be screened in an ophthalmologist's office with a dilated retinal exam. Please give your best answer.

What number best expresses where you think the *quality* of the retinal exam is best:

1	2	3	4	5
(Better in ophthalmologist's office)		undecided		(Better through Telediagnosis)

(3 circled)

What number best expresses *where* you prefer to have the retinal exam:

1	2	3	4	5
(Prefer ophthalmologist's office)		undecided		(Prefer family physician)

(5 circled)

☐ yes ☒ no **Do you expect the camera flashlight to bother you?**

☐ yes ☒ no **Do you expect the pupil dilating drops to bother you?**

☒ yes ☐ no **In the past five years have you been examined by an ophthalmologist?**

☒ yes ☐ no **Do you currently carry health insurance?**

☐ yes ☒ no **Sometimes photographs can only be made if your pupils are made larger with a drop. Were you aware of this?**

AFTER telediagnosis

What number best expresses your *satisfaction* with telediagnosis:

1	2	3	4	5
(Terrible)		undecided		(Very good)

(5 circled)

What number best expresses your where you think the *quality* of the retinal exam is best::

1	2	3	4	5
(Better in ophthalmologist's office)		undecided		(Better through Telediagnosis)

(1 circled)

What number best expresses *where* you prefer to have the retinal exam <u>now</u>:

1	2	3	4	5
(Prefer ophthalmologist's office)		undecided		(Prefer family physician)

(5 circled)

☐ yes ☒ no **Did the camera flashlight bother you?**

☐ yes ☒ no **Did the pupil dilating drops bother you?** ☐ not applicable

[redacted]

[redacted]

Patient Questionnaire

Name [redacted] Date [redacted]

BEFORE [redacted]telediagnosis

Thank you for your willingness to participate in this research project.

You are about to undergo digital photographing and diagnosis over the internet by ophthalmologists who are retinal specialists at the University of Iowa. They will evaluate your photographs for signs of diabetic retinopathy, a complication of diabetes. This new technique is called 'telediagnosis'. Until telediagnosis was available, you could only be screened in an ophthalmologist's office with a dilated retinal exam. Please give your best answer.

What number best expresses where you think the *quality* of the retinal exam is best:

1	2	3	4	5
(Better in ophthalmologist's office)		undecided		(Better through Telediagnosis)

What number best expresses *where* you prefer to have the retinal exam:

1	2	3	4	5
(Prefer ophthalmologist's office)		undecided		(Prefer family physician)

☐ yes ☒ no **Do you expect the camera flashlight to bother you?**

☐ yes ☒ no **Do you expect the pupil dilating drops to bother you?**

☐ yes ☒ no **In the past five years have you been examined by an ophthalmologist?**

☒ yes ☐ no **Do you currently carry health insurance?**

☒ yes ☐ no **Sometimes photographs can only be made if your pupils are made larger with a drop. Were you aware of this?**

AFTER telediagnosis

What number best expresses your *satisfaction* with telediagnosis:

1	2	3	4	5
(Terrible)		undecided		(Very good)

What number best expresses your where you think the *quality* of the retinal exam is best::

1	2	3	4	5
(Better in ophthalmologist's office)		undecided		(Better through Telediagnosis)

What number best expresses *where* you prefer to have the retinal exam now:

1	2	3	4	5
(Prefer ophthalmologist's office)		undecided		(Prefer family physician)

☐ yes ☒ no **Did the camera flashlight bother you?**

☐ yes ☐ no **Did the pupil dilating drops bother you?** ☒ not applicable

[redacted]

[redacted]

Patient Questionnaire

Name [redacted] **Date** [redacted]

BEFORE telediagnosis

Thank you for your willingness to participate in this research project.

You are about to undergo digital photographing and diagnosis over the internet by ophthalmologists who are retinal specialists at the University of Iowa. They will evaluate your photographs for signs of diabetic retinopathy, a complication of diabetes. This new technique is called 'telediagnosis'. Until telediagnosis was available, you could only be screened in an ophthalmologist's office with a dilated retinal exam. Please give your best answer.

What number best expresses where you think the *quality* of the retinal exam is best:

1	2	3	4	5
(Better in ophthalmologist's office)		undecided		(Better through Telediagnosis)

What number best expresses *where* you prefer to have the retinal exam:

1	2	3	4	5
(Prefer ophthalmologist's office)		undecided		(Prefer family physician)

☑ yes ☐ no **Do you expect the camera flashlight to bother you?**

☑ yes ☐ no **Do you expect the pupil dilating drops to bother you?**

☑ yes ☐ no **In the past five years have you been examined by an ophthalmologist?**

☑ yes ☐ no **Do you currently carry health insurance?**

☑ yes ☐ no **Sometimes photographs can only be made if your pupils are made larger with a drop. Were you aware of this?**

AFTER telediagnosis

What number best expresses your *satisfaction* with telediagnosis:

1	2	3	4	5
(Terrible)		undecided		(Very good)

What number best expresses your where you think the *quality* of the retinal exam is best::

1	2	3	4	5
(Better in ophthalmologist's office)		undecided		(Better through Telediagnosis)

What number best expresses *where* you prefer to have the retinal exam now:

1	2	3	4	5
(Prefer ophthalmologist's office)		undecided		(Prefer family physician)

☑ yes ☐ no **Did the camera flashlight bother you?**

☐ yes ☐ no **Did the pupil dilating drops bother you?** ☑ not applicable

[redacted]

[redacted]

[redacted] Questionnaire

Name[redacted] Date[redacted]

BEFORE telediagnosis

Thank you for your willingness to participate in this research project.

You are about to undergo digital photographing and diagnosis over the internet by ophthalmologists who are retinal specialists at the University of Iowa. They will evaluate your photographs for signs of diabetic retinopathy, a complication of diabetes. This new technique is called 'telediagnosis'. Until telediagnosis was available, you could only be screened in an ophthalmologist's office with a dilated retinal exam. Please give your best answer.

What number best expresses where you think the *quality* of the retinal exam is best:

1	2	3 (circled)	4	5
(Better in ophthalmologist's office)		undecided		(Better through Telediagnosis)

What number best expresses *where* you prefer to have the retinal exam:

1	2	3 (circled)	4	5
(Prefer ophthalmologist's office)		undecided		(Prefer family physician)

☒ yes ☐ no **Do you expect the camera flashlight to bother you?**

☐ yes ☒ no **Do you expect the pupil dilating drops to bother you?**

☐ yes ☒ no **In the past five years have you been examined by an ophthalmologist?**

☒ yes ☐ no **Do you currently carry health insurance?**

☒ yes ☐ no **Sometimes photographs can only be made if your pupils are made larger with a drop. Were you aware of this?**

AFTER telediagnosis

What number best expresses your *satisfaction* with telediagnosis:

1	2	3	4	5 (circled)
(Terrible)		undecided		(Very good)

What number best expresses your where you think the *quality* of the retinal exam is best::

1	2	3 (circled)	4	5
(Better in ophthalmologist's office)		undecided		(Better through Telediagnosis)

What number best expresses *where* you prefer to have the retinal exam now:

1	2	3	4 (circled)	5
(Prefer ophthalmologist's office)		undecided		(Prefer family physician)

☐ yes ☑ no **Did the camera flashlight bother you?**

☐ yes ☐ no **Did the pupil dilating drops bother you?** ☑ not applicable

[redacted]

[redacted]

[redacted]

Patient Questionnaire

Name [redacted] Date [redacted]

BEFORE telediagnosis

Thank you for your willingness to participate in this research project.

You are about to undergo digital photographing and diagnosis over the internet by ophthalmologists who are retinal specialists at the University of Iowa. They will evaluate your photographs for signs of diabetic retinopathy, a complication of diabetes. This new technique is called 'telediagnosis'. Until telediagnosis was available, you could only be screened in an ophthalmologist's office with a dilated retinal exam. Please give your best answer.

What number best expresses where you think the *quality* of the retinal exam is best:

1	2	3	4	5
(Better in ophthalmologist's office)		undecided		(Better through Telediagnosis)

What number best expresses *where* you prefer to have the retinal exam:

1	2	3	4	5
(Prefer ophthalmologist's office)		undecided		(Prefer family physician)

☒ yes ☐ no **Do you expect the camera flashlight to bother you?**

☒ yes ☐ no **Do you expect the pupil dilating drops to bother you?**

☐ yes ☒ no **In the past five years have you been examined by an ophthalmologist?**

☒ yes ☐no **Do you currently carry health insurance?**

☒ yes ☐no **Sometimes photographs can only be made if your pupils are made larger with a drop. Were you aware of this?**

AFTER telediagnosis

What number best expresses your *satisfaction* with telediagnosis:

1	2	3	4	5
(Terrible)		undecided		(Very good)

What number best expresses your where you think the *quality* of the retinal exam is best::

1	2	3	4	5
(Better in ophthalmologist's office)		undecided		(Better through Telediagnosis)

What number best expresses *where* you prefer to have the retinal exam now:

1	2	3	4	5
(Prefer ophthalmologist's office)		undecided		(Prefer family physician)

☐ yes ☒ no **Did the camera flashlight bother you?**

☐ yes ☐ no **Did the pupil dilating drops bother you?** ☒ not applicable

[redacted]

[redacted]

[redacted]stionnaire

Name [redacted] Date [redacted]

BEFORE telediagnosis

Thank you for your willingness to participate in this research project.

You are about to undergo digital photographing and diagnosis over the internet by ophthalmologists who are retinal specialists at the University of Iowa. They will evaluate your photographs for signs of diabetic retinopathy, a complication of diabetes. This new technique is called 'telediagnosis'. Until telediagnosis was available, you could only be screened in an ophthalmologist's office with a dilated retinal exam. Please give your best answer.

What number best expresses where you think the *quality* of the retinal exam is best:

1	2	3	4	5
(Better in ophthalmologist's office)		undecided		(Better through Telediagnosis)

What number best expresses *where* you prefer to have the retinal exam:

1	2	3	4	5
(Prefer ophthalmologist's office)		undecided		(Prefer family physician)

☐ yes ☑ no **Do you expect the camera flashlight to bother you?**

☐ yes ☑ no **Do you expect the pupil dilating drops to bother you?**

☑ yes ☐ no **In the past five years have you been examined by an ophthalmologist?**

☑ yes ☐ no **Do you currently carry health insurance?**

☐ yes ☑ no **Sometimes photographs can only be made if your pupils are made larger with a drop. Were you aware of this?**

AFTER telediagnosis

What number best expresses your *satisfaction* with telediagnosis:

1	2	3	4	5
(Terrible)		undecided		(Very good)

What number best expresses your where you think the *quality* of the retinal exam is best::

1	2	3	4	5
(Better in ophthalmologist's office)		undecided		(Better through Telediagnosis)

What number best expresses *where* you prefer to have the retinal exam <u>now</u>:

1	2	3	4	5
(Prefer ophthalmologist's office)		undecided		(Prefer family physician)

☐ yes ☑ no **Did the camera flashlight bother you?**

☐ yes ☐ no **Did the pupil dilating drops bother you?** ☑ not applicable

[redacted]

[redacted]

[redacted]tient questionnaire
[redacted]EFORE telediagnosis

Name:[redacted] **Date:**[redacted]

Thank you for your willingness to participate in this research project.

You are about to undergo digital photographing and diagnosis over the internet by ophthalmologists who are retinal specialists at the University of Iowa. They will evaluate your photographs for signs of diabetic retinopathy, a complication of diabetes. This new technique is called 'telediagnosis'. Until telediagnosis was available, you could only be screened in an ophthalmologist's office with a dilated retinal exam.

Please give your best answer to the following questions

Circle where you expect the quality of the retinal exam to be better:

1 2 (3) 4 5

Ophthalmologist's office — Telediagnosis

Circle which retinal exam you prefer:

1 2 (3) 4 5

Ophthalmologist's office — Telediagnosis

□ yes ☒ no Do you expect the camera flashlight to bother you
□ yes ☒ no Do you expect the pupil dilating drops to bother you
☒ yes □ no In the past five years have you been examined by an ophthalmologist
☒ yes □ no Do you currently carry health insurance
☒ yes □ no In some cases the photographs can only be made if your pupils are dilated with a drop. Were you aware of this?

Patient questionnaire
AFTER telediagnosis results have been discussed

Name:____________________ **Date:**____________

Circle your satisfaction with digital photography of your retina

1 2 3 4 (5)

Terrible — Very good

Circle where you think the retinal exam is better:

1 2 (3) 4 5

Ophthalmologist's office — Telediagnosis

Circle which retinal exam you prefer now:

1 2 (3) 4 5

Ophthalmologist's office — Telediagnosis

□ yes ☒ no Did the camera flashlight bother you
□ yes ☒ no Did the pupil dilating drops bother you

After form is completed, please enter into Excel spreadsheet or send to:

Dr. Michael D. Abramoff, MD, PhD
Department of Ophthalmology and Visual Sciences
PFP 11290C
University of Iowa Hospitals and Clinics
200 Hawkins, Iowa City, IA 52242
Tel (319) 384 5833, fax: (319) 353 7996

[redacted]

Patient questionnaire

[redacted]

Name: [redacted]

Thank you for your willingness to participate in this research project/
You are about to undergo digital photographing and diagnosis over the internet by ophthalmologists who are retinal specialists at the University of Iowa. They will evaluate your photographs for signs of diabetic retinopathy, a complication of diabetes. This new technique is called 'telediagnosis'. Until telediagnosis was available, you could only be screened in an ophthalmologist's office with a dilated retinal exam.

Please give your best answer to the following questions

Circle where you expect the quality of the retinal exam to be better:

1	2	3	4	5
Ophthalmologist's office			Telediagnosis	

Circle which retinal exam you prefer:

1	2	3	4	5
Ophthalmologist's office			Telediagnosis	

☒ yes □ no Do you expect the camera flashlight to bother you
☒ yes □ no Do you expect the pupil dilating drops to bother you
☒ yes □ no In the past five years have you been examined by an ophthalmologist
☒ yes □ no Do you currently carry health insurance
☒ yes □ no In some cases the photographs can only be made if your pupils are dilated with a drop. Were you aware of this?

Patient questionnaire

AFTER telediagnosis results have been discussed

Name:______________________________ **Date:**_______________

Circle your satisfaction with digital photography of your retina

1	2	3	4	5
Terrible				Very good

Circle where you think the retinal exam is better:

1	2	3	4	5
Ophthalmologist's office				Telediagnosis

Circle which retinal exam you prefer now:

1	2	3	4	5
Ophthalmologist's office				Telediagnosis

□ yes ☒ no Did the camera flashlight bother you
□ yes ☒ no Did the pupil dilating drops bother you

After form is completed, please enter into Excel spreadsheet or send to:

Dr. Michael D. Abramoff, MD, PhD
Department of Ophthalmology and Visual Sciences
PFP 11290C
University of Iowa Hospitals and Clinics
200 Hawkins, Iowa City, IA 52242
Tel (319) 384 5833, fax: (319) 353 7996

[redacted]

[redacted]

[redacted]

[redacted]onnaire

Name [redacted] **Date** [redacted]

BEFORE telediagnosis

Thank you for your willingness to participate in this research project.

You are about to undergo digital photographing and diagnosis over the internet by ophthalmologists who are retinal specialists at the University of Iowa. They will evaluate your photographs for signs of diabetic retinopathy, a complication of diabetes. This new technique is called 'telediagnosis'. Until telediagnosis was available, you could only be screened in an ophthalmologist's office with a dilated retinal exam. Please give your best answer.

What number best expresses where you think the *quality* of the retinal exam is best:

1<br>(Better in ophthalmologist's office)	2	3<br>undecided	4	5<br>(Better through Telediagnosis)

What number best expresses *where* you prefer to have the retinal exam:

1<br>(Prefer ophthalmologist's office)	2	3<br>undecided	4	5<br>(Prefer family physician)

☐ yes ☒ no **Do you expect the camera flashlight to bother you?**

☐ yes ☒ no **Do you expect the pupil dilating drops to bother you?**

☐ yes ☒ no **In the past five years have you been examined by an ophthalmologist?**

☒ yes ☐ no **Do you currently carry health insurance?**

☒ yes ☐ no **Sometimes photographs can only be made if your pupils are made larger with a drop. Were you aware of this?**

AFTER telediagnosis

What number best expresses your *satisfaction* with telediagnosis:

1<br>(Terrible)	2	3<br>undecided	4	5<br>(Very good)

What number best expresses your where you think the *quality* of the retinal exam is best::

1<br>(Better in ophthalmologist's office)	2	3<br>undecided	4	5<br>(Better through Telediagnosis)

What number best expresses *where* you prefer to have the retinal exam now:

1<br>(Prefer ophthalmologist's office)	2	3<br>undecided	4	5<br>(Prefer family physician)

☐ yes ☒ no **Did the camera flashlight bother you?**

☐ yes ☒ no **Did the pupil dilating drops bother you?** ☐ not applicable

[redacted]

[redacted]

[redacted]

Patient Questionnaire

Name [redacted] Date [redacted]

BEFORE telediagnosis

Thank you for your willingness to participate in this research project.

You are about to undergo digital photographing and diagnosis over the internet by ophthalmologists who are retinal specialists at the University of Iowa. They will evaluate your photographs for signs of diabetic retinopathy, a complication of diabetes. This new technique is called 'telediagnosis'. Until telediagnosis was available, you could only be screened in an ophthalmologist's office with a dilated retinal exam. Please give your best answer.

What number best expresses where you think the *quality* of the retinal exam is best:

1	2	3	4	5
(Better in ophthalmologist's office)		undecided		(Better through Telediagnosis)

What number best expresses *where* you prefer to have the retinal exam:

1	2	3	4	5
(Prefer ophthalmologist's office)		undecided		(Prefer family physician)

☐ yes ☒ no **Do you expect the camera flashlight to bother you?**

☐ yes ☒ no **Do you expect the pupil dilating drops to bother you?**

☒ yes ☐ no **In the past five years have you been examined by an ophthalmologist?**

☒ yes ☐ no **Do you currently carry health insurance?**

☒ yes ☐ no **Sometimes photographs can only be made if your pupils are made larger with a drop. Were you aware of this?**

AFTER telediagnosis

What number best expresses your *satisfaction* with telediagnosis:

1	2	3	4	5
(Terrible)		undecided		(Very good)

What number best expresses your where you think the *quality* of the retinal exam is best::

1	2	3	4	5
(Better in ophthalmologist's office)		undecided		(Better through Telediagnosis)

What number best expresses *where* you prefer to have the retinal exam now:

1	2	3	4	5
(Prefer ophthalmologist's office)		undecided		(Prefer family physician)

☐ yes ☐ no **Did the camera flashlight bother you?**

☐ yes ☐ no **Did the pupil dilating drops bother you?** ☐ not applicable

[redacted]

[redacted]

Patient Questionnaire

Name [redacted] **Date** [redacted]

BEFORE telediagnosis [redacted]

Thank you for your willingness to participate in this research project.

You are about to undergo digital photographing and diagnosis over the internet by ophthalmologists who are retinal specialists at the University of Iowa. They will evaluate your photographs for signs of diabetic retinopathy, a complication of diabetes. This new technique is called 'telediagnosis'. Until telediagnosis was available, you could only be screened in an ophthalmologist's office with a dilated retinal exam. Please give your best answer.

What number best expresses where you think the *quality* of the retinal exam is best:

1	2	3	4	5
(Better in ophthalmologist's office)		undecided		(Better through Telediagnosis)

What number best expresses *where* you prefer to have the retinal exam:

1	2	3	4	5
(Prefer ophthalmologist's office)		undecided		(Prefer family physician)

☐ yes ☐ no **Do you expect the camera flashlight to bother you?**

☐ yes ☐ no **Do you expect the pupil dilating drops to bother you?**

☐ yes ☐ no **In the past five years have you been examined by an ophthalmologist?**

☐ yes ☐no **Do you currently carry health insurance?**

☐ yes ☐no **Sometimes photographs can only be made if your pupils are made larger with a drop. Were you aware of this?**

AFTER telediagnosis

What number best expresses your *satisfaction* with telediagnosis:

1	2	3	4	5
(Terrible)		undecided		(Very good)

What number best expresses your where you think the *quality* of the retinal exam is best::

1	2	3	4	5
(Better in ophthalmologist's office)		undecided		(Better through Telediagnosis)

What number best expresses *where* you prefer to have the retinal exam <u>now</u>:

1	2	3	4	5
(Prefer ophthalmologist's office)		undecided		(Prefer family physician)

☐ yes ☐ no **Did the camera flashlight bother you?**

☐ yes ☐ no **Did the pupil dilating drops bother you?** ☐ not applicable

[redacted]

[redacted]

Patient Questionnaire

Name [redacted] Date [redacted]

BEFORE telediagnosis

Thank you for your willingness to participate in this research project.

You are about to undergo digital photographing and diagnosis over the internet by ophthalmologists who are retinal specialists at the University of Iowa. They will evaluate your photographs for signs of diabetic retinopathy, a complication of diabetes. This new technique is called 'telediagnosis'. Until telediagnosis was available, you could only be screened in an ophthalmologist's office with a dilated retinal exam. Please give your best answer.

What number best expresses where you think the *quality* of the retinal exam is best:

1	2	3	4	5
(Better in ophthalmologist's office)		undecided		(Better through Telediagnosis)

What number best expresses *where* you prefer to have the retinal exam:

1	2	3	4	5
(Prefer ophthalmologist's office)		undecided		(Prefer family physician)

☐ yes ☐ no **Do you expect the camera flashlight to bother you?**

☐ yes ☐ no **Do you expect the pupil dilating drops to bother you?**

☐ yes ☐ no **In the past five years have you been examined by an ophthalmologist?**

☐ yes ☐ no **Do you currently carry health insurance?**

☐ yes ☐ no **Sometimes photographs can only be made if your pupils are made larger with a drop. Were you aware of this?**

AFTER telediagnosis

What number best expresses your *satisfaction* with telediagnosis:

1	2	3	4	5
(Terrible)		undecided		(Very good)

What number best expresses your where you think the *quality* of the retinal exam is best::

1	2	3	4	5
(Better in ophthalmologist's office)		undecided		(Better through Telediagnosis)

What number best expresses *where* you prefer to have the retinal exam now:

1	2	3	4	5
(Prefer ophthalmologist's office)		undecided		(Prefer family physician)

☐ yes ☐ no **Did the camera flashlight bother you?**

☐ yes ☐ no **Did the pupil dilating drops bother you?** ☐ not applicable

[redacted]

[redacted]

[redacted]

Patient questionnaire

BEFORE telediagnosis

Name:[redacted]

Thank you for your willingness to participate in this research project.

You are about to undergo digital photographing and diagnosis over the internet by ophthalmologists who are retinal specialists at the University of Iowa. They will evaluate your photographs for signs of diabetic retinopathy, a complication of diabetes. This new technique is called 'telediagnosis'. Until telediagnosis was available, you could only be screened in an ophthalmologist's office with a dilated retinal exam.

Please give your best answer to the following questions

Circle where you expect the quality of the retinal exam to be better:

1	2	3	4	5
Ophthalmologist's office			Telediagnosis	

Circle which retinal exam you prefer:

1	2	3	4	5
Ophthalmologist's office			Telediagnosis	

□ yes □ no Do you expect the camera flashlight to bother you
□ yes □ no Do you expect the pupil dilating drops to bother you
□ yes □ no In the past five years have you been examined by an ophthalmologist
□ yes □ no Do you currently carry health insurance
□ yes □ no In some cases the photographs can only be made if your pupils are dilated with a drop. Were you aware of this?

Patient questionnaire

AFTER telediagnosis results have been discussed

Name:______________________________ **Date:**_______________

Circle your satisfaction with digital photography of your retina

1	2	3	4	5
Terrible				Very good

Circle where you think the retinal exam is better:

1	2	3	4	5
Ophthalmologist's office				Telediagnosis

Circle which retinal exam you prefer now:

1	2	3	4	5
Ophthalmologist's office				Telediagnosis

□ yes □ no Did the camera flashlight bother you
□ yes □ no Did the pupil dilating drops bother you [redacted]

After form is completed, please enter into Excel spreadsheet or send to:

Dr. Michael D. Abramoff, MD, PhD
Department of Ophthalmology and Visual Sciences
PFP 11290C
University of Iowa Hospitals and Clinics
200 Hawkins, Iowa City, IA 52242
Tel (319) 384 5833, fax: (319) 353 7996

[redacted]

[redacted]

Patient Questionnaire

Name [redacted] Date [redacted]

BEFORE telediagnosis

Thank you for your willingness to participate in this research project.

You are about to undergo digital photographing and diagnosis over the internet by ophthalmologists who are retinal specialists at the University of Iowa. They will evaluate your photographs for signs of diabetic retinopathy, a complication of diabetes. This new technique is called 'telediagnosis'. Until telediagnosis was available, you could only be screened in an ophthalmologist's office with a dilated retinal exam. Please give your best answer.

What number best expresses where you think the *quality* of the retinal exam is best:

1	2	3	4	5
(Better in ophthalmologist's office)		undecided	(Better through Telediagnosis)	

What number best expresses *where* you prefer to have the retinal exam:

1	2	3	4	5
(Prefer ophthalmologist's office)		undecided	(Prefer family physician)	

☐ yes ☑ no **Do you expect the camera flashlight to bother you?**

☐ yes ☑ no **Do you expect the pupil dilating drops to bother you?**

☑ yes ☐ no **In the past five years have you been examined by an ophthalmologist?**

☑ yes ☐ no **Do you currently carry health insurance?**

☑ yes ☐ no **Sometimes photographs can only be made if your pupils are made larger with a drop. Were you aware of this?**

AFTER telediagnosis

What number best expresses your *satisfaction* with telediagnosis:

1	2	3	4	5
(Terrible)		undecided		(Very good)

What number best expresses your where you think the *quality* of the retinal exam is best::

1	2	3	4	5
(Better in ophthalmologist's office)		undecided	(Better through Telediagnosis)	

What number best expresses *where* you prefer to have the retinal exam now:

1	2	3	4	5
(Prefer ophthalmologist's office)		undecided	(Prefer family physician)	

☐ yes ☒ no **Did the camera flashlight bother you?**

☐ yes ☒ no **Did the pupil dilating drops bother you?** ☐ not applicable

[redacted]

[redacted]

Patient Questionnaire

Name [redacted] **Date** [redacted]

[redacted]

BEFORE telediagnosis

Thank you for your willingness to participate in this research project.

You are about to undergo digital photographing and diagnosis over the internet by ophthalmologists who are retinal specialists at the University of Iowa. They will evaluate your photographs for signs of diabetic retinopathy, a complication of diabetes. This new technique is called 'telediagnosis'. Until telediagnosis was available, you could only be screened in an ophthalmologist's office with a dilated retinal exam. Please give your best answer.

What number best expresses where you think the *quality* of the retinal exam is best:

1 (Better in ophthalmologist's office)	2	3 undecided	4	5 (Better through Telediagnosis)

What number best expresses *where* you prefer to have the retinal exam:

1 (Prefer ophthalmologist's office)	2	3 undecided	4	5 (Prefer family physician)

☐ yes ☑ no **Do you expect the camera flashlight to bother you?**

☐ yes ☑ no **Do you expect the pupil dilating drops to bother you?**

☐ yes ☑ no **In the past five years have you been examined by an ophthalmologist?**

☑ yes ☐ no **Do you currently carry health insurance?**

☐ yes ☑ no **Sometimes photographs can only be made if your pupils are made larger with a drop. Were you aware of this?**

AFTER telediagnosis

What number best expresses your *satisfaction* with telediagnosis:

1 (Terrible)	2	3 undecided	4	5 (Very good)

What number best expresses your where you think the *quality* of the retinal exam is best::

1 (Better in ophthalmologist's office)	2	3 undecided	4	5 (Better through Telediagnosis)

What number best expresses *where* you prefer to have the retinal exam now:

1 (Prefer ophthalmologist's office)	2	3 undecided	4	5 (Prefer family physician)

☐ yes ☑ no **Did the camera flashlight bother you?**

☐ yes ☐ no **Did the pupil dilating drops bother you?** ☑ not applicable

[redacted]

[redacted]

Patient Questionnaire

Name [redacted] Date [redacted]

BEFORE telediagnosis

Thank you for your willingness to participate in this research project.

You are about to undergo digital photographing and diagnosis over the internet by ophthalmologists who are retinal specialists at the University of Iowa. They will evaluate your photographs for signs of diabetic retinopathy, a complication of diabetes. This new technique is called 'telediagnosis'. Until telediagnosis was available, you could only be screened in an ophthalmologist's office with a dilated retinal exam. Please give your best answer.

What number best expresses where you think the *quality* of the retinal exam is best:

1	2	3	4	5
(Better in ophthalmologist's office)		undecided		(Better through Telediagnosis)

What number best expresses *where* you prefer to have the retinal exam:

1	2	3	4	5
(Prefer ophthalmologist's office)		undecided		(Prefer family physician)

☐ yes ☒ no **Do you expect the camera flashlight to bother you?**

☐ yes ☒ no **Do you expect the pupil dilating drops to bother you?**

☐ yes ☒ no **In the past five years have you been examined by an ophthalmologist?**

☒ yes ☐ no **Do you currently carry health insurance?**

☒ yes ☐ no **Sometimes photographs can only be made if your pupils are made larger with a drop. Were you aware of this?**

AFTER telediagnosis

What number best expresses your *satisfaction* with telediagnosis:

1	2	3	4	5
(Terrible)		undecided		(Very good)

What number best expresses your where you think the *quality* of the retinal exam is best::

1	2	3	4	5
(Better in ophthalmologist's office)		undecided		(Better through Telediagnosis)

What number best expresses *where* you prefer to have the retinal exam now:

1	2	3	4	5
(Prefer ophthalmologist's office)		undecided		(Prefer family physician)

☐ yes ☒ no **Did the camera flashlight bother you?**

[redacted] ☐ yes ☐ no **Did the pupil dilating drops bother you?** ☒ not applicable

[redacted]

[redacted]

Patient questionnaire

[redacted]

Name[redacted]

Thank you for your willingness to participate in this research project.
You are about to undergo digital photographing and diagnosis over the internet by ophthalmologists who are retinal specialists at the University of Iowa. They will evaluate your photographs for signs of diabetic retinopathy, a complication of diabetes. This new technique is called 'telediagnosis'. Until telediagnosis was available, you could only be screened in an ophthalmologist's office with a dilated retinal exam.

Please give your best answer to the following questions

Circle where you expect the quality of the retinal exam to be better:

1	2	(3)	4	5
Ophthalmologist's office			Telediagnosis	

Circle which retinal exam you prefer:

1	2	3	4	(5)
Ophthalmologist's office			Telediagnosis	

☐ yes ☒ no Do you expect the camera flashlight to bother you
☐ yes ☒ no Do you expect the pupil dilating drops to bother you
☐ yes ☒ no In the past five years have you been examined by an ophthalmologist
☒ yes ☐ no Do you currently carry health insurance
☒ yes ☐ no In some cases the photographs can only be made if your pupils are dilated with a drop. Were you aware of this?

Patient questionnaire

AFTER telediagnosis results have been discussed

Name:________________________ **Date:**____________

Circle your satisfaction with digital photography of your retina

1	2	3	4	(5)
Terrible				Very good

Circle where you think the retinal exam is better:

1	2	(3)	4	5
Ophthalmologist's office				Telediagnosis

Circle which retinal exam you prefer now:

1	2	3	4	(5)
Ophthalmologist's office				Telediagnosis

[redacted]
☐ yes ☒ no Did the camera flashlight bother you
☐ yes ☐ no Did the pupil dilating drops bother you

After form is completed, please enter into Excel spreadsheet or send to:

Dr. Michael D. Abramoff, MD, PhD
Department of Ophthalmology and Visual Sciences
PFP 11290C
University of Iowa Hospitals and Clinics
200 Hawkins, Iowa City, IA 52242
Tel (319) 384 5833, fax: (319) 353 7996

[redacted]

[redacted]

Patient Questionnaire

Name [redacted] **Date** [redacted]

BEFORE telediagnosis

Thank you for your willingness to participate in this research project.

You are about to undergo digital photographing and diagnosis over the internet by ophthalmologists who are retinal specialists at the University of Iowa. They will evaluate your photographs for signs of diabetic retinopathy, a complication of diabetes. This new technique is called 'telediagnosis'. Until telediagnosis was available, you could only be screened in an ophthalmologist's office with a dilated retinal exam. Please give your best answer.

What number best expresses where you think the *quality* of the retinal exam is best:

1	2	3	4	5
(Better in ophthalmologist's office)		undecided		(Better through Telediagnosis)

What number best expresses *where* you prefer to have the retinal exam:

1	2	3	4	5
(Prefer ophthalmologist's office)		undecided		(Prefer family physician)

☐ yes ☑ no **Do you expect the camera flashlight to bother you?**

☐ yes ☐ no **Do you expect the pupil dilating drops to bother you?**

☐ yes ☑ no **In the past five years have you been examined by an ophthalmologist?**

☑ yes ☐ no **Do you currently carry health insurance?**

☑ yes ☐ no **Sometimes photographs can only be made if your pupils are made larger with a drop. Were you aware of this?**

AFTER telediagnosis

What number best expresses your *satisfaction* with telediagnosis:

1	2	3	4	5
(Terrible)		undecided		(Very good)

What number best expresses your where you think the *quality* of the retinal exam is best::

1	2	3	4	5
(Better in ophthalmologist's office)		undecided		(Better through Telediagnosis)

What number best expresses *where* you prefer to have the retinal exam now:

1	2	3	4	5
(Prefer ophthalmologist's office)		undecided		(Prefer family physician)

☐ yes ☑ no **Did the camera flashlight bother you?**

☐ yes ☐ no **Did the pupil dilating drops bother you?** ☑ not applicable

[redacted]

[redacted]

Patient Questionnaire

Name [redacted]

Date [redacted]

BEFORE telediagnosis

Thank you for your willingness to participate in this research project.

You are about to undergo digital photographing and diagnosis over the internet by ophthalmologists who are retinal specialists at the University of Iowa. They will evaluate your photographs for signs of diabetic retinopathy, a complication of diabetes. This new technique is called 'telediagnosis'. Until telediagnosis was available, you could only be screened in an ophthalmologist's office with a dilated retinal exam. Please give your best answer.

What number best expresses where you think the *quality* of the retinal exam is best:

1	2	3	4	5
(Better in ophthalmologist's office)	(2) circled	undecided		(Better through Telediagnosis)

What number best expresses *where* you prefer to have the retinal exam:

1	2	3	4	5
(Prefer ophthalmologist's office)	(2) circled	undecided		(Prefer family physician)

☐ yes ☒ no **Do you expect the camera flashlight to bother you?**

☐ yes ☒ no **Do you expect the pupil dilating drops to bother you?**

☒ yes ☐ no **In the past five years have you been examined by an ophthalmologist?**

☒ yes ☐ no **Do you currently carry health insurance?**

☐ yes ☒ no **Sometimes photographs can only be made if your pupils are made larger with a drop. Were you aware of this?**

AFTER telediagnosis

What number best expresses your *satisfaction* with telediagnosis:

1	2	3	4	5
(Terrible)		undecided		(5) circled (Very good)

What number best expresses your where you think the *quality* of the retinal exam is best::

1	2	3	4	5
(Better in ophthalmologist's office)		undecided		(5) circled (Better through Telediagnosis)

What number best expresses *where* you prefer to have the retinal exam now:

1	2	3	4	5
(Prefer ophthalmologist's office)		undecided		(5) circled (Prefer family physician)

☐ yes ☒ no **Did the camera flashlight bother you?**

☐ yes ☐ no **Did the pupil dilating drops bother you?** ☒ not applicable

[redacted]

[redacted]

Patient Questionnaire

Name [redacted] Date [redacted]

BEFORE telediagnosis

Thank you for your willingness to participate in this research project.

You are about to undergo digital photographing and diagnosis over the internet by ophthalmologists who are retinal specialists at the University of Iowa. They will evaluate your photographs for signs of diabetic retinopathy, a complication of diabetes. This new technique is called 'telediagnosis'. Until telediagnosis was available, you could only be screened in an ophthalmologist's office with a dilated retinal exam. Please give your best answer.

What number best expresses where you think the *quality* of the retinal exam is best:

1	2	3	4	5
(Better in ophthalmologist's office)		undecided		(Better through Telediagnosis)

What number best expresses *where* you prefer to have the retinal exam:

1	2	3	4	5
(Prefer ophthalmologist's office)		undecided		(Prefer family physician)

☐ yes ☒ no **Do you expect the camera flashlight to bother you?**

☐ yes ☒ no **Do you expect the pupil dilating drops to bother you?**

☒ yes ☐ no **In the past five years have you been examined by an ophthalmologist?**

☒ yes ☐ no **Do you currently carry health insurance?**

☒ yes ☐ no **Sometimes photographs can only be made if your pupils are made larger with a drop. Were you aware of this?**

AFTER telediagnosis

What number best expresses your *satisfaction* with telediagnosis:

1	2	3	4	5
(Terrible)		undecided		(Very good)

What number best expresses your where you think the *quality* of the retinal exam is best::

1	2	3	4	5
(Better in ophthalmologist's office)		undecided		(Better through Telediagnosis)

What number best expresses *where* you prefer to have the retinal exam <u>now</u>:

1	2	3	4	5
(Prefer ophthalmologist's office)		undecided		(Prefer family physician)

☐ yes ☒ no **Did the camera flashlight bother you?**

☐ yes ☐ no **Did the pupil dilating drops bother you?** ☒ not applicable

[redacted]

Patient Questionnaire

Name [redacted] Date [redacted]

BEFORE telediagnosis

Thank you for your willingness to participate in this research project.

You are about to undergo digital photographing and diagnosis over the internet by ophthalmologists who are retinal specialists at the University of Iowa. They will evaluate your photographs for signs of diabetic retinopathy, a complication of diabetes. This new technique is called 'telediagnosis'. Until telediagnosis was available, you could only be screened in an ophthalmologist's office with a dilated retinal exam. Please give your best answer.

What number best expresses where you think the *quality* of the retinal exam is best:

1	2	3	4	(5)
(Better in ophthalmologist's office)		undecided		(Better through Telediagnosis)

What number best expresses *where* you prefer to have the retinal exam:

1	2	3	4	(5)
(Prefer ophthalmologist's office)		undecided		(Prefer family physician)

☐ yes ☑ no **Do you expect the camera flashlight to bother you?**

☐ yes ☑ no **Do you expect the pupil dilating drops to bother you?**

☐ yes ☒ no **In the past five years have you been examined by an ophthalmologist?**

☐ yes ☑ no **Do you currently carry health insurance?**

☒ yes ☐ no **Sometimes photographs can only be made if your pupils are made larger with a drop. Were you aware of this?**

AFTER telediagnosis

What number best expresses your *satisfaction* with telediagnosis:

1	2	3	4	(5)
(Terrible)		undecided		(Very good)

What number best expresses your where you think the *quality* of the retinal exam is best::

1	2	3	4	(5)
(Better in ophthalmologist's office)		undecided		(Better through Telediagnosis)

What number best expresses *where* you prefer to have the retinal exam <u>now</u>:

1	2	3	4	(5)
(Prefer ophthalmologist's office)		undecided		(Prefer family physician)

☐ yes ☒ no **Did the camera flashlight bother you?**

☐ yes ☐ no **Did the pupil dilating drops bother you?** ☑ not applicable

[redacted]naire

Name [redacted] Date [redacted]

BEFORE telediagnosis

Thank you for your willingness to participate in this research project.

You are about to undergo digital photographing and diagnosis over the internet by ophthalmologists who are retinal specialists at the University of Iowa. They will evaluate your photographs for signs of diabetic retinopathy, a complication of diabetes. This new technique is called 'telediagnosis'. Until telediagnosis was available, you could only be screened in an ophthalmologist's office with a dilated retinal exam. Please give your best answer.

What number best expresses where you think the *quality* of the retinal exam is best:

1	2	3	4	**5**
(Better in ophthalmologist's office)		undecided		(Better through Telediagnosis)

What number best expresses *where* you prefer to have the retinal exam:

1	2	3	4	**5**
(Prefer ophthalmologist's office)		undecided		(Prefer family physician)

☐ yes ☒ no **Do you expect the camera flashlight to bother you?**

☐ yes ☒ no **Do you expect the pupil dilating drops to bother you?**

☒ yes ☐ no **In the past five years have you been examined by an ophthalmologist?**

☒ yes ☐ no **Do you currently carry health insurance?**

☒ yes ☐ no **Sometimes photographs can only be made if your pupils are made larger with a drop. Were you aware of this?**

AFTER telediagnosis

What number best expresses your *satisfaction* with telediagnosis:

1	2	3	4	**5**
(Terrible)		undecided		(Very good)

What number best expresses your where you think the *quality* of the retinal exam is best::

1	2	3	4	**5**
(Better in ophthalmologist's office)		undecided		(Better through Telediagnosis)

What number best expresses *where* you prefer to have the retinal exam <u>now</u>:

1	2	3	4	**5**
(Prefer ophthalmologist's office)		undecided		(Prefer family physician)

☐ yes ☒ no **Did the camera flashlight bother you?**

☐ yes ☐ no **Did the pupil dilating drops bother you?** ☒ not applicable

[redacted]

[redacted]estionnaire

Name [redacted] Date [redacted]

BEFOR[redacted]

Thank you for your willingness to participate in this research project.

You are about to undergo digital photographing and diagnosis over the internet by ophthalmologists who are retinal specialists at the University of Iowa. They will evaluate your photographs for signs of diabetic retinopathy, a complication of diabetes. This new technique is called 'telediagnosis'. Until telediagnosis was available, you could only be screened in an ophthalmologist's office with a dilated retinal exam. Please give your best answer.

What number best expresses where you think the *quality* of the retinal exam is best:

1	2	3	4	5
(Better in ophthalmologist's office)		undecided		(Better through Telediagnosis)

What number best expresses *where* you prefer to have the retinal exam:

1	2	3	4	5
(Prefer ophthalmologist's office)		undecided		(Prefer family physician)

☐ yes ☐ no **Do you expect the camera flashlight to bother you?**

☐ yes ☐ no **Do you expect the pupil dilating drops to bother you?**

☐ yes ☐ no **In the past five years have you been examined by an ophthalmologist?**

☐ yes ☐ no **Do you currently carry health insurance?**

☐ yes ☐ no **Sometimes photographs can only be made if your pupils are made larger with a drop. Were you aware of this?**

AFTER telediagnosis

What number best expresses your *satisfaction* with telediagnosis:

1	2	3	4	5
(Terrible)		undecided		(Very good)

What number best expresses your where you think the *quality* of the retinal exam is best::

1	2	3	4	5
(Better in ophthalmologist's office)		undecided		(Better through Telediagnosis)

What number best expresses *where* you prefer to have the retinal exam <u>now</u>:

1	2	3	4	5
(Prefer ophthalmologist's office)		undecided		(Prefer family physician)

☐ yes ☐ no **Did the camera flashlight bother you?**

☐ yes ☐ no **Did the pupil dilating drops bother you?** ☐ not applicable

[redacted]

[redacted]

[redacted]

[redacted]onnaire

Name [redacted] Date [redacted]

BEFORE [redacted]

Thank you for your willingness to participate in this research project.

You are about to undergo digital photographing and diagnosis over the internet by ophthalmologists who are retinal specialists at the University of Iowa. They will evaluate your photographs for signs of diabetic retinopathy, a complication of diabetes. This new technique is called 'telediagnosis'. Until telediagnosis was available, you could only be screened in an ophthalmologist's office with a dilated retinal exam. Please give your best answer.

What number best expresses where you think the *quality* of the retinal exam is best:

1	2	3	4	5
(Better in ophthalmologist's office)		undecided		(Better through Telediagnosis)

What number best expresses *where* you prefer to have the retinal exam:

1	2	3	4	5
(Prefer ophthalmologist's office)		undecided		(Prefer family physician)

☐ yes ☑ no **Do you expect the camera flashlight to bother you?**

☐ yes ☑ no **Do you expect the pupil dilating drops to bother you?**

☐ yes ☑ no **In the past five years have you been examined by an ophthalmologist?**

☑ yes ☐ no **Do you currently carry health insurance?**

☑ yes ☐ no **Sometimes photographs can only be made if your pupils are made larger with a drop. Were you aware of this?**

AFTER telediagnosis

What number best expresses your *satisfaction* with telediagnosis:

1	2	3	4	5
(Terrible)		undecided		(Very good)

What number best expresses your where you think the *quality* of the retinal exam is best::

1	2	3	4	5
(Better in ophthalmologist's office)		undecided		(Better through Telediagnosis)

What number best expresses *where* you prefer to have the retinal exam now:

1	2	3	4	5
(Prefer ophthalmologist's office)		undecided		(Prefer family physician)

☐ yes ☑ no **Did the camera flashlight bother you?**

☐ yes ☐ no **Did the pupil dilating drops bother you?** ☑ not applicable

[redacted]

[redacted] t Questionnaire

Name [redacted] Date [redacted]

BEFORE telediagnosis

Thank you for your willingness to participate in this research project.

You are about to undergo digital photographing and diagnosis over the internet by ophthalmologists who are retinal specialists at the University of Iowa. They will evaluate your photographs for signs of diabetic retinopathy, a complication of diabetes. This new technique is called 'telediagnosis'. Until telediagnosis was available, you could only be screened in an ophthalmologist's office with a dilated retinal exam. Please give your best answer.

What number best expresses where you think the *quality* of the retinal exam is best:

1	2	3	4	5
(Better in ophthalmologist's office)		undecided		(Better through Telediagnosis)

What number best expresses *where* you prefer to have the retinal exam:

1	2	3	4	5
(Prefer ophthalmologist's office)		undecided		(Prefer family physician)

☐ yes ☒ no **Do you expect the camera flashlight to bother you?**

☐ yes ☒ no **Do you expect the pupil dilating drops to bother you?**

☐ yes ☒ no **In the past five years have you been examined by an ophthalmologist?**

☒ yes ☐ no **Do you currently carry health insurance?**

☒ yes ☐ no **Sometimes photographs can only be made if your pupils are made larger with a drop. Were you aware of this?**

AFTER telediagnosis

What number best expresses your *satisfaction* with telediagnosis:

1	2	3	4	5
(Terrible)		undecided		(Very good)

What number best expresses your where you think the *quality* of the retinal exam is best::

1	2	3	4	5
(Better in ophthalmologist's office)		undecided		(Better through Telediagnosis)

What number best expresses *where* you prefer to have the retinal exam now:

1	2	3	4	5
(Prefer ophthalmologist's office)		undecided		(Prefer family physician)

☐ yes ☒ no **Did the camera flashlight bother you?**

☒ yes ☐ no **Did the pupil dilating drops bother you?** ☐ not applicable

[redacted]

[redacted]

[redacted]

[redacted] Questionnaire

Name [redacted] Date [redacted]

BEFO[redacted]

Thank you for your willingness to participate in this research project.

You are about to undergo digital photographing and diagnosis over the internet by ophthalmologists who are retinal specialists at the University of Iowa. They will evaluate your photographs for signs of diabetic retinopathy, a complication of diabetes. This new technique is called 'telediagnosis'. Until telediagnosis was available, you could only be screened in an ophthalmologist's office with a dilated retinal exam. Please give your best answer.

What number best expresses where you think the *quality* of the retinal exam is best:

1	2	3	4	5
(Better in ophthalmologist's office)		undecided	(Better through Telediagnosis)	

What number best expresses *where* you prefer to have the retinal exam:

1	2	3	4	5
(Prefer ophthalmologist's office)		undecided	(Prefer family physician)	

☐ yes ☑ no **Do you expect the camera flashlight to bother you?**

☑ yes ☐ no **Do you expect the pupil dilating drops to bother you?**

☐ yes ☑ no **In the past five years have you been examined by an ophthalmologist?**

☑ yes ☐ no **Do you currently carry health insurance?**

☑ yes ☐ no **Sometimes photographs can only be made if your pupils are made larger with a drop. Were you aware of this?**

AFTER telediagnosis

What number best expresses your *satisfaction* with telediagnosis:

1	2	3	4	5
(Terrible)		undecided		**5** (Very good)

What number best expresses your where you think the *quality* of the retinal exam is best::

1	2	3	4	5
(Better in ophthalmologist's office)		undecided	(Better through Telediagnosis)	**5**

What number best expresses *where* you prefer to have the retinal exam <u>now</u>:

1	2	3	4	5
(Prefer ophthalmologist's office)		undecided	(Prefer family physician)	**5**

☐ yes ☑ no **Did the camera flashlight bother you?**

☐ yes ☐ no **Did the pupil dilating drops bother you?** ☑ not applicable

[redacted]

[redacted]

Patient Questionnaire

Name [redacted] Date [redacted]

BEFORE telediagnosis

Thank you for your willingness to participate in this research project.

You are about to undergo digital photographing and diagnosis over the internet by ophthalmologists who are retinal specialists at the University of Iowa. They will evaluate your photographs for signs of diabetic retinopathy, a complication of diabetes. This new technique is called 'telediagnosis'. Until telediagnosis was available, you could only be screened in an ophthalmologist's office with a dilated retinal exam. Please give your best answer.

What number best expresses where you think the *quality* of the retinal exam is best:

1	2	3	4	5 (circled)
(Better in ophthalmologist's office)		undecided		(Better through Telediagnosis)

What number best expresses *where* you prefer to have the retinal exam:

1	2	3	4	5 (circled)
(Prefer ophthalmologist's office)		undecided		(Prefer family physician)

☑ yes ☐ no **Do you expect the camera flashlight to bother you?**

☑ yes ☐ no **Do you expect the pupil dilating drops to bother you?**

☐ yes ☑ no **In the past five years have you been examined by an ophthalmologist?**

☑ yes ☐ no **Do you currently carry health insurance?**

☐ yes ☑ no **Sometimes photographs can only be made if your pupils are made larger with a drop. Were you aware of this?**

AFTER telediagnosis

What number best expresses your *satisfaction* with telediagnosis:

1	2	3	4	5 (circled)
(Terrible)		undecided		(Very good)

What number best expresses your where you think the *quality* of the retinal exam is best::

1	2	3	4	5 (circled)
(Better in ophthalmologist's office)		undecided		(Better through Telediagnosis)

What number best expresses *where* you prefer to have the retinal exam <u>now</u>:

1	2	3	4	5 (circled)
(Prefer ophthalmologist's office)		undecided		(Prefer family physician)

☐ yes ☑ no **Did the camera flashlight bother you?**

☑ yes ☐ no **Did the pupil dilating drops bother you?** ☐ not applicable

[redacted]

[redacted]

Patient Questionnaire

Name [redacted] Date [redacted]

[redacted]

BEFORE telediagnosis

Thank you for your willingness to participate in this research project.

You are about to undergo digital photographing and diagnosis over the internet by ophthalmologists who are retinal specialists at the University of Iowa. They will evaluate your photographs for signs of diabetic retinopathy, a complication of diabetes. This new technique is called 'telediagnosis'. Until telediagnosis was available, you could only be screened in an ophthalmologist's office with a dilated retinal exam. Please give your best answer.

What number best expresses where you think the *quality* of the retinal exam is best:

1	2	3	4	5
(Better in ophthalmologist's office)		undecided		(Better through Telediagnosis)

What number best expresses *where* you prefer to have the retinal exam:

1	2	3	4	5
(Prefer ophthalmologist's office)		undecided		(Prefer family physician)

☐ yes ☑ no **Do you expect the camera flashlight to bother you?**

☐ yes ☑ no **Do you expect the pupil dilating drops to bother you?**

☐ yes ☑ no **In the past five years have you been examined by an ophthalmologist?**

☑ yes ☐ no **Do you currently carry health insurance?**

☑ yes ☐ no **Sometimes photographs can only be made if your pupils are made larger with a drop. Were you aware of this?**

AFTER telediagnosis

What number best expresses your *satisfaction* with telediagnosis:

1	2	3	4	5
(Terrible)		undecided		(Very good)

What number best expresses your where you think the *quality* of the retinal exam is best::

1	2	3	4	5
(Better in ophthalmologist's office)		undecided		(Better through Telediagnosis)

What number best expresses *where* you prefer to have the retinal exam <u>now</u>:

1	2	3	4	5
(Prefer ophthalmologist's office)		undecided		(Prefer family physician)

☐ yes ☑ no **Did the camera flashlight bother you?**

☐ yes ☐ no **Did the pupil dilating drops bother you?** ☑ not applicable

[redacted]

[redacted]

Patient Questionnaire

[redacted]

Name [redacted] Date [redacted]

[redacted]

BEFORE telediagnosis

Thank you for your willingness to participate in this research project.

You are about to undergo digital photographing and diagnosis over the internet by ophthalmologists who are retinal specialists at the University of Iowa. They will evaluate your photographs for signs of diabetic retinopathy, a complication of diabetes. This new technique is called 'telediagnosis'. Until telediagnosis was available, you could only be screened in an ophthalmologist's office with a dilated retinal exam. Please give your best answer.

What number best expresses where you think the *quality* of the retinal exam is best:

**(1)**	2	3	4	5
(Better in ophthalmologist's office)		undecided	(Better through Telediagnosis)	

What number best expresses *where* you prefer to have the retinal exam:

1	2	3	4	**(5)**
(Prefer ophthalmologist's office)		undecided	(Prefer family physician)	

☐ yes ☒ no Do you expect the camera flashlight to bother you?

☐ yes ☒ no Do you expect the pupil dilating drops to bother you?

☒ yes ☐ no In the past five years have you been examined by an ophthalmologist?

☒ yes ☐ no Do you currently carry health insurance?

☒ yes ☐ no Sometimes photographs can only be made if your pupils are made larger with a drop. Were you aware of this?

AFTER telediagnosis

What number best expresses your *satisfaction* with telediagnosis:

1	2	3	4	**(5)**
(Terrible)		undecided		(Very good)

What number best expresses your where you think the *quality* of the retinal exam is best::

1	2	3	4	**(5)**
(Better in ophthalmologist's office)		undecided	(Better through Telediagnosis)	

What number best expresses *where* you prefer to have the retinal exam <u>now</u>:

1	2	3	4	**(5)**
(Prefer ophthalmologist's office)		undecided	(Prefer family physician)	

☐ yes ☒ no Did the camera flashlight bother you?

☐ yes ☐ no Did the pupil dilating drops bother you? ☒ not applicable

[redacted] [redacted]

[redacted]

Patient Questionnaire

Name [redacted] Date [redacted]

BEFORE telediagnosis

Thank you for your willingness to participate in this research project.

You are about to undergo digital photographing and diagnosis over the internet by ophthalmologists who are retinal specialists at the University of Iowa. They will evaluate your photographs for signs of diabetic retinopathy, a complication of diabetes. This new technique is called 'telediagnosis'. Until telediagnosis was available, you could only be screened in an ophthalmologist's office with a dilated retinal exam. Please give your best answer.

What number best expresses where you think the *quality* of the retinal exam is best:

1 (Better in ophthalmologist's office)	2	3 undecided	4 (Better through Telediagnosis)	5

What number best expresses *where* you prefer to have the retinal exam:

1 (Prefer ophthalmologist's office)	2	3 undecided	4 (Prefer family physician)	5

☐ yes ☐ no **Do you expect the camera flashlight to bother you?**

☐ yes ☐ no **Do you expect the pupil dilating drops to bother you?**

☐ yes ☐ no **In the past five years have you been examined by an ophthalmologist?**

☐ yes ☐ no **Do you currently carry health insurance?**

☐ yes ☐ no **Sometimes photographs can only be made if your pupils are made larger with a drop. Were you aware of this?**

AFTER telediagnosis

What number best expresses your *satisfaction* with telediagnosis:

1 (Terrible)	2	3 undecided	4	5 (Very good)

What number best expresses your where you think the *quality* of the retinal exam is best::

1 (Better in ophthalmologist's office)	2	3 undecided	4 (Better through Telediagnosis)	5

What number best expresses *where* you prefer to have the retinal exam <u>now</u>:

1 (Prefer ophthalmologist's office)	2	3 undecided	4 (Prefer family physician)	5

☐ yes ☐ no **Did the camera flashlight bother you?**

☐ yes ☐ no **Did the pupil dilating drops bother you?** ☐ not applicable

[redacted]

[redacted]

[redacted]naire

Name [redacted] Date [redacted]

BEFORE telediagnosis

Thank you for your willingness to participate in this research project.

You are about to undergo digital photographing and diagnosis over the internet by ophthalmologists who are retinal specialists at the University of Iowa. They will evaluate your photographs for signs of diabetic retinopathy, a complication of diabetes. This new technique is called 'telediagnosis'. Until telediagnosis was available, you could only be screened in an ophthalmologist's office with a dilated retinal exam. Please give your best answer.

What number best expresses where you think the *quality* of the retinal exam is best:

1 (Better in ophthalmologist's office)	2	3 undecided	4	5 (Better through Telediagnosis)

What number best expresses *where* you prefer to have the retinal exam:

1 (Prefer ophthalmologist's office)	2	3 undecided	4	5 (Prefer family physician)

☐ yes ☑ no **Do you expect the camera flashlight to bother you?**

☑ yes ☐ no **Do you expect the pupil dilating drops to bother you?**

☐ yes ☑ no **In the past five years have you been examined by an ophthalmologist?**

☑ yes ☐ no **Do you currently carry health insurance?**

☐ yes ☑ no **Sometimes photographs can only be made if your pupils are made larger with a drop. Were you aware of this?**

AFTER telediagnosis

What number best expresses your *satisfaction* with telediagnosis:

1 (Terrible)	2	3 undecided	4	5 (Very good)

What number best expresses your where you think the *quality* of the retinal exam is best::

1 (Better in ophthalmologist's office)	2	3 undecided	4	5 (Better through Telediagnosis)

What number best expresses *where* you prefer to have the retinal exam <u>now</u>:

1 (Prefer ophthalmologist's office)	2	3 undecided	4	5 (Prefer family physician)

☐ yes ☒ no **Did the camera flashlight bother you?**

☒ yes ☐ no **Did the pupil dilating drops bother you?** ☐ not applicable

[redacted] [redacted]

[redacted] [redacted]

Patient Questionnaire

Name [redacted] Date [redacted]

BEFORE telediagnosis

Thank you for your willingness to participate in this research project.

You are about to undergo digital photographing and diagnosis over the internet by ophthalmologists who are retinal specialists at the University of Iowa. They will evaluate your photographs for signs of diabetic retinopathy, a complication of diabetes. This new technique is called 'telediagnosis'. Until telediagnosis was available, you could only be screened in an ophthalmologist's office with a dilated retinal exam. Please give your best answer.

What number best expresses where you think the *quality* of the retinal exam is best:

1 (Better in ophthalmologist's office)	2	3 undecided	(4) circled	5 (Better through Telediagnosis)

What number best expresses *where* you prefer to have the retinal exam:

1 (Prefer ophthalmologist's office)	2	3 undecided	(4) circled	5 (Prefer family physician)

☐ yes ☒ no Do you expect the camera flashlight to bother you?

☒ yes ☐ no Do you expect the pupil dilating drops to bother you?

☐ yes ☒ no In the past five years have you been examined by an ophthalmologist?

☒ yes ☐ no Do you currently carry health insurance?

☒ yes ☐ no Sometimes photographs can only be made if your pupils are made larger with a drop. Were you aware of this?

AFTER telediagnosis

What number best expresses your *satisfaction* with telediagnosis:

1 (Terrible)	2	3 undecided	(4) circled	5 (Very good)

What number best expresses your where you think the *quality* of the retinal exam is best::

1 (Better in ophthalmologist's office)	2	3 undecided	(4) circled	5 (Better through Telediagnosis)

What number best expresses *where* you prefer to have the retinal exam <u>now</u>:

1 (Prefer ophthalmologist's office)	2	3 undecided	(4) circled	5 (Prefer family physician)

☐ yes ☒ no Did the camera flashlight bother you?

☐ yes ☐ no Did the pupil dilating drops bother you? ☒ not applicable

Patient Questionnaire

Name [redacted] Date [redacted]

BEFORE telediagnosis

Thank you for your willingness to participate in this research project.

You are about to undergo digital photographing and diagnosis over the internet by ophthalmologists who are retinal specialists at the University of Iowa. They will evaluate your photographs for signs of diabetic retinopathy, a complication of diabetes. This new technique is called 'telediagnosis'. Until telediagnosis was available, you could only be screened in an ophthalmologist's office with a dilated retinal exam. Please give your best answer.

What number best expresses where you think the *quality* of the retinal exam is best:

1	2	3	4	5
(Better in ophthalmologist's office)		undecided		(Better through Telediagnosis)

What number best expresses *where* you prefer to have the retinal exam:

1	2	3	4	5
(Prefer ophthalmologist's office)		undecided		(Prefer family physician)

☐ yes ☒ no **Do you expect the camera flashlight to bother you?**

☐ yes ☒ no **Do you expect the pupil dilating drops to bother you?**

☐ yes ☒ no **In the past five years have you been examined by an ophthalmologist?**

☒ yes ☐ no **Do you currently carry health insurance?**

☐ yes ☒ no **Sometimes photographs can only be made if your pupils are made larger with a drop. Were you aware of this?**

AFTER telediagnosis

What number best expresses your *satisfaction* with telediagnosis:

1	2	3	4	5
(Terrible)		undecided		(Very good)

What number best expresses your where you think the *quality* of the retinal exam is best::

1	2	3	4	5
(Better in ophthalmologist's office)		undecided		(Better through Telediagnosis)

What number best expresses *where* you prefer to have the retinal exam <u>now</u>:

1	2	3	4	5
(Prefer ophthalmologist's office)		undecided		(Prefer family physician)

☐ yes ☒ no **Did the camera flashlight bother you?**

☐ yes ☐ no **Did the pupil dilating drops bother you?** ☒ not applicable

[redacted]

Patient Questionnaire

Name [redacted] Date [redacted]

BEFORE telediagnosis

Thank you for your willingness to participate in this research project.

You are about to undergo digital photographing and diagnosis over the internet by ophthalmologists who are retinal specialists at the University of Iowa. They will evaluate your photographs for signs of diabetic retinopathy, a complication of diabetes. This new technique is called 'telediagnosis'. Until telediagnosis was available, you could only be screened in an ophthalmologist's office with a dilated retinal exam. Please give your best answer.

What number best expresses where you think the *quality* of the retinal exam is best:

1	2	3	4	5
(Better in ophthalmologist's office)		undecided	(Better through Telediagnosis)	

What number best expresses *where* you prefer to have the retinal exam:

1	2	3	4	5
(Prefer ophthalmologist's office)		undecided	(Prefer family physician)	

☐ yes ☐ no **Do you expect the camera flashlight to bother you?**

☐ yes ☐ no **Do you expect the pupil dilating drops to bother you?**

☐ yes ☐ no **In the past five years have you been examined by an ophthalmologist?**

☐ yes ☐ no **Do you currently carry health insurance?**

☐ yes ☐ no **Sometimes photographs can only be made if your pupils are made larger with a drop. Were you aware of this?**

AFTER telediagnosis

What number best expresses your *satisfaction* with telediagnosis:

1	2	3	4	5
(Terrible)		undecided		(Very good)

What number best expresses your where you think the *quality* of the retinal exam is best::

1	2	3	4	5
(Better in ophthalmologist's office)		undecided	(Better through Telediagnosis)	

What number best expresses *where* you prefer to have the retinal exam <u>now</u>:

1	2	3	4	5
(Prefer ophthalmologist's office)		undecided	(Prefer family physician)	

☐ yes ☐ no **Did the camera flashlight bother you?**

☐ yes ☐ no **Did the pupil dilating drops bother you?** ☐ not applicable

[redacted]

[redacted]

Patient Questionnaire

Name [redacted] Date [redacted]

BEFORE telediagnosis

Thank you for your willingness to participate in this research project.

You are about to undergo digital photographing and diagnosis over the internet by ophthalmologists who are retinal specialists at the University of Iowa. They will evaluate your photographs for signs of diabetic retinopathy, a complication of diabetes. This new technique is called 'telediagnosis'. Until telediagnosis was available, you could only be screened in an ophthalmologist's office with a dilated retinal exam. Please give your best answer.

What number best expresses where you think the *quality* of the retinal exam is best:

1	2	3	4	5
(Better in ophthalmologist's office)		undecided		(Better through Telediagnosis)

What number best expresses *where* you prefer to have the retinal exam:

1	2	3	4	5
(Prefer ophthalmologist's office)		undecided		(Prefer family physician)

☐ yes ☒ no **Do you expect the camera flashlight to bother you?**

☐ yes ☒ no **Do you expect the pupil dilating drops to bother you?**

☐ yes ☒ no **In the past five years have you been examined by an ophthalmologist?**

☒ yes ☐ no **Do you currently carry health insurance?**

☒ yes ☐ no **Sometimes photographs can only be made if your pupils are made larger with a drop. Were you aware of this?**

AFTER telediagnosis

What number best expresses your *satisfaction* with telediagnosis:

1	2	3	4	5
(Terrible)		undecided		(Very good)

What number best expresses your where you think the *quality* of the retinal exam is best::

1	2	3	4	5
(Better in ophthalmologist's office)		undecided		(Better through Telediagnosis)

What number best expresses *where* you prefer to have the retinal exam <u>now</u>:

1	2	3	4	5
(Prefer ophthalmologist's office)		undecided		(Prefer family physician)

[redacted]

☐ yes ☒ no **Did the camera flashlight bother you?**

☐ yes ☐ no **Did the pupil dilating drops bother you?** ☒ not applicable

Patient Questionnaire

Name [redacted] Date [redacted]

BEFORE telediagnosis

Thank you for your willingness to participate in this research project.

You are about to undergo digital photographing and diagnosis over the internet by ophthalmologists who are retinal specialists at the University of Iowa. They will evaluate your photographs for signs of diabetic retinopathy, a complication of diabetes. This new technique is called 'telediagnosis'. Until telediagnosis was available, you could only be screened in an ophthalmologist's office with a dilated retinal exam. Please give your best answer.

What number best expresses where you think the *quality* of the retinal exam is best:

1	2	3	4	5
(Better in ophthalmologist's office)		undecided		(Better through Telediagnosis)

What number best expresses *where* you prefer to have the retinal exam:

1	2	3	4	5
(Prefer ophthalmologist's office)		undecided		(Prefer family physician)

☐ yes ☒ no **Do you expect the camera flashlight to bother you?**

☐ yes ☒ no **Do you expect the pupil dilating drops to bother you?**

☐ yes ☒ no ? **In the past five years have you been examined by an ophthalmologist?**

☒ yes ☐ no **Do you currently carry health insurance?**

☒ yes ☐ no **Sometimes photographs can only be made if your pupils are made larger with a drop. Were you aware of this?**

AFTER telediagnosis

What number best expresses your *satisfaction* with telediagnosis:

1	2	3	4	5
(Terrible)		undecided		(Very good)

What number best expresses your where you think the *quality* of the retinal exam is best::

1	2	3	4	5
(Better in ophthalmologist's office)		undecided		(Better through Telediagnosis)

What number best expresses *where* you prefer to have the retinal exam <u>now</u>:

1	2	3	4	5
(Prefer ophthalmologist's office)		undecided		(Prefer family physician)

☐ yes ☒ no **Did the camera flashlight bother you?**

☐ yes ☒ no **Did the pupil dilating drops bother you?** ☐ not applicable

[redacted]

Patient Questionnaire

Name [redacted] Date_ [redacted]

BEFORE telediagnosis

Thank you for your willingness to participate in this research project.

You are about to undergo digital photographing and diagnosis over the internet by ophthalmologists who are retinal specialists at the University of Iowa. They will evaluate your photographs for signs of diabetic retinopathy, a complication of diabetes. This new technique is called 'telediagnosis'. Until telediagnosis was available, you could only be screened in an ophthalmologist's office with a dilated retinal exam. Please give your best answer.

What number best expresses where you think the *quality* of the retinal exam is best:

1	2	3	4	5
(Better in ophthalmologist's office)		undecided		(Better through Telediagnosis)

What number best expresses *where* you prefer to have the retinal exam:

1	2	3	4	5
(Prefer ophthalmologist's office)		undecided		(Prefer family physician)

☐ yes ☒ no **Do you expect the camera flashlight to bother you?**

☐ yes ☒ no **Do you expect the pupil dilating drops to bother you?**

☒ yes ☐ no **In the past five years have you been examined by an ophthalmologist?**

☒ yes ☐ no **Do you currently carry health insurance?**

☒ yes ☐ no **Sometimes photographs can only be made if your pupils are made larger with a drop. Were you aware of this?**

AFTER telediagnosis

What number best expresses your *satisfaction* with telediagnosis:

1	2	3	4	5
(Terrible)		undecided		(Very good)

What number best expresses your where you think the *quality* of the retinal exam is best::

1	2	3	4	5
(Better in ophthalmologist's office)		undecided		(Better through Telediagnosis)

What number best expresses *where* you prefer to have the retinal exam now:

1	2	3	4	5
(Prefer ophthalmologist's office)		undecided		(Prefer family physician)

☐ yes ☒ no **Did the camera flashlight bother you?**

☐ yes ☐ no **Did the pupil dilating drops bother you?** ☒ not applicable

[redacted]

[redacted]

[redacted]t Questionnaire

Name [redacted] Date [redacted]

BEFORE telediagnosis

Thank you for your willingness to participate in this research project.

You are about to undergo digital photographing and diagnosis over the internet by ophthalmologists who are retinal specialists at the University of Iowa. They will evaluate your photographs for signs of diabetic retinopathy, a complication of diabetes. This new technique is called 'telediagnosis'. Until telediagnosis was available, you could only be screened in an ophthalmologist's office with a dilated retinal exam. Please give your best answer.

What number best expresses where you think the *quality* of the retinal exam is best:

1	2	3	4	5
(Better in ophthalmologist's office)		undecided		(Better through Telediagnosis)

What number best expresses *where* you prefer to have the retinal exam:

1	2	3	4	5
(Prefer ophthalmologist's office)		undecided		(Prefer family physician)

☐ yes ☒ no **Do you expect the camera flashlight to bother you?**

☐ yes ☒ no **Do you expect the pupil dilating drops to bother you?**

☒ yes ☐ no **In the past five years have you been examined by an ophthalmologist?**

☒ yes ☐ no **Do you currently carry health insurance?**

☒ yes ☐ no **Sometimes photographs can only be made if your pupils are made larger with a drop. Were you aware of this?**

AFTER telediagnosis

What number best expresses your *satisfaction* with telediagnosis:

1	2	3	4	5
(Terrible)		undecided		(Very good)

What number best expresses your where you think the *quality* of the retinal exam is best::

1	2	3	4	5
(Better in ophthalmologist's office)		undecided		(Better through Telediagnosis)

What number best expresses *where* you prefer to have the retinal exam <u>now</u>:

1	2	3	4	5
(Prefer ophthalmologist's office)		undecided		(Prefer family physician)

☐ yes ☒ no **Did the camera flashlight bother you?**

☐ yes ☐ no **Did the pupil dilating drops bother you?** ☒ not applicable

[redacted]

[redacted]

Patient Questionnaire

Name [redacted] Date [redacted]

BEFORE telediagnosis

Thank you for your willingness to participate in this research project.

You are about to undergo digital photographing and diagnosis over the internet by ophthalmologists who are retinal specialists at the University of Iowa. They will evaluate your photographs for signs of diabetic retinopathy, a complication of diabetes. This new technique is called 'telediagnosis'. Until telediagnosis was available, you could only be screened in an ophthalmologist's office with a dilated retinal exam. Please give your best answer.

What number best expresses where you think the *quality* of the retinal exam is best:

1 (Better in ophthalmologist's office)	2	3 undecided	4	5 (Better through Telediagnosis)

What number best expresses *where* you prefer to have the retinal exam:

1 (Prefer ophthalmologist's office)	2	3 undecided	4	5 (Prefer family physician)

☐ yes ☒ no **Do you expect the camera flashlight to bother you?**

☐ yes ☒ no **Do you expect the pupil dilating drops to bother you?**

☒ yes ☐ no **In the past five years have you been examined by an ophthalmologist?**

☒ yes ☐ no **Do you currently carry health insurance?**

☐ yes ☒ no **Sometimes photographs can only be made if your pupils are made larger with a drop. Were you aware of this?**

AFTER telediagnosis

What number best expresses your *satisfaction* with telediagnosis:

1 (Terrible)	2	3 undecided	4	5 (Very good)

What number best expresses your where you think the *quality* of the retinal exam is best::

1 (Better in ophthalmologist's office)	2	3 undecided	4	5 (Better through Telediagnosis)

What number best expresses *where* you prefer to have the retinal exam <u>now</u>:

1 (Prefer ophthalmologist's office)	2	3 undecided	4	5 (Prefer family physician)

☐ yes ☒ no **Did the camera flashlight bother you?**

☐ yes ☒ no **Did the pupil dilating drops bother you?** ☐ not applicable

[redacted]

Patient Questionnaire

Name [redacted] Date [redacted]

BEFORE telediagnosis

Thank you for your willingness to participate in this research project.

You are about to undergo digital photographing and diagnosis over the internet by ophthalmologists who are retinal specialists at the University of Iowa. They will evaluate your photographs for signs of diabetic retinopathy, a complication of diabetes. This new technique is called 'telediagnosis'. Until telediagnosis was available, you could only be screened in an ophthalmologist's office with a dilated retinal exam. Please give your best answer.

What number best expresses where you think the *quality* of the retinal exam is best:

1	2	3	4	5
(Better in ophthalmologist's office)		undecided		(Better through Telediagnosis)

What number best expresses *where* you prefer to have the retinal exam:

1	2	3	4	5
(Prefer ophthalmologist's office)		undecided		(Prefer family physician)

☐ yes ☒ no Do you expect the camera flashlight to bother you?

☐ yes ☒ no Do you expect the pupil dilating drops to bother you?

☐ yes ☒ no In the past five years have you been examined by an ophthalmologist?

☒ yes ☐ no Do you currently carry health insurance?

☒ yes ☐ no Sometimes photographs can only be made if your pupils are made larger with a drop. Were you aware of this?

AFTER telediagnosis

What number best expresses your *satisfaction* with telediagnosis:

1	2	3	4	5
(Terrible)		undecided		(Very good)

What number best expresses your where you think the *quality* of the retinal exam is best::

1	2	3	4	5
(Better in ophthalmologist's office)		undecided		(Better through Telediagnosis)

What number best expresses *where* you prefer to have the retinal exam <u>now</u>:

1	2	3	4	5
(Prefer ophthalmologist's office)		undecided		(Prefer family physician)

☐ yes ☒ no Did the camera flashlight bother you?

☒ yes ☐ no Did the pupil dilating drops bother you? ☐ not applicable

[redacted]

[redacted]

Patient Questionnaire

[redacted]

Name [redacted] Date [redacted]

BEFORE telediagnosis

Thank you for your willingness to participate in this research project.

You are about to undergo digital photographing and diagnosis over the internet by ophthalmologists who are retinal specialists at the University of Iowa. They will evaluate your photographs for signs of diabetic retinopathy, a complication of diabetes. This new technique is called 'telediagnosis'. Until telediagnosis was available, you could only be screened in an ophthalmologist's office with a dilated retinal exam. Please give your best answer.

What number best expresses where you think the *quality* of the retinal exam is best:

1	2	3	4	5
(Better in ophthalmologist's office)		undecided		(Better through Telediagnosis)

What number best expresses *where* you prefer to have the retinal exam:

1	2	3	4	5
(Prefer ophthalmologist's office)		undecided		(Prefer family physician)

☐ yes ☒ no Do you expect the camera flashlight to bother you?

☐ yes ☒ no Do you expect the pupil dilating drops to bother you?

☒ yes ☐ no In the past five years have you been examined by an ophthalmologist?

☒ yes ☐ no Do you currently carry health insurance?

☐ yes ☒ no Sometimes photographs can only be made if your pupils are made larger with a drop. Were you aware of this?

AFTER telediagnosis

What number best expresses your *satisfaction* with telediagnosis:

1	2	3	4	5
(Terrible)		undecided		(Very good)

What number best expresses your where you think the *quality* of the retinal exam is best::

1	2	3	4	5
(Better in ophthalmologist's office)		undecided		(Better through Telediagnosis)

What number best expresses *where* you prefer to have the retinal exam <u>now</u>:

1	2	3	4	5
(Prefer ophthalmologist's office)		undecided		(Prefer family physician)

☒ yes ☐ no Did the camera flashlight bother you?

☐ yes ☒ no Did the pupil dilating drops bother you? ☐ not applicable

[redacted]

Patient Questionnaire

Name [redacted] Date [redacted]

BEFORE telediagnosis

Thank you for your willingness to participate in this research project.

You are about to undergo digital photographing and diagnosis over the internet by ophthalmologists who are retinal specialists at the University of Iowa. They will evaluate your photographs for signs of diabetic retinopathy, a complication of diabetes. This new technique is called 'telediagnosis'. Until telediagnosis was available, you could only be screened in an ophthalmologist's office with a dilated retinal exam. Please give your best answer.

What number best expresses where you think the *quality* of the retinal exam is best:

1	2	3	4	5
(Better in ophthalmologist's office)		undecided		(Better through Telediagnosis)

What number best expresses *where* you prefer to have the retinal exam:

1	2	3	4	5
(Prefer ophthalmologist's office)		undecided		(Prefer family physician)

☐ yes ☒ no **Do you expect the camera flashlight to bother you?**

☐ yes ☒ no **Do you expect the pupil dilating drops to bother you?**

☐ yes ☒ no **In the past five years have you been examined by an ophthalmologist?**

☒ yes ☐ no **Do you currently carry health insurance?** [redacted]

☒ yes ☐ no **Sometimes photographs can only be made if your pupils are made larger with a drop. Were you aware of this?**

AFTER telediagnosis

What number best expresses your *satisfaction* with telediagnosis:

1	2	3	4	5
(Terrible)		undecided		(Very good)

What number best expresses your where you think the *quality* of the retinal exam is best::

1	2	3	4	5
(Better in ophthalmologist's office)		undecided		(Better through Telediagnosis)

What number best expresses *where* you prefer to have the retinal exam <u>now</u>:

1	2	3	4	5
(Prefer ophthalmologist's office)		undecided		(Prefer family physician)

☐ yes ☒ no **Did the camera flashlight bother you?**

☐ yes ☒ no **Did the pupil dilating drops bother you?** ☐ not applicable

[redacted]

[redacted]

Patient Questionnaire

Name [redacted] Date [redacted]

BEFORE telediagnosis

Thank you for your willingness to participate in this research project.

You are about to undergo digital photographing and diagnosis over the internet by ophthalmologists who are retinal specialists at the University of Iowa. They will evaluate your photographs for signs of diabetic retinopathy, a complication of diabetes. This new technique is called 'telediagnosis'. Until telediagnosis was available, you could only be screened in an ophthalmologist's office with a dilated retinal exam. Please give your best answer.

What number best expresses where you think the *quality* of the retinal exam is best:

1 (Better in ophthalmologist's office)	2	3 undecided	4	5 (Better through Telediagnosis)

What number best expresses *where* you prefer to have the retinal exam:

1 (Prefer ophthalmologist's office)	2	3 undecided	4	5 (Prefer family physician)

☐ yes ☒ no **Do you expect the camera flashlight to bother you?**

☐ yes ☒ no **Do you expect the pupil dilating drops to bother you?**

☒ yes ☒ no **In the past five years have you been examined by an ophthalmologist?**

☒ yes ☐ no **Do you currently carry health insurance?**

☒ yes ☐ no **Sometimes photographs can only be made if your pupils are made larger with a drop. Were you aware of this?**

AFTER telediagnosis

What number best expresses your *satisfaction* with telediagnosis:

1 (Terrible)	2	3 undecided	4	5 (Very good)

What number best expresses your where you think the *quality* of the retinal exam is best::

1 (Better in ophthalmologist's office)	2	3 undecided	4	5 (Better through Telediagnosis)

What number best expresses *where* you prefer to have the retinal exam <u>now</u>:

1 (Prefer ophthalmologist's office)	2	3 undecided	4	5 (Prefer family physician)

☐ yes ☒ no **Did the camera flashlight bother you?**

☐ yes ☐ no **Did the pupil dilating drops bother you?** ☒ not applicable

Patient Questionnaire

Name [redacted] Date [redacted]

BEFORE telediagnosis

Thank you for your willingness to participate in this research project.

You are about to undergo digital photographing and diagnosis over the internet by ophthalmologists who are retinal specialists at the University of Iowa. They will evaluate your photographs for signs of diabetic retinopathy, a complication of diabetes. This new technique is called 'telediagnosis'. Until telediagnosis was available, you could only be screened in an ophthalmologist's office with a dilated retinal exam. Please give your best answer.

What number best expresses where you think the *quality* of the retinal exam is best:

1	2	3	4	5
(Better in ophthalmologist's office)		undecided	(Better through Telediagnosis)	

What number best expresses *where* you prefer to have the retinal exam:

1	2	3	4	5
(Prefer ophthalmologist's office)		undecided		(Prefer family physician)

☐ yes ☒ no **Do you expect the camera flashlight to bother you?**

☐ yes ☒ no **Do you expect the pupil dilating drops to bother you?**

☒ yes ☐ no **In the past five years have you been examined by an ophthalmologist?**

☒ yes ☐ no **Do you currently carry health insurance?**

☒ yes ☐ no **Sometimes photographs can only be made if your pupils are made larger with a drop. Were you aware of this?**

AFTER telediagnosis

What number best expresses your *satisfaction* with telediagnosis:

1	2	3	4	5
(Terrible)		undecided		(Very good)

What number best expresses your where you think the *quality* of the retinal exam is best::

1	2	3	4	5
(Better in ophthalmologist's office)		undecided	(Better through Telediagnosis)	

What number best expresses *where* you prefer to have the retinal exam <u>now</u>:

1	2	3	4	5
(Prefer ophthalmologist's office)		undecided		(Prefer family physician)

☐ yes ☒ no **Did the camera flashlight bother you?**

☐ yes ☐ no **Did the pupil dilating drops bother you?** ☒ not applicable

[redacted]

Patient Questionnaire

Name [redacted] Date [redacted]

BEFORE telediagnosis

Thank you for your willingness to participate in this research project.

You are about to undergo digital photographing and diagnosis over the internet by ophthalmologists who are retinal specialists at the University of Iowa. They will evaluate your photographs for signs of diabetic retinopathy, a complication of diabetes. This new technique is called 'telediagnosis'. Until telediagnosis was available, you could only be screened in an ophthalmologist's office with a dilated retinal exam. Please give your best answer.

What number best expresses where you think the *quality* of the retinal exam is best:

1	2	3 (circled)	4	5
(Better in ophthalmologist's office)		undecided		(Better through Telediagnosis)

What number best expresses *where* you prefer to have the retinal exam:

1	2	3	4	5 (circled)
(Prefer ophthalmologist's office)		undecided		(Prefer family physician)

☐ yes ☒ no **Do you expect the camera flashlight to bother you?**

☐ yes ☒ no **Do you expect the pupil dilating drops to bother you?**

☐ yes ☒ no **In the past five years have you been examined by an ophthalmologist?**

☒ yes ☐ no **Do you currently carry health insurance?**

☒ yes ☐ no **Sometimes photographs can only be made if your pupils are made larger with a drop. Were you aware of this?**

AFTER telediagnosis

What number best expresses your *satisfaction* with telediagnosis:

1	2	3	4	5 (circled)
(Terrible)		undecided		(Very good)

What number best expresses your where you think the *quality* of the retinal exam is best::

1	2	3 (circled)	4	5
(Better in ophthalmologist's office)		undecided		(Better through Telediagnosis)

What number best expresses *where* you prefer to have the retinal exam now:

1	2	3 (circled)	4	5
(Prefer ophthalmologist's office)		undecided		(Prefer family physician)

☐ yes ☒ no **Did the camera flashlight bother you?**

☐ yes ☐ no **Did the pupil dilating drops bother you?** ☒ not applicable

[redacted]

Patient Questionnaire

Name [redacted] Date [redacted]

BEFORE telediagnosis

Thank you for your willingness to participate in this research project.

You are about to undergo digital photographing and diagnosis over the internet by ophthalmologists who are retinal specialists at the University of Iowa. They will evaluate your photographs for signs of diabetic retinopathy, a complication of diabetes. This new technique is called 'telediagnosis'. Until telediagnosis was available, you could only be screened in an ophthalmologist's office with a dilated retinal exam. Please give your best answer.

What number best expresses where you think the *quality* of the retinal exam is best:

1 (Better in ophthalmologist's office)	2	3 undecided	4	5 (Better through Telediagnosis)

What number best expresses *where* you prefer to have the retinal exam:

1 (Prefer ophthalmologist's office)	2	3 undecided	4	5 (Prefer family physician)

☐ yes ☑ no Do you expect the camera flashlight to bother you?

☐ yes ☑ no Do you expect the pupil dilating drops to bother you?

☑ yes ☐ no In the past five years have you been examined by an ophthalmologist?

☑ yes ☐ no Do you currently carry health insurance?

☐ yes ☑ no Sometimes photographs can only be made if your pupils are made larger with a drop. Were you aware of this?

AFTER telediagnosis

What number best expresses your *satisfaction* with telediagnosis:

1 (Terrible)	2	3 undecided	4	5 (Very good)

What number best expresses your where you think the *quality* of the retinal exam is best::

1 (Better in ophthalmologist's office)	2	3 undecided	4	5 (Better through Telediagnosis)

What number best expresses *where* you prefer to have the retinal exam now:

1 (Prefer ophthalmologist's office)	2	3 undecided	4	5 (Prefer family physician)

☐ yes ☒ no Did the camera flashlight bother you?

☐ yes ☒ no Did the pupil dilating drops bother you? ☐ not applicable

[redacted]

[redacted]

Patient Questionnaire

Name [redacted] Date [redacted]

BEFORE telediagnosis

Thank you for your willingness to participate in this research project.

You are about to undergo digital photographing and diagnosis over the internet by ophthalmologists who are retinal specialists at the University of Iowa. They will evaluate your photographs for signs of diabetic retinopathy, a complication of diabetes. This new technique is called 'telediagnosis'. Until telediagnosis was available, you could only be screened in an ophthalmologist's office with a dilated retinal exam. Please give your best answer.

What number best expresses where you think the *quality* of the retinal exam is best:

1	2	3	4	5
(Better in ophthalmologist's office)		undecided		(Better through Telediagnosis)

What number best expresses *where* you prefer to have the retinal exam:

1	2	3	4	5
(Prefer ophthalmologist's office)		undecided		(Prefer family physician)

☐ yes ☐ no **Do you expect the camera flashlight to bother you?**

☐ yes ☐ no **Do you expect the pupil dilating drops to bother you?**

☐ yes ☐ no **In the past five years have you been examined by an ophthalmologist?**

☐ yes ☐ no **Do you currently carry health insurance?**

☐ yes ☐ no **Sometimes photographs can only be made if your pupils are made larger with a drop. Were you aware of this?**

AFTER telediagnosis

What number best expresses your *satisfaction* with telediagnosis:

1	2	3	4	5
(Terrible)		undecided		(Very good)

What number best expresses your where you think the *quality* of the retinal exam is best::

1	2	3	4	5
(Better in ophthalmologist's office)		undecided		(Better through Telediagnosis)

What number best expresses *where* you prefer to have the retinal exam <u>now</u>:

1	2	3	4	5
(Prefer ophthalmologist's office)		undecided		(Prefer family physician)

☐ yes ☐ no **Did the camera flashlight bother you?**

☐ yes ☐ no **Did the pupil dilating drops bother you?** ☐ not applicable

[redacted]

Patient Questionnaire

Name [redacted] Date [redacted]

BEFORE telediagnosis

Thank you for your willingness to participate in this research project.

You are about to undergo digital photographing and diagnosis over the internet by ophthalmologists who are retinal specialists at the University of Iowa. They will evaluate your photographs for signs of diabetic retinopathy, a complication of diabetes. This new technique is called 'telediagnosis'. Until telediagnosis was available, you could only be screened in an ophthalmologist's office with a dilated retinal exam. Please give your best answer.

What number best expresses where you think the *quality* of the retinal exam is best:

1	2	3	4	5
(Better in ophthalmologist's office)		undecided		(Better through Telediagnosis)

What number best expresses *where* you prefer to have the retinal exam:

1	2	3	4	5
(Prefer ophthalmologist's office)		undecided		(Prefer family physician)

☒ yes ☐ no Do you expect the camera flashlight to bother you?

☒ yes ☐ no Do you expect the pupil dilating drops to bother you?

☒ yes ☐ no In the past five years have you been examined by an ophthalmologist?

☒ yes ☐ no Do you currently carry health insurance?

☐ yes ☒ no Sometimes photographs can only be made if your pupils are made larger with a drop. Were you aware of this?

AFTER telediagnosis

What number best expresses your *satisfaction* with telediagnosis:

1	2	3	4	5
(Terrible)		undecided		(Very good)

What number best expresses your where you think the *quality* of the retinal exam is best::

1	2	3	4	5
(Better in ophthalmologist's office)		undecided		(Better through Telediagnosis)

What number best expresses *where* you prefer to have the retinal exam now:

1	2	3	4	5
(Prefer ophthalmologist's office)		undecided		(Prefer family physician)

☐ yes ☒ no Did the camera flashlight bother you?

☐ yes ☐ no Did the pupil dilating drops bother you? ☒ not applicable

[redacted]

[redacted]

Patient Questionnaire

Name [redacted] Date [redacted]

BEFORE telediagnosis

Thank you for your willingness to participate in this research project.

You are about to undergo digital photographing and diagnosis over the internet by ophthalmologists who are retinal specialists at the University of Iowa. They will evaluate your photographs for signs of diabetic retinopathy, a complication of diabetes. This new technique is called 'telediagnosis'. Until telediagnosis was available, you could only be screened in an ophthalmologist's office with a dilated retinal exam. Please give your best answer.

What number best expresses where you think the *quality* of the retinal exam is best:

1	2	3	4	5
(Better in ophthalmologist's office)		undecided		(Better through Telediagnosis)

What number best expresses *where* you prefer to ~~have the~~ retinal exam:

1	2	3	4	5
(Prefer ophthalmologist's office)		undecided		(Prefer family physician)

☐ yes ☒ no **Do you expect the camera flashlight to bother you?**

☐ yes ☒ no **Do you expect the pupil dilating drops to bother you?**

☐ yes ☒ no **In the past five years have you been examined by an ophthalmologist?**

☒ yes ☐ no **Do you currently carry health insurance?**

☒ yes ☐ no **Sometimes photographs can only be made if your pupils are made larger with a drop. Were you aware of this?**

AFTER telediagnosis

What number best expresses your *satisfaction* with telediagnosis:

1	2	3	4	5
(Terrible)		undecided		(Very good)

What number best expresses your where you think the *quality* of the retinal exam is best::

1	2	3	4	5
(Better in ophthalmologist's office)		undecided		(Better through Telediagnosis)

What number best expresses *where* you prefer to have the retinal exam <u>now</u>:

1	2	3	4	5
(Prefer ophthalmologist's office)		undecided		(Prefer family physician)

☐ yes ☒ no **Did the camera flashlight bother you?**

☐ yes ☐ no **Did the pupil dilating drops bother you?** ☒ not applicable

[redacted]

Patient Questionnaire

Name [redacted] Date [redacted]

BEFORE telediagnosis

Thank you for your willingness to participate in this research project.

You are about to undergo digital photographing and diagnosis over the internet by ophthalmologists who are retinal specialists at the University of Iowa. They will evaluate your photographs for signs of diabetic retinopathy, a complication of diabetes. This new technique is called 'telediagnosis'. Until telediagnosis was available, you could only be screened in an ophthalmologist's office with a dilated retinal exam. Please give your best answer.

What number best expresses where you think the *quality* of the retinal exam is best:

1	2	3	4	5
(Better in ophthalmologist's office)		undecided		(Better through Telediagnosis)

What number best expresses *where* you prefer to have the retinal exam:

1	2	3	4	5
(Prefer ophthalmologist's office)		undecided		(Prefer family physician)

☐ yes ☒ no **Do you expect the camera flashlight to bother you?**

☐ yes ☒ no **Do you expect the pupil dilating drops to bother you?**

☐ yes ☒ no **In the past five years have you been examined by an ophthalmologist?**

☒ yes ☐ no **Do you currently carry health insurance?**

☒ yes ☐ no **Sometimes photographs can only be made if your pupils are made larger with a drop. Were you aware of this?**

AFTER telediagnosis

What number best expresses your *satisfaction* with telediagnosis:

1	2	3	4	5
(Terrible)		undecided		(Very good)

What number best expresses your where you think the *quality* of the retinal exam is best::

1	2	3	4	5
(Better in ophthalmologist's office)		undecided		(Better through Telediagnosis)

What number best expresses *where* you prefer to have the retinal exam <u>now</u>:

1	2	3	4	5
(Prefer ophthalmologist's office)		undecided		(Prefer family physician)

☐ yes ☒ no **Did the camera flashlight bother you?**

☐ yes ☐ no **Did the pupil dilating drops bother you?** ☒ not applicable

[redacted]

Patient Questionnaire

Name [redacted] Date [redacted]

BEFORE telediagnosis

Thank you for your willingness to participate in this research project.

You are about to undergo digital photographing and diagnosis over the internet by ophthalmologists who are retinal specialists at the University of Iowa. They will evaluate your photographs for signs of diabetic retinopathy, a complication of diabetes. This new technique is called 'telediagnosis'. Until telediagnosis was available, you could only be screened in an ophthalmologist's office with a dilated retinal exam. Please give your best answer.

What number best expresses where you think the *quality* of the retinal exam is best:

1	2	3	4	5
(Better in ophthalmologist's office)		undecided		(Better through Telediagnosis)

What number best expresses *where* you prefer to have the retinal exam:

1	2	3	4	5
(Prefer ophthalmologist's office)		undecided		(Prefer family physician)

☐ yes ☒ no **Do you expect the camera flashlight to bother you?**

☐ yes ☒ no **Do you expect the pupil dilating drops to bother you?**

☐ yes ☒ no **In the past five years have you been examined by an ophthalmologist?**

☒ yes ☐ no **Do you currently carry health insurance?**

☒ yes ☐ no **Sometimes photographs can only be made if your pupils are made larger with a drop. Were you aware of this?**

AFTER telediagnosis

What number best expresses your *satisfaction* with telediagnosis:

1	2	3	4	5
(Terrible)		undecided		(Very good)

What number best expresses your where you think the *quality* of the retinal exam is best::

1	2	3	4	5
(Better in ophthalmologist's office)		undecided		(Better through Telediagnosis)

What number best expresses *where* you prefer to have the retinal exam <u>now</u>:

1	2	3	4	5
(Prefer ophthalmologist's office)		undecided		(Prefer family physician)

☐ yes ☒ no **Did the camera flashlight bother you?**

☐ yes ☐ no **Did the pupil dilating drops bother you?** ☒ not applicable

[redacted]

[redacted] Patient Questionnaire [redacted]

Name [redacted] Date [redacted]

BEFORE telediagnosis

Thank you for your willingness to participate in this research project.

You are about to undergo digital photographing and diagnosis over the internet by ophthalmologists who are retinal specialists at the University of Iowa. They will evaluate your photographs for signs of diabetic retinopathy, a complication of diabetes. This new technique is called 'telediagnosis'. Until telediagnosis was available, you could only be screened in an ophthalmologist's office with a dilated retinal exam. Please give your best answer.

What number best expresses where you think the *quality* of the retinal exam is best:

1	2	3	4	5
(Better in ophthalmologist's office)		**(3)** undecided		(Better through Telediagnosis)

What number best expresses *where* you prefer to have the retinal exam:

1	2	3	4	5
(Prefer ophthalmologist's office)		**(3)** undecided		(Prefer family physician)

☒ yes ☐ no **Do you expect the camera flashlight to bother you?**

☐ yes ☒ no **Do you expect the pupil dilating drops to bother you?**

☐ yes ☒ no **In the past five years have you been examined by an ophthalmologist?** [redacted]

☒ yes ☐ no **Do you currently carry health insurance?**

☒ yes ☐ no **Sometimes photographs can only be made if your pupils are made larger with a drop. Were you aware of this?**

AFTER telediagnosis

What number best expresses your *satisfaction* with telediagnosis:

1	2	3	4	5
(Terrible)		undecided		**(5)** (Very good)

What number best expresses your where you think the *quality* of the retinal exam is best::

1	2	3	4	5
(Better in ophthalmologist's office)		undecided		**(5)** (Better through Telediagnosis)

What number best expresses *where* you prefer to have the retinal exam <u>now</u>:

1	2	3	4	5
(Prefer ophthalmologist's office)		undecided		**(5)** (Prefer family physician)

☐ yes ☒ no **Did the camera flashlight bother you?**

☐ yes ☒ no **Did the pupil dilating drops bother you?** ☐ not applicable

[redacted]

[redacted]

[redacted]nnaire

Name [redacted] Date [redacted]

BEFORE telediagnosis

Thank you for your willingness to participate in this research project.

You are about to undergo digital photographing and diagnosis over the internet by ophthalmologists who are retinal specialists at the University of Iowa. They will evaluate your photographs for signs of diabetic retinopathy, a complication of diabetes. This new technique is called 'telediagnosis'. Until telediagnosis was available, you could only be screened in an ophthalmologist's office with a dilated retinal exam. Please give your best answer.

What number best expresses where you think the *quality* of the retinal exam is best:

1	2	3	4	5
(Better in ophthalmologist's office)		**3** undecided		(Better through Telediagnosis)

What number best expresses *where* you prefer to have the retinal exam:

1	2	3	4	5
(Prefer ophthalmologist's office)		**3** undecided		(Prefer family physician)

☐ yes ☒ no **Do you expect the camera flashlight to bother you?**

☐ yes ☒ no **Do you expect the pupil dilating drops to bother you?**

☐ yes ☒ no **In the past five years have you been examined by an ophthalmologist?**

☒ yes ☐ no **Do you currently carry health insurance?**

☐ yes ☒ no **Sometimes photographs can only be made if your pupils are made larger with a drop. Were you aware of this?**

AFTER telediagnosis

What number best expresses your *satisfaction* with telediagnosis:

1	2	3	4	5
(Terrible)		undecided		**5** (Very good)

What number best expresses your where you think the *quality* of the retinal exam is best::

1	2	3	4	5
(Better in ophthalmologist's office)		undecided		**5** (Better through Telediagnosis)

What number best expresses *where* you prefer to have the retinal exam <u>now</u>:

1	2	3	4	5
(Prefer ophthalmologist's office)		undecided		**5** (Prefer family physician)

☐ yes ☒ no **Did the camera flashlight bother you?**

☐ yes ☒ no **Did the pupil dilating drops bother you?** ☐ not applicable

[redacted]

[redacted]

[redacted]

[redacted]uestionnaire

Name[redacted] **Date**_[redacted]

BEFORE telediagnosis

Thank you for your willingness to participate in this research project.

You are about to undergo digital photographing and diagnosis over the internet by ophthalmologists who are retinal specialists at the University of Iowa. They will evaluate your photographs for signs of diabetic retinopathy, a complication of diabetes. This new technique is called 'telediagnosis'. Until telediagnosis was available, you could only be screened in an ophthalmologist's office with a dilated retinal exam. Please give your best answer.

What number best expresses where you think the *quality* of the retinal exam is best:

1	2	3	4	5
(Better in ophthalmologist's office)		undecided **(3)**		(Better through Telediagnosis)

What number best expresses *where* you prefer to have the retinal exam:

1	2	3	4	5
(Prefer ophthalmologist's office)		undecided **(3)**		(Prefer family physician)

☐ yes ☒ no **Do you expect the camera flashlight to bother you?**

☐ yes ☒ no **Do you expect the pupil dilating drops to bother you?**

☒ yes ☐ no **In the past five years have you been examined by an ophthalmologist?**

☒ yes ☐ no **Do you currently carry health insurance?**

☒ yes ☐ no **Sometimes photographs can only be made if your pupils are made larger with a drop. Were you aware of this?**

AFTER telediagnosis

What number best expresses your *satisfaction* with telediagnosis:

1	2	3	4	5
(Terrible)		undecided		(Very good) **(5)**

What number best expresses your where you think the *quality* of the retinal exam is best::

1	2	3	4	5
(Better in ophthalmologist's office)		undecided		(Better through Telediagnosis) **(5)**

What number best expresses *where* you prefer to have the retinal exam <u>now</u>:

1	2	3	4	5
(Prefer ophthalmologist's office)		undecided	**(4)**	(Prefer family physician)

☐ yes ☒ no **Did the camera flashlight bother you?**

☐ yes ☒ no **Did the pupil dilating drops bother you?** ☐ not applicable

[redacted]

[redacted]

Patient Questionnaire

Name [redacted] Date [redacted]

BEFORE telediagnosis

Thank you for your willingness to participate in this research project.

You are about to undergo digital photographing and diagnosis over the internet by ophthalmologists who are retinal specialists at the University of Iowa. They will evaluate your photographs for signs of diabetic retinopathy, a complication of diabetes. This new technique is called 'telediagnosis'. Until telediagnosis was available, you could only be screened in an ophthalmologist's office with a dilated retinal exam. Please give your best answer.

What number best expresses where you think the *quality* of the retinal exam is best:

1 (Better in ophthalmologist's office)	2	3 undecided	4	5 (Better through Telediagnosis)

What number best expresses *where* you prefer to have the retinal exam:

1 (Prefer ophthalmologist's office)	2	3 undecided	4	5 (Prefer family physician)

☐ yes ☐ no **Do you expect the camera flashlight to bother you?**

☐ yes ☐ no **Do you expect the pupil dilating drops to bother you?**

☑ yes ☐ no **In the past five years have you been examined by an ophthalmologist?**

☑ yes ☐ no **Do you currently carry health insurance?**

☐ yes ☑ no **Sometimes photographs can only be made if your pupils are made larger with a drop. Were you aware of this?**

AFTER telediagnosis

What number best expresses your *satisfaction* with telediagnosis:

1 (Terrible)	2	3 undecided	4	5 (Very good)

What number best expresses your where you think the *quality* of the retinal exam ~~is best~~::

1 (Better in ophthalmologist's office)	2	3 undecided	4	5 (Better through Telediagnosis)

What number best expresses *where* you prefer to have the retinal exam now:

1 (Prefer ophthalmologist's office)	2	3 undecided	4	5 (Prefer family physician)

☐ yes ☑ no **Did the camera flashlight bother you?**

☐ yes ☐ no **Did the pupil dilating drops bother you?** ☑ not applicable

[redacted]

[redacted]

Patient Questionnaire

Name [redacted]________ **Date**_[redacted]

BEFORE telediagnosis

Thank you for your willingness to participate in this research project.

You are about to undergo digital photographing and diagnosis over the internet by ophthalmologists who are retinal specialists at the University of Iowa. They will evaluate your photographs for signs of diabetic retinopathy, a complication of diabetes. This new technique is called 'telediagnosis'. Until telediagnosis was available, you could only be screened in an ophthalmologist's office with a dilated retinal exam. Please give your best answer.

What number best expresses where you think the *quality* of the retinal exam is best:

1	2	3	4	5
(Better in ophthalmologist's office)		undecided		(Better through Telediagnosis)

What number best expresses *where* you prefer to have the retinal exam:

1	2	3	4	5
(Prefer ophthalmologist's office)		undecided		(Prefer family physician)

☐ yes ☒ no **Do you expect the camera flashlight to bother you?**

☐ yes ☒ no **Do you expect the pupil dilating drops to bother you?**

☐ yes ☒ no **In the past five years have you been examined by an ophthalmologist?**

☒ yes ☐ no **Do you currently carry health insurance?**

☒ yes ☐ no **Sometimes photographs can only be made if your pupils are made larger with a drop. Were you aware of this?**

AFTER telediagnosis

What number best expresses your *satisfaction* with telediagnosis:

1	2	3	4	5
(Terrible)		undecided		(Very good)

What number best expresses your where you think the *quality* of the retinal exam is best::

1	2	3	4	5
(Better in ophthalmologist's office)		undecided		(Better through Telediagnosis)

What number best expresses *where* you prefer to have the retinal exam <u>now</u>:

1	2	3	4	5
(Prefer ophthalmologist's office)		undecided		(Prefer family physician)

☐ yes ☒ no **Did the camera flashlight bother you?**

☐ yes ☐ no **Did the pupil dilating drops bother you?** ☒ not applicable

[redacted]

[redacted]

Patient Questionnaire

Name [redacted] Date [redacted]

BEFORE telediagnosis

Thank you for your willingness to participate in this research project.

You are about to undergo digital photographing and diagnosis over the internet by ophthalmologists who are retinal specialists at the University of Iowa. They will evaluate your photographs for signs of diabetic retinopathy, a complication of diabetes. This new technique is called 'telediagnosis'. Until telediagnosis was available, you could only be screened in an ophthalmologist's office with a dilated retinal exam. Please give your best answer.

What number best expresses where you think the *quality* of the retinal exam is best:

1	2	3	4	5
(Better in ophthalmologist's office)		undecided		(Better through Telediagnosis)

What number best expresses *where* you prefer to have the retinal exam:

1	2	3	4	5
(Prefer ophthalmologist's office)		undecided		(Prefer family physician)

☐ yes ☑ no **Do you expect the camera flashlight to bother you?**

☑ yes ☐ no **Do you expect the pupil dilating drops to bother you?**

☑ yes ☐ no **In the past five years have you been examined by an ophthalmologist?**

☑ yes ☐ no **Do you currently carry health insurance?**

☑ yes ☐ no **Sometimes photographs can only be made if your pupils are made larger with a drop. Were you aware of this?**

AFTER telediagnosis

What number best expresses your *satisfaction* with telediagnosis:

1	2	3	4	5
(Terrible)		undecided		(Very good)

What number best expresses your where you think the *quality* of the retinal exam is best::

1	2	3	4	5
(Better in ophthalmologist's office)		undecided		(Better through Telediagnosis)

What number best expresses *where* you prefer to have the retinal exam <u>now</u>:

1	2	3	4	5
(Prefer ophthalmologist's office)		undecided		(Prefer family physician)

☐ yes ☑ no **Did the camera flashlight bother you?**

☐ yes ☐ no **Did the pupil dilating drops bother you?** ☑ not applicable

Patient Questionnaire

Name [redacted] Date [redacted]

BEFORE telediagnosis

Thank you for your willingness to participate in this research project.

You are about to undergo digital photographing and diagnosis over the internet by ophthalmologists who are retinal specialists at the University of Iowa. They will evaluate your photographs for signs of diabetic retinopathy, a complication of diabetes. This new technique is called 'telediagnosis'. Until telediagnosis was available, you could only be screened in an ophthalmologist's office with a dilated retinal exam. Please give your best answer.

What number best expresses where you think the *quality* of the retinal exam is best:

1 (Better in ophthalmologist's office)	2	3 undecided	4	5 (Better through Telediagnosis)

What number best expresses *where* you prefer to have the retinal exam:

1 (Prefer ophthalmologist's office)	2	3 undecided	4	5 (Prefer family physician)

☐ yes ☒ no Do you expect the camera flashlight to bother you?

☐ yes ☒ no Do you expect the pupil dilating drops to bother you?

☒ yes ☐ no In the past five years have you been examined by an ophthalmologist?

☒ yes ☐ no Do you currently carry health insurance?

☒ yes ☐ no Sometimes photographs can only be made if your pupils are made larger with a drop. Were you aware of this?

AFTER telediagnosis

What number best expresses your *satisfaction* with telediagnosis:

1 (Terrible)	2	3 undecided	4	5 (Very good)

What number best expresses your where you think the *quality* of the retinal exam is best::

1 (Better in ophthalmologist's office)	2	3 undecided	4	5 (Better through Telediagnosis)

What number best expresses *where* you prefer to have the retinal exam now:

1 (Prefer ophthalmologist's office)	2	3 undecided	4	5 (Prefer family physician)

☐ yes ☒ no Did the camera flashlight bother you?

☐ yes ☐ no Did the pupil dilating drops bother you? ☒ not applicable

[redacted]

Patient Questionnaire

Name [redacted] Date [redacted]

BEFORE telediagnosis

Thank you for your willingness to participate in this research project.

You are about to undergo digital photographing and diagnosis over the internet by ophthalmologists who are retinal specialists at the University of Iowa. They will evaluate your photographs for signs of diabetic retinopathy, a complication of diabetes. This new technique is called 'telediagnosis'. Until telediagnosis was available, you could only be screened in an ophthalmologist's office with a dilated retinal exam. Please give your best answer.

What number best expresses where you think the *quality* of the retinal exam is best:

1 (circled)	2	3	4	5
(Better in ophthalmologist's office)		undecided		(Better through Telediagnosis)

What number best expresses *where* you prefer to have the retinal exam:

1 (circled)	2	3	4	5
(Prefer ophthalmologist's office)		undecided		(Prefer family physician)

☐ yes ☑ no **Do you expect the camera flashlight to bother you?**

☐ yes ☑ no **Do you expect the pupil dilating drops to bother you?**

☑ yes ☐ no **In the past five years have you been examined by an ophthalmologist?**

☑ yes ☐ no **Do you currently carry health insurance?**

☑ yes ☐ no **Sometimes photographs can only be made if your pupils are made larger with a drop. Were you aware of this?**

AFTER telediagnosis

What number best expresses your *satisfaction* with telediagnosis:

1	2	3	4	5 (circled)
(Terrible)		undecided		(Very good)

What number best expresses your where you think the *quality* of the retinal exam is best::

1	2	3	4	5 (circled)
(Better in ophthalmologist's office)		undecided		(Better through Telediagnosis)

What number best expresses *where* you prefer to have the retinal exam <u>now</u>:

1	2	3	4	5 (circled)
(Prefer ophthalmologist's office)		undecided		(Prefer family physician)

☐ yes ☑ no **Did the camera flashlight bother you?**

☐ yes ☐ no **Did the pupil dilating drops bother you?** ☑ not applicable

[redacted]

[redacted]

Patient Questionnaire

Name [redacted] Date [redacted]

BEFOR [redacted]

Thank you for your willingness to participate in this research project.

You are about to undergo digital photographing and diagnosis over the internet by ophthalmologists who are retinal specialists at the University of Iowa. They will evaluate your photographs for signs of diabetic retinopathy, a complication of diabetes. This new technique is called 'telediagnosis'. Until telediagnosis was available, you could only be screened in an ophthalmologist's office with a dilated retinal exam. Please give your best answer.

What number best expresses where you think the *quality* of the retinal exam is best:

1	2	3	4	5
(Better in ophthalmologist's office)		undecided		(Better through Telediagnosis)

What number best expresses *where* you prefer to have the retinal exam:

1	2	3	4	5
(Prefer ophthalmologist's office)		undecided		(Prefer family physician)

☐ yes ☒ no **Do you expect the camera flashlight to bother you?**

☐ yes ☒ no **Do you expect the pupil dilating drops to bother you?**

☐ yes ☒ no **In the past five years have you been examined by an ophthalmologist?**

☒ yes ☐ no **Do you currently carry health insurance?**

☒ yes ☐ no **Sometimes photographs can only be made if your pupils are made larger with a drop. Were you aware of this?**

AFTER telediagnosis

What number best expresses your *satisfaction* with telediagnosis:

1	2	3	4	5
(Terrible)		undecided		(Very good)

What number best expresses your where you think the *quality* of the retinal exam is best::

1	2	3	4	5
(Better in ophthalmologist's office)		undecided		(Better through Telediagnosis)

What number best expresses *where* you prefer to have the retinal exam <u>now</u>:

1	2	3	4	5
(Prefer ophthalmologist's office)		undecided		(Prefer family physician)

☐ yes ☒ no **Did the camera flashlight bother you?**

☐ yes ☐ no **Did the pupil dilating drops bother you?** ☒ not applicable

[redacted]

Patient Questionnaire

[redacted]

Name [redacted] Date [redacted]

BEFORE telediagnosis

Thank you for your willingness to participate in this research project.

You are about to undergo digital photographing and diagnosis over the internet by ophthalmologists who are retinal specialists at the University of Iowa. They will evaluate your photographs for signs of diabetic retinopathy, a complication of diabetes. This new technique is called 'telediagnosis'. Until telediagnosis was available, you could only be screened in an ophthalmologist's office with a dilated retinal exam. Please give your best answer.

What number best expresses where you think the *quality* of the retinal exam is best:

1	2	3	4	5
(Better in ophthalmologist's office)		undecided		(Better through Telediagnosis)

What number best expresses *where* you prefer to have the retinal exam:

1	2	3	4	5
(Prefer ophthalmologist's office)		undecided		(Prefer family physician)

☐ yes ☑ no **Do you expect the camera flashlight to bother you?**

☐ yes ☑ no **Do you expect the pupil dilating drops to bother you?**

☑ yes ☐ no **In the past five years have you been examined by an ophthalmologist?**

☑ yes ☐ no **Do you currently carry health insurance?**

☑ yes ☐ no **Sometimes photographs can only be made if your pupils are made larger with a drop. Were you aware of this?**

AFTER telediagnosis

What number best expresses your *satisfaction* with telediagnosis:

1	2	3	4	5
(Terrible)		undecided		(Very good)

What number best expresses your where you think ~~the *quality*~~ of the retinal exam is best::

1	2	3	4	5
(Better in ophthalmologist's office)		undecided		(Better through Telediagnosis)

What number best expresses *where* you prefer to have the retinal exam <u>now</u>:

1	2	3	4	5
(Prefer ophthalmologist's office)		undecided		(Prefer family physician)

☐ yes ☒ no **Did the camera flashlight bother you?**

☐ yes ☐ no **Did the pupil dilating drops bother you?** ☒ not applicable

[redacted]

Patient Questionnaire

[redacted]

Name [redacted] Date [redacted]

BEFORE telediagnosis

Thank you for your willingness to participate in this research project.

You are about to undergo digital photographing and diagnosis over the internet by ophthalmologists who are retinal specialists at the University of Iowa. They will evaluate your photographs for signs of diabetic retinopathy, a complication of diabetes. This new technique is called 'telediagnosis'. Until telediagnosis was available, you could only be screened in an ophthalmologist's office with a dilated retinal exam. Please give your best answer.

What number best expresses where you think the *quality* of the retinal exam is best:

1 (Better in ophthalmologist's office)	2	3 undecided	4	5 (Better through Telediagnosis)

What number best expresses *where* you prefer to have the retinal exam:

1 (Prefer ophthalmologist's office)	2	3 undecided	4	5 (Prefer family physician)

☐ yes ☑ no **Do you expect the camera flashlight to bother you?**

☐ yes ☑ no **Do you expect the pupil dilating drops to bother you?**

☑ yes ☐ no **In the past five years have you been examined by an ophthalmologist?**

☑ yes ☐ no **Do you currently carry health insurance?**

☑ yes ☐ no **Sometimes photographs can only be made if your pupils are made larger with a drop. Were you aware of this?**

AFTER telediagnosis

What number best expresses your *satisfaction* with telediagnosis:

1 (Terrible)	2	3 undecided	4	5 (Very good)

What number best expresses your where you think the *quality* of the retinal exam is best::

1 (Better in ophthalmologist's office)	2	3 undecided	4	5 (Better through Telediagnosis)

What number best expresses *where* you prefer to have the retinal exam now:

1 (Prefer ophthalmologist's office)	2	3 undecided	4	5 (Prefer family physician)

☐ yes ☑ no **Did the camera flashlight bother you?**

☐ yes ☐ no **Did the pupil dilating drops bother you?** ☑ not applicable

[redacted]

Patient Questionnaire

Name [redacted] Date [redacted]

BEFORE telediagnosis

Thank you for your willingness to participate in this research project.

You are about to undergo digital photographing and diagnosis over the internet by ophthalmologists who are retinal specialists at the University of Iowa. They will evaluate your photographs for signs of diabetic retinopathy, a complication of diabetes. This new technique is called 'telediagnosis'. Until telediagnosis was available, you could only be screened in an ophthalmologist's office with a dilated retinal exam. Please give your best answer.

What number best expresses where you think the *quality* of the retinal exam is best:

1 (Better in ophthalmologist's office)	2	3 undecided	4	5 (Better through Telediagnosis)

What number best expresses *where* you prefer to have the retinal exam:

1 (Prefer ophthalmologist's office)	2	3 undecided	4	5 (Prefer family physician)

☐ yes ☒ no **Do you expect the camera flashlight to bother you?**

☐ yes ☒ no **Do you expect the pupil dilating drops to bother you?**

☒ yes ☐ no **In the past five years have you been examined by an ophthalmologist?**

☒ yes ☐ no **Do you currently carry health insurance?**

☒ yes ☐ no **Sometimes photographs can only be made if your pupils are made larger with a drop. Were you aware of this?**

AFTER telediagnosis

What number best expresses your *satisfaction* with telediagnosis:

1 (Terrible)	2	3 undecided	4	5 (Very good)

What number best expresses your where you think the *quality* of the retinal exam is best::

1 (Better in ophthalmologist's office)	2	3 undecided	4	5 (Better through Telediagnosis)

What number best expresses *where* you prefer to have the retinal exam <u>now</u>:

1 (Prefer ophthalmologist's office)	2	3 undecided	4	5 (Prefer family physician)

☐ yes ☒ no **Did the camera flashlight bother you?**

☐ yes ☐ no **Did the pupil dilating drops bother you?** ☒ not applicable

[redacted]

Patient Questionnaire

Name [redacted] Date [redacted]

BEFORE telediagnosis

Thank you for your willingness to participate in this research project.

You are about to undergo digital photographing and diagnosis over the internet by ophthalmologists who are retinal specialists at the University of Iowa. They will evaluate your photographs for signs of diabetic retinopathy, a complication of diabetes. This new technique is called 'telediagnosis'. Until telediagnosis was available, you could only be screened in an ophthalmologist's office with a dilated retinal exam. Please give your best answer.

What number best expresses where you think the *quality* of the retinal exam is best:

1	2	3	4	5
(Better in ophthalmologist's office)		undecided		(Better through Telediagnosis)

What number best expresses *where* you prefer to have the retinal exam:

1	2	3	4	5
(Prefer ophthalmologist's office)		undecided		(Prefer family physician)

☐ yes ☐ no **Do you expect the camera flashlight to bother you?**

☐ yes ☐ no **Do you expect the pupil dilating drops to bother you?**

☐ yes ☐ no **In the past five years have you been examined by an ophthalmologist?**

☐ yes ☐ no **Do you currently carry health insurance?**

☐ yes ☐ no **Sometimes photographs can only be made if your pupils are made larger with a drop. Were you aware of this?**

AFTER telediagnosis

What number best expresses your *satisfaction* with telediagnosis:

1	2	3	4	5
(Terrible)		undecided		(Very good)

What number best expresses your where you think the *quality* of the retinal exam is best::

1	2	3	4	5
(Better in ophthalmologist's office)		undecided		(Better through Telediagnosis)

What number best expresses *where* you prefer to have the retinal exam <u>now</u>:

1	2	3	4	5
(Prefer ophthalmologist's office)		undecided		(Prefer family physician)

☐ yes ☐ no **Did the camera flashlight bother you?**

☐ yes ☐ no **Did the pupil dilating drops bother you?** ☐ not applicable

[redacted]

Patient Questionnaire

Name [redacted] Date [redacted]

BEFORE telediagnosis

Thank you for your willingness to participate in this research project.

You are about to undergo digital photographing and diagnosis over the internet by ophthalmologists who are retinal specialists at the University of Iowa. They will evaluate your photographs for signs of diabetic retinopathy, a complication of diabetes. This new technique is called 'telediagnosis'. Until telediagnosis was available, you could only be screened in an ophthalmologist's office with a dilated retinal exam. Please give your best answer.

What number best expresses where you think the *quality* of the retinal exam is best:

1	2	3	4	5
(Better in ophthalmologist's office)		undecided		(Better through Telediagnosis)

What number best expresses *where* you prefer to have the retinal exam:

1	2	3	4	5
(Prefer ophthalmologist's office)		undecided		(Prefer family physician)

☐ yes ☒ no **Do you expect the camera flashlight to bother you?**

☐ yes ☐ no **Do you expect the pupil dilating drops to bother you?**

☐ yes ☒ no **In the past five years have you been examined by an ophthalmologist?**

☒ yes ☐ no **Do you currently carry health insurance?**

☒ yes ☐ no **Sometimes photographs can only be made if your pupils are made larger with a drop. Were you aware of this?**

AFTER telediagnosis

What number best expresses your *satisfaction* with telediagnosis:

1	2	3	4	5
(Terrible)		undecided		(Very good)

What number best expresses your where you think the *quality* of the retinal exam is best::

1	2	3	4	5
(Better in ophthalmologist's office)		undecided		(Better through Telediagnosis)

What number best expresses *where* you prefer to have the retinal exam now:

1	2	3	4	5
(Prefer ophthalmologist's office)		undecided		(Prefer family physician)

☐ yes ☒ no **Did the camera flashlight bother you?**

☐ yes ☐ no **Did the pupil dilating drops bother you?** ☒ not applicable

[redacted]

Patient Questionnaire

Name [redacted]

Date [redacted]

BEFORE telediagnosis

Thank you for your willingness to participate in this research project.

You are about to undergo digital photographing and diagnosis over the internet by ophthalmologists who are retinal specialists at the University of Iowa. They will evaluate your photographs for signs of diabetic retinopathy, a complication of diabetes. This new technique is called 'telediagnosis'. Until telediagnosis was available, you could only be screened in an ophthalmologist's office with a dilated retinal exam. Please give your best answer.

What number best expresses where you think the *quality* of the retinal exam is best:

1	2	3	4	5
(Better in ophthalmologist's office)		undecided		(Better through Telediagnosis)

What number best expresses *where* you prefer to have the retinal exam:

1	2	3	4	5
(Prefer ophthalmologist's office)		undecided		(Prefer family physician)

☐ yes ☒ no **Do you expect the camera flashlight to bother you?**

☐ yes ☒ no **Do you expect the pupil dilating drops to bother you?**

☐ yes ☒ no **In the past five years have you been examined by an ophthalmologist?**

☒ yes ☐ no **Do you currently carry health insurance?**

☒ yes ☐ no **Sometimes photographs can only be made if your pupils are made larger with a drop. Were you aware of this?**

AFTER telediagnosis

What number best expresses your *satisfaction* with telediagnosis:

1	2	3	4	5
(Terrible)		undecided		(Very good)

What number best expresses your where you think the *quality* of the retinal exam is best::

1	2	3	4	5
(Better in ophthalmologist's office)		undecided		(Better through Telediagnosis)

What number best expresses *where* you prefer to have the retinal exam <u>now</u>:

1	2	3	4	5
(Prefer ophthalmologist's office)		undecided		(Prefer family physician)

☐ yes ☒ no **Did the camera flashlight bother you?**

☐ yes ☐ no **Did the pupil dilating drops bother you?** ☒ not applicable

[redacted]

Patient Questionnaire

Name [redacted] Date [redacted]

BEFORE telediagnosis

Thank you for your willingness to participate in this research project.

You are about to undergo digital photographing and diagnosis over the internet by ophthalmologists who are retinal specialists at the University of Iowa. They will evaluate your photographs for signs of diabetic retinopathy, a complication of diabetes. This new technique is called 'telediagnosis'. Until telediagnosis was available, you could only be screened in an ophthalmologist's office with a dilated retinal exam. Please give your best answer.

What number best expresses where you think the *quality* of the retinal exam is best:

1	2	3	4	5
(Better in ophthalmologist's office)		undecided		(Better through Telediagnosis)

What number best expresses *where* you prefer to have the retinal exam:

1	2	3	4	5
(Prefer ophthalmologist's office)		undecided		(Prefer family physician)

☐ yes ☐ no **Do you expect the camera flashlight to bother you?**

unsure

☐ yes ☐ no **Do you expect the pupil dilating drops to bother you?**

☐ yes ☐ no **In the past five years have you been examined by an ophthalmologist?**

☐ yes ☐ no **Do you currently carry health insurance?**

☐ yes ☐ no **Sometimes photographs can only be made if your pupils are made larger with a drop. Were you aware of this?**

AFTER telediagnosis

What number best expresses your *satisfaction* with telediagnosis:

1	2	3	4	5
(Terrible)		undecided		(Very good)

What number best expresses your where you think the *quality* of the retinal exam is best::

1	2	3	4	5
(Better in ophthalmologist's office)		undecided		(Better through Telediagnosis)

What number best expresses *where* you prefer to have the retinal exam now:

1	2	3	4	5
(Prefer ophthalmologist's office)		undecided		(Prefer family physician)

☐ yes ☐ no **Did the camera flashlight bother you?**

☐ yes ☐ no **Did the pupil dilating drops bother you?** ☐ not applicable

Patient Questionnaire

Name [redacted] Date [redacted]

BEFORE telediagnosis

Thank you for your willingness to participate in this research project.

You are about to undergo digital photographing and diagnosis over the internet by ophthalmologists who are retinal specialists at the University of Iowa. They will evaluate your photographs for signs of diabetic retinopathy, a complication of diabetes. This new technique is called 'telediagnosis'. Until telediagnosis was available, you could only be screened in an ophthalmologist's office with a dilated retinal exam. Please give your best answer.

What number best expresses where you think the *quality* of the retinal exam is best:

1	2	3	4	5
(Better in ophthalmologist's office)		undecided		(Better through Telediagnosis)

What number best expresses *where* you prefer to have the retinal exam:

1	2	3	4	5
(Prefer ophthalmologist's office)		undecided		(Prefer family physician)

☐ yes ☒ no **Do you expect the camera flashlight to bother you?**

☐ yes ☒ no **Do you expect the pupil dilating drops to bother you?**

☐ yes ☒ no **In the past five years have you been examined by an ophthalmologist?**

☒ yes ☐ no **Do you currently carry health insurance?**

☒ yes ☐ no **Sometimes photographs can only be made if your pupils are made larger with a drop. Were you aware of this?**

AFTER telediagnosis

What number best expresses your *satisfaction* with telediagnosis:

1	2	3	4	5
(Terrible)		undecided		(Very good)

What number best expresses your where you think the *quality* of the retinal exam is best::

1	2	3	4	5
(Better in ophthalmologist's office)		undecided		(Better through Telediagnosis)

What number best expresses *where* you prefer to have the retinal exam <u>now</u>:

1	2	3	4	5
(Prefer ophthalmologist's office)		undecided		(Prefer family physician)

☐ yes ☒ no **Did the camera flashlight bother you?**

☐ yes ☐ no **Did the pupil dilating drops bother you?** ☒ not applicable

[redacted]

[redacted]

Patient Questionnaire

Name [redacted] Date [redacted]

BEFORE telediagnosis

Thank you for your willingness to participate in this research project.

You are about to undergo digital photographing and diagnosis over the internet by ophthalmologists who are retinal specialists at the University of Iowa. They will evaluate your photographs for signs of diabetic retinopathy, a complication of diabetes. This new technique is called 'telediagnosis'. Until telediagnosis was available, you could only be screened in an ophthalmologist's office with a dilated retinal exam. Please give your best answer.

What number best expresses where you think the *quality* of the retinal exam is best:

1	2	3	4	5
(Better in ophthalmologist's office)		undecided		(Better through Telediagnosis)

What number best expresses *where* you prefer to have the retinal exam:

1	2	3	4	5
(Prefer ophthalmologist's office)		undecided		(Prefer family physician)

☐ yes ☑ no **Do you expect the camera flashlight to bother you?**

☐ yes ☑ no **Do you expect the pupil dilating drops to bother you?**

☑ yes ☐ no **In the past five years have you been examined by an ophthalmologist?**

☑ yes ☐ no **Do you currently carry health insurance?**

☑ yes ☐ no **Sometimes photographs can only be made if your pupils are made larger with a drop. Were you aware of this?**

AFTER telediagnosis

What number best expresses your *satisfaction* with telediagnosis:

1	2	3	4	5
(Terrible)		undecided		(Very good)

What number best expresses your where you think the *quality* of the retinal exam is best::

1	2	3	4	5
(Better in ophthalmologist's office)		undecided		(Better through Telediagnosis)

What number best expresses *where* you prefer to have the retinal exam <u>now</u>:

1	2	3	4	5
(Prefer ophthalmologist's office)		undecided		(Prefer family physician)

☐ yes ☒ no **Did the camera flashlight bother you?**

☐ yes ☐ no **Did the pupil dilating drops bother you?** ☒ not applicable

[redacted]nnaire

Name [redacted] Date [redacted]

BEFORE telediagnosis

Thank you for your willingness to participate in this research project.

You are about to undergo digital photographing and diagnosis over the internet by ophthalmologists who are retinal specialists at the University of Iowa. They will evaluate your photographs for signs of diabetic retinopathy, a complication of diabetes. This new technique is called 'telediagnosis'. Until telediagnosis was available, you could only be screened in an ophthalmologist's office with a dilated retinal exam. Please give your best answer.

What number best expresses where you think the *quality* of the retinal exam is best:

1	2	3	4	5
(Better in ophthalmologist's office)		undecided		(Better through Telediagnosis)

What number best expresses *where* you prefer to have the retinal exam:

1	2	3	4	5
(Prefer ophthalmologist's office)		undecided		(Prefer family physician)

☐ yes ☑ no **Do you expect the camera flashlight to bother you?**

☐ yes ☑ no **Do you expect the pupil dilating drops to bother you?**

☐ yes ☑ no **In the past five years have you been examined by an ophthalmologist?**

☐ yes ☑ no **Do you currently carry health insurance?**

☑ yes ☐ no **Sometimes photographs can only be made if your pupils are made larger with a drop. Were you aware of this?**

AFTER telediagnosis

What number best expresses your *satisfaction* with telediagnosis:

1	2	3	4	5
(Terrible)		undecided		(Very good)

What number best expresses your where you think the *quality* of the retinal exam is best::

1	2	3	4	5
(Better in ophthalmologist's office)		undecided		(Better through Telediagnosis)

What number best expresses *where* you prefer to have the retinal exam <u>now</u>:

1	2	3	4	5
(Prefer ophthalmologist's office)		undecided		(Prefer family physician)

☐ yes ☑ no **Did the camera flashlight bother you?**

☐ yes ☐ no **Did the pupil dilating drops bother you?** ☑ not applicable

[redacted]

Patient Questionnaire

Name [redacted] Date [redacted]

BEFORE telediagnosis

Thank you for your willingness to participate in this research project.

You are about to undergo digital photographing and diagnosis over the internet by ophthalmologists who are retinal specialists at the University of Iowa. They will evaluate your photographs for signs of diabetic retinopathy, a complication of diabetes. This new technique is called 'telediagnosis'. Until telediagnosis was available, you could only be screened in an ophthalmologist's office with a dilated retinal exam. Please give your best answer.

What number best expresses where you think the *quality* of the retinal exam is best:

1	2	3	4	5
(Better in ophthalmologist's office)		undecided		(Better through Telediagnosis)

What number best expresses *where* you prefer to have the retinal exam:

1	2	3	4	5
(Prefer ophthalmologist's office)		undecided		(Prefer family physician)

☐ yes ☐ no **Do you expect the camera flashlight to bother you?**

☐ yes ☐ no **Do you expect the pupil dilating drops to bother you?**

☐ yes ☐ no **In the past five years have you been examined by an ophthalmologist?**

☐ yes ☐ no **Do you currently carry health insurance?**

☐ yes ☐ no **Sometimes photographs can only be made if your pupils are made larger with a drop. Were you aware of this?**

AFTER telediagnosis

What number best expresses your *satisfaction* with telediagnosis:

1	2	3	4	5
(Terrible)		undecided		(Very good)

What number best expresses your where you think the *quality* of the retinal exam is best::

1	2	3	4	5
(Better in ophthalmologist's office)		undecided		(Better through Telediagnosis)

What number best expresses *where* you prefer to have the retinal exam <u>now</u>:

1	2	3	4	5
(Prefer ophthalmologist's office)		undecided		(Prefer family physician)

☐ yes ☐ no **Did the camera flashlight bother you?**

☐ yes ☐ no **Did the pupil dilating drops bother you?** ☐ not applicable

[redacted]

[redacted]

Patient Questionnaire

[redacted]

Name [redacted] ______________ Date [redacted]

BEFORE telediagnosis

Thank you for your willingness to participate in this research project.

You are about to undergo digital photographing and diagnosis over the internet by ophthalmologists who are retinal specialists at the University of Iowa. They will evaluate your photographs for signs of diabetic retinopathy, a complication of diabetes. This new technique is called 'telediagnosis'. Until telediagnosis was available, you could only be screened in an ophthalmologist's office with a dilated retinal exam. Please give your best answer.

What number best expresses where you think the *quality* of the retinal exam is best:

1	2	3	4	5
(Better in ophthalmologist's office)		undecided		(Better through Telediagnosis)

What number best expresses *where* you prefer to have the retinal exam:

1	2	3	4	5
(Prefer ophthalmologist's office)		undecided		(Prefer family physician)

☐ yes ☐ no **Do you expect the camera flashlight to bother you?**

☐ yes ☐ no **Do you expect the pupil dilating drops to bother you?**

☐ yes ☐ no **In the past five years have you been examined by an ophthalmologist?**

☐ yes ☐ no **Do you currently carry health insurance?**

☐ yes ☐ no **Sometimes photographs can only be made if your pupils are made larger with a drop. Were you aware of this?**

AFTER telediagnosis

What number best expresses your *satisfaction* with telediagnosis:

1	2	3	4	5
(Terrible)		undecided		(Very good)

What number best expresses your where you think the *quality* of the retinal exam is best::

1	2	3	4	5
(Better in ophthalmologist's office)		undecided		(Better through Telediagnosis)

What number best expresses *where* you prefer to have the retinal exam now:

1	2	3	4	5
(Prefer ophthalmologist's office)		undecided		(Prefer family physician)

☐ yes ☐ no **Did the camera flashlight bother you?**

☐ yes ☐ no **Did the pupil dilating drops bother you?** ☐ not applicable

[redacted]

Patient Questionnaire

Name [redacted] Date [redacted]

BEFORE telediagnosis

Thank you for your willingness to participate in this research project.

You are about to undergo digital photographing and diagnosis over the internet by ophthalmologists who are retinal specialists at the University of Iowa. They will evaluate your photographs for signs of diabetic retinopathy, a complication of diabetes. This new technique is called 'telediagnosis'. Until telediagnosis was available, you could only be screened in an ophthalmologist's office with a dilated retinal exam. Please give your best answer.

What number best expresses where you think the *quality* of the retinal exam is best:

1	2	3	4	5
(Better in ophthalmologist's office)		undecided	(Better through Telediagnosis)	

What number best expresses *where* you prefer to have the retinal exam:

1	2	3	4	5
(Prefer ophthalmologist's office)		undecided		(Prefer family physician)

☐ yes ☑ no **Do you expect the camera flashlight to bother you?**

☐ yes ☑ no **Do you expect the pupil dilating drops to bother you?**

☑ yes ☐ no **In the past five years have you been examined by an ophthalmologist?**

☑ yes ☐ no **Do you currently carry health insurance?**

☑ yes ☐ no **Sometimes photographs can only be made if your pupils are made larger with a drop. Were you aware of this?**

AFTER telediagnosis

What number best expresses your *satisfaction* with telediagnosis:

1	2	3	4	5
(Terrible)		undecided		(Very good)

What number best expresses your where you think the *quality* of the retinal exam is best::

1	2	3	4	5
(Better in ophthalmologist's office)		undecided	(Better through Telediagnosis)	

What number best expresses *where* you prefer to have the retinal exam <u>now</u>:

1	2	3	4	5
(Prefer ophthalmologist's office)		undecided		(Prefer family physician)

☐ yes ☑ no **Did the camera flashlight bother you?**

☐ yes ☐ no **Did the pupil dilating drops bother you?** ☑ not applicable

Patient Questionnaire

Name [redacted] Date [redacted]

BEFORE telediagnosis

Thank you for your willingness to participate in this research project.

You are about to undergo digital photographing and diagnosis over the internet by ophthalmologists who are retinal specialists at the University of Iowa. They will evaluate your photographs for signs of diabetic retinopathy, a complication of diabetes. This new technique is called 'telediagnosis'. Until telediagnosis was available, you could only be screened in an ophthalmologist's office with a dilated retinal exam. Please give your best answer.

What number best expresses where you think the *quality* of the retinal exam is best:

1 (Better in ophthalmologist's office)	2	3 undecided	4	5 (Better through Telediagnosis)

What number best expresses *where* you prefer to have the retinal exam:

1 (Prefer ophthalmologist's office)	2	3 undecided	4	5 (Prefer family physician)

☐ yes ☒ no **Do you expect the camera flashlight to bother you?**

☒ yes ☐ no **Do you expect the pupil dilating drops to bother you?**

☒ yes ☐ no **In the past five years have you been examined by an ophthalmologist?**

☒ yes ☐ no **Do you currently carry health insurance?**

☒ yes ☐ no **Sometimes photographs can only be made if your pupils are made larger with a drop. Were you aware of this?**

AFTER telediagnosis

What number best expresses your *satisfaction* with telediagnosis:

1 (Terrible)	2	3 undecided	4	5 (Very good)

What number best expresses your where you think the *quality* of the retinal exam is best::

1 (Better in ophthalmologist's office)	2	3 undecided	4	5 (Better through Telediagnosis)

What number best expresses *where* you prefer to have the retinal exam <u>now</u>:

1 (Prefer ophthalmologist's office)	2	3 undecided	4	5 (Prefer family physician)

☐ yes ☒ no **Did the camera flashlight bother you?**

☐ yes ☐ no **Did the pupil dilating drops bother you?** ☒ not applicable

[redacted]

Patient questionnaire [redacted]

[redacted]

Name: [redacted]

Thank you for your willingness to participate in this research project.
You are about to undergo digital photographing and diagnosis over the internet by ophthalmologists who are retinal specialists at the University of Iowa. They will evaluate your photographs for signs of diabetic retinopathy, a complication of diabetes. This new technique is called 'telediagnosis'. Until telediagnosis was available, you could only be screened in an ophthalmologist's office with a dilated retinal exam.

Please give your best answer to the following questions

Circle where you expect the quality of the retinal exam to be better:

1	2	3	4	5
Ophthalmologist's office			Telediagnosis	

Circle which retinal exam you prefer:

1	2	3	4	5
Ophthalmologist's office			Telediagnosis	

☐ yes ☐ no Do you expect the camera flashlight to bother you
☐ yes ☐ no Do you expect the pupil dilating drops to bother you
☐ yes ☐ no In the past five years have you been examined by an ophthalmologist
☐ yes ☐ no Do you currently carry health insurance
☐ yes ☐ no In some cases the photographs can only be made if your pupils are dilated with a drop. Were you aware of this?

Patient questionnaire

AFTER telediagnosis results have been discussed

Name:____________________ **Date:**____________

Circle your satisfaction with digital photography of your retina

1	2	3	4	5
Terrible				Very good

Circle where you think the retinal exam is better:

1	2	3	4	5
Ophthalmologist's office				Telediagnosis

Circle which retinal exam you prefer now:

1	2	3	4	5
Ophthalmologist's office				Telediagnosis

☐ yes ☐ no Did the camera flashlight bother you
☐ yes ☐ no Did the pupil dilating drops bother you [redacted]

After form is completed, please enter into Excel spreadsheet or send to:

Dr. Michael D. Abramoff, MD, PhD
Department of Ophthalmology and Visual Sciences
PFP 11290C
University of Iowa Hospitals and Clinics
200 Hawkins, Iowa City, IA 52242
Tel (319) 384 5833, fax: (319) 353 7996

[redacted]

[redacted]

Patient Questionnaire

Name [redacted] Date [redacted]

BEFORE telediagnosis

Thank you for your willingness to participate in this research project.

You are about to undergo digital photographing and diagnosis over the internet by ophthalmologists who are retinal specialists at the University of Iowa. They will evaluate your photographs for signs of diabetic retinopathy, a complication of diabetes. This new technique is called 'telediagnosis'. Until telediagnosis was available, you could only be screened in an ophthalmologist's office with a dilated retinal exam. Please give your best answer.

What number best expresses where you think the *quality* of the retinal exam is best:

1 (Better in ophthalmologist's office)	2	3 undecided	4	5 (Better through Telediagnosis)

What number best expresses *where* you prefer to have the retinal exam:

1 (Prefer ophthalmologist's office)	2	3 undecided	4	5 (Prefer family physician)

☐ yes ☒ no **Do you expect the camera flashlight to bother you?**

☐ yes ☒ no **Do you expect the pupil dilating drops to bother you?**

☐ yes ☒ no **In the past five years have you been examined by an ophthalmologist?**

☒ yes ☐ no **Do you currently carry health insurance?**

☒ yes ☐ no **Sometimes photographs can only be made if your pupils are made larger with a drop. Were you aware of this?**

AFTER telediagnosis

What number best expresses your *satisfaction* with telediagnosis:

1 (Terrible)	2	3 undecided	4	5 (Very good)

What number best expresses your where you think the *quality* of the retinal exam is best::

1 (Better in ophthalmologist's office)	2	3 undecided	4	5 (Better through Telediagnosis)

What number best expresses *where* you prefer to have the retinal exam now:

1 (Prefer ophthalmologist's office)	2	3 undecided	4	5 (Prefer family physician)

☐ yes ☒ no **Did the camera flashlight bother you?**

☐ yes ☐ no **Did the pupil dilating drops bother you?** ☒ not applicable

Patient Questionnaire

Name [redacted] Date [redacted]

BEFORE [redacted]

Thank you for your willingness to participate in this research project.

You are about to undergo digital photographing and diagnosis over the internet by ophthalmologists who are retinal specialists at the University of Iowa. They will evaluate your photographs for signs of diabetic retinopathy, a complication of diabetes. This new technique is called 'telediagnosis'. Until telediagnosis was available, you could only be screened in an ophthalmologist's office with a dilated retinal exam. Please give your best answer.

What number best expresses where you think the *quality* of the retinal exam is best:

1	2	3	4	5
(Better in ophthalmologist's office)		undecided		(Better through Telediagnosis)

What number best expresses *where* you prefer to have the retinal exam:

1	2	3	4	5
(Prefer ophthalmologist's office)		undecided		(Prefer family physician)

☐ yes ☑ no **Do you expect the camera flashlight to bother you?**

☐ yes ☑ no **Do you expect the pupil dilating drops to bother you?**

☐ yes ☑ no **In the past five years have you been examined by an ophthalmologist?**

☑ yes ☐ no **Do you currently carry health insurance?**

☑ yes ☐ no **Sometimes photographs can only be made if your pupils are made larger with a drop. Were you aware of this?**

AFTER telediagnosis

What number best expresses your *satisfaction* with telediagnosis:

1	2	3	4	5
(Terrible)		undecided		(Very good)

What number best expresses your where you think the *quality* of the retinal exam is best::

1	2	3	4	5
(Better in ophthalmologist's office)		undecided		(Better through Telediagnosis)

What number best expresses *where* you prefer to have the retinal exam now:

1	2	3	4	5
(Prefer ophthalmologist's office)		undecided		(Prefer family physician)

☒ yes ☐ no **Did the camera flashlight bother you?**

☐ yes ☐ no **Did the pupil dilating drops bother you?** ☒ not applicable

[redacted]

[redacted]

Patient Questionnaire

Name [redacted] Date [redacted]

BEFORE telediagnosis

Thank you for your willingness to participate in this research project.

You are about to undergo digital photographing and diagnosis over the internet by ophthalmologists who are retinal specialists at the University of Iowa. They will evaluate your photographs for signs of diabetic retinopathy, a complication of diabetes. This new technique is called 'telediagnosis'. Until telediagnosis was available, you could only be screened in an ophthalmologist's office with a dilated retinal exam. Please give your best answer.

What number best expresses where you think the *quality* of the retinal exam is best:

1 (Better in ophthalmologist's office)	2	3 undecided	4	5 (Better through Telediagnosis)

What number best expresses *where* you prefer to have the retinal exam:

1 (Prefer ophthalmologist's office)	2	3 undecided	4	5 (Prefer family physician)

☐ yes ☒ no **Do you expect the camera flashlight to bother you?**

☐ yes ☒ no **Do you expect the pupil dilating drops to bother you?**

☒ yes ☐ no **In the past five years have you been examined by an ophthalmologist?**

☒ yes ☐ no **Do you currently carry health insurance?** [redacted]

☐ yes ☒ no **Sometimes photographs can only be made if your pupils are made larger with a drop. Were you aware of this?**

AFTER telediagnosis

What number best expresses your *satisfaction* with telediagnosis:

1 (Terrible)	2	3 undecided	4	5 (Very good)

What number best expresses your where you think the *quality* of the retinal exam is best::

1 (Better in ophthalmologist's office)	2	3 undecided	4	5 (Better through Telediagnosis)

What number best expresses *where* you prefer to have the retinal exam now:

1 (Prefer ophthalmologist's office)	2	3 undecided	4	5 (Prefer family physician)

☐ yes ☒ no **Did the camera flashlight bother you?**

☐ yes ☐ no **Did the pupil dilating drops bother you?** ☒ not applicable

Patient Questionnaire

Name [redacted] Date [redacted]

BEFORE telediagnosis

Thank you for your willingness to participate in this research project.

You are about to undergo digital photographing and diagnosis over the internet by ophthalmologists who are retinal specialists at the University of Iowa. They will evaluate your photographs for signs of diabetic retinopathy, a complication of diabetes. This new technique is called 'telediagnosis'. Until telediagnosis was available, you could only be screened in an ophthalmologist's office with a dilated retinal exam. Please give your best answer.

What number best expresses where you think the *quality* of the retinal exam is best:

1	2	3	4	(5)
(Better in ophthalmologist's office)		undecided		(Better through Telediagnosis)

What number best expresses *where* you prefer to have the retinal exam:

1	2	3	4	(5)
(Prefer ophthalmologist's office)		undecided		(Prefer family physician)

☐ yes ☒ no **Do you expect the camera flashlight to bother you?**

☐ yes ☒ no **Do you expect the pupil dilating drops to bother you?**

☒ yes ☐ no **In the past five years have you been examined by an ophthalmologist?**

☒ yes ☐ no **Do you currently carry health insurance?**

☒ yes ☐ no **Sometimes photographs can only be made if your pupils are made larger with a drop. Were you aware of this?**

AFTER telediagnosis

What number best expresses your *satisfaction* with telediagnosis:

1	2	3	4	(5)
(Terrible)		undecided		(Very good)

What number best expresses your where you think the *quality* of the retinal exam is best::

1	2	3	4	(5)
(Better in ophthalmologist's office)		undecided		(Better through Telediagnosis)

What number best expresses *where* you prefer to have the retinal exam <u>now</u>:

1	2	3	4	(5)
(Prefer ophthalmologist's office)		undecided		(Prefer family physician)

☐ yes ☒ no **Did the camera flashlight bother you?**

☐ yes ☒ no **Did the pupil dilating drops bother you?** ☐ not applicable

[redacted]

Patient Questionnaire

Name [redacted] Date [redacted]

BEFORE telediagnosis

Thank you for your willingness to participate in this research project.

You are about to undergo digital photographing and diagnosis over the internet by ophthalmologists who are retinal specialists at the University of Iowa. They will evaluate your photographs for signs of diabetic retinopathy, a complication of diabetes. This new technique is called 'telediagnosis'. Until telediagnosis was available, you could only be screened in an ophthalmologist's office with a dilated retinal exam. Please give your best answer.

What number best expresses where you think the *quality* of the retinal exam is best:

1	2	3	4	5
(Better in ophthalmologist's office)		undecided		(Better through Telediagnosis)

What number best expresses *where* you prefer to have the retinal exam:

1	2	3	4	5
(Prefer ophthalmologist's office)		undecided		(Prefer family physician)

☐ yes ☐ no **Do you expect the camera flashlight to bother you?**

☐ yes ☐ no **Do you expect the pupil dilating drops to bother you?**

☐ yes ☐ no **In the past five years have you been examined by an ophthalmologist?**

☐ yes ☐ no **Do you currently carry health insurance?**

☐ yes ☐ no **Sometimes photographs can only be made if your pupils are made larger with a drop. Were you aware of this?**

AFTER telediagnosis

What number best expresses your *satisfaction* with telediagnosis:

1	2	3	4	5
(Terrible)		undecided		(Very good)

What number best expresses your where you think the *quality* of the retinal exam is best::

1	2	3	4	5
(Better in ophthalmologist's office)		undecided		(Better through Telediagnosis)

What number best expresses *where* you prefer to have the retinal exam <u>now</u>:

1	2	3	4	5
(Prefer ophthalmologist's office)		undecided		(Prefer family physician)

☐ yes ☒ no **Did the camera flashlight bother you?**

☐ yes ☐ no **Did the pupil dilating drops bother you?** ☒ not applicable

[redacted]

Patient Questionnaire

Name [redacted] Date [redacted]

BEFORE telediagnosis

Thank you for your willingness to participate in this research project.

You are about to undergo digital photographing and diagnosis over the internet by ophthalmologists who are retinal specialists at the University of Iowa. They will evaluate your photographs for signs of diabetic retinopathy, a complication of diabetes. This new technique is called 'telediagnosis'. Until telediagnosis was available, you could only be screened in an ophthalmologist's office with a dilated retinal exam. Please give your best answer.

What number best expresses where you think the *quality* of the retinal exam is best:

1 (Better in ophthalmologist's office)	2	(3) undecided	4	5 (Better through Telediagnosis)

What number best expresses *where* you prefer to have the retinal exam:

1 (Prefer ophthalmologist's office)	2	(3) undecided	4	5 (Prefer family physician)

☑ yes ☐ no **Do you expect the camera flashlight to bother you?**

☑ yes ☐ no **Do you expect the pupil dilating drops to bother you?**

☑ yes ☐ no **In the past five years have you been examined by an ophthalmologist?**

☑ yes ☐ no **Do you currently carry health insurance?**

☑ yes ☐ no **Sometimes photographs can only be made if your pupils are made larger with a drop. Were you aware of this?**

AFTER telediagnosis

What number best expresses your *satisfaction* with telediagnosis:

1 (Terrible)	2	3 undecided	(4)	5 (Very good)

What number best expresses your where you think the *quality* of the retinal exam is best::

1 (Better in ophthalmologist's office)	2	(3) undecided	4	5 (Better through Telediagnosis)

What number best expresses *where* you prefer to have the retinal exam now:

1 (Prefer ophthalmologist's office)	2	(3) undecided	4	5 (Prefer family physician)

☐ yes ☑ no **Did the camera flashlight bother you?**

☐ yes ☐ no **Did the pupil dilating drops bother you?** ☑ not applicable

[redacted]

[redacted]

[redacted]tient Questionnaire

Name [redacted] Date [redacted]

BEFORE telediagnosis

Thank you for your willingness to participate in this research project.

You are about to undergo digital photographing and diagnosis over the internet by ophthalmologists who are retinal specialists at the University of Iowa. They will evaluate your photographs for signs of diabetic retinopathy, a complication of diabetes. This new technique is called 'telediagnosis'. Until telediagnosis was available, you could only be screened in an ophthalmologist's office with a dilated retinal exam. Please give your best answer.

What number best expresses where you think the *quality* of the retinal exam is best:

1	2	3	4	5
(Better in ophthalmologist's office)		undecided		(Better through Telediagnosis)

What number best expresses *where* you prefer to have the retinal exam:

1	2	3	4	5
(Prefer ophthalmologist's office)		undecided		(Prefer family physician)

☐ yes ☒ no **Do you expect the camera flashlight to bother you?**

☐ yes ☒ no **Do you expect the pupil dilating drops to bother you?**

☒ yes ☐ no **In the past five years have you been examined by an ophthalmologist?**

☒ yes ☐ no **Do you currently carry health insurance?**

☒ yes ☐ no **Sometimes photographs can only be made if your pupils are made larger with a drop. Were you aware of this?**

AFTER telediagnosis

What number best expresses your *satisfaction* with telediagnosis:

1	2	3	4	5
(Terrible)		undecided		(Very good)

What number best expresses your where you think the *quality* of the retinal exam is best::

1	2	3	4	5
(Better in ophthalmologist's office)		undecided		(Better through Telediagnosis)

What number best expresses *where* you prefer to have the retinal exam now:

1	2	3	4	5
(Prefer ophthalmologist's office)		undecided		(Prefer family physician)

☐ yes ☒ no **Did the camera flashlight bother you?**

☐ yes ☒ no **Did the pupil dilating drops bother you?** ☐ not applicable

[redacted]

[redacted]

Patient Questionnaire

Name [redacted] Date [redacted]

BEFORE telediagnosis

Thank you for your willingness to participate in this research project.

You are about to undergo digital photographing and diagnosis over the internet by ophthalmologists who are retinal specialists at the University of Iowa. They will evaluate your photographs for signs of diabetic retinopathy, a complication of diabetes. This new technique is called 'telediagnosis'. Until telediagnosis was available, you could only be screened in an ophthalmologist's office with a dilated retinal exam. Please give your best answer.

What number best expresses where you think the *quality* of the retinal exam is best:

1 (Better in ophthalmologist's office)	2	3 undecided	4	5 (Better through Telediagnosis)

What number best expresses *where* you prefer to have the retinal exam:

1 (Prefer ophthalmologist's office)	2	3 undecided	4	5 (Prefer family physician)

☐ yes ☑ no **Do you expect the camera flashlight to bother you?**

☐ yes ☑ no **Do you expect the pupil dilating drops to bother you?**

☐ yes ☑ no **In the past five years have you been examined by an ophthalmologist?**

☑ yes ☐ no **Do you currently carry health insurance?**

☑ yes ☐ no **Sometimes photographs can only be made if your pupils are made larger with a drop. Were you aware of this?**

AFTER telediagnosis

What number best expresses your *satisfaction* with telediagnosis:

1 (Terrible)	2	3 undecided	4	5 (Very good)

What number best expresses your where you think the *quality* of the retinal exam is best::

1 (Better in ophthalmologist's office)	2	3 undecided	4	5 (Better through Telediagnosis)

What number best expresses *where* you prefer to have the retinal exam <u>now</u>:

1 (Prefer ophthalmologist's office)	2	3 undecided	4	5 (Prefer family physician)

☐ yes ☑ no **Did the camera flashlight bother you?**

☐ yes ☐ no **Did the pupil dilating drops bother you?** ☑ not applicable

Patient Questionnaire

Name [redacted] Date [redacted]

BEFORE telediagnosis

Thank you for your willingness to participate in this research project.

You are about to undergo digital photographing and diagnosis over the internet by ophthalmologists who are retinal specialists at the University of Iowa. They will evaluate your photographs for signs of diabetic retinopathy, a complication of diabetes. This new technique is called 'telediagnosis'. Until telediagnosis was available, you could only be screened in an ophthalmologist's office with a dilated retinal exam. Please give your best answer.

What number best expresses where you think the *quality* of the retinal exam is best:

1	2	3	4	5
(Better in ophthalmologist's office)		undecided		**(5)** (Better through Telediagnosis)

What number best expresses *where* you prefer to have the retinal exam:

1	2	3	4	5
(Prefer ophthalmologist's office)		undecided		**(5)** (Prefer family physician)

☐ yes ☒ no **Do you expect the camera flashlight to bother you?**

☐ yes ☒ no **Do you expect the pupil dilating drops to bother you?**

☒ yes ☐ no **In the past five years have you been examined by an ophthalmologist?**

☒ yes ☐ no **Do you currently carry health insurance?**

☐ yes ☒ no **Sometimes photographs can only be made if your pupils are made larger with a drop. Were you aware of this?**

AFTER telediagnosis

What number best expresses your *satisfaction* with telediagnosis:

1	2	3	4	5
(Terrible)		undecided		**(5)** (Very good)

What number best expresses your where you think the *quality* of the retinal exam is best::

1	2	3	4	5
(Better in ophthalmologist's office)		undecided		**(5)** (Better through Telediagnosis)

What number best expresses *where* you prefer to have the retinal exam now:

1	2	3	4	5
(Prefer ophthalmologist's office)		undecided		**(5)** (Prefer family physician)

☐ yes ☒ no **Did the camera flashlight bother you?**

☐ yes ☐ no **Did the pupil dilating drops bother you?** ☒ not applicable

[redacted]

Patient Questionnaire

Name ___ [redacted]

BEFORE [redacted]

Thank you for your willingness to participate in this research project.

You are about to undergo digital photographing and diagnosis over the internet by [redacted] specialists at the University of Iowa. They will evaluate your photographs for signs of diabetic retinopathy, a complication of diabetes. This new technique is called 'telediagnosis'. Until telediagnosis was available, you could only be screened in an ophthalmologist's office with a dilated retinal exam. Please give your best answer.

What number best expresses where you think the *quality* of the retinal exam is best:

1	2	3	4	5
(Better in ophthalmologist's office)		undecided		(Better through Telediagnosis)

What number best expresses *where* you prefer to have the retinal exam:

1	2	3	4	5
(Prefer ophthalmologist's office)		undecided		(Prefer family physician)

☐ yes ☒ no Do you expect the camera flashlight to bother you?

☐ yes ☒ no Do you expect the pupil dilating drops to bother you?

☒ yes ☐ no In the past five years have you been examined by an ophthalmologist?

☒ yes ☐ no Do you currently carry health insurance?

☒ yes ☐ no Sometimes photographs can only be made if your pupils are made larger with a drop. Were you aware of this?

AFTER telediagnosis

What number best expresses your *satisfaction* with telediagnosis:

1	2	3	4	5
(Terrible)		undecided		(Very good)

What number best expresses your where you think the *quality* of the retinal exam is best::

1	2	3	4	5
(Better in ophthalmologist's office)		undecided		(Better through Telediagnosis)

What number best expresses *where* you prefer to have the retinal exam <u>now</u>:

1	2	3	4	5
(Prefer ophthalmologist's office)		undecided		(Prefer family physician)

☐ yes ☒ no Did the camera flashlight bother you?

☐ yes ☒ no Did the pupil dilating drops bother you? ☐ not applicable

[redacted]

Patient Questionnaire

Name [redacted] Date [redacted]

BEFORE telediagnosis

Thank you for your willingness to participate in this research project.

You are about to undergo digital photographing and diagnosis over the internet by ophthalmologists who are retinal specialists at the University of Iowa. They will evaluate your photographs for signs of diabetic retinopathy, a complication of diabetes. This new technique is called 'telediagnosis'. Until telediagnosis was available, you could only be screened in an ophthalmologist's office with a dilated retinal exam. Please give your best answer.

What number best expresses where you think the *quality* of the retinal exam is best:

1 (Better in ophthalmologist's office)	2	3 undecided	4	5 (Better through Telediagnosis)

What number best expresses *where* you prefer to have the retinal exam:

1 (Prefer ophthalmologist's office)	2	3 undecided	4	5 (Prefer family physician)

☐ yes ☑ no **Do you expect the camera flashlight to bother you?**

☐ yes ☑ no **Do you expect the pupil dilating drops to bother you?**

☐ yes ☑ no **In the past five years have you been examined by an ophthalmologist?**

☐ yes ☑ no **Do you currently carry health insurance?**

☐ yes ☑ no **Sometimes photographs can only be made if your pupils are made larger with a drop. Were you aware of this?**

AFTER telediagnosis

What number best expresses your *satisfaction* with telediagnosis:

1 (Terrible)	2	3 undecided	4	5 (Very good)

What number best expresses your where you think the *quality* of the retinal exam is best::

1 (Better in ophthalmologist's office)	2	3 undecided	4	5 (Better through Telediagnosis)

What number best expresses *where* you prefer to have the retinal exam <u>now</u>:

1 (Prefer ophthalmologist's office)	2	3 undecided	4	5 (Prefer family physician)

☐ yes ☑ no **Did the camera flashlight bother you?**

☐ yes ☐ no **Did the pupil dilating drops bother you?** ☑ not applicable

[redacted]

[redacted]

[redacted] Patient Questionnaire

Name [redacted]

Date [redacted]

BEFORE telediagnosis

Thank you for your willingness to participate in this research project.

You are about to undergo digital photographing and diagnosis over the internet by ophthalmologists who are retinal specialists at the University of Iowa. They will evaluate your photographs for signs of diabetic retinopathy, a complication of diabetes. This new technique is called 'telediagnosis'. Until telediagnosis was available, you could only be screened in an ophthalmologist's office with a dilated retinal exam. Please give your best answer.

What number best expresses where you think the *quality* of the retinal exam is best:

1	2	3	4	5
(Better in ophthalmologist's office)		undecided		(Better through Telediagnosis)

What number best expresses *where* you prefer to have the retinal exam:

1	2	3	4	5
(Prefer ophthalmologist's office)		undecided		(Prefer family physician)

☐ yes ☒ no **Do you expect the camera flashlight to bother you?**

☐ yes ☒ no **Do you expect the pupil dilating drops to bother you?**

☐ yes ☒ no **In the past five years have you been examined by an ophthalmologist?**

☒ yes ☐ no **Do you currently carry health insurance?**

☒ yes ☐ no **Sometimes photographs can only be made if your pupils are made larger with a drop. Were you aware of this?**

AFTER telediagnosis

What number best expresses your *satisfaction* with telediagnosis:

1	2	3	4	5
(Terrible)		undecided		(Very good)

What number best expresses your where you think the *quality* of the retinal exam is best::

1	2	3	4	5
(Better in ophthalmologist's office)		undecided		(Better through Telediagnosis)

What number best expresses *where* you prefer to have the retinal exam <u>now</u>:

1	2	3	4	5
(Prefer ophthalmologist's office)		undecided		(Prefer family physician)

☐ yes ☒ no **Did the camera flashlight bother you?**

☐ yes ☐ no **Did the pupil dilating drops bother you?** ☒ not applicable

[redacted]

Patient Questionnaire

Name [redacted] Date [redacted]

BEFORE telediagnosis

Thank you for your willingness to participate in this research project.

You are about to undergo digital photographing and diagnosis over the internet by ophthalmologists who are retinal specialists at the University of Iowa. They will evaluate your photographs for signs of diabetic retinopathy, a complication of diabetes. This new technique is called 'telediagnosis'. Until telediagnosis was available, you could only be screened in an ophthalmologist's office with a dilated retinal exam. Please give your best answer.

What number best expresses where you think the *quality* of the retinal exam is best:

1	2	3	4	5
(Better in ophthalmologist's office)		undecided		(Better through Telediagnosis)

What number best expresses *where* you prefer to have the retinal exam:

1	2	3	4	5
(Prefer ophthalmologist's office)		undecided		(Prefer family physician)

☐ yes ☐ no **Do you expect the camera flashlight to bother you?**

☐ yes ☐ no **Do you expect the pupil dilating drops to bother you?**

☐ yes ☐ no **In the past five years have you been examined by an ophthalmologist?**

☐ yes ☐ no **Do you currently carry health insurance?**

☐ yes ☐ no **Sometimes photographs can only be made if your pupils are made larger with a drop. Were you aware of this?**

AFTER telediagnosis

What number best expresses your *satisfaction* with telediagnosis:

1	2	3	4	5
(Terrible)		undecided		(Very good)

What number best expresses your where you think the *quality* of the retinal exam is best::

1	2	3	4	5
(Better in ophthalmologist's office)		undecided		(Better through Telediagnosis)

What number best expresses *where* you prefer to have the retinal exam <u>now</u>:

1	2	3	4	5
(Prefer ophthalmologist's office)		undecided		(Prefer family physician)

☐ yes ☐ no **Did the camera flashlight bother you?**

☐ yes ☐ no **Did the pupil dilating drops bother you?** ☐ not applicable

[redacted]

Patient Questionnaire

Name [redacted] ______________ Date [redacted]

BEFORE telediagnosis

Thank you for your willingness to participate in this research project.

You are about to undergo digital photographing and diagnosis over the internet by ophthalmologists who are retinal specialists at the University of Iowa. They will evaluate your photographs for signs of diabetic retinopathy, a complication of diabetes. This new technique is called 'telediagnosis'. Until telediagnosis was available, you could only be screened in an ophthalmologist's office with a dilated retinal exam. Please give your best answer.

What number best expresses where you think the *quality* of the retinal exam is best:

1	2	3	4	5
(Better in ophthalmologist's office)		undecided		(Better through Telediagnosis)

What number best expresses *where* you prefer to have the retinal exam:

1	2	3	4	5
(Prefer ophthalmologist's office)		undecided		(Prefer family physician)

☐ yes ☒ no **Do you expect the camera flashlight to bother you?**

☐ yes ☒ no **Do you expect the pupil dilating drops to bother you?**

☒ yes ☐ no **In the past five years have you been examined by an ophthalmologist?**

☒ yes ☐ no **Do you currently carry health insurance?**

☒ yes ☐ no **Sometimes photographs can only be made if your pupils are made larger with a drop. Were you aware of this?**

AFTER telediagnosis

What number best expresses your *satisfaction* with telediagnosis:

1	2	3	4	5
(Terrible)		undecided		(Very good)

What number best expresses your where you think the *quality* of the retinal exam is best::

1	2	3	4	5
(Better in ophthalmologist's office)		undecided		(Better through Telediagnosis)

What number best expresses *where* you prefer to have the retinal exam <u>now</u>:

1	2	3	4	5
(Prefer ophthalmologist's office)		undecided		(Prefer family physician)

☐ yes ☒ no **Did the camera flashlight bother you?**

☐ yes ☐ no **Did the pupil dilating drops bother you?** ☒ not applicable

Patient Questionnaire

Name [redacted] Date [redacted]

BEFORE telediagnosis

Thank you for your willingness to participate in this research project.

You are about to undergo digital photographing and diagnosis over the internet by ophthalmologists who are retinal specialists at the University of Iowa. They will evaluate your photographs for signs of diabetic retinopathy, a complication of diabetes. This new technique is called 'telediagnosis'. Until telediagnosis was available, you could only be screened in an ophthalmologist's office with a dilated retinal exam. Please give your best answer.

What number best expresses where you think the *quality* of the retinal exam is best:

1	2	3	4	5
(Better in ophthalmologist's office)		undecided		(Better through Telediagnosis)

What number best expresses *where* you prefer to have the retinal exam:

1	2	3	4	5
(Prefer ophthalmologist's office)		undecided		(Prefer family physician)

☐ yes ☐ no **Do you expect the camera flashlight to bother you?**

☐ yes ☐ no **Do you expect the pupil dilating drops to bother you?**

☐ yes ☐ no **In the past five years have you been examined by an ophthalmologist?**

☐ yes ☐ no **Do you currently carry health insurance?**

☐ yes ☐ no **Sometimes photographs can only be made if your pupils are made larger with a drop. Were you aware of this?**

AFTER telediagnosis

What number best expresses your *satisfaction* with telediagnosis:

1	2	3	4	5
(Terrible)		undecided		(Very good)

What number best expresses your where you think the *quality* of the retinal exam is best::

1	2	3	4	5
(Better in ophthalmologist's office)		undecided		(Better through Telediagnosis)

What number best expresses *where* you prefer to have the retinal exam now:

1	2	3	4	5
(Prefer ophthalmologist's office)		undecided		(Prefer family physician)

☐ yes ☐ no **Did the camera flashlight bother you?**

☐ yes ☐ no **Did the pupil dilating drops bother you?** ☐ not applicable

[redacted]

[redacted]

Patient Questionnaire

Name [redacted] Date [redacted]

BEFORE telediagnosis

Thank you for your willingness to participate in this research project.

You are about to undergo digital photographing and diagnosis over the internet by ophthalmologists who are retinal specialists at the University of Iowa. They will evaluate your photographs for signs of diabetic retinopathy, a complication of diabetes. This new technique is called 'telediagnosis'. Until telediagnosis was available, you could only be screened in an ophthalmologist's office with a dilated retinal exam. Please give your best answer.

What number best expresses where you think the *quality* of the retinal exam is best:

1	2	3	4	5
(Better in ophthalmologist's office)		undecided		(Better through Telediagnosis)

What number best expresses *where* you prefer to have the retinal exam:

1	2	3	4	5
(Prefer ophthalmologist's office)		undecided		(Prefer family physician)

☐ yes ☒ no **Do you expect the camera flashlight to bother you?**

☐ yes ☐ no **Do you expect the pupil dilating drops to bother you?**

☐ yes ☐ no **In the past five years have you been examined by an ophthalmologist?**

☐ yes ☐ no **Do you currently carry health insurance?**

☑ yes ☐ no **Sometimes photographs can only be made if your pupils are made larger with a drop. Were you aware of this?**

AFTER telediagnosis

What number best expresses your *satisfaction* with telediagnosis:

1	2	3	4	5
(Terrible)		undecided		(Very good)

What number best expresses your where you think the *quality* of the retinal exam is best::

1	2	3	4	5
(Better in ophthalmologist's office)		undecided		(Better through Telediagnosis)

What number best expresses *where* you prefer to have the retinal exam now:

1	2	3	4	5
(Prefer ophthalmologist's office)		undecided		(Prefer family physician)

☐ yes ☒ no **Did the camera flashlight bother you?**

☐ yes ☐ no **Did the pupil dilating drops bother you?** ☐ not applicable

[redacted] Patient Questionnaire

Name [redacted] Date [redacted]

BEFORE telediagnosis

Thank you for your willingness to participate in this research project.

You are about to undergo digital photographing and diagnosis over the internet by ophthalmologists who are retinal specialists at the University of Iowa. They will evaluate your photographs for signs of diabetic retinopathy, a complication of diabetes. This new technique is called 'telediagnosis'. Until telediagnosis was available, you could only be screened in an ophthalmologist's office with a dilated retinal exam. Please give your best answer.

What number best expresses where you think the *quality* of the retinal exam is best:

1	2	3 (circled)	4	5
(Better in ophthalmologist's office)		undecided		(Better through Telediagnosis)

What number best expresses *where* you prefer to have the retinal exam:

1	2	3 (circled)	4	5
(Prefer ophthalmologist's office)		undecided		(Prefer family physician)

☐ yes ☒ no **Do you expect the camera flashlight to bother you?**

☐ yes ☒ no **Do you expect the pupil dilating drops to bother you?**

☐ yes ☒ no **In the past five years have you been examined by an ophthalmologist?**

☒ yes ☐ no **Do you currently carry health insurance?**

☒ yes ☐ no **Sometimes photographs can only be made if your pupils are made larger with a drop. Were you aware of this?**

AFTER telediagnosis

What number best expresses your *satisfaction* with telediagnosis:

1	2	3	4	5 (circled)
(Terrible)		undecided		(Very good)

What number best expresses your where you think the *quality* of the retinal exam is best::

1	2	3 (circled)	4	5
(Better in ophthalmologist's office)		undecided		(Better through Telediagnosis)

What number best expresses *where* you prefer to have the retinal exam now:

1	2	3 (circled)	4	5
(Prefer ophthalmologist's office)		undecided		(Prefer family physician)

☐ yes ☒ no **Did the camera flashlight bother you?**

☐ yes ☐ no **Did the pupil dilating drops bother you?** ☒ not applicable

[redacted]

P[redacted]tient Questionnaire

Name [redacted] ________ Date [redacted]

BEFORE telediagnosis

Thank you for your willingness to participate in this research project.

You are about to undergo digital photographing and diagnosis over the internet by ophthalmologists who are retinal specialists at the University of Iowa. They will evaluate your photographs for signs of diabetic retinopathy, a complication of diabetes. This new technique is called 'telediagnosis'. Until telediagnosis was available, you could only be screened in an ophthalmologist's office with a dilated retinal exam. Please give your best answer.

What number best expresses where you think the *quality* of the retinal exam is best:

1	2	3	4	5
(Better in ophthalmologist's office)		undecided		(Better through Telediagnosis)

What number best expresses *where* you prefer to have the retinal exam:

1	2	3	4	5
(Prefer ophthalmologist's office)		undecided		(Prefer family physician)

☐ yes ☑ no **Do you expect the camera flashlight to bother you?**

☐ yes ☑ no **Do you expect the pupil dilating drops to bother you?**

☑ yes ☐ no **In the past five years have you been examined by an ophthalmologist?**

☑ yes ☐ no **Do you currently carry health insurance?**

☑ yes ☐ no **Sometimes photographs can only be made if your pupils are made larger with a drop. Were you aware of this?**

AFTER telediagnosis

What number best expresses your *satisfaction* with telediagnosis:

1	2	3	4	5
(Terrible)		undecided		(Very good)

What number best expresses your where you think the *quality* of the retinal exam is best::

1	2	3	4	5
(Better in ophthalmologist's office)		undecided		(Better through Telediagnosis)

What number best expresses *where* you prefer to have the retinal exam <u>now</u>:

1	2	3	4	5
(Prefer ophthalmologist's office)		undecided		(Prefer family physician)

☐ yes ☑ no **Did the camera flashlight bother you?**

☐ yes ☐ no **Did the pupil dilating drops bother you?** ☑ not applicable

[redacted]

Patient Questionnaire

Name [redacted] Date [redacted]

BEFORE telediagnosis

Thank you for your willingness to participate in this research project.

You are about to undergo digital photographing and diagnosis over the internet by ophthalmologists who are retinal specialists at the University of Iowa. They will evaluate your photographs for signs of diabetic retinopathy, a complication of diabetes. This new technique is called 'telediagnosis'. Until telediagnosis was available, you could only be screened in an ophthalmologist's office with a dilated retinal exam. Please give your best answer.

What number best expresses where you think the *quality* of the retinal exam is best:

1	2	3	4	5
(Better in ophthalmologist's office)		undecided		(Better through Telediagnosis)

What number best expresses *where* you prefer to have the retinal exam:

1	2	3	4	5
(Prefer ophthalmologist's office)		undecided		(Prefer family physician)

☐ yes ☒ no **Do you expect the camera flashlight to bother you?**

☐ yes ☒ no **Do you expect the pupil dilating drops to bother you?**

☒ yes ☐ no **In the past five years have you been examined by an ophthalmologist?**

[redacted] ☒ yes ☐ no **Do you currently carry health insurance?**

☒ yes ☐ no **Sometimes photographs can only be made if your pupils are made larger with a drop. Were you aware of this?**

AFTER telediagnosis

What number best expresses your *satisfaction* with telediagnosis:

1	2	3	4	5
(Terrible)		undecided		(Very good)

What number best expresses your where you think the *quality* of the retinal exam is best::

1	2	3	4	5
(Better in ophthalmologist's office)		undecided		(Better through Telediagnosis)

What number best expresses *where* you prefer to have the retinal exam now:

1	2	3	4	5
(Prefer ophthalmologist's office)		undecided		(Prefer family physician)

☐ yes ☒ no **Did the camera flashlight bother you?**

☐ yes ☒ no **Did the pupil dilating drops bother you?** ☐ not applicable

[redacted]

Patient Questionnaire

Name [redacted] Date [redacted]

BEFORE telediagnosis

Thank you for your willingness to participate in this research project.

You are about to undergo digital photographing and diagnosis over the internet by ophthalmologists who are retinal specialists at the University of Iowa. They will evaluate your photographs for signs of diabetic retinopathy, a complication of diabetes. This new technique is called 'telediagnosis'. Until telediagnosis was available, you could only be screened in an ophthalmologist's office with a dilated retinal exam. Please give your best answer.

What number best expresses where you think the *quality* of the retinal exam is best:

1	2	3	4	5
(Better in ophthalmologist's office)		undecided		(Better through Telediagnosis)

What number best expresses *where* you prefer to have the retinal exam:

1	2	3	4	5
(Prefer ophthalmologist's office)		undecided		(Prefer family physician)

☐ yes ☑ no **Do you expect the camera flashlight to bother you?**

☐ yes ☑ no **Do you expect the pupil dilating drops to bother you?**

☑ yes ☐ no **In the past five years have you been examined by an ophthalmologist?**

☑ yes ☐ no **Do you currently carry health insurance?**

☑ yes ☐ no **Sometimes photographs can only be made if your pupils are made larger with a drop. Were you aware of this?**

AFTER telediagnosis

What number best expresses your *satisfaction* with telediagnosis:

1	2	3	4	5
(Terrible)		undecided		(Very good)

What number best expresses your where you think the *quality* of the retinal exam is best::

1	2	3	4	5
(Better in ophthalmologist's office)		undecided		(Better through Telediagnosis)

What number best expresses *where* you prefer to have the retinal exam <u>now</u>:

1	2	3	4	5
(Prefer ophthalmologist's office)		undecided		(Prefer family physician)

☐ yes ☑ no **Did the camera flashlight bother you?**

☐ yes ☐ no **Did the pupil dilating drops bother you?** ☑ not applicable

[redacted]

Patient Questionnaire

Name [redacted] Date [redacted]

BEFORE telediagnosis

Thank you for your willingness to participate in this research project.

You are about to undergo digital photographing and diagnosis over the internet by ophthalmologists who are retinal specialists at the University of Iowa. They will evaluate your photographs for signs of diabetic retinopathy, a complication of diabetes. This new technique is called 'telediagnosis'. Until telediagnosis was available, you could only be screened in an ophthalmologist's office with a dilated retinal exam. Please give your best answer.

What number best expresses where you think the *quality* of the retinal exam is best:

1	2	3	4	5
(Better in ophthalmologist's office)		undecided		(Better through Telediagnosis)

What number best expresses *where* you prefer to have the retinal exam:

1	2	3	4	5
(Prefer ophthalmologist's office)		undecided		(Prefer family physician)

☐ yes ☒ no **Do you expect the camera flashlight to bother you?**

☐ yes ☒ no **Do you expect the pupil dilating drops to bother you?**

☐ yes ☒ no **In the past five years have you been examined by an ophthalmologist?**

☒ yes ☒ no **Do you currently carry health insurance?**

☒ yes ☐ no **Sometimes photographs can only be made if your pupils are made larger with a drop. Were you aware of this?**

AFTER telediagnosis

What number best expresses your *satisfaction* with telediagnosis:

1	2	3	4	5
(Terrible)		undecided		(Very good)

What number best expresses your where you think the *quality* of the retinal exam is best::

1	2	3	4	5
(Better in ophthalmologist's office)		undecided		(Better through Telediagnosis)

What number best expresses *where* you prefer to have the retinal exam now:

1	2	3	4	5
(Prefer ophthalmologist's office)		undecided		(Prefer family physician)

☐ yes ☒ no **Did the camera flashlight bother you?**

☐ yes ☐ no **Did the pupil dilating drops bother you?** ☒ not applicable

[redacted]

Patient Questionnaire

Name [redacted] Date [redacted]

BEFORE telediagnosis

Thank you for your willingness to participate in this research project.

You are about to undergo digital photographing and diagnosis over the internet by ophthalmologists who are retinal specialists at the University of Iowa. They will evaluate your photographs for signs of diabetic retinopathy, a complication of diabetes. This new technique is called 'telediagnosis'. Until telediagnosis was available, you could only be screened in an ophthalmologist's office with a dilated retinal exam. Please give your best answer.

What number best expresses where you think the *quality* of the retinal exam is best:

1 (Better in ophthalmologist's office)	2	3 undecided	4	5 (Better through Telediagnosis)

What number best expresses *where* you prefer to have the retinal exam:

1 (Prefer ophthalmologist's office)	2	3 undecided	4	5 (Prefer family physician)

☐ yes ☑ no **Do you expect the camera flashlight to bother you?**

☐ yes ☐ no **Do you expect the pupil dilating drops to bother you?**

☑ yes ☐ no **In the past five years have you been examined by an ophthalmologist?**

☐ yes ☐ no **Do you currently carry health insurance?**

☐ yes ☑ no **Sometimes photographs can only be made if your pupils are made larger with a drop. Were you aware of this?**

AFTER telediagnosis

What number best expresses your *satisfaction* with telediagnosis:

1 (Terrible)	2	3 undecided	4	5 (Very good)

What number best expresses your where you think the *quality* of the retinal exam is best::

1 (Better in ophthalmologist's office)	2	3 undecided	4	5 (Better through Telediagnosis)

What number best expresses *where* you prefer to have the retinal exam now:

1 (Prefer ophthalmologist's office)	2	3 undecided	4	5 (Prefer family physician)

☑ yes ☐ no **Did the camera flashlight bother you?**

☐ yes ☐ no **Did the pupil dilating drops bother you?** ☒ not applicable

Patient Questionnaire

Name [redacted] Date [redacted]

BEFORE telediagnosis

Thank you for your willingness to participate in this research project.

You are about to undergo digital photographing and diagnosis over the internet by ophthalmologists who are retinal specialists at the University of Iowa. They will evaluate your photographs for signs of diabetic retinopathy, a complication of diabetes. This new technique is called 'telediagnosis'. Until telediagnosis was available, you could only be screened in an ophthalmologist's office with a dilated retinal exam. Please give your best answer.

What number best expresses where you think the *quality* of the retinal exam is best:

1	2	3	4	5
(Better in ophthalmologist's office)		undecided	(Better through Telediagnosis)	

What number best expresses *where* you prefer to have the retinal exam:

1	2	3	4	5
(Prefer ophthalmologist's office)		undecided	(Prefer family physician)	

☐ yes ☒ no **Do you expect the camera flashlight to bother you?**

☐ yes ☒ no **Do you expect the pupil dilating drops to bother you?**

☒ yes ☐ no **In the past five years have you been examined by an ophthalmologist?**

☒ yes ☐ no **Do you currently carry health insurance?**

☒ yes ☐ no **Sometimes photographs can only be made if your pupils are made larger with a drop. Were you aware of this?**

AFTER telediagnosis

What number best expresses your *satisfaction* with telediagnosis:

1	2	3	4	5
(Terrible)		undecided		(Very good)

What number best expresses your where you think the *quality* of the retinal exam is best::

1	2	3	4	5
(Better in ophthalmologist's office)		undecided	(Better through Telediagnosis)	

What number best expresses *where* you prefer to have the retinal exam now:

1	2	3	4	5
(Prefer ophthalmologist's office)		undecided	(Prefer family physician)	

☐ yes ☒ no **Did the camera flashlight bother you?**

☐ yes ☐ no **Did the pupil dilating drops bother you?** ☒ not applicable

Patient Questionnaire

Name [redacted] **Date** [redacted]

BEFORE telediagnosis

Thank you for your willingness to participate in this research project.

You are about to undergo digital photographing and diagnosis over the internet by ophthalmologists who are retinal specialists at the University of Iowa. They will evaluate your photographs for signs of diabetic retinopathy, a complication of diabetes. This new technique is called 'telediagnosis'. Until telediagnosis was available, you could only be screened in an ophthalmologist's office with a dilated retinal exam. Please give your best answer.

What number best expresses where you think the *quality* of the retinal exam is best:

1	2	3	4	5
(Better in ophthalmologist's office)		undecided		(Better through Telediagnosis)

What number best expresses *where* you prefer to have the retinal exam:

1	2	3	4	5
(Prefer ophthalmologist's office)		undecided		(Prefer family physician)

☐ yes ☑ no **Do you expect the camera flashlight to bother you?**

☐ yes ☑ no **Do you expect the pupil dilating drops to bother you?**

☐ yes ☑ no **In the past five years have you been examined by an ophthalmologist?**

☑ yes ☐ no **Do you currently carry health insurance?**

☐ yes ☑ no **Sometimes photographs can only be made if your pupils are made larger with a drop. Were you aware of this?**

AFTER telediagnosis

What number best expresses your *satisfaction* with telediagnosis:

1	2	3	4	5
(Terrible)		undecided		(Very good)

What number best expresses your where you think the *quality* of the retinal exam is best::

1	2	3	4	5
(Better in ophthalmologist's office)		undecided		(Better through Telediagnosis)

What number best expresses *where* you prefer to have the retinal exam now:

1	2	3	4	5
(Prefer ophthalmologist's office)		undecided		(Prefer family physician)

☐ yes ☒ no **Did the camera flashlight bother you?**

☐ yes ☐ no **Did the pupil dilating drops bother you?** ☒ not applicable

Patient Questionnaire

Name [redacted] Date [redacted]

BEFORE telediagnosis

Thank you for your willingness to participate in this research project.

You are about to undergo digital photographing and diagnosis over the internet by ophthalmologists who are retinal specialists at the University of Iowa. They will evaluate your photographs for signs of diabetic retinopathy, a complication of diabetes. This new technique is called 'telediagnosis'. Until telediagnosis was available, you could only be screened in an ophthalmologist's office with a dilated retinal exam. Please give your best answer.

What number best expresses where you think the *quality* of the retinal exam is best:

1 (Better in ophthalmologist's office)	2	(3) undecided	4	5 (Better through Telediagnosis)

What number best expresses *where* you prefer to have the retinal exam:

1 (Prefer ophthalmologist's office)	2	3 undecided	4	(5) (Prefer family physician)

☐ yes ☒ no **Do you expect the camera flashlight to bother you?**

☐ yes ☒ no **Do you expect the pupil dilating drops to bother you?**

☐ yes ☒ no **In the past five years have you been examined by an ophthalmologist?**

☒ yes ☐ no **Do you currently carry health insurance?**

☒ yes ☐ no **Sometimes photographs can only be made if your pupils are made larger with a drop. Were you aware of this?**

AFTER telediagnosis

What number best expresses your *satisfaction* with telediagnosis:

1 (Terrible)	2	3 undecided	4	(5) (Very good)

What number best expresses your where you think the *quality* of the retinal exam is best::

1 (Better in ophthalmologist's office)	2	(3) undecided	4	5 (Better through Telediagnosis)

What number best expresses *where* you prefer to have the retinal exam <u>now</u>:

1 (Prefer ophthalmologist's office)	2	3 undecided	4	(5) (Prefer family physician)

☐ yes ☒ no **Did the camera flashlight bother you?**

☐ yes ☐ no **Did the pupil dilating drops bother you?** ☒ not applicable

Patient Questionnaire

Name [redacted] ____________ Date [redacted]

BEFORE telediagnosis *l*

Thank you for your willingness to participate in this research project.

You are about to undergo digital photographing and diagnosis over the internet by ophthalmologists who are retinal specialists at the University of Iowa. They will evaluate your photographs for signs of diabetic retinopathy, a complication of diabetes. This new technique is called 'telediagnosis'. Until telediagnosis was available, you could only be screened in an ophthalmologist's office with a dilated retinal exam. Please give your best answer.

What number best expresses where you think the *quality* of the retinal exam is best:

1	2	3	4	5 (circled)
(Better in ophthalmologist's office)		undecided		(Better through Telediagnosis)

What number best expresses *where* you prefer to have the retinal exam:

1	2	3	4	5 (circled)
(Prefer ophthalmologist's office)		undecided		(Prefer family physician)

☐ yes ☒ no **Do you expect the camera flashlight to bother you?**

☐ yes ☐ no **Do you expect the pupil dilating drops to bother you?**

☒ yes ☐ no **In the past five years have you been examined by an ophthalmologist?**

☒ yes ☐ no **Do you currently carry health insurance?**

☒ yes ☐ no **Sometimes photographs can only be made if your pupils are made larger with a drop. Were you aware of this?**

AFTER telediagnosis

What number best expresses your *satisfaction* with telediagnosis:

1	2	3	4	5 (circled)
(Terrible)		undecided		(Very good)

What number best expresses your where you think the *quality* of the retinal exam is best::

1	2	3	4	5 (circled)
(Better in ophthalmologist's office)		undecided		(Better through Telediagnosis)

What number best expresses *where* you prefer to have the retinal exam now:

1	2	3	4	5 (circled)
(Prefer ophthalmologist's office)		undecided		(Prefer family physician)

☐ yes ☒ no **Did the camera flashlight bother you?**

☐ yes ☐ no **Did the pupil dilating drops bother you?** ☐ not applicable

[redacted]

[redacted]ient Questionnaire

Name [redacted] ____________ Date [redacted]

BEFORE telediagnosis

Thank you for your willingness to participate in this research project.

You are about to undergo digital photographing and diagnosis over the internet by ophthalmologists who are retinal specialists at the University of Iowa. They will evaluate your photographs for signs of diabetic retinopathy, a complication of diabetes. This new technique is called 'telediagnosis'. Until telediagnosis was available, you could only be screened in an ophthalmologist's office with a dilated retinal exam. Please give your best answer.

What number best expresses where you think the *quality* of the retinal exam is best:

1	2	3	4	5
(Better in ophthalmologist's office)		undecided	(Better through Telediagnosis)	

What number best expresses *where* you prefer to have the retinal exam:

1	2	3	4	5
(Prefer ophthalmologist's office)		undecided	(Prefer family physician)	

☐ yes ☑ no **Do you expect the camera flashlight to bother you?**

☐ yes ☐ no **Do you expect the pupil dilating drops to bother you?**

☑ yes ☐ no **In the past five years have you been examined by an ophthalmologist?**

☑ yes ☐ no **Do you currently carry health insurance?**

☑ yes ☐ no **Sometimes photographs can only be made if your pupils are made larger with a drop. Were you aware of this?**

AFTER telediagnosis

What number best expresses your *satisfaction* with telediagnosis:

1	2	3	4	5
(Terrible)		undecided		(Very good)

What number best expresses your where you think the *quality* of the retinal exam is best::

1	2	3	4	5
(Better in ophthalmologist's office)		undecided	(Better through Telediagnosis)	

What number best expresses *where* you prefer to have the retinal exam <u>now</u>:

1	2	3	4	5
(Prefer ophthalmologist's office)		undecided	(Prefer family physician)	

☐ yes ☑ no **Did the camera flashlight bother you?**

☐ yes ☐ no **Did the pupil dilating drops bother you?** ☐ not applicable

[redacted] Patient Questionnaire

Name [redacted] Date [redacted]

BEFORE telediagnosis

Thank you for your willingness to participate in this research project.

You are about to undergo digital photographing and diagnosis over the internet by ophthalmologists who are retinal specialists at the University of Iowa. They will evaluate your photographs for signs of diabetic retinopathy, a complication of diabetes. This new technique is called 'telediagnosis'. Until telediagnosis was available, you could only be screened in an ophthalmologist's office with a dilated retinal exam. Please give your best answer.

What number best expresses where you think the *quality* of the retinal exam is best:

1	2	3	4	**5**
(Better in ophthalmologist's office)		undecided		(Better through Telediagnosis)

What number best expresses *where* you prefer to have the retinal exam:

1	2	3	4	**5**
(Prefer ophthalmologist's office)		undecided		(Prefer family physician)

☐ yes ☒ no **Do you expect the camera flashlight to bother you?**

☐ yes ☐ no **Do you expect the pupil dilating drops to bother you?**

☐ yes ☒ no **In the past five years have you been examined by an ophthalmologist?**

☒ yes ☐ no **Do you currently carry health insurance?**

☒ yes ☐ no **Sometimes photographs can only be made if your pupils are made larger with a drop. Were you aware of this?**

AFTER telediagnosis

What number best expresses your *satisfaction* with telediagnosis:

1	2	3	4	**5**
(Terrible)		undecided		(Very good)

What number best expresses your where you think the *quality* of the retinal exam is best::

1	2	3	4	**5**
(Better in ophthalmologist's office)		undecided		(Better through Telediagnosis)

What number best expresses *where* you prefer to have the retinal exam <u>now</u>:

1	2	3	4	**5**
(Prefer ophthalmologist's office)		undecided		(Prefer family physician)

☐ yes ☒ no **Did the camera flashlight bother you?**

☐ yes ☐ no **Did the pupil dilating drops bother you?** ☒ not applicable

Patient Questionnaire

Name [redacted] Date [redacted]

BEFORE telediagnosis

Thank you for your willingness to participate in this research project.

You are about to undergo digital photographing and diagnosis over the internet by ophthalmologists who are retinal specialists at the University of Iowa. They will evaluate your photographs for signs of diabetic retinopathy, a complication of diabetes. This new technique is called 'telediagnosis'. Until telediagnosis was available, you could only be screened in an ophthalmologist's office with a dilated retinal exam. Please give your best answer.

What number best expresses where you think the *quality* of the retinal exam is best:

1	2	3	4	5
(Better in ophthalmologist's office)		undecided		(Better through Telediagnosis)

What number best expresses *where* you prefer to have the retinal exam:

1	2	3	4	5
(Prefer ophthalmologist's office)		undecided		(Prefer family physician)

☐ yes ☒ no **Do you expect the camera flashlight to bother you?**

☐ yes ☐ no **Do you expect the pupil dilating drops to bother you?**

☒ yes ☐ no **In the past five years have you been examined by an ophthalmologist?**

☒ yes ☐ no **Do you currently carry health insurance?**

☐ yes ☒ no **Sometimes photographs can only be made if your pupils are made larger with a drop. Were you aware of this?**

AFTER telediagnosis

What number best expresses your *satisfaction* with telediagnosis:

1	2	3	4	5
(Terrible)		undecided		(Very good)

What number best expresses your where you think the *quality* of the retinal exam is best::

1	2	3	4	5
(Better in ophthalmologist's office)		undecided		(Better through Telediagnosis)

What number best expresses *where* you prefer to have the retinal exam <u>now</u>:

1	2	3	4	5
(Prefer ophthalmologist's office)		undecided		(Prefer family physician)

☐ yes ☒ no **Did the camera flashlight bother you?**

☐ yes ☐ no **Did the pupil dilating drops bother you?** ☒ not applicable

[redacted]

[redacted] **Patient Questionnaire**

Name [redacted] Date [redacted]

BEFORE telediagnosis

Thank you for your willingness to participate in this research project.

You are about to undergo digital photographing and diagnosis over the internet by ophthalmologists who are retinal specialists at the University of Iowa. They will evaluate your photographs for signs of diabetic retinopathy, a complication of diabetes. This new technique is called 'telediagnosis'. Until telediagnosis was available, you could only be screened in an ophthalmologist's office with a dilated retinal exam. Please give your best answer.

What number best expresses where you think the *quality* of the retinal exam is best:

1	2	3	4	5
(Better in ophthalmologist's office)		undecided		(Better through Telediagnosis)

What number best expresses *where* you prefer to have the retinal exam:

1	2	3	4	5
(Prefer ophthalmologist's office)		undecided		(Prefer family physician)

☑ yes ☐ no **Do you expect the camera flashlight to bother you?**

☑ yes ☐ no **Do you expect the pupil dilating drops to bother you?**

☑ yes ☐ no **In the past five years have you been examined by an ophthalmologist?**

☑ yes ☐ no **Do you currently carry health insurance?**

☐ yes ☒ no **Sometimes photographs can only be made if your pupils are made larger with a drop. Were you aware of this?**

AFTER telediagnosis

What number best expresses your *satisfaction* with telediagnosis:

1	2	3	4	5
(Terrible)		undecided		(Very good)

What number best expresses your where you think the *quality* of the retinal exam is best::

1	2	3	4	5
(Better in ophthalmologist's office)		undecided		(Better through Telediagnosis)

What number best expresses *where* you prefer to have the retinal exam now:

1	2	3	4	5
(Prefer ophthalmologist's office)		undecided		(Prefer family physician)

☐ yes ☒ no **Did the camera flashlight bother you?**

☐ yes ☐ no **Did the pupil dilating drops bother you?** ☒ not applicable

Patient Questionnaire

Name [redacted] Date [redacted]

BEFORE telediagnosis

Thank you for your willingness to participate in this research project.

You are about to undergo digital photographing and diagnosis over the internet by ophthalmologists who are retinal specialists at the University of Iowa. They will evaluate your photographs for signs of diabetic retinopathy, a complication of diabetes. This new technique is called 'telediagnosis'. Until telediagnosis was available, you could only be screened in an ophthalmologist's office with a dilated retinal exam. Please give your best answer.

What number best expresses where you think the *quality* of the retinal exam is best:

1 (Better in ophthalmologist's office)	2	3 undecided	4	5 (Better through Telediagnosis)

What number best expresses *where* you prefer to have the retinal exam:

1 (Prefer ophthalmologist's office)	2	3 undecided	4	5 (Prefer family physician)

☐ yes ☒ no **Do you expect the camera flashlight to bother you?**

☐ yes ☒ no **Do you expect the pupil dilating drops to bother you?**

☒ yes ☐ no **In the past five years have you been examined by an ophthalmologist?**

☒ yes ☐ no **Do you currently carry health insurance?**

☒ yes ☐ no **Sometimes photographs can only be made if your pupils are made larger with a drop. Were you aware of this?**

AFTER telediagnosis

What number best expresses your *satisfaction* with telediagnosis:

1 (Terrible)	2	3 undecided	4	5 (Very good)

What number best expresses your where you think the *quality* of the retinal exam is best::

1 (Better in ophthalmologist's office)	2	3 undecided	4	5 (Better through Telediagnosis)

What number best expresses *where* you prefer to have the retinal exam now:

1 (Prefer ophthalmologist's office)	2	3 undecided	4	5 (Prefer family physician)

☐ yes ☒ no **Did the camera flashlight bother you?**

☐ yes ☐ no **Did the pupil dilating drops bother you?** ☒ not applicable

Patient Questionnaire

Name [redacted] Date [redacted]

BEFORE telediagnosis

Thank you for your willingness to participate in this research project.

You are about to undergo digital photographing and diagnosis over the internet by ophthalmologists who are retinal specialists at the University of Iowa. They will evaluate your photographs for signs of diabetic retinopathy, a complication of diabetes. This new technique is called 'telediagnosis'. Until telediagnosis was available, you could only be screened in an ophthalmologist's office with a dilated retinal exam. Please give your best answer.

What number best expresses where you think the *quality* of the retinal exam is best:

1	2	3	4	5
(Better in ophthalmologist's office)		undecided		(Better through Telediagnosis)

What number best expresses *where* you prefer to have the retinal exam:

1	2	3	4	5
(Prefer ophthalmologist's office)		undecided		(Prefer family physician)

☐ yes ☑ no **Do you expect the camera flashlight to bother you?**

☐ yes ☑ no **Do you expect the pupil dilating drops to bother you?**

☑ yes ☐ no **In the past five years have you been examined by an ophthalmologist?**

☑ yes ☐ no **Do you currently carry health insurance?**

☑ yes ☐ no **Sometimes photographs can only be made if your pupils are made larger with a drop. Were you aware of this?**

AFTER telediagnosis

What number best expresses your *satisfaction* with telediagnosis:

1	2	3	4	5
(Terrible)		undecided		(Very good)

What number best expresses your where you think the *quality* of the retinal exam is best::

1	2	3	4	5
(Better in ophthalmologist's office)		undecided		(Better through Telediagnosis)

What number best expresses *where* you prefer to have the retinal exam <u>now</u>:

1	2	3	4	5
(Prefer ophthalmologist's office)		undecided		(Prefer family physician)

☐ yes ☑ no **Did the camera flashlight bother you?**

☐ yes ☐ no **Did the pupil dilating drops bother you?** ☑ not applicable

[redacted]

Patient Questionnaire

Name [redacted] Date [redacted]

BEFORE telediagnosis

Thank you for your willingness to participate in this research project.

You are about to undergo digital photographing and diagnosis over the internet by ophthalmologists who are retinal specialists at the University of Iowa. They will evaluate your photographs for signs of diabetic retinopathy, a complication of diabetes. This new technique is called 'telediagnosis'. Until telediagnosis was available, you could only be screened in an ophthalmologist's office with a dilated retinal exam. Please give your best answer.

What number best expresses where you think the *quality* of the retinal exam is best:

1	2	3	4	5
(Better in ophthalmologist's office)		undecided		(Better through Telediagnosis)

What number best expresses *where* you prefer to have the retinal exam:

1	2	3	4	5
(Prefer ophthalmologist's office)		undecided		(Prefer family physician)

☐ yes ☒ no **Do you expect the camera flashlight to bother you?**

☒ yes ☐ no **Do you expect the pupil dilating drops to bother you?**

☐ yes ☒ no **In the past five years have you been examined by an ophthalmologist?**

☒ yes ☐ no **Do you currently carry health insurance?**

☒ yes ☐ no **Sometimes photographs can only be made if your pupils are made larger with a drop. Were you aware of this?**

AFTER telediagnosis

What number best expresses your *satisfaction* with telediagnosis:

1	2	3	4	5
(Terrible)		undecided		(Very good)

What number best expresses your where you think the *quality* of the retinal exam is best::

1	2	3	4	5
(Better in ophthalmologist's office)		undecided		(Better through Telediagnosis)

What number best expresses *where* you prefer to have the retinal exam now:

1	2	3	4	5
(Prefer ophthalmologist's office)		undecided		(Prefer family physician)

☐ yes ☒ no **Did the camera flashlight bother you?**

☐ yes ☐ no **Did the pupil dilating drops bother you?** ☒ not applicable

[redacted]

Patient Questionnaire

Name [redacted] Date [redacted]

BEFORE telediagnosis

Thank you for your willingness to participate in this research project.

You are about to undergo digital photographing and diagnosis over the internet by ophthalmologists who are retinal specialists at the University of Iowa. They will evaluate your photographs for signs of diabetic retinopathy, a complication of diabetes. This new technique is called 'telediagnosis'. Until telediagnosis was available, you could only be screened in an ophthalmologist's office with a dilated retinal exam. Please give your best answer.

What number best expresses where you think the *quality* of the retinal exam is best:

1 (Better in ophthalmologist's office)	2	3 undecided	4	5 (Better through Telediagnosis)

What number best expresses *where* you prefer to have the retinal exam:

1 (Prefer ophthalmologist's office)	2	3 undecided	4	5 (Prefer family physician)

☐ yes ☑ no **Do you expect the camera flashlight to bother you?**

~~☐ yes ☐ no~~ **Do you expect the pupil dilating drops to bother you?**

☐ yes ☑ no **In the past five years have you been examined by an ophthalmologist?**

☑ yes ☐ no **Do you currently carry health insurance?**

~~☐ yes ☐ no~~ **Sometimes photographs can only be made if your pupils are made larger with a drop. Were you aware of this?**

AFTER telediagnosis

What number best expresses your *satisfaction* with telediagnosis:

1 (Terrible)	2	3 undecided	4	(5) (Very good)

What number best expresses your where you think the *quality* of the retinal exam is best::

1 (Better in ophthalmologist's office)	2	(3) undecided	4	5 (Better through Telediagnosis)

What number best expresses *where* you prefer to have the retinal exam now:

1 (Prefer ophthalmologist's office)	2	(3) undecided	4	5 (Prefer family physician)

☐ yes ☑ no **Did the camera flashlight bother you?**

☐ yes ☐ no **Did the pupil dilating drops bother you?** ☑ not applicable

[redacted]

Patient Questionnaire

Name [redacted] Date [redacted]

BEFORE telediagnosis

Thank you for your willingness to participate in this research project.

You are about to undergo digital photographing and diagnosis over the internet by ophthalmologists who are retinal specialists at the University of Iowa. They will evaluate your photographs for signs of diabetic retinopathy, a complication of diabetes. This new technique is called 'telediagnosis'. Until telediagnosis was available, you could only be screened in an ophthalmologist's office with a dilated retinal exam. Please give your best answer.

What number best expresses where you think the *quality* of the retinal exam is best:

1	2	3	4	5
(Better in ophthalmologist's office)		undecided		(Better through Telediagnosis)

What number best expresses *where* you prefer to have the retinal exam:

1	2	3	4	5
(Prefer ophthalmologist's office)		undecided		(Prefer family physician)

☐ yes ☒ no **Do you expect the camera flashlight to bother you?**

☐ yes ☐ no **Do you expect the pupil dilating drops to bother you?**

☒ yes ☐ no **In the past five years have you been examined by an ophthalmologist?**

☒ yes ☐ no **Do you currently carry health insurance?**

☐ yes ☒ no **Sometimes photographs can only be made if your pupils are made larger with a drop. Were you aware of this?**

AFTER telediagnosis

What number best expresses your *satisfaction* with telediagnosis:

1	2	3	4	5
(Terrible)		undecided		(Very good)

What number best expresses your where you think the *quality* of the retinal exam is best::

1	2	3	4	5
(Better in ophthalmologist's office)		undecided		(Better through Telediagnosis)

What number best expresses *where* you prefer to have the retinal exam now:

1	2	3	4	5
(Prefer ophthalmologist's office)		undecided		(Prefer family physician)

☐ yes ☒ no **Did the camera flashlight bother you?**

☐ yes ☐ no **Did the pupil dilating drops bother you?** ☒ not applicable

Patient Questionnaire

Name [redacted] Date [redacted]

BEFORE telediagnosis

Thank you for your willingness to participate in this research project.

You are about to undergo digital photographing and diagnosis over the internet by ophthalmologists who are retinal specialists at the University of Iowa. They will evaluate your photographs for signs of diabetic retinopathy, a complication of diabetes. This new technique is called 'telediagnosis'. Until telediagnosis was available, you could only be screened in an ophthalmologist's office with a dilated retinal exam. Please give your best answer.

What number best expresses where you think the *quality* of the retinal exam is best:

1	2	3	4	5
(Better in ophthalmologist's office)		undecided		(Better through Telediagnosis)

What number best expresses *where* you prefer to have the retinal exam:

1	2	3	4	5
(Prefer ophthalmologist's office)		undecided		(Prefer family physician)

□ yes □ no **Do you expect the camera flashlight to bother you?**

□ yes □ no ~~**Do you expect the pupil dilating drops to bother you?**~~

□ yes □ no **In the past five years have you been examined by an ophthalmologist?**

□ yes □ no **Do you currently carry health insurance?**

□ yes □ no **Sometimes photographs can only be made if your pupils are made larger with a drop. Were you aware of this?**

AFTER telediagnosis

What number best expresses your *satisfaction* with telediagnosis:

1	2	3	4	5
(Terrible)		undecided		(Very good)

What number best expresses your where you think the *quality* of the retinal exam is best::

1	2	3	4	5
(Better in ophthalmologist's office)		undecided		(Better through Telediagnosis)

What number best expresses *where* you prefer to have the retinal exam <u>now</u>:

1	2	3	4	5
(Prefer ophthalmologist's office)		undecided		(Prefer family physician)

□ yes □ no **Did the camera flashlight bother you?**

□ yes □ no **Did the pupil dilating drops bother you?** □ not applicable

Patient Questionnaire

Name [redacted] Date [redacted]

BEFORE telediagnosis

Thank you for your willingness to participate in this research project.

You are about to undergo digital photographing and diagnosis over the internet by ophthalmologists who are retinal specialists at the University of Iowa. They will evaluate your photographs for signs of diabetic retinopathy, a complication of diabetes. This new technique is called 'telediagnosis'. Until telediagnosis was available, you could only be screened in an ophthalmologist's office with a dilated retinal exam. Please give your best answer.

What number best expresses where you think the *quality* of the retinal exam is best:

1	2	3	4	5
(Better in ophthalmologist's office)		undecided		(Better through Telediagnosis)

What number best expresses *where* you prefer to have the retinal exam:

1	2	3	4	5
(Prefer ophthalmologist's office)		undecided		(Prefer family physician)

☐ yes ☒ no **Do you expect the camera flashlight to bother you?**

☐ yes ☒ no **Do you expect the pupil dilating drops to bother you?**

☒ yes ☐ no **In the past five years have you been examined by an ophthalmologist?**

☒ yes ☐ no **Do you currently carry health insurance?**

☒ yes ☐ no **Sometimes photographs can only be made if your pupils are made larger with a drop. Were you aware of this?**

AFTER telediagnosis

What number best expresses your *satisfaction* with telediagnosis:

1	2	3	4	5
(Terrible)		undecided		(Very good)

What number best expresses your where you think the *quality* of the retinal exam is best::

1	2	3	4	5
(Better in ophthalmologist's office)		undecided		(Better through Telediagnosis)

What number best expresses *where* you prefer to have the retinal exam <u>now</u>:

1	2	3	4	5
(Prefer ophthalmologist's office)		undecided		(Prefer family physician)

☐ yes ☒ no **Did the camera flashlight bother you?**

☐ yes ☒ no **Did the pupil dilating drops bother you?** ☐ not applicable

Patient Questionnaire

Name [redacted] Date [redacted]

BEFORE telediagnosis

Thank you for your willingness to participate in this research project.

You are about to undergo digital photographing and diagnosis over the internet by ophthalmologists who are retinal specialists at the University of Iowa. They will evaluate your photographs for signs of diabetic retinopathy, a complication of diabetes. This new technique is called 'telediagnosis'. Until telediagnosis was available, you could only be screened in an ophthalmologist's office with a dilated retinal exam. Please give your best answer.

What number best expresses where you think the *quality* of the retinal exam is best:

1	2	3	4	5
(Better in ophthalmologist's office)		undecided		(Better through Telediagnosis)

What number best expresses *where* you prefer to have the retinal exam:

1	2	3	4	5
(Prefer ophthalmologist's office)		undecided		(Prefer family physician)

☐ yes ☒ no **Do you expect the camera flashlight to bother you?**

☐ yes ☒ no **Do you expect the pupil dilating drops to bother you?**

☐ yes ☒ no **In the past five years have you been examined by an ophthalmologist?**

☒ yes ☐ no **Do you currently carry health insurance?**

☒ yes ☐ no **Sometimes photographs can only be made if your pupils are made larger with a drop. Were you aware of this?**

AFTER telediagnosis

What number best expresses your *satisfaction* with telediagnosis:

1	2	3	4	5
(Terrible)		undecided		(Very good)

What number best expresses your where you think the *quality* of the retinal exam is best::

1	2	3	4	5
(Better in ophthalmologist's office)		undecided		(Better through Telediagnosis)

What number best expresses *where* you prefer to have the retinal exam now:

1	2	3	4	5
(Prefer ophthalmologist's office)		undecided		(Prefer family physician)

☐ yes ☒ no **Did the camera flashlight bother you?**

☐ yes ☐ no **Did the pupil dilating drops bother you?** ☒ not applicable

Patient Questionnaire

Name [redacted] Date [redacted]

BEFORE telediagnosis

Thank you for your willingness to participate in this research project.

You are about to undergo digital photographing and diagnosis over the internet by ophthalmologists who are retinal specialists at the University of Iowa. They will evaluate your photographs for signs of diabetic retinopathy, a complication of diabetes. This new technique is called 'telediagnosis'. Until telediagnosis was available, you could only be screened in an ophthalmologist's office with a dilated retinal exam. Please give your best answer.

What number best expresses where you think the *quality* of the retinal exam is best:

1	2	3	4	5
(Better in ophthalmologist's office)		undecided		(Better through Telediagnosis)

What number best expresses *where* you prefer to have the retinal exam:

1	2	3	4	5
(Prefer ophthalmologist's office)		undecided		(Prefer family physician)

☐ yes ☒ no **Do you expect the camera flashlight to bother you?**

☐ yes ☒ no **Do you expect the pupil dilating drops to bother you?**

☒ yes ☐ no **In the past five years have you been examined by an ophthalmologist?**

☒ yes ☐ no **Do you currently carry health insurance?**

☐ yes ☐ no **Sometimes photographs can only be made if your pupils are made larger with a drop. Were you aware of this?**

AFTER telediagnosis

What number best expresses your *satisfaction* with telediagnosis:

1	2	3	4	5
(Terrible)		undecided		(Very good)

What number best expresses your where you think the *quality* of the retinal exam is best::

1	2	3	4	5
(Better in ophthalmologist's office)		undecided		(Better through Telediagnosis)

What number best expresses *where* you prefer to have the retinal exam <u>now</u>:

1	2	3	4	5
(Prefer ophthalmologist's office)		undecided		(Prefer family physician)

☐ yes ☒ no **Did the camera flashlight bother you?**

☐ yes ☐ no **Did the pupil dilating drops bother you?** ☒ not applicable

Patient Questionnaire

Name [redacted] Date [redacted]

BEFORE telediagnosis

Thank you for your willingness to participate in this research project.

You are about to undergo digital photographing and diagnosis over the internet by ophthalmologists who are retinal specialists at the University of Iowa. They will evaluate your photographs for signs of diabetic retinopathy, a complication of diabetes. This new technique is called 'telediagnosis'. Until telediagnosis was available, you could only be screened in an ophthalmologist's office with a dilated retinal exam. Please give your best answer.

What number best expresses where you think the *quality* of the retinal exam is best:

1	2	3	4	5
(Better in ophthalmologist's office)		undecided		(Better through Telediagnosis)

What number best expresses *where* you prefer to have the retinal exam:

1	2	3	4	5
(Prefer ophthalmologist's office)		undecided		(Prefer family physician)

☐ yes ☑ no **Do you expect the camera flashlight to bother you?**

☐ yes ☑ no **Do you expect the pupil dilating drops to bother you?**

☐ yes ☑ no **In the past five years have you been examined by an ophthalmologist?**

☑ yes ☐ no **Do you currently carry health insurance?**

☑ yes ☐ no **Sometimes photographs can only be made if your pupils are made larger with a drop. Were you aware of this?**

AFTER telediagnosis

What number best expresses your *satisfaction* with telediagnosis:

1	2	3	4	5
(Terrible)		undecided		(Very good)

What number best expresses your where you think the *quality* of the retinal exam is best::

1	2	3	4	5
(Better in ophthalmologist's office)		undecided		(Better through Telediagnosis)

What number best expresses *where* you prefer to have the retinal exam <u>now</u>:

1	2	3	4	5
(Prefer ophthalmologist's office)		undecided		(Prefer family physician)

☐ yes ☑ no **Did the camera flashlight bother you?**

☐ yes ☑ no **Did the pupil dilating drops bother you?** ☐ not applicable

[redacted]

Patient Questionnaire

Name [redacted] Date [redacted]

BEFORE telediagnosis

Thank you for your willingness to participate in this research project.

You are about to undergo digital photographing and diagnosis over the internet by ophthalmologists who are retinal specialists at the University of Iowa. They will evaluate your photographs for signs of diabetic retinopathy, a complication of diabetes. This new technique is called 'telediagnosis'. Until telediagnosis was available, you could only be screened in an ophthalmologist's office with a dilated retinal exam. Please give your best answer.

What number best expresses where you think the *quality* of the retinal exam is best:

1	2	3	4	5 (circled)
(Better in ophthalmologist's office)		undecided		(Better through Telediagnosis)

What number best expresses *where* you prefer to have the retinal exam:

1	2	3	4	5 (circled)
(Prefer ophthalmologist's office)		undecided		(Prefer family physician)

☐ yes ☒ no **Do you expect the camera flashlight to bother you?**

☐ yes ☒ no **Do you expect the pupil dilating drops to bother you?**

☒ yes ☐ no **In the past five years have you been examined by an ophthalmologist?**

☒ yes ☐ no **Do you currently carry health insurance?**

☒ yes ☐ no **Sometimes photographs can only be made if your pupils are made larger with a drop. Were you aware of this?**

AFTER telediagnosis

What number best expresses your *satisfaction* with telediagnosis:

1	2	3	4	5 (circled)
(Terrible)		undecided		(Very good)

What number best expresses your where you think the *quality* of the retinal exam is best::

1	2	3	4	5 (circled)
(Better in ophthalmologist's office)		undecided		(Better through Telediagnosis)

What number best expresses *where* you prefer to have the retinal exam <u>now</u>:

1	2	3	4	5 (circled)
(Prefer ophthalmologist's office)		undecided		(Prefer family physician)

☐ yes ☒ no **Did the camera flashlight bother you?**

☐ yes ☐ no **Did the pupil dilating drops bother you?** ☒ not applicable

Patient Questionnaire

Name [redacted] Date [redacted]

BEFORE telediagnosis

Thank you for your willingness to participate in this research project.

You are about to undergo digital photographing and diagnosis over the internet by ophthalmologists who are retinal specialists at the University of Iowa. They will evaluate your photographs for signs of diabetic retinopathy, a complication of diabetes. This new technique is called 'telediagnosis'. Until telediagnosis was available, you could only be screened in an ophthalmologist's office with a dilated retinal exam. Please give your best answer.

What number best expresses where you think the *quality* of the retinal exam is best:

1	2	3 (circled)	4	5
(Better in ophthalmologist's office)		undecided		(Better through Telediagnosis)

What number best expresses *where* you prefer to have the retinal exam:

1	2	3 (circled)	4	5
(Prefer ophthalmologist's office)		undecided		(Prefer family physician)

☐ yes ☒ no **Do you expect the camera flashlight to bother you?**

☐ yes ☒ no **Do you expect the pupil dilating drops to bother you?**

☐ yes ☒ no **In the past five years have you been examined by an ophthalmologist?**

☒ yes ☐ no **Do you currently carry health insurance?**

☐ yes ☐ no **Sometimes photographs can only be made if your pupils are made larger with a drop. Were you aware of this?**

AFTER telediagnosis

What number best expresses your *satisfaction* with telediagnosis:

1	2	3 (circled)	4	5
(Terrible)		undecided		(Very good)

What number best expresses your where you think the *quality* of the retinal exam is best::

1	2	3 (circled)	4	5
(Better in ophthalmologist's office)		undecided		(Better through Telediagnosis)

What number best expresses *where* you prefer to have the retinal exam now:

1	2	3 (circled)	4	5
(Prefer ophthalmologist's office)		undecided		(Prefer family physician)

☐ yes ☐ no **Did the camera flashlight bother you?**

☐ yes ☐ no **Did the pupil dilating drops bother you?** ☐ not applicable

[redacted]t Questionnaire

Name [redacted] Date [redacted]

BEFORE telediagnosis

Thank you for your willingness to participate in this research project.

You are about to undergo digital photographing and diagnosis over the internet by ophthalmologists who are retinal specialists at the University of Iowa. They will evaluate your photographs for signs of diabetic retinopathy, a complication of diabetes. This new technique is called 'telediagnosis'. Until telediagnosis was available, you could only be screened in an ophthalmologist's office with a dilated retinal exam. Please give your best answer.

What number best expresses where you think the *quality* of the retinal exam is best:

1	2	3	4	5
(Better in ophthalmologist's office)		undecided	(Better through Telediagnosis)	

What number best expresses *where* you prefer to have the retinal exam:

1	2	3	4	5
(Prefer ophthalmologist's office)		undecided		(Prefer family physician)

☐ yes ☑ no **Do you expect the camera flashlight to bother you?**

☐ yes ☐ no **Do you expect the pupil dilating drops to bother you?**

☑ yes ☐ no **In the past five years have you been examined by an ophthalmologist?**

☑ yes ☐ no **Do you currently carry health insurance?**

☑ yes ☐ no **Sometimes photographs can only be made if your pupils are made larger with a drop. Were you aware of this?**

AFTER telediagnosis

What number best expresses your *satisfaction* with telediagnosis:

1	2	3	4	5
(Terrible)		undecided		(Very good)

What number best expresses your where you think the *quality* of the retinal exam is best::

1	2	3	4	5
(Better in ophthalmologist's office)		undecided	(Better through Telediagnosis)	

What number best expresses *where* you prefer to have the retinal exam now:

1	2	3	4	5
(Prefer ophthalmologist's office)		undecided		(Prefer family physician)

☐ yes ☑ no **Did the camera flashlight bother you?**

☐ yes ☐ no **Did the pupil dilating drops bother you?** ☐ not applicable

Patient Questionnaire

Name [redacted] Date [redacted]

BEFORE telediagnosis

Thank you for your willingness to participate in this research project.

You are about to undergo digital photographing and diagnosis over the internet by ophthalmologists who are retinal specialists at the University of Iowa. They will evaluate your photographs for signs of diabetic retinopathy, a complication of diabetes. This new technique is called 'telediagnosis'. Until telediagnosis was available, you could only be screened in an ophthalmologist's office with a dilated retinal exam. Please give your best answer.

What number best expresses where you think the *quality* of the retinal exam is best:

1	2	3	4	5
(Better in ophthalmologist's office)		undecided		(Better through Telediagnosis)

What number best expresses *where* you prefer to have the retinal exam:

1	2	3	4	5
(Prefer ophthalmologist's office)		undecided		(Prefer family physician)

☐ yes ☑ no **Do you expect the camera flashlight to bother you?**

☐ yes ☑ no **Do you expect the pupil dilating drops to bother you?**

☑ yes ☐ no **In the past five years have you been examined by an ophthalmologist?**

☑ yes ☐ no **Do you currently carry health insurance?**

☑ yes ☐ no **Sometimes photographs can only be made if your pupils are made larger with a drop. Were you aware of this?**

AFTER telediagnosis

What number best expresses your *satisfaction* with telediagnosis:

1	2	3	4	5
(Terrible)		undecided		(Very good)

What number best expresses your where you think the *quality* of the retinal exam is best::

1	2	3	4	5
(Better in ophthalmologist's office)		undecided		(Better through Telediagnosis)

What number best expresses *where* you prefer to have the retinal exam <u>now</u>:

1	2	3	4	5
(Prefer ophthalmologist's office)		undecided		(Prefer family physician)

☐ yes ☑ no **Did the camera flashlight bother you?**

☐ yes ☐ no **Did the pupil dilating drops bother you?** ☑ not applicable

Patient Questionnaire

Name [redacted] Date [redacted]

BEFORE telediagnosis

Thank you for your willingness to participate in this research project.

You are about to undergo digital photographing and diagnosis over the internet by ophthalmologists who are retinal specialists at the University of Iowa. They will evaluate your photographs for signs of diabetic retinopathy, a complication of diabetes. This new technique is called 'telediagnosis'. Until telediagnosis was available, you could only be screened in an ophthalmologist's office with a dilated retinal exam. Please give your best answer.

What number best expresses where you think the *quality* of the retinal exam is best:

1	2	3	4	5
(Better in ophthalmologist's office)		undecided		(Better through Telediagnosis)

What number best expresses *where* you prefer to have the retinal exam:

1	2	3	4	5
(Prefer ophthalmologist's office)		undecided		(Prefer family physician)

☐ yes ☑ no Do you expect the camera flashlight to bother you?

☐ yes ☑ no Do you expect the pupil dilating drops to bother you?

☑ yes ☐ no In the past five years have you been examined by an ophthalmologist?

☑ yes ☐ no Do you currently carry health insurance?

☑ yes ☐ no Sometimes photographs can only be made if your pupils are made larger with a drop. Were you aware of this?

AFTER telediagnosis

What number best expresses your *satisfaction* with telediagnosis:

1	2	3	4	5
(Terrible)		undecided		(Very good)

What number best expresses your where you think the *quality* of the retinal exam is best::

1	2	3	4	5
(Better in ophthalmologist's office)		undecided		(Better through Telediagnosis)

What number best expresses *where* you prefer to have the retinal exam now:

1	2	3	4	5
(Prefer ophthalmologist's office)		undecided		(Prefer family physician)

☐ yes ☑ no Did the camera flashlight bother you?

☐ yes ☐ no Did the pupil dilating drops bother you? ☑ not applicable

Patient Questionnaire

Name [redacted] Date [redacted]

BEFORE telediagnosis

Thank you for your willingness to participate in this research project.

You are about to undergo digital photographing and diagnosis over the internet by ophthalmologists who are retinal specialists at the University of Iowa. They will evaluate your photographs for signs of diabetic retinopathy, a complication of diabetes. This new technique is called 'telediagnosis'. Until telediagnosis was available, you could only be screened in an ophthalmologist's office with a dilated retinal exam. Please give your best answer.

What number best expresses where you think the *quality* of the retinal exam is best:

1	2	3	4	5
(Better in ophthalmologist's office)		undecided		(Better through Telediagnosis)

What number best expresses *where* you prefer to have the retinal exam:

1	2	3	4	5
(Prefer ophthalmologist's office)		undecided		(Prefer family physician)

☐ yes ☒ no **Do you expect the camera flashlight to bother you?**

☐ yes ☒ no **Do you expect the pupil dilating drops to bother you?**

☒ yes ☐ no **In the past five years have you been examined by an ophthalmologist?**

☒ yes ☐ no **Do you currently carry health insurance?**

☒ yes ☐ no **Sometimes photographs can only be made if your pupils are made larger with a drop. Were you aware of this?**

AFTER telediagnosis

What number best expresses your *satisfaction* with telediagnosis:

1	2	3	4	5
(Terrible)		undecided		(Very good)

What number best expresses your where you think the *quality* of the retinal exam is best::

1	2	3	4	5
(Better in ophthalmologist's office)		undecided		(Better through Telediagnosis)

What number best expresses *where* you prefer to have the retinal exam now:

1	2	3	4	5
(Prefer ophthalmologist's office)		undecided		(Prefer family physician)

☐ yes ☒ no **Did the camera flashlight bother you?**

☐ yes ☐ no **Did the pupil dilating drops bother you?** ☒ not applicable

Patient Questionnaire

Name [redacted] **Date** [redacted]

BEFORE telediagnosis

Thank you for your willingness to participate in this research project.

You are about to undergo digital photographing and diagnosis over the internet by ophthalmologists who are retinal specialists at the University of Iowa. They will evaluate your photographs for signs of diabetic retinopathy, a complication of diabetes. This new technique is called 'telediagnosis'. Until telediagnosis was available, you could only be screened in an ophthalmologist's office with a dilated retinal exam. Please give your best answer.

What number best expresses where you think the *quality* of the retinal exam is best:

1	2	3	4	5
(Better in ophthalmologist's office)		undecided		(Better through Telediagnosis)

What number best expresses *where* you prefer to have the retinal exam:

1	2	3	4	5
(Prefer ophthalmologist's office)		undecided		(Prefer family physician)

☐ yes ☒ no **Do you expect the camera flashlight to bother you?**

☒ yes ☐ no **Do you expect the pupil dilating drops to bother you?**

☐ yes ☒ no **In the past five years have you been examined by an ophthalmologist?**

☒ yes ☐ no **Do you currently carry health insurance?**

☒ yes ☐ no **Sometimes photographs can only be made if your pupils are made larger with a drop. Were you aware of this?**

AFTER telediagnosis

What number best expresses your *satisfaction* with telediagnosis:

1	2	3	4	5
(Terrible)		undecided		(Very good)

What number best expresses your where you think the *quality* of the retinal exam is best::

1	2	3	4	5
(Better in ophthalmologist's office)		undecided		(Better through Telediagnosis)

What number best expresses *where* you prefer to have the retinal exam <u>now</u>:

1	2	3	4	5
(Prefer ophthalmologist's office)		undecided		(Prefer family physician)

☒ yes ☐ no **Did the camera flashlight bother you?**

☐ yes ☐ no **Did the pupil dilating drops bother you?** ☒ not applicable

Patient Questionnaire

Name [redacted] Date [redacted]

BEFORE telediagnosis

Thank you for your willingness to participate in this research project.

You are about to undergo digital photographing and diagnosis over the internet by ophthalmologists who are retinal specialists at the University of Iowa. They will evaluate your photographs for signs of diabetic retinopathy, a complication of diabetes. This new technique is called 'telediagnosis'. Until telediagnosis was available, you could only be screened in an ophthalmologist's office with a dilated retinal exam. Please give your best answer.

What number best expresses where you think the *quality* of the retinal exam is best:

1	2	3	4	(5)
(Better in ophthalmologist's office)		undecided		(Better through Telediagnosis)

What number best expresses *where* you prefer to have the retinal exam:

1	2	3	4	(5)
(Prefer ophthalmologist's office)		undecided		(Prefer family physician)

☐ yes ☒ no **Do you expect the camera flashlight to bother you?**

☐ yes ☒ no **Do you expect the pupil dilating drops to bother you?**

☐ yes ☒ no **In the past five years have you been examined by an ophthalmologist?**

☒ yes ☐ no **Do you currently carry health insurance?**

☒ yes ☐ no **Sometimes photographs can only be made if your pupils are made larger with a drop. Were you aware of this?**

AFTER telediagnosis

What number best expresses your *satisfaction* with telediagnosis:

1	2	3	4	(5)
(Terrible)		undecided		(Very good)

What number best expresses your where you think the *quality* of the retinal exam is best::

1	2	3	4	(5)
(Better in ophthalmologist's office)		undecided		(Better through Telediagnosis)

What number best expresses *where* you prefer to have the retinal exam <u>now</u>:

1	2	3	4	(5)
(Prefer ophthalmologist's office)		undecided		(Prefer family physician)

☐ yes ☒ no **Did the camera flashlight bother you?**

☐ yes ☐ no **Did the pupil dilating drops bother you?** ☒ not applicable

Patient Questionnaire

Name [redacted] Date [redacted]

BEFORE telediagnosis

Thank you for your willingness to participate in this research project.

You are about to undergo digital photographing and diagnosis over the internet by ophthalmologists who are retinal specialists at the University of Iowa. They will evaluate your photographs for signs of diabetic retinopathy, a complication of diabetes. This new technique is called 'telediagnosis'. Until telediagnosis was available, you could only be screened in an ophthalmologist's office with a dilated retinal exam. Please give your best answer.

What number best expresses where you think the *quality* of the retinal exam is best:

1	2	3	4	5
(Better in ophthalmologist's office)		undecided	(Better through Telediagnosis)	

What number best expresses *where* you prefer to have the retinal exam:

1	2	3	4	5
(Prefer ophthalmologist's office)		undecided		(Prefer family physician)

☐ yes ☑ no **Do you expect the camera flashlight to bother you?**

☐ yes ☑ no **Do you expect the pupil dilating drops to bother you?**

☑ yes ☐ no **In the past five years have you been examined by an ophthalmologist?**

☑ yes ☐ no **Do you currently carry health insurance?**

☑ yes ☐ no **Sometimes photographs can only be made if your pupils are made larger with a drop. Were you aware of this?**

AFTER telediagnosis

What number best expresses your *satisfaction* with telediagnosis:

1	2	3	4	5
(Terrible)		undecided		(Very good)

What number best expresses your where you think the *quality* of the retinal exam is best::

1	2	3	4	5
(Better in ophthalmologist's office)		undecided	(Better through Telediagnosis)	

What number best expresses *where* you prefer to have the retinal exam now:

1	2	3	4	5
(Prefer ophthalmologist's office)		undecided		(Prefer family physician)

☐ yes ☑ no **Did the camera flashlight bother you?**

☐ yes ☑ no **Did the pupil dilating drops bother you?** ☐ not applicable

Patient Questionnaire

Name [redacted] Date [redacted]

BEFORE telediagnosis

Thank you for your willingness to participate in this research project.

You are about to undergo digital photographing and diagnosis over the internet by ophthalmologists who are retinal specialists at the University of Iowa. They will evaluate your photographs for signs of diabetic retinopathy, a complication of diabetes. This new technique is called 'telediagnosis'. Until telediagnosis was available, you could only be screened in an ophthalmologist's office with a dilated retinal exam. Please give your best answer.

What number best expresses where you think the *quality* of the retinal exam is best:

1	2	3	4	5
(Better in ophthalmologist's office)		undecided		(Better through Telediagnosis)

What number best expresses *where* you prefer to have the retinal exam:

1	2	3	4	5
(Prefer ophthalmologist's office)		undecided		(Prefer family physician)

☐ yes ☒ no **Do you expect the camera flashlight to bother you?**

☐ yes ☒ no **Do you expect the pupil dilating drops to bother you?**

☒ yes ☐ no **In the past five years have you been examined by an ophthalmologist?**

☒ yes ☐ no **Do you currently carry health insurance?**

☒ yes ☐ no **Sometimes photographs can only be made if your pupils are made larger with a drop. Were you aware of this?**

AFTER telediagnosis

What number best expresses your *satisfaction* with telediagnosis:

1	2	3	4	5
(Terrible)		undecided		(Very good)

What number best expresses your where you think the *quality* of the retinal exam is best::

1	2	3	4	5
(Better in ophthalmologist's office)		undecided		(Better through Telediagnosis)

What number best expresses *where* you prefer to have the retinal exam now:

1	2	3	4	5
(Prefer ophthalmologist's office)		undecided		(Prefer family physician)

☐ yes ☒ no **Did the camera flashlight bother you?**

☐ yes ☐ no **Did the pupil dilating drops bother you?** ☒ not applicable

[illegible]estionnaire

Name [redacted] [redacted]

BEFORE telediagnosis

Thank you for your willingness to participate in this research project.

You are about to undergo digital photographing and diagnosis over the internet by ophthalmologists who are retinal specialists at the University of Iowa. They will evaluate your photographs for signs of diabetic retinopathy, a complication of diabetes. This new technique is called 'telediagnosis'. Until telediagnosis was available, you could only be screened in an ophthalmologist's office with a dilated retinal exam. Please give your best answer.

What number best expresses where you think the *quality* of the retinal exam is best:

1	2	3	4	5
(Better in ophthalmologist's office)		undecided		(Better through Telediagnosis)

What number best expresses *where* you prefer to have the retinal exam:

1	2	3	4	5
(Prefer ophthalmologist's office)		undecided		(Prefer family physician)

☐ yes ☐ no **Do you expect the camera flashlight to bother you?**

☐ yes ☐ no **Do you expect the pupil dilating drops to bother you?**

☐ yes ☐ no **In the past five years have you been examined by an ophthalmologist?**

☐ yes ☐ no **Do you currently carry health insurance?**

☐ yes ☐ no **Sometimes photographs can only be made if your pupils are made larger with a drop. Were you aware of this?**

AFTER telediagnosis

What number best expresses your *satisfaction* with telediagnosis:

1	2	3	4	5
(Terrible)		undecided		(Very good)

What number best expresses your where you think the *quality* of the retinal exam is best::

1	2	3	4	5
(Better in ophthalmologist's office)		undecided		(Better through Telediagnosis)

What number best expresses *where* you prefer to have the retinal exam now:

1	2	3	4	5
(Prefer ophthalmologist's office)		undecided		(Prefer family physician)

☐ yes ☐ no **Did the camera flashlight bother you?**

☐ yes ☐ no **Did the pupil dilating drops bother you?** ☐ not applicable

[illegible]onnaire

Name [redacted] Da[redacted]

BEFORE telediagnosis

Thank you for your willingness to participate in this research project.

You are about to undergo digital photographing and diagnosis over the internet by ophthalmologists who are retinal specialists at the University of Iowa. They will evaluate your photographs for signs of diabetic retinopathy, a complication of diabetes. This new technique is called 'telediagnosis'. Until telediagnosis was available, you could only be screened in an ophthalmologist's office with a dilated retinal exam. Please give your best answer.

What number best expresses where you think the *quality* of the retinal exam is best: Hasn't had before

1	2	3	4	5
(Better in ophthalmologist's office)		undecided		(Better through Telediagnosis)

What number best expresses *where* you prefer to have the retinal exam:

1	2	3	4	5
(Prefer ophthalmologist's office)		undecided		(Prefer family physician)

☐ yes ☒ no **Do you expect the camera flashlight to bother you?**

☐ yes ☒ no **Do you expect the pupil dilating drops to bother you?**

☒ yes ☐ no **In the past five years have you been examined by an ophthalmologist?**

☒ yes ☐ no **Do you currently carry health insurance?**

☒ yes ☐ no **Sometimes photographs can only be made if your pupils are made larger with a drop. Were you aware of this?**

AFTER telediagnosis

What number best expresses your *satisfaction* with telediagnosis:

1	2	3	(4)	5
(Terrible)		undecided		(Very good)

What number best expresses your where you think the *quality* of the retinal exam is best::

1	2	3	(4)	5
(Better in ophthalmologist's office)		undecided		(Better through Telediagnosis)

What number best expresses *where* you prefer to have the retinal exam now:

1	2	3	(4)	5
(Prefer ophthalmologist's office)		undecided		(Prefer family physician)

☐ yes ☒ no **Did the camera flashlight bother you?**

☐ yes ☐ no **Did the pupil dilating drops bother you?** ☒ not applicable

Patient Questionnaire

Name [redacted] Date [redacted]

BEFORE telediagnosis

Thank you for your willingness to participate in this research project.

You are about to undergo digital photographing and diagnosis over the internet by ophthalmologists who are retinal specialists at the University of Iowa. They will evaluate your photographs for signs of diabetic retinopathy, a complication of diabetes. This new technique is called 'telediagnosis'. Until telediagnosis was available, you could only be screened in an ophthalmologist's office with a dilated retinal exam. Please give your best answer.

What number best expresses where you think the *quality* of the retinal exam is best:

1 (Better in ophthalmologist's office)	2	3 undecided	4	5 (Better through Telediagnosis)

What number best expresses *where* you prefer to have the retinal exam:

1 (Prefer ophthalmologist's office)	2	3 undecided	4	5 (Prefer family physician)

☐ yes ☒ no **Do you expect the camera flashlight to bother you?**

☐ yes ☒ no **Do you expect the pupil dilating drops to bother you?**

☒ yes ☐ no **In the past five years have you been examined by an ophthalmologist?**

☒ yes ☐ no **Do you currently carry health insurance?**

☒ yes ☐ no **Sometimes photographs can only be made if your pupils are made larger with a drop. Were you aware of this?**

AFTER telediagnosis

What number best expresses your *satisfaction* with telediagnosis:

1 (Terrible)	2	3 undecided	4	5 (Very good)

What number best expresses your where you think the *quality* of the retinal exam is best::

1 (Better in ophthalmologist's office)	2	3 undecided	4	5 (Better through Telediagnosis)

What number best expresses *where* you prefer to have the retinal exam <u>now</u>:

1 (Prefer ophthalmologist's office)	2	3 undecided	4	5 (Prefer family physician)

☐ yes ☒ no **Did the camera flashlight bother you?**

☐ yes ☐ no **Did the pupil dilating drops bother you?** ☒ not applicable

[redacted]stionnaire

Name [redacted] Date [redacted]

BEFORE telediagnosis

Thank you for your willingness to participate in this research project.

You are about to undergo digital photographing and diagnosis over the internet by ophthalmologists who are retinal specialists at the University of Iowa. They will evaluate your photographs for signs of diabetic retinopathy, a complication of diabetes. This new technique is called 'telediagnosis'. Until telediagnosis was available, you could only be screened in an ophthalmologist's office with a dilated retinal exam. Please give your best answer.

What number best expresses where you think the *quality* of the retinal exam is best:

1 (Better in ophthalmologist's office)	2	3 undecided	4	5 (Better through Telediagnosis)

What number best expresses *where* you prefer to have the retinal exam:

1 (Prefer ophthalmologist's office)	2	3 undecided	4	5 (Prefer family physician)

☐ yes ☒ no **Do you expect the camera flashlight to bother you?**

☐ yes ☒ no **Do you expect the pupil dilating drops to bother you?**

☐ yes ☒ no **In the past five years have you been examined by an ophthalmologist?**

☒ yes ☐ no **Do you currently carry health insurance?**

☒ yes ☐ no **Sometimes photographs can only be made if your pupils are made larger with a drop. Were you aware of this?**

AFTER telediagnosis

What number best expresses your *satisfaction* with telediagnosis:

1 (Terrible)	2	3 undecided	4	5 (Very good)

What number best expresses your where you think the *quality* of the retinal exam is best::

1 (Better in ophthalmologist's office)	2	3 undecided	4	5 (Better through Telediagnosis)

What number best expresses *where* you prefer to have the retinal exam <u>now</u>:

1 (Prefer ophthalmologist's office)	2	3 undecided	4	5 (Prefer family physician)

☐ yes ☒ no **Did the camera flashlight bother you?**

☐ yes ☐ no **Did the pupil dilating drops bother you?** ☒ not applicable

Patient Questionnaire

Name [redacted] Date [redacted]

BEFORE telediagnosis

Thank you for your willingness to participate in this research project.

You are about to undergo digital photographing and diagnosis over the internet by ophthalmologists who are retinal specialists at the University of Iowa. They will evaluate your photographs for signs of diabetic retinopathy, a complication of diabetes. This new technique is called 'telediagnosis'. Until telediagnosis was available, you could only be screened in an ophthalmologist's office with a dilated retinal exam. Please give your best answer.

What number best expresses where you think the *quality* of the retinal exam is best:

1	2	3	4	5
(Better in ophthalmologist's office)		undecided		(Better through Telediagnosis)

What number best expresses *where* you prefer to have the retinal exam:

1	2	3	4	5
(Prefer ophthalmologist's office)		undecided		(Prefer family physician)

☐ yes ☒ no **Do you expect the camera flashlight to bother you?**

☐ yes ☒ no **Do you expect the pupil dilating drops to bother you?**

☒ yes ☐ no **In the past five years have you been examined by an ophthalmologist?**

☒ yes ☐ no **Do you currently carry health insurance?**

☒ yes ☐ no **Sometimes photographs can only be made if your pupils are made larger with a drop. Were you aware of this?**

AFTER telediagnosis

What number best expresses your *satisfaction* with telediagnosis:

1	2	3	4	5
(Terrible)		undecided		(Very good)

What number best expresses your where you think the *quality* of the retinal exam is best::

1	2	3	4	5
(Better in ophthalmologist's office)		undecided		(Better through Telediagnosis)

What number best expresses *where* you prefer to have the retinal exam now:

1	2	3	4	5
(Prefer ophthalmologist's office)		undecided		(Prefer family physician)

☐ yes ☒ no **Did the camera flashlight bother you?**

☐ yes ☐ no **Did the pupil dilating drops bother you?** ☒ not applicable

[redacted]

Patient questionnaire

BEFORE telediagnosis

Name: [redacted] **Date:** [redacted]

Thank you for your willingness to participate in this research project.

You are about to undergo digital photographing and diagnosis over the internet by ophthalmologists who are retinal specialists at the University of Iowa. They will evaluate your photographs for signs of diabetic retinopathy, a complication of diabetes. This new technique is called 'telediagnosis'. Until telediagnosis was available, you could only be screened in an ophthalmologist's office with a dilated retinal exam.

Please give your best answer to the following questions

Circle where you expect the quality of the retinal exam to be better:

(1) 2 3 4 5

Ophthalmologist's office ... Telediagnosis

Circle which retinal exam you prefer:

(1) 2 3 4 5

Ophthalmologist's office ... Telediagnosis

- ☐ yes ☒ no Do you expect the camera flashlight to bother you
- ☐ yes ☒ no Do you expect the pupil dilating drops to bother you
- ☐ yes ☒ no In the past five years have you been examined by an ophthalmologist
- ☒ yes ☐ no Do you currently carry health insurance
- ☒ yes ☐ no In some cases the photographs can only be made if your pupils are dilated with a drop. Were you aware of this?

Patient questionnaire

AFTER telediagnosis results have been discussed

Name:____________________ **Date:**__________

Circle your satisfaction with digital photography of your retina

1 2 3 4 (5)

Terrible ... Very good

Circle where you think the retinal exam is better:

1 2 3 4 (5)

Ophthalmologist's office ... Telediagnosis

Circle which retinal exam you prefer now:

1 2 3 4 (5)

Ophthalmologist's office ... Telediagnosis

- ☒ yes ☐ no Did the camera flashlight bother you
- ☐ yes ☒ no Did the pupil dilating drops bother you

After form is completed, please enter into Excel spreadsheet or send to:

Dr. Michael D. Abramoff, MD, PhD
Department of Ophthalmology and Visual Sciences
PFP 11290C
University of Iowa Hospitals and Clinics
200 Hawkins, Iowa City, IA 52242
Tel (319) 384 5833, fax: (319) 353 7996

[redacted]

[redacted]

Patient questionnaire

BEFORE telediagnosis

Name: [redacted] **Date** [redacted]

Thank you for your willingness to participate in this research project.

You are about to undergo digital photographing and diagnosis over the internet by ophthalmologists who are retinal specialists at the University of Iowa. They will evaluate your photographs for signs of diabetic retinopathy, a complication of diabetes. This new technique is called 'telediagnosis'. Until telediagnosis was available, you could only be screened in an ophthalmologist's office with a dilated retinal exam.

Please give your best answer to the following questions

Circle where you expect the quality of the retinal exam to be better:

1 2 (3) 4 5

Ophthalmologist's office — Telediagnosis

Circle which retinal exam you prefer:

1 2 (3) 4 5

Ophthalmologist's office — Telediagnosis

- ☑ yes □ no Do you expect the camera flashlight to bother you
- ☑ yes □ no Do you expect the pupil dilating drops to bother you
- ☑ yes ☒ no In the past five years have you been examined by an ophthalmologist
- ☑ yes □ no Do you currently carry health insurance
- ☑ yes □ no In some cases the photographs can only be made if your pupils are dilated with a drop. Were you aware of this?

Patient questionnaire

AFTER telediagnosis results have been discussed

Name:____________________________ **Date:**______________

Circle your satisfaction with digital photography of your retina

1 2 3 4 (5)

Terrible — Very good

Circle where you think the retinal exam is better:

1 2 3 4 (5)

Ophthalmologist's office — Telediagnosis

Circle which retinal exam you prefer now:

1 2 3 4 (5)

Ophthalmologist's office — Telediagnosis

- □ yes ☒ no Did the camera flashlight bother you
- □ yes ☒ no Did the pupil dilating drops bother you

After form is completed, please enter into Excel spreadsheet or send to:

Dr. Michael D. Abramoff, MD, PhD
Department of Ophthalmology and Visual Sciences
PFP 11290C
University of Iowa Hospitals and Clinics
200 Hawkins, Iowa City, IA 52242
Tel (319) 384 5833, fax: (319) 353 7996

[redacted]

m

[redacted]

Patient Questionnaire

Name [redacted] Date [redacted]

BEFORE telediagnosis

Thank you for your willingness to participate in this research project.

You are about to undergo digital photographing and diagnosis over the internet by ophthalmologists who are retinal specialists at the University of Iowa. They will evaluate your photographs for signs of diabetic retinopathy, a complication of diabetes. This new technique is called 'telediagnosis'. Until telediagnosis was available, you could only be screened in an ophthalmologist's office with a dilated retinal exam. Please give your best answer.

What number best expresses where you think the *quality* of the retinal exam is best:

1	2	3	4	5
(Better in ophthalmologist's office)	(2) circled	undecided		(Better through Telediagnosis)

What number best expresses *where* you prefer to have the retinal exam:

1	2	3	4	5
(Prefer ophthalmologist's office)	(2) circled	undecided		(Prefer family physician)

☑ yes ☐ no Do you expect the camera flashlight to bother you?

☑ yes ☐ no Do you expect the pupil dilating drops to bother you?

☑ yes ☐ no In the past five years have you been examined by an ophthalmologist?

☑ yes ☐ no Do you currently carry health insurance?

☑ yes ☐ no Sometimes photographs can only be made if your pupils are made larger with a drop. Were you aware of this?

AFTER telediagnosis

What number best expresses your *satisfaction* with telediagnosis:

1	2	3	4	5
(Terrible)	(2) circled	undecided		(Very good)

What number best expresses your where you think the *quality* of the retinal exam is best::

1	2	3	4	5
(Better in ophthalmologist's office)	(2) circled	undecided		(Better through Telediagnosis)

What number best expresses *where* you prefer to have the retinal exam <u>now</u>:

1	2	3	4	5
(Prefer ophthalmologist's office)	(2) circled	undecided		(Prefer family physician)

☑ yes ☐ no Did the camera flashlight bother you?

☑ yes ☐ no Did the pupil dilating drops bother you? ☐ not applicable

[redacted]

[redacted]

Patient questionnaire

BEFORE telediagnosis

Name: [redacted] **Date:** [redacted]

Thank you for your willingness to participate in this research project.

You are about to undergo digital photographing and diagnosis over the internet by ophthalmologists who are retinal specialists at the University of Iowa. They will evaluate your photographs for signs of diabetic retinopathy, a complication of diabetes. This new technique is called 'telediagnosis'. Until telediagnosis was available, you could only be screened in an ophthalmologist's office with a dilated retinal exam.

Please give your best answer to the following questions

Circle where you expect the quality of the retinal exam to be better:

1	2	3	4	5
Ophthalmologist's office			Telediagnosis	

Circle which retinal exam you prefer:

1	2	3	4	5
Ophthalmologist's office			Telediagnosis	

□ yes □ no Do you expect the camera flashlight to bother you
□ yes □ no Do you expect the pupil dilating drops to bother you
□ yes □ no In the past five years have you been examined by an ophthalmologist
□ yes □ no Do you currently carry health insurance
□ yes □ no In some cases the photographs can only be made if your pupils are dilated with a drop. Were you aware of this?

Patient questionnaire

AFTER telediagnosis results have been discussed

Name:____________________ **Date:**____________

Circle your satisfaction with digital photography of your retina

1	2	3	4	5
Terrible				Very good

Circle where you think the retinal exam is better:

1	2	3	4	5
Ophthalmologist's office				Telediagnosis

Circle which retinal exam you prefer now:

1	2	3	4	5
Ophthalmologist's office				Telediagnosis

□ yes □ no Did the camera flashlight bother you
□ yes □ [redacted] pupil dilating drops bother you

After form is completed, please enter into Excel spreadsheet or send to:

Dr. Michael D. Abramoff, MD, PhD
Department of Ophthalmology and Visual Sciences
PFP 11290C
University of Iowa Hospitals and Clinics
200 Hawkins, Iowa City, IA 52242
Tel (319) 384 5833, fax: (319) 353 7996

[redacted]

Patient questionnaire

BEFORE telediagnosis

Name: [redacted] **Date** [redacted]

Thank you for your willingness to participate in this research project.
You are about to undergo digital photographing and diagnosis over the internet by ophthalmologists who are retinal specialists at the University of Iowa. They will evaluate your photographs for signs of diabetic retinopathy, a complication of diabetes. This new technique is called 'telediagnosis'. Until telediagnosis was available, you could only be screened in an ophthalmologist's office with a dilated retinal exam.

Please give your best answer to the following questions

Circle where you expect the quality of the retinal exam to be better:

1 Ophthalmologist's office 2 3 4 Telediagnosis 5 [redacted]

Circle which retinal exam you prefer:

1 Ophthalmologist's office 2 3 4 Telediagnosis 5 [redacted]

☑ yes ☐ no Do you expect the camera flashlight to bother you [redacted]
☐ yes ☑ no Do you expect the pupil dilating drops to bother you
☑ yes ☐ no In the past five years have you been examined by an ophthalmologist
☑ yes ☐ no Do you currently carry health insurance
☑ yes ☐ no In some cases the photographs can only be made if your pupils are dilated with a drop. Were you aware of this?

Patient questionnaire

AFTER telediagnosis results have been discussed

Name:____________________ **Date:**__________

Circle your satisfaction with digital photography of your retina

1 Terrible 2 3 4 5 Very good

Circle where you think the retinal exam is better:

1 Ophthalmologist's office 2 3 4 5 Telediagnosis

Circle which retinal exam you prefer now:

1 Ophthalmologist's office 2 3 4 5 Telediagnosis

☑ yes ☐ no Did the camera flashlight bother you [redacted]
☐ yes ☑ no Did the pupil dilating drops bother you

After form is completed, please enter into Excel spreadsheet or send to:

Dr. Michael D. Abramoff, MD, PhD
Department of Ophthalmology and Visual Sciences
PFP 11290C
University of Iowa Hospitals and Clinics
200 Hawkins, Iowa City, IA 52242
Tel (319) 384 5833, fax: (319) 353 7996

[redacted]

[redacted]

Patient questionnaire

BEFORE telediagnosis

Name:[redacted] **Date:**[redacted]

Thank you for your willingness to participate in this research project.

You are about to undergo digital photographing and diagnosis over the internet by ophthalmologis[redacted] retinal specialists at the University of Iowa. They will evaluate your photographs for signs of diabetic retinopathy, a complication of diabetes. This new technique is called 'telediagnosis'. Until telediagnosis was available, you could only be screened in an ophthalmologist's office with a dilated retinal exam.

Please give your best answer to the following questions

Circle where you expect the quality of the retinal exam to be better:

1	2	3	4	5
Ophthalmologist's office			Telediagnosis	

Circle which retinal exam you prefer:

1	2	3	4	5
Ophthalmologist's office			Telediagnosis	

□ yes ☒ no Do you expect the camera flashlight to bother you
□ yes ☒ no Do you expect the pupil dilating drops to bother you
☒ yes □ no In the past five years have you been examined by an ophthalmologist
☒ yes □ no Do you currently carry health insurance
□ yes ☒ no In some cases the photographs can only be made if your pupils are dilated with a drop. Were you aware of this?

Patient questionnaire

AFTER telediagnosis results have been discussed

Name:______________________________ **Date:**_______________

Circle your satisfaction with digital photography of your retina

1	2	3	4	5
Terrible				Very good

Circle where you think the retinal exam is better:

1	2	3	4	5
Ophthalmologist's office				Telediagnosis

Circle which retinal exam you prefer now:

1	2	3	4	5
Ophthalmologist's office				Telediagnosis

☒ yes □ no Did the camera flashlight bother you
□ yes ☒ no Did the pupil dilating drops bother you

After form is completed, please enter into Excel spreadsheet or send to:

Dr. Michael D. Abramoff, MD, PhD
Department of Ophthalmology and Visual Sciences
PFP 11290C
University of Iowa Hospitals and Clinics
200 Hawkins, Iowa City, IA 52242
Tel (319) 384 5833, fax: (319) 353 7996

[redacted]

Patient questionnaire

BEFORE telediagnosis

Name: [redacted] **Date:** [redacted]

Thank you for your willingness to participate in this research project.

You are about to undergo digital photographing and diagnosis over the internet by ophthalmologists who are retinal specialists at the University of Iowa. They will evaluate your photographs for signs of diabetic retinopathy, a complication of diabetes. This new technique is called 'telediagnosis'. Until telediagnosis was available, you could only be screened in an ophthalmologist's office with a dilated retinal exam.

Please give your best answer to the following questions

Circle where you expect the quality of the retinal exam to be better:

1 2 (3) 4 5

Ophthalmologist's office — Telediagnosis

Circle which retinal exam you prefer:

1 2 (3) 4 5

Ophthalmologist's office — Telediagnosis

□ yes ☑ no Do you expect the camera flashlight to bother you

☑ yes □ no Do you expect the pupil dilating drops to bother you

☑ yes □ no In the past five years have you been examined by an ophthalmologist

☑ yes □ no Do you currently carry health insurance

☑ yes □ no In some cases the photographs can only be made if your pupils are dilated with a drop. Were you aware of this?

Patient questionnaire

AFTER telediagnosis results have been discussed

Name:____________________ **Date:**__________

Circle your satisfaction with digital photography of your retina

1 2 3 4 (5)

Terrible — Very good

Circle where you think the retinal exam is better:

1 2 3 4 (5)

Ophthalmologist's office — Telediagnosis

Circle which retinal exam you prefer now:

1 2 3 4 (5)

Ophthalmologist's office — Telediagnosis

□ yes ☑ no Did the camera flashlight bother you

□ yes □ no Did the pupil dilating drops bother you [redacted]

After form is completed, please enter into Excel spreadsheet or send to:

Dr. Michael D. Abramoff, MD, PhD
Department of Ophthalmology and Visual Sciences
PFP 11290C
University of Iowa Hospitals and Clinics
200 Hawkins, Iowa City, IA 52242
Tel (319) 384 5833, fax: (319) 353 7996

[redacted]

[redacted]

Patient questionnaire

BEFORE telediagnosis

Name:[redacted]**Date:**[redacted]

Thank you for your willingness to participate in this research project.

You are about to undergo digital photographing and diagnosis over the internet by ophthalmologists who are retinal specialists at the University of Iowa. They will evaluate your photographs for signs of diabetic retinopathy, a complication of diabetes. This new technique is called 'telediagnosis'. Until telediagnosis was available, you could only be screened in an ophthalmologist's office with a dilated retinal exam.

Please give your best answer to the following questions

Circle where you expect the quality of the retinal exam to be better:

1 2 3 4 5

Ophthalmologist's office — Telediagnosis

Circle which retinal exam you prefer:

1 2 3 4 5

Ophthalmologist's office — Telediagnosis

- ☐ yes ☒ no Do you expect the camera flashlight to bother you
- ☐ yes ☒ no Do you expect the pupil dilating drops to bother you
- ☐ yes ☒ no In the past five years have you been examined by an ophthalmologist
- ☒ yes ☐ no Do you currently carry health insurance
- ☐ yes ☒ no In some cases the photographs can only be made if your pupils are dilated with a drop. Were you aware of this?

Patient questionnaire

AFTER telediagnosis results have been discussed

Name:____________________ **Date:**__________

Circle your satisfaction with digital photography of your retina

1 2 3 4 5

Terrible — Very good

Circle where you think the retinal exam is better:

1 2 3 4 5

Ophthalmologist's office — Telediagnosis

Circle which retinal exam you prefer now:

1 2 3 4 5

Ophthalmologist's office — Telediagnosis

- ☐ yes ☒ no Did the camera flashlight bother you
- ☐ yes ☒ no Did the pupil dilating drops bother you

After form is completed, please enter into Excel spreadsheet or send to:

Dr. Michael D. Abramoff, MD, PhD
Department of Ophthalmology and Visual Sciences
PFP 11290C
University of Iowa Hospitals and Clinics
200 Hawkins, Iowa City, IA 52242
Tel (319) 384 5833, fax: (319) 353 7996

[redacted]
